# Exploring the Reactivity of Rigid 1-Azadienes Derived from Methylene γ-Lactams. Applications to the Stereoselective Synthesis of Spiro-γ-Lactams

**DOI:** 10.1021/acs.joc.4c00822

**Published:** 2024-06-19

**Authors:** Adrián López-Francés, Zuriñe Serna-Burgos, Xabier del Corte, Jesús M. de los Santos, Abel de Cózar, Javier Vicario

**Affiliations:** † Department of Organic Chemistry I, Faculty of Pharmacy, University of the Basque Country, UPV/EHU. Paseo de la Universidad 7, Vitoria-Gasteiz 01006, Spain; ‡ Department of Organic Chemistry I, Donostia International Physics Centre (DIPC), University of the Basque Country, UPV/EHU. Paseo Manuel de Lardizabal, 3, Donostia-San Sebastián 20018, Spain; § Ikerbasque, Basque Foundation for Science, Plaza Euskadi 5, Bilbao 48009, Spain

## Abstract

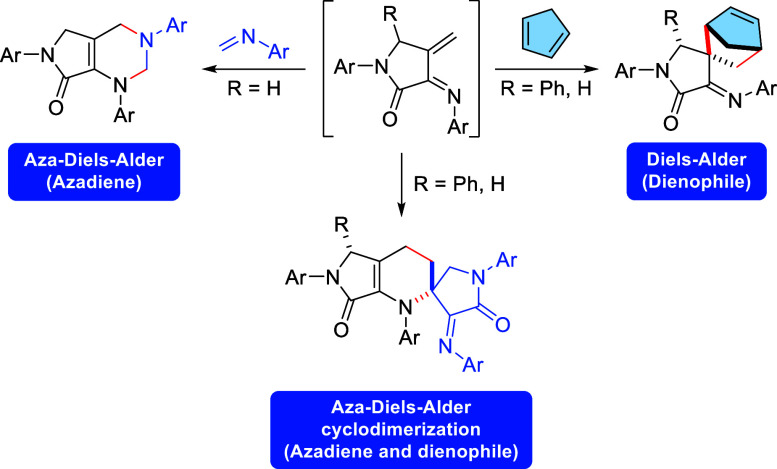

A study on the reactivity of rigid 1-azadienes derived from methylene γ-lactams is reported. Through the functionalization of 1-amino α,β-unsaturated γ-lactam derivatives, easily available from a multicomponent reaction of amines, aldehydes, and pyruvates, it is possible to in situ generate rigid 1-azadienes locked by a γ-lactam core. The 4π-electron system of those rigid 1-azadienes can behave as both diene and dienophile species through a spontaneous cyclodimerization reaction or exclusively as dienes or dienophiles if they are trapped with imines or cyclopentadiene, respectively. The use of chiral rigid 1-azadienes as dienophiles in the cycloaddition reaction with cyclopentadiene leads to the formation of spiro-γ-lactams bearing four stereogenic centers in a highly stereospecific manner, reporting the first example of the use of methylene-γ-lactams in the synthesis of spirocycles.

## Introduction

Spirocycles are a fascinating and essential class of chemical structures in organic chemistry.^1^ They are characterized by the presence of two or more rings that share a single common atom, creating a unique and intricate molecular arrangement. Owing to their unique three-dimensional architecture and favorable physicochemical attributes,^2^ spirocycles have found applications in various areas of organic chemistry, including drug discovery^3^ and natural product synthesis.^4^ The distinct geometry of spirocycles imparts steric rigidity, which can enhance their stability and resistance to degradation, rendering them valuable scaffolds in medicinal chemistry. Numerous biologically active compounds, such as antioxidants, antibiotics, antidiabetic and antiviral agents, and contraceptive and anticancer drugs, feature spirocyclic motifs in their structures, providing improved pharmacokinetic properties and target specificity.^5^ The ability to modulate the spatial arrangement and electronic properties of spirocycles further contributes to their role as privileged structures in drug design.^3d,6^

**Figure 1:**
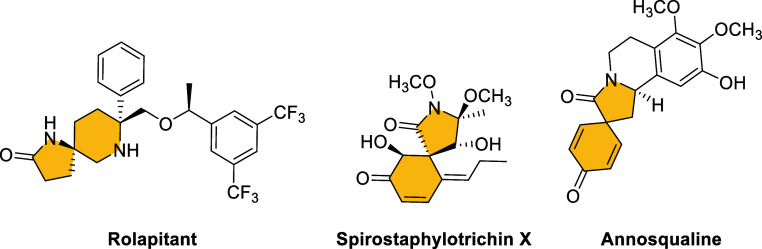
Structure of some relevant γ-spirolactams.

Within this family of compounds, spirolactams are spirocyclic structures in which a quaternary bridgehead center is contained within a lactam ring. In particular, spirocycles containing γ-lactam structures can be found in a wide range of natural products, pharmaceuticals, and biologically active compounds. Interestingly, many of these compounds exhibit important biological activities, such as antiemetic Rolapitant,^7a^ Spirostaphylotrichin X, an anti-influenza agent isolated from a marine-derived fungus,^7b^ or the isoquinoline-cored alkaloid Annosqualine isolated in 2004 from the stem of *Annona squamosa*(Figure 1).^7c^

The construction of all classes of spirocycles involves the creation of a quaternary center, which is itself a significant challenge in synthetic organic chemistry.^8^ In this regard, among the innumerable established approaches for the construction of 6-membered cyclic structures, the Diels–Alder cycloaddition and its analogous reactions, where one of the carbon atoms of the diene or the dienophile is replaced by a heteroatom, are some of the most efficient methods, leading to the formation of substituted spirocyclic derivatives.^9^ The use of the Diels–Alder reaction as a tool for the synthesis of 6-membered spirocyclic compounds obviously requires the presence of an exocyclic C=C double bond at the (hetero)diene or (hetero)dienophile species in order to create the quaternary bridgehead center at the fusion point of both rings of the final structure.

In this context, there are some limited examples that illustrate the use of methylene-γ-lactones as dienophiles in intramolecular^10^ or intermolecular^11^ Diels–Alder reactions for the synthesis of spiro-γ-lactones, most of them reported as single examples of general methodologies. However, as far as we are concerned, there are no described examples in the literature where the analogous methylene-γ-lactams are used as substrates in [4 + 2] processes leading to spiro-γ-lactams.

In the past, we have reported an efficient synthesis of 3-amino unsaturated γ-lactam derivatives through a Brønsted acid-catalyzed multicomponent reaction between amines, aldehydes, and pyruvate derivatives.^12^ Key features of the structure of those substrates are the presence of a reactive endocyclic enamine moiety embedded in a chiral environment and, taking the advantage of those two attributes, we have used these γ-lactam substrates in diverse stereoselective reactions.^12c,13^ Particularly, those substrates were found to be very adequate for the generation of rigid 1-azadienes and, very recently, we have reported a bispericyclic cyclodimerization reaction of chiral 1-azadienes derived from methylene-γ-lactams, leading to complex γ-spirolactam derivatives bearing two γ-lactam cores and a dihydropyridine ring.^14^ Remarkably, in this report, although a racemic mixture of chiral azadienes is used, a single diastereomer is obtained instead of the expected statistical mixture, postulating a strong chiral self-recognition phenomenon in the cycloaddition process, associated with a combination of stabilizing electrostatic and dispersion interaction energies. As part of our ongoing pursuit in the development of new methodologies for the construction of scaffolds found in drug structures, we decided to explore the potential of the Diels–Alder reaction using methylene-γ-lactam-derived rigid 1-azadienes in the creation of novel spirocyclic systems. For all the reasons mentioned above, herein, we report a study on the reactivity of rigid 1-azadienes derived from methylene-γ-lactams and their applications to the stereoselective synthesis of novel γ-spirolactams.

## Results and Discussion

**Scheme 1:**
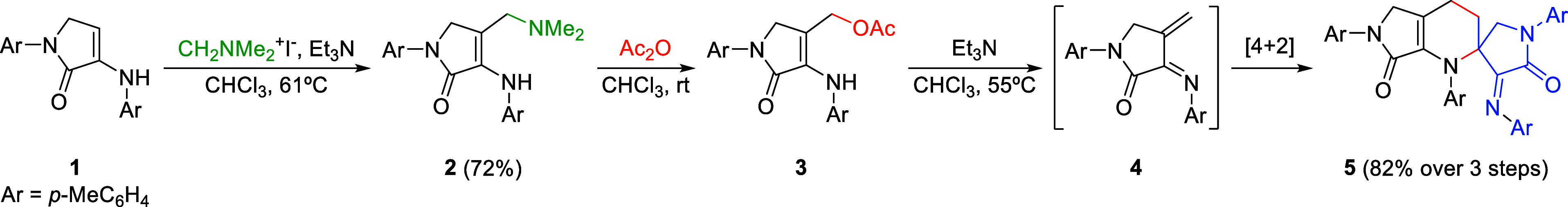
Synthetic Protocol for the Generation of Rigid 1-Azadiene **4** and Its Spontaneous Cyclodimerization Reaction

Initially, following a known procedure,^12^ the starting 3-amino γ-lactam derivative **1** was prepared through a multicomponent protocol consisting of the reaction of formaldehyde, *p*-toluidine, and ethyl pyruvate in the presence of a Brønsted acid catalyst (see the Supporting Information). Next, the functionalization of substituted γ-lactam substrate **1** with Eschenmoser’s salt was accomplished in very good yield in the presence of triethylamine using refluxing chloroform as a solvent (Scheme 1). Attempting to prepare 1-azadiene species from substrate **2** via direct elimination of trimethylamine by traditional methods using methyl iodide or Me_2_SO_4_ did not result in the target azadiene product and, for this reason, the dimethylamino moiety was replaced by an acetoxy group by stirring functionalized γ-lactam **2** in neat acetic anhydride at room temperature, leading instantaneously to substituted γ-lactam **3**. Owing to the quick decomposition of substrate **3** under exposure to silica or alumina, the isolation of a pure sample by chromatography was unachievable. For this reason, acetoxy-substituted γ-lactam **3** was used in the next step without further purification.

Next, in order to promote the elimination of acetic acid and the formation of the target azadiene, substrate **3** was treated under basic conditions in the presence of triethylamine in heated chloroform. However, under those reaction conditions, spiro-γ-lactam **5** was obtained as the sole reaction product. In congruence with our previous research, we theorized that bicyclic spiro-γ-lactam **5** might be formed through the initial formation of rigid 1-azadiene **4**, followed by a fast [4 + 2] cyclodimerization reaction (Scheme 1). Indeed, this fast dimerization reaction has been attributed to the formation of dimer aggregates of the starting acetylated γ-lactam units **3** in solution prior to the formation of the azadiene species **4**. The computational studies show that γ-lactam substrates **3** are strongly associated by means of two reciprocal π-stacking interactions between the two aromatic substituents at the enamine and the lactamic nitrogen, leading to a bispericiclic transition state, where the concept “hermaphroditism of molecules” was proposed for such behavior.^14^

Considering this, we theorized that the formation of the dimer aggregate could be prevented in the presence of an excess of a reagent with a similar affinity in solution for γ-lactam substrates **3**. This would allow avoiding the intrinsic dimerization reaction, thus leading to the reaction of the 1-azadiene moiety with other substrates rather than with itself. With this concept in mind and in order to further extend the synthetic applications of rigid 1-azadiene **4**, next, we tried to capture the in situ-generated substrate **4** by the generation of the 4π-electron system in the presence of different dienophile species. The specific procedure involved the treatment of the acetylated substrate **3** with triethylamine in the presence of a dienophile. Since 1-azadiene **4** is, in principle, an electron-poor 4π-electron system, the inverse electron demand Diels–Alder reaction was initially studied using electron-rich dienophiles. However, the presence of various enamines or enols as dienophiles during the generation of 1-azadiene **4** did not lead to the expected reaction, and only the formation of dimer **5** was observed.

In view of the very strained structure expected in substrate **4**, we thought that, maybe due to the rigidity of the cyclic structure, the amide carbonyl at the γ-lactam ring may be pushed out from the planarity, thus inhibiting the conjugation with the 4π-system, which may make the electron-withdrawing effect of such substituent ineffective. This is in agreement with the crystal structure reported for similar substrates.^12c,14^ For this reason, next, we generated the 1-azadiene species **4** in the presence of electron-poor dienophiles, such as dimethyl acetylenedicarboxylate, methyl acrylate, or maleic anhydride. However, under those conditions, no aza-Diels–Alder product was obtained, and only dimerization reaction was again observed.

**Scheme 2:**
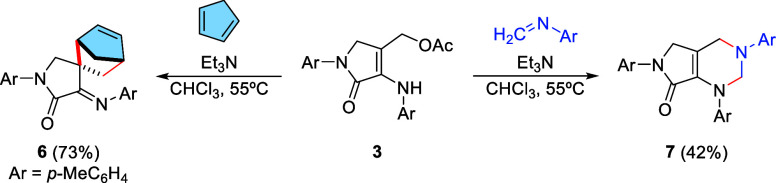
Reaction of In Situ-Generated 1-Azadienes with Cyclopentadiene and *N*-*p*-Tolyl-methanimine

Still hoping that our theory regarding the deactivation of the conjugation was nonfictional, the reaction was studied using simple alkenes as 2π-electron systems. We were disappointed to discover that the use of styrene, cyclopentene, cyclohexene, or indene in the reaction did not provide the expected aza-Diels–Alder substrate, resulting once again only in the formation of dimeric compound **5**. However, when the generation of 1-azadiene **4** was carried out in the presence of cyclopentadiene, a new product was observed, whose molecular formula matched the sum of both starting products. Although the result seemed to indicate an aza-Diels–Alder reaction, where cyclopentadiene (CpH) acted as the dienophile, a careful examination of the spectroscopic data led to the conclusion that what actually occurred was a [4 + 2] cycloaddition reaction, where cyclopentadiene acted as the 4π-electron system, while the conjugated double bond of 1-azadiene **4** acted as the dienophile, resulting in the formation of spirocyclic γ-lactam **6** with good yield and as a single diastereomer (Scheme 2).

Following with our quest on the investigation of the reactivity of 1-azadiene **4** and, still keeping our hopes that substrate **4** could act as 4π-electron system in a cycloaddition reaction, we performed a careful check of all the trace products obtained in the reactions. In fact, we could determine in the crude the presence of an almost undetectable trace that we believed could come from the aza-Diels–Alder reaction of 1-azadiene species with an imine substrate resultant from some impurities in the preparation of starting γ-lactam substrates **1**. Therefore, we carried out the generation of 1-azadiene **4** from acetylated substrate **3** in the presence of trietylamine and *N*-*p*-tolyl-methanimine as the aza-2π-electron system. To our delight, under those reaction conditions, the selective formation of a 1,3-pyrimidine ring was observed through a process that we assumed to proceed through a [4 + 2] cycloaddition reaction where 1-azadiene species **4** acted as the 4π-electron system, leading to the formation of bicyclic substrate **7** in good yield (Scheme 2).

**Scheme 3:**
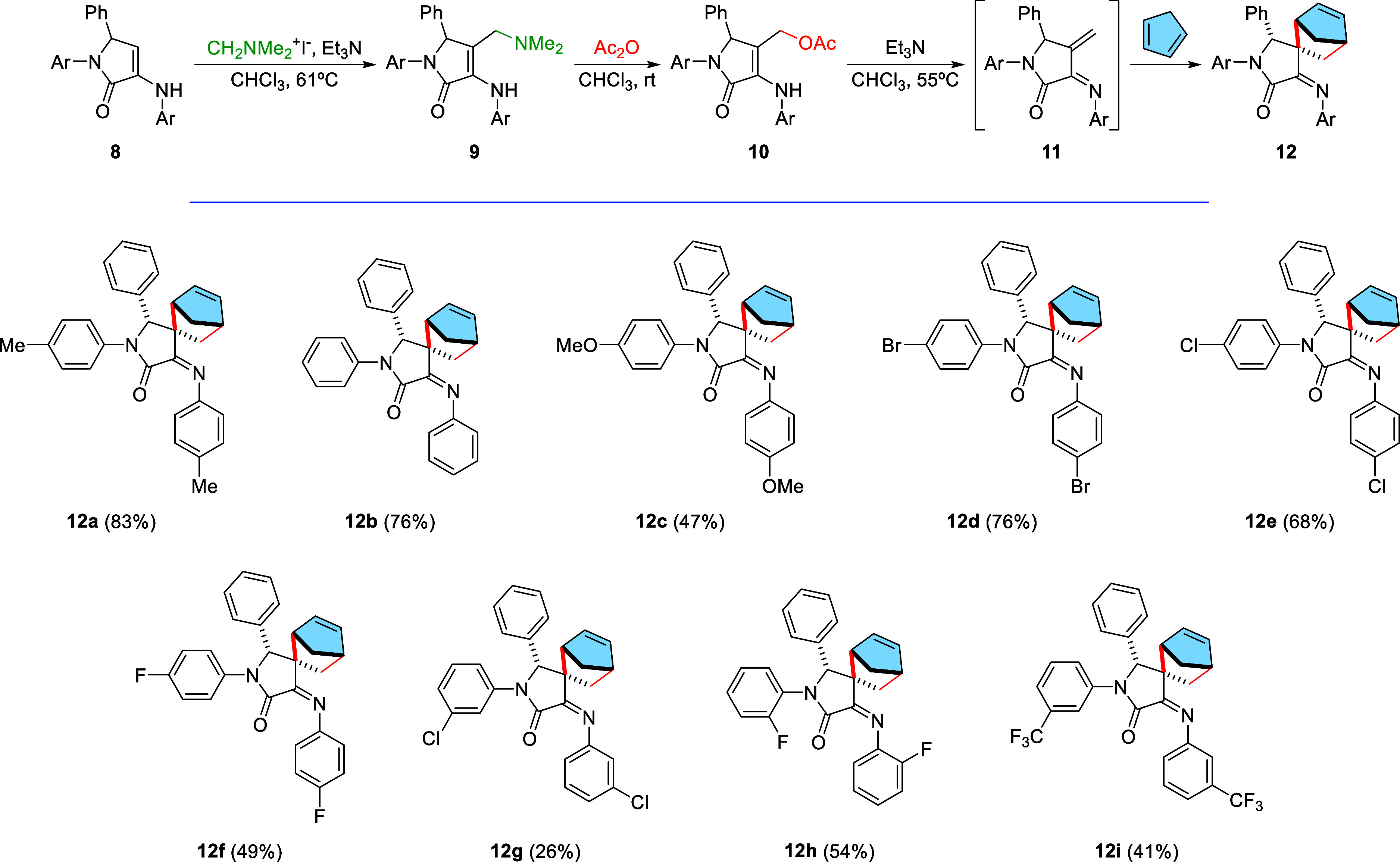
Substrate Scope of 1-Azadienes **11** in the Stereoselective Diels–Alder Reaction with Cyclopentadiene

In the next stage, due to the easiness for the preparation of starting γ-lactam substrates through a multicomponent reaction,^12^ we extended our research to the investigation of the reactivity and stereoselectivity of rigid 1-azadienes generated from chiral substrates. As in the previous case, dimethylaminomethyl-substituted γ-lactams **9** were prepared by the reaction of γ-lactams **8** with Eschenmoser’s salt in the presence of trimethylamine as a base and using refluxing chloroform as a solvent. Next, the dimethylamino group in **9** was replaced by an acetoxy group upon treatment with neat acetic anhydride. The generation of 1-azadienes **11** with trimethylamine in heated chloroform leads to the cyclodimerization products **5′** (see the Supporting Information) analogous to substrate **5** (see Scheme 1) as expected, due to the prior formation of the dimer aggregate of substrates **10** in solution.^14^ Following the successful method described previously, for the aza-Diels–Alder reaction, it implies the generation of the 1-azadiene species **11** in the presence of an excess of *N*-*p*-tolyl-methanimine did not provide the cycloaddition product and only the presence of cyclodimerization substrate was detected by nuclear magnetic resonance (NMR). However, under similar conditions, in the presence of an excess of cyclopentadiene, spiro-γ-lactams **12** were obtained as single diastereomers through a highly stereoselective Diels–Alder reaction (Scheme 3).

In view of the interest of the spiro-γ-lactam substrates obtained and the high stereoselectivity observed in the process, we extended the scope of the reaction to the use of differently substituted γ-lactam substrates **10**. Besides the model reaction starting from *p*-toluidine-derived γ-lactam **10a** (Ar = *p*-MeC_6_H_4_), the reaction proceeds with good yield and the same degree of stereoselectivity when using the substrate **10b** (Ar = Ph), derived from simple aniline. However, the use of functionalized γ-lactam **10c** (Ar = *p*-MeOC_6_H_4_), derived from an electron-rich aniline such as *p*-anisidine, resulted in a decrease in the reaction yield. The reaction was also applied to the use of substrates derived from *para*-halogen-substituted anilines **10d**–**f** (Ar = *p*-BrC_6_H_4_, *p*-ClC_6_H_4_, *p*-FC_6_H_4_), obtaining spiro-γ-lactams **12d**–**f** in good yields. Likewise, a slight drop in the reaction yields was observed when using γ-lactams **10g**–**h** (Ar = *m*-ClC_6_H_4_, *o*-FC_6_H_4_), derived from *m*-chloroaniline and *o*-fluoroaniline. Finally, the use of γ-lactam **10i** (Ar = *m*-CF_3_C_6_H_4_), derived from *m*-trifluoromethylaniline, also led to the cycloaddition product **12i**, although with a moderate yield (Scheme 3).

As usual, the substrates **12**, resulting from the [4 + 2] cycloaddition reaction, were characterized based on their spectroscopic data and high-resolution mass spectroscopy (HRMS). In the ^1^H NMR spectrum of compound **12a**, the most characteristic chemical shifts correspond to the two protons of the C=C double bond in the norbornene unit at δ_H_ = 6.55 and 6.42 ppm, appearing as two double doublets with a reciprocal coupling constant ^3^*J*_HH_ = 5.7 Hz, typical for a double bond in a *cis* configuration, and both showing an identical coupling constant ^3^*J*_HH_ = 3.1 Hz with the bridging CH groups. The four diastereotopic protons of the two methylene group of norbornene unit in **12a** appear as one complex multiplet in the range δ_H_ = 2.22–2.13 ppm, for two of them, a second multiplet integrating one proton at δ_H_ = 1.48 ppm and a clear double doublet for the fourth proton at δ_H_ = 0.84 ppm, with a geminal coupling constant ^2^*J*_HH_ = 12.4 Hz and a second vicinal coupling constant ^3^*J*_HH_ = 2.9 Hz. The two CH groups of norbornene appear at δ_H_ = 2.89 and 3.12 ppm. Due to the weak and poorly resolved coupling with the neighboring protons, both signals appear as two broad singlets. Finally, the CH of the asymmetric carbon belonging to the γ-lactam core appears as a singlet at δ_H_ = 4.85 ppm.

Regarding the ^13^C NMR spectrum of spiro-γ-lactams **12**, the most characteristic chemical shifts for compound **12a** are those corresponding to the γ-lactam ring, which appear at δ_C_ = 165.3 and 159.0 ppm, typical for an amide carbonyl and an imine, respectively, both within a cycle, the quaternary carbon at δ_C_ = 58.1 ppm, and the CH of the asymmetric carbon at δ_C_ = 69.6 ppm. Additionally, the presence of the norbornene ring is inferred by the presence of the two olefinic CH groups at δ_C_ = 142.6 and 134.3 ppm, the two aliphatic CH carbons at δ_C_ = 51.9 and 43.0 ppm, and the two methylene groups at δ_C_ = 46.1 and 34.9 ppm. The multiplicity of the signals in the ^13^C NMR spectrum was confirmed through distortionless enhanced polarization transfer (DEPT) and heteronuclear single quantum coherence (HSQC) experiments.

**Figure 2:**
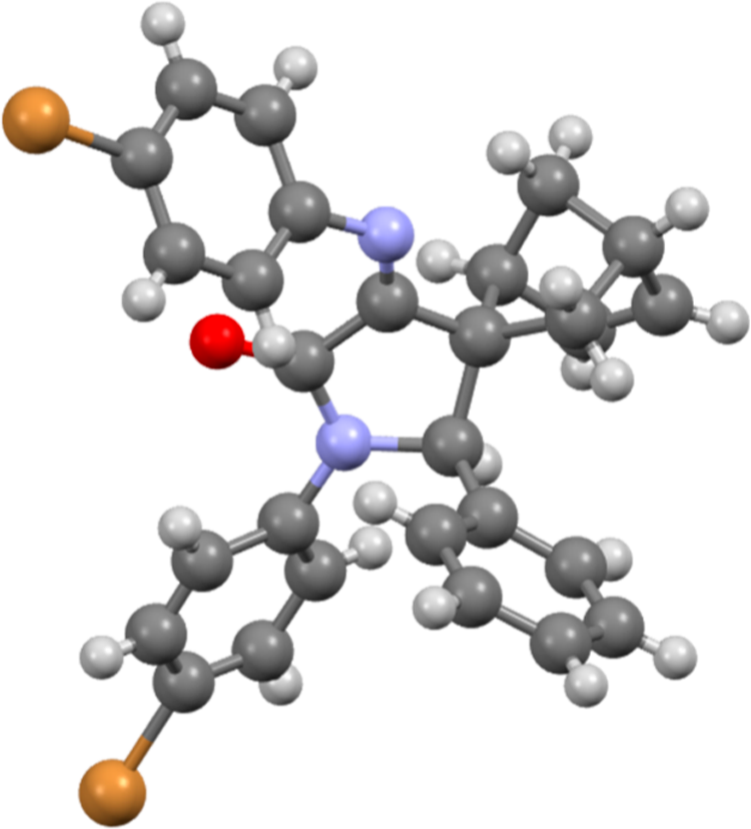
X-ray structure of spiro-γ-lactam **12d** (H, white; C, gray; O, red; N, blue; Br, orange) (1*R*,2*S*,2′*S*,4*R* enantiomer shown).

It is worth noting that a multigram scale reaction was also performed starting from 2.17 g (4 mmol) of γ-lactam **9d**, leading to 1.46 g of spiro-γ-lactam **12d** in 65% yield. Taking the advantage of this reaction and, in order to unambiguously elucidate the structure of the substrates obtained in the cycloaddition reaction as well as the relative configuration of the stereocenters, a single crystal of spirocyclic γ-lactam **12d** was isolated. The X-ray structure of **12d** revealed a relative configuration 1R*,2S*,2′S*,4R* for the four stereocenters of the final substrate (Figure 2). According to this configuration, an *endo* stereospecific transition state is proposed for the cycloaddition reaction, where the diene species approaches from the less hindered face, that is, the opposite to the phenyl group at the chiral carbon, which leads to the formation of one exclusive diastereomer bearing four stereocenters.

Intrigued by the results obtained through the reaction of azadienes **11** in the presence of CpH, where exclusive formation of cycloadduct **12** was observed, we decided to carry out a density functional theory (DFT) mechanistic study in order to shed some light on how CpH prevents the azadiene dimerization. With this purpose, we initially evaluated the possible interaction between the acetylated precursor **10** and CpH by means of binding free energies as outlined below
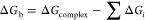
1where Δ*G*_*i*_ indicates the free energy of the isolated species.

In a previous investigation, it was demonstrated that analogous acetylated γ-lactams tend to aggregate due to strong π–π interaction (Δ*G*_b_ (**10d·10d**) of −12.7 kcal·mol^–1^ for the case of *p*-bromine-substituted aniline).^14^

**Figure 3:**
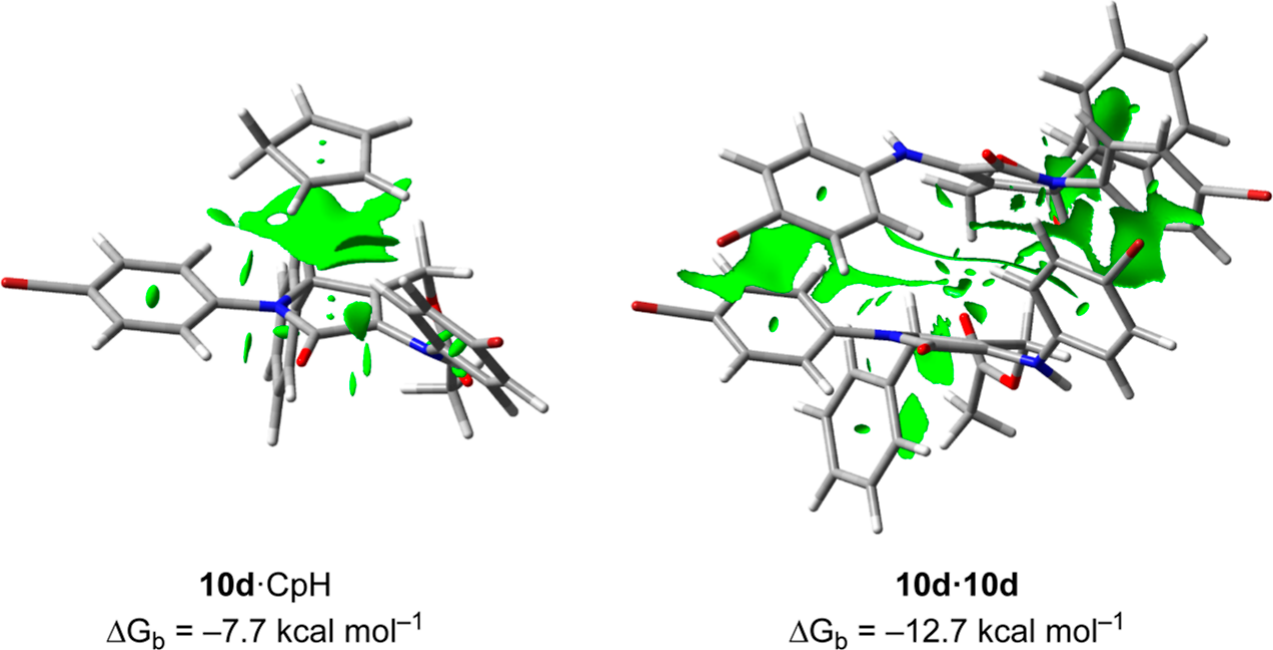
Gibbs binding free energies (Δ*G*_b_) and contour plots of the RGD isosurfaces (density cutoff = 0.20 au) of complexes **10d**·CpH and **10d**·**10d** computed at the M06-2X-GD3 (PCM)/6-31+G**//M06-2X-GD3 (PCM)/6-31G* level of theory. The green surfaces indicate attractive noncovalent interactions. Data of complex **10d**·**10d** taken from ref (14).

In the case under study, the calculated Δ*G*_b_ (**10d**·CpH) was −7.7 kcal·mol^–1^ (Figure 3). This result indicates that the π–π stacking interaction between **10d** and CpH favors **10d**·CpH formation. However, the computed CpH–**10d** interaction is weaker than the one obtained for **10d**·**10d** as reflected in the larger green surface observed in the computed reduced density gradient (RGD) plot (the more extensive surface is related with higher noncovalent interactions, Figure 3). Therefore, despite of the high excess of CpH (6 equiv), on the basis of these results, we could not ensure, in a theoretical manner, that CpH would be capable of preventing the **10d**·**10d** aggregation related to the dimeric spirocycle formation.

**Figure 4:**
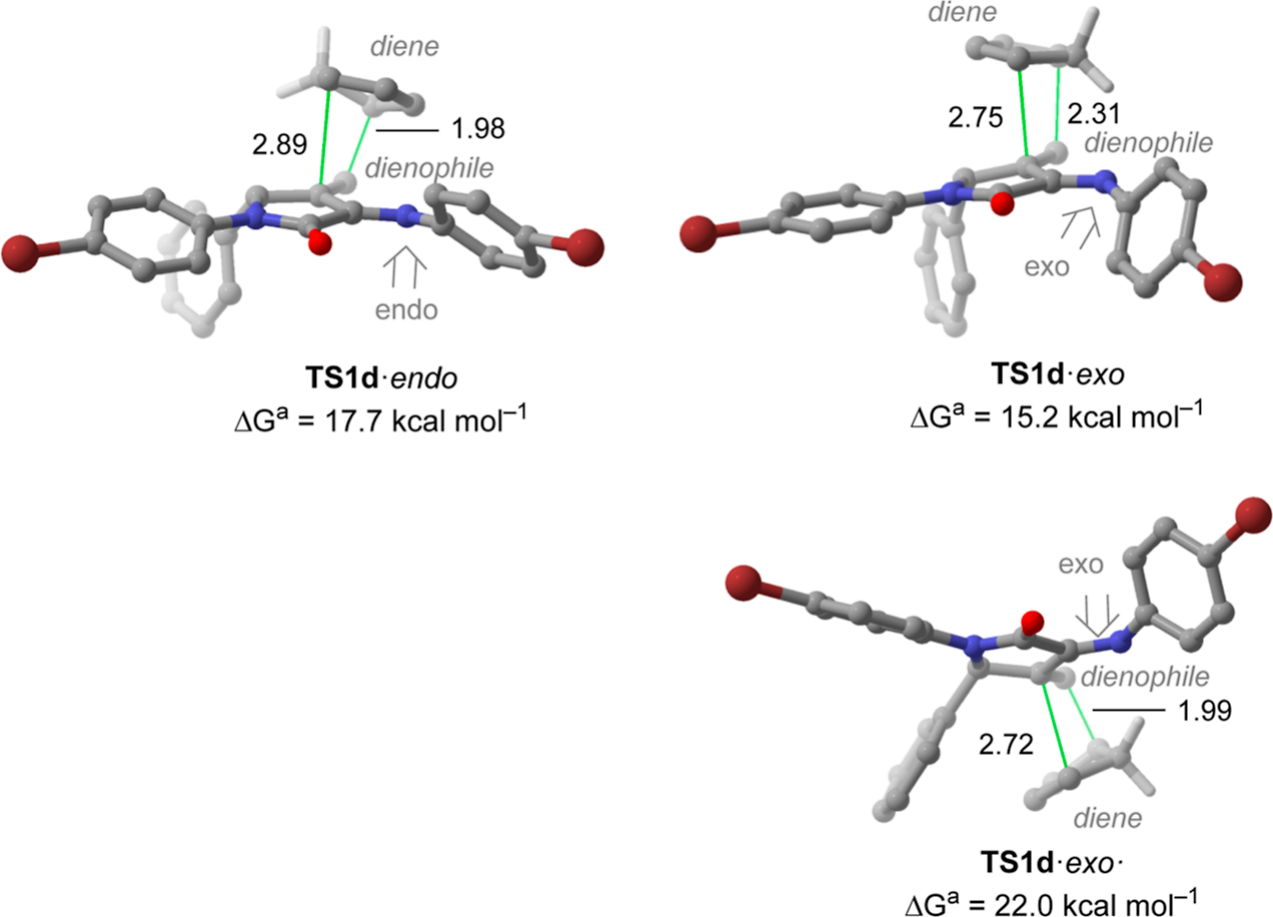
Main geometrical features and Gibbs activation energies computed for the less energetic transition structures associated with the [4 + 2] cycloaddition reaction of **11d** and CpH (CpH acting as a diene) computed at the M06-2X-GD3 (PCM)/6-31+G**//M06-2X-GD3 (PCM)/6-31G* level of theory. Distances are in Å.

**Figure 5:**
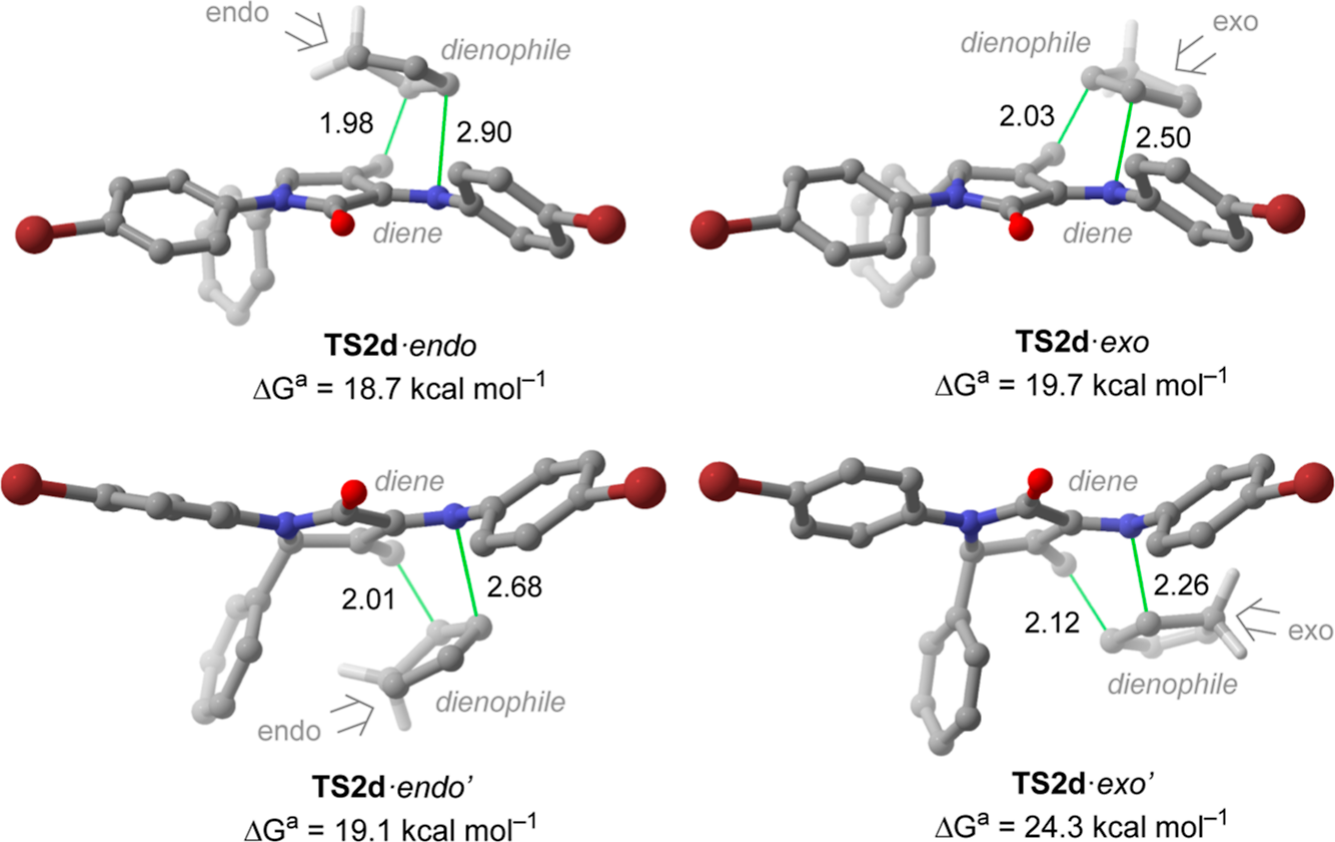
Main geometrical features and Gibbs activation energies computed for the less energetic transition structures associated with the [4 + 2] cycloaddition reaction of **11d** and CpH (CpH acting as a dienophile) computed at the M06-2X-GD3 (PCM)/6-31+G**//M06-2X-GD3 (PCM)/6-31G* level of theory. Distances are in Å.

Subsequently, we explored the energetic profiles related to the [4 + 2] cycloaddition between **11d** and CpH. In order to have a complete overview of the reaction, all the possible roles of both reagents were analyzed [i.e.,**11d** acting as both as diene (namely, **TS1**) and dienophile (denoted as **TS2**)]. In Figures 4 and 5 are collected the main geometrical features of the computed transition structures and the activation free energy barriers related to this reaction (see the Supporting Information for further details).

As far as azadiene **11d** acts as a dienophile, our calculations show that the less energetic transition structure is, as expected, the one in which CpH approaches the opposite side of the phenyl substituent of the γ-lactam ring in an *exo* fashion (Gibbs activation barrier of 15.2 kcal mol^–1^ for **TS1d**·*exo* in Figure 4). Geometrical inspection of **TS1d**·*exo* shows that it corresponds to a concerted but highly asynchronous process where the methylene C–C bond develops earlier than the other C–C bond. Furthermore, it was observed that the analogous *endo* TS (**TS1d**·*endo*) lies +2.5 kcal mol^–1^ above, probably due to a stronger stabilizing interaction between CpH and the γ-lactam ring, analogously to the one present on **10d**·CpH mentioned above (Figure 3). We also analyzed the profiles associated with the CpH reacting through the same side of the phenyl substituent. In this case, the computed activation barrier associated with an *exo* approach (**TS1d**·*exo*) is +6.8 kcal mol^–1^ upper than the less energetic transition state (**TS1d**·*exo*). We relate that phenomenon to the higher steric hindrance between the incoming CpH and the phenyl moiety. Unfortunately, all our attempts to isolate the analogous stationary point related with the *endo* attack by the phenyl face were unsuccessful, leading to a transition structure in which the γ-lactam acts as an azadiene in few optimization steps.

We were also able to isolate the stationary point associated with **11d** acting as an azadiene. In this context, we observed a rise in the energy barrier in all cases, for instance, the less energetic transition structure associated with this chemoselectivity (**TS2d**·*endo* in Figure 5) is 3.5 kcal mol^–1^ more energetic than **TS1d**·*exo*. It is worth mentioning that again the approaching of the CpH to the γ-lactam ring is favored from the opposite face of the phenyl moiety. The observed differences in the computed activation free energies indicate a strong preference toward the diastereoselective formation of **12d** through **TS1d**·*exo*, in perfect agreement with the experimental results.

Remarkably, the activation barrier of 15.2 kcal mol^–1^ associated with this cycloaddition is 0.8 kcal mol^–1^ lower than the one computed for the dimerization of **11d** (Δ*G*^a^ = 16.0 kcal mol^–1^).^14^ Therefore, this former process is kinetically favored, but mixtures of products would be expected on the basis of that energetic difference. We hypothesize that both, the lower free activation barrier of **TS1d**, when CpH acts as diene, and the high excess of CpH used, may compensate the higher preference toward acetylated **10d**·**10d** aggregation, thus favoring **12d** formation instead of the dimerization reaction.

## Conclusions

The presence of an endocyclic enamine group within γ-lactam derivatives, synthesized via a multicomponent reaction involving amines, aldehydes, and pyruvates, renders them highly advantageous as precursors for the generation of cyclic rigid 1-azadienes through their functionalization with Eschenmoser’s salt. The in situ-generated rigid 1-azadienes undergo a fast spontaneous cyclodimerization process, where the 1-azadiene moiety acts as both diene and dienophile species. However, it is possible to trap the dienic system, working exclusively as a diene species, in the presence of *N*-*p*-tolyl-methanimine, or as a dienophile, if an excess of cyclopentadiene is present during its generation. In addition, the utilization of chiral 1-azadienes as dienophiles in the cycloaddition reaction with cyclopentadiene leads to the formation of spiro-γ-lactams bearing four stereogenic centers in a highly stereospecific manner. DFT calculations indicate that this fact can be attributed to a combination of a lower activation barrier, with the use of excess of CpH, which prevents the dimer formation. As far as we are concerned, this represents the first example of a cycloaddition reaction leading to the formation of spiro-γ-lactams using methylene γ-lactams as starting materials.

## Experimental Section

### General Information

Solvents for extraction and chromatography were of technical grade. All solvents used in reactions were freshly distilled from appropriate drying agents before use. All other reagents were recrystallized or distilled as necessary. All reactions were performed under an atmosphere of dry nitrogen. Analytical thin-layer chromatography was performed with silica gel 60 F_254_ plates, and visualization was accomplished by UV light. ^1^H, ^13^C, and ^19^F NMR spectra were recorded at 25 °C on a Bruker Advance 400 (at 400, 101, and 282 MHz, respectively), and TMS was used as the internal standard for ^1^H and ^13^C and CFCl_3_ for the ^19^F nucleus. Coupling constants (*J*) are reported in Hertz to the nearest 0.1 Hz. Data for ^1^H NMR spectra are reported as follows: (chemical shift, multiplicity, coupling constant, integration). Multiplicity abbreviations are as follows: (s = singlet, d = doublet, t = triplet, q = quartet, m = multiplet) and br (broad). ^13^C NMR values were recorded with complete proton decoupling. Carbon types, structure assignments, and attribution of peaks were determined from DEPT-NMR. Structural assignments were made with additional information from g-correlated spectroscopy, gHSQC, and g-heteronuclear multiple bond correlation experiments. Relative stereochemistry was assigned based on the 1D-NOE experiments. HRMS spectra were obtained by positive-ion electrospray ionization (ESI Agilent Jet Stream) through a liquid chromatography-quadrupole time of flight spectrometry method. Data are reported in the form *m*/*z* (intensity relative to base = 100). The structure of compounds **12d** and **12f** was determined on a crystal prepared from a CDCl_3_ solvent system by slow evaporation in a vial at room temperature. X-ray data were obtained using an Agilent Technologies Super-Nova (Cu) diffractometer. Infrared (IR) spectra were taken in a Nicolet iS10 Termo Scientific spectrometer as neat solids.

Theoretical calculations have been carried out within the DFT framework.^15^ Reaction profiles analysis have been carried out at the M06-2X-GD3(PCM)/6-31G* level by using the GAUSSIAN 16^16^ suite of programs. Single-point energy calculations have been computed at M06-2X(PCM)/6-31+G** from previously optimized structures. This highly parametrized method was well suited for the treatment of nonbonding interactions.^17^ Thermal Gibbs corrections were computed at the same level, at the selected temperature, and were not scaled. Solvent effects were estimated by the polarization continuum model^18^ (PCM) method within the self-consistent reaction field approach.^19^ All SCRF-PCM calculations were performed using chloroform (ε = 4.7113) as the model solvent. All the stationary points were characterized by harmonic vibrational analysis. Local minima showed positive definite Hessians. Fully optimized transition structures showed only one imaginary frequency associated with nuclear motion along the chemical transformation under study. Reaction paths were checked by intrinsic reaction coordinate (IRC) calculations. Activation Gibbs free energies were computed by using stationary points directly connected by IRC calculations. Representation of the noncovalent interactions (NCI plots) were computed using the NCIPLOT3^20^ program using wave functions computed at the M06-2X-GD3(PCM)/6-31G* level of optimized structures.

### General Procedure for the Synthesis of γ-Lactams **1** and **8**

Following a literature procedure,^12,13^ a solution of amine (2 equiv), aldehyde (1 equiv), ethyl pyruvate (3 equiv), and 1,1′-binaphthyl-2,2′-diyl hydrogen phosphate (10% mol) in CH_2_Cl_2_ (10 mL) was stirred overnight at room temperature in the presence of anhydrous MgSO_4_. Next, the reaction was filtered, and the resulting crude residue was purified by crystallization or flash column chromatography to afford pure 3-amino-3-pyrrolidin-2-ones **1** and **8**.

#### 1-(*p*-Tolyl)-3-(*p*-tolylamino)-1,5-dihydro-2*H*-pyrrol-2-one (**1**)

The general procedure was followed using *p*-toluidine (0.215 g, 2 mmol, 2 equiv), a 37% aqueous solution of formaldehyde (0.075 mL, 1 mmol, 1 equiv), and ethyl pyruvate (0.335 mL, 3 mmol, 3 equiv) in Et_2_O, affording 0.217 g (78%) of **1** as an orange solid after flash column chromatography (hexanes/AcOEt 9:1). mp (Et_2_O): 178–180 °C. ^1^H NMR (400 MHz, CDCl_3_) δ 7.64 (d, ^3^*J*_HH_ = 8.5 Hz, 2 × CH_Ar_), 7.20 (d, ^3^*J*_HH_ = 8.2 Hz, 2 × CH_Ar_), 7.13 (d, ^3^*J*_HH_ = 8.2 Hz, 2 × CH_Ar_), 7.1 (d, ^3^*J*_HH_ = 8.5 Hz, 2 × CH_Ar_), 6.53 (s, 1H, NH), 5.97 (t, ^3^*J*_HH_ = 2.6 Hz, 1H, = CH), 4.37 (d, ^3^*J*_HH_ = 2.6 Hz, 2H, CH_2_), 2.34 (s, 3H, CH_3_), 2.32 (s, 3H, CH_3_) ppm. ^13^C {^1^H} NMR (101 MHz, CDCl_3_) δ 166.5 (C=O), 139.2 (C_quat_), 136.8 (C_quat_), 134.5 (C_quat_), 134.3 (C_quat_), 130.7 (=C_quat_), 130.0 (2 × CH_Ar_), 129.8 (2 × CH_Ar_), 119.0 (2 × CH_Ar_), 116.8 (2 × CH_Ar_), 99.8 (=CH), 49.8 (CH_2_), 21.0 (CH_3_), 20.8 (CH_3_) ppm. FTIR (neat) *n*_max_: 3325 (NH_st_), 3073 (=CH_st_), 1671 (C=O_st_), 1644 (C=C_st_) cm^–1^. HRMS (ESI-TOF) *m*/*z* calcd for C_18_H_19_N_2_O [M + H]^+^, 279.1497; found, 279.1501.

#### 5-Phenyl-1-(*p*-tolyl)-3-(*p*-tolylamino)-1,5-dihydro-2*H*-pyrrol-2-one (**8a**)

The general procedure was followed using *p*-toluidine (0.215 g, 2 mmol, 2 equiv), benzaldehyde (0.102 mL, 1 mmol, 1 equiv), and ethyl pyruvate (0.335 mL, 3 mmol, 3 equiv), affording 0.340 g (96%) of **8a** as a white solid after crystallization (Et_2_O). mp (Et_2_O): 214–215 °C. ^1^H NMR (400 MHz, CDCl_3_) δ 7.40 (d, ^3^*J*_HH_ = 8.5 Hz, 2H, 2 × CH_Ar_), 7.32–7.17 (m, 5H, 5 × CH_Ar_), 7.10 (d, ^3^*J*_HH_ = 8.0 Hz, 2H, 2 × CH_Ar_), 7.09 (d, ^3^*J*_HH_ = 8.0 Hz, 2H, 2 × CH_Ar_), 6.98 (d, ^3^*J*_HH_ = 8.5 Hz, 2H, 2 × CH_Ar_), 6.58 (s, 1H, NH), 6.01 (d, ^3^*J*_HH_ = 2.6 Hz, 1H, = CH), 5.63 (d, ^3^*J*_HH_ = 2.6 Hz, 1H, CHN), 2.29 (s, 3H, CH_3_), 2.26 (s, 3H, CH_3_) ppm. ^13^C {^1^H} NMR (101 MHz, CDCl_3_) δ 167.0 (C=O), 138.8 (C_quat_), 137.6 (C_quat_), 134.8 (C_quat_), 134.8 (C_quat_), 132.0 (C_quat_), 130.5 (C_quat_), 129.9 (2 × CH_Ar_), 129.6 (2 × CH_Ar_), 129.0 (2 × CH_Ar_), 128.2 (CH_Ar_), 126.9 (2 × CH_Ar_), 121.6 (2 × CH_Ar_), 116.8 (2 × CH_Ar_), 107.2 (=CH), 64.3 (CHN), 21.0 (CH_3_), 20.8 (CH_3_) ppm. FTIR (neat) *n*_max_: 3306 (NH_st_), 1684 (C=O_st_), 1665 (C=C_st_) cm^–1^. HRMS (ESI-TOF) *m*/*z*: [M + H]^+^, calcd for C_24_H_23_N_2_O 355.1810; found, 355.1805.

#### 1,5-Diphenyl-3-(phenylamino)-1,5-dihydro-2*H*-pyrrol-2-one (**8b**)

The general procedure was followed using aniline (0.182 mL, 2 mmol, 2 equiv), benzaldehyde (0.102 mL, 1 mmol, 1 equiv), and ethyl pyruvate (0.335 mL, 3 mmol, 3 equiv), affording 0.212 g (65%) of **8b** as white crystals after crystallization (Et_2_O). mp (Et_2_O) = 224–225 °C. ^1^H NMR (400 MHz, DMSO-*d*_6_) δ 8.09 (s, 1H, NH), 7.62 (dd, ^3^*J*_HH_ = 8.7 Hz, ^4^*J*_HH_ = 1.2 Hz, 2H, 2 × CH_Ar_), 7.43–7.15 (m, 9H, 9 × CH_Ar_), 7.09–7.04 (m, 2H, 2 × CH_Ar_), 6.86 (tt, ^3^*J*_HH_ = 7.0 Hz, ^4^*J*_HH_ = 1.4 Hz, 2H, 2 × CH_Ar_), 6.34 (d, ^3^*J*_HH_ = 2.7 Hz, 1H, = CH), 6.06 (d, ^3^*J*_HH_ = 2.7 Hz, 1H, CHN) ppm. ^13^C {^1^H} NMR (101 MHz, DMSO-*d*_6_) δ 166.52 (C=O), 142.0 (C_quat_), 138.0 (C_quat_), 137.2 (C_quat_), 131.8 (C_quat_), 129.0 (2 × CH_Ar_), 128.8 (2 × CH_Ar_), 128.7 (2 × CH_Ar_), 127.7 (CH_Ar_), 126.8 (2 × CH_Ar_), 124.4 (CH_Ar_), 121.5 (2 × CH_Ar_), 120.2 (CH_Ar_), 116.7 (2 × CH_Ar_), 109.8 (=CH), 62.4 (CHN) ppm. FTIR (neat) *n*_max_: 3303 (NH_st_), 1681 (C=O_st_), 1666 (C=C_st_) cm^–1^. HRMS (ESI-TOF) *m*/*z*: [M + H]^+^, calcd for C_22_H_19_N_2_O 327.1497; found, 327.1501.

#### 1-(4-Methoxyphenyl)-3-((4-methoxyphenyl)amino)-5-phenyl-1,5-dihydro-2*H*-pyrrol-2-one (**8c**)

The general procedure was followed using *p*-anisidine (0.246 g, 2 mmol, 2 equiv), benzaldehyde (0.102 mL, 1 mmol, 1 equiv), and ethyl pyruvate (0.335 mL, 3 mmol, 3 equiv), affording 0.300 g (78%) of **8c** as a white solid after flash column chromatography (hexanes/AcOEt 8:2). mp (Et_2_O): 198–200 °C. ^1^H NMR (300 MHz, CDCl_3_) δ 7.36 (d, ^3^*J*_HH_ = 9.1 Hz, 2H, 2 × CH_Ar_), 7.30–7.16 (m, 5H, 5 × CH_Ar_), 7.03 (d, ^3^*J*_HH_ = 8.9 Hz, 2H, 2 × CH_Ar_), 6.86 (d, ^3^*J*_HH_ = 8.9 Hz, 2H, 2 × CH_Ar_), 6.81 (d, ^3^*J*_HH_ = 9.1 Hz, 2H, 2 × CH_Ar_), 6.46 (br s, 1H, NH), 5.94 (d, ^3^*J*_HH_ = 2.5 Hz, 1H, = CH), 5.57 (d, ^3^*J*_HH_ = 2.5 Hz, 1H, CHN), 3.78 (s, 3H, CH_3_), 3.74 (s, 3H, CH_3_) ppm. ^13^C {^1^H} NMR (75 MHz, CDCl_3_) δ 167.3 (C=O), 157.1 (C_quat_), 154.5 (C_quat_), 137.8 (C_quat_), 135.0 (C_quat_), 133.1 (C_quat_), 130.4 (C_quat_), 129.0 (2 × CH_Ar_), 128.2 (2 × CH_Ar_), 127.1 (2 × CH_Ar_), 123.9 (2 × CH_Ar_), 118.6 (2 × CH_Ar_), 114.8 (2 × CH_Ar_), 114.3 (2 × CH_Ar_), 106.3 (=CH), 64.9 (CHN), 55.7 (CH_3_), 55.5 (CH_3_) ppm. FTIR (neat) *n*_max_: 3304 (NH_st_), 3017 (=CH_st_), 1669 (C=O_st_), 1659 (C=C_st_), 1250 (C–O_st_), 1032 (C–O_st_) cm^–1^. HRMS (ESI-TOF) *m*/*z*: [M + H]^+^, calcd for C_24_H_23_N_2_O_3_ 387.1709; found, 387.1702.

#### 1-(4-Bromophenyl)-3-((4-bromophenyl)amino)-5-phenyl-1,5-dihydro-2*H*-pyrrol-2-one (**8d**)

The general procedure was followed using *p*-bromoaniline (0.344 g, 2 mmol, 2 equiv), benzaldehyde (0.102 mL, 1 mmol, 1 equiv), and ethyl pyruvate (0.335 mL, 3 mmol, 3 equiv), affording 0.396 g (82%) of **8d** as a white solid after flash column chromatography (hexanes/AcOEt 8:2). mp (Et_2_O): 225–226 °C. ^1^H NMR (400 MHz, CDCl_3_) δ 7.45 (d, ^3^*J*_HH_ = 9.0 Hz, 2H, 2 × CH_Ar_), 7.39 (d, ^3^*J*_HH_ = 9.2 Hz, 2H, 2 × CH_Ar_), 7.38 (d, ^3^*J*_HH_ = 8.9 Hz, 2H, 2 × CH_Ar_), 7.32–7.24 (m, 3H, 3 × CH_Ar_), 7.18 (d, ^3^*J*_HH_ = 8.3 Hz, 2H, 2 × CH_Ar_), 6.94 (d, ^3^*J*_HH_ = 8.9 Hz, 2H, 2 × CH_Ar_), 6.66 (s, 1H, NH), 6.05 (d, ^3^*J*_HH_ = 2.6 Hz, 1H, = CH), 5.63 (d, ^3^*J*_HH_ = 2.6 Hz, 1H, CHN) ppm. ^13^C {^1^H} NMR (101 MHz, CDCl_3_) δ 167.1 (C=O), 140.3 (C_quat_), 136.9 (C_quat_), 136.4 (C_quat_), 132.4 (2 × CH_Ar_), 132.1 (2 × CH_Ar_), 131.8 (C_quat_), 129.3 (2 × CH_Ar_), 128.6 (CH_Ar_), 126.7 (2 × CH_Ar_), 122.9 (2 × CH_Ar_), 118.4 (2 × CH_Ar_), 118.1 (C_quat_), 113.6 (C_quat_), 108.9 (=CH), 64.3 (CHN) ppm. FTIR (neat) *n*_max_: 3327 (NH_st_), 1672 (C=O_st_), 1642 (C=C_st_), 1073 (C–Br_st_), 820 (C–Br_st_) cm^–1^. HRMS (ESI-TOF) *m*/*z*: [M + H]^+^, calcd for C_22_H_17_Br_2_N_2_O 482.9708; found, 482.9715.

#### 1-(4-Chlorophenyl)-3-((4-chlorophenyl)amino)-5-phenyl-1,5-dihydro-2*H*-pyrrol-2-one (**8e**)

The general procedure was followed using *p*-chloroaniline (0.212 g, 2 mmol, 2 equiv), benzaldehyde (0.102 mL, 1 mmol, 1 equiv), and ethyl pyruvate (0.335 mL, 3 mmol, 3 equiv), affording 0.304 g (77%) of **8e** as a white solid after crystallization (Et_2_O). mp (Et_2_O) = 207–209 °C. ^1^H NMR (400 MHz, CDCl_3_) δ 7.53–7.46 (m, 2H, 2 × CH_Ar_), 7.33–7.26 (m, 3H, 3 × CH_Ar_), 7.26–7.22 (m, 4H, 4 × CH_Ar_), 7.19 (dd, ^3^*J*_HH_ = 8.1, ^4^*J*_HH_ = 1.6 Hz, 2H, 2 × CH_Ar_). 7.03–6.95 (m, 2H, 2 × CH_Ar_), 6.63 (s, 1H, NH), 6.05 (d, ^3^*J*_HH_ = 2.6 Hz, 1H, = CH), 5.64 (d, ^3^*J*_HH_ = 2.6 Hz, 1H, CHN) ppm. ^13^C {^1^H} NMR (101 MHz, CDCl_3_) δ 167.1 (C=O), 139.9 (C_quat_), 137.0 (C_quat_), 135.9 (C_quat_), 131.9 (C_quat_), 130.4 (C_quat_), 129.5 (2 × CH_Ar_), 129.3 (2 × CH_Ar_), 129.2 (2 × CH_Ar_), 128.6 (CH_Ar_), 126.8 (2 × CH_Ar_), 126.4 (C_quat_), 122.6 (2 × CH_Ar_), 118.0 (2 × CH_Ar_), 108.7 (=CH), 64.3 (CHN) ppm. FTIR (neat) *n*_max_: 3328 (NH_st_), 1664 (C=O_st_), 1617 (C=C_st_) cm^–1^. HRMS (ESI-TOF) *m*/*z*: [M + H]^+^, calcd for C_22_H_17_Cl_2_N_2_O 395.0718; found, 395.0715.

#### 1-(4-Fluorophenyl)-3-((4-fluorophenyl)amino)-5-phenyl-1,5-dihydro-2*H*-pyrrol-2-one (**8f**)

The general procedure was followed using *p*-fluoroaniline (0.222 g, 2 mmol, 2 equiv), benzaldehyde (0.102 mL, 1 mmol, 1 equiv), and ethyl pyruvate (0.335 mL, 3 mmol, 3 equiv) to afford 0.344 g (95%) of **8f** as a yellow solid after crystallization (Et_2_O). mp (Et_2_O) = 213-215 °C. ^1^H NMR (400 MHz, CDCl_3_) δ 7.49–7.43 (m, 2H, 2 × CH_Ar_), 7.32–7.26 (m, 2H, 2 × CH_Ar_), 7.25–7.22 (m, 1H, CH_Ar_), 7.20–7.16 (m, 2H, 2 × CH_Ar_), 7.04–6.93 (m, 6H, 6 × CH_Ar_), 6.55 (s, 1H, NH), 6.00 (d, ^3^*J*_HH_ = 2.6 Hz, 1H, = CH), 5.61 (d, ^3^*J*_HH_ = 2.6 Hz, 1H, CHN) ppm. ^13^C {^1^H} NMR (101 MHz, CDCl_3_) δ 167.2 (C=O), 160.0 (d, ^1^*J*_FC_ = 245.1 Hz, C_quat_), 157.8 (d, ^1^*J*_FC_ = 240.7 Hz, C_quat_), 137.5 (d, ^4^*J*_FC_ = 2.4 Hz, C_quat_), 137.2 (C_quat_), 133.5 (d, ^4^*J*_FC_ = 3.0 Hz, C_quat_), 132.6 (C_quat_), 129.2 (2 × CH_Ar_), 128.5 (CH_Ar_), 126.9 (2 × CH_Ar_), 123.7 (d, ^2^*J*_FC_ = 8.0 Hz 2 × CH_Ar_), 118.4 (d, ^3^*J*_FC_ = 7.7 Hz 2 × CH_Ar_), 116.2 (d, ^3^*J*_FC_ = 28.0 Hz 2 × CH_Ar_), 115.9 (d, ^2^*J*_FC_ = 28.0 Hz, 2 × CH_Ar_), 107.4 (=CH), 77.4 (CH), 64.7 (CHN) ppm. ^19^F {^1^H} NMR (282 MHz, CDCl_3_) δ −116.9, −121.9 ppm. FTIR (neat) *n*_max_: 3328 (NH_st_), 1664 (C=O_st_), 1615 (C=C_st_) cm^–1^. HRMS (ESI-TOF) *m*/*z*: [M + H]^+^, calcd for C_22_H_17_F_2_N_2_O 363.1309; found, 363.1307.

#### 1-(3-Chlorophenyl)-3-((3-chlorophenyl)amino)-5-phenyl-1,5-dihydro-2*H*-pyrrol-2-one (**8g**)

The general procedure was followed using *m*-chloroaniline (0.212 mL, 2 mmol, 2 equiv), benzaldehyde (0.102 mL, 1 mmol, 1 equiv), and ethyl pyruvate (0.335 mL, 3 mmol, 3 equiv), affording 0.350 g (89%) of **8g** as white crystals after crystallization (Et_2_O). mp (Et_2_O) = 203–205 °C. ^1^H NMR (400 MHz, CDCl_3_) δ 7.70 (t, ^4^*J*_HH_ = 2.0 Hz, 1H, CH_Ar_), 7.38 (ddd, ^3^*J*_HH_ = 8.3 Hz, 4*J*_HH_ = 2.2 Hz, 4*J*_HH_ = 1.0 Hz, 1H, CH_Ar_), 7.35–7.25 (m, 3H, 3 × CH_Ar_), 7.24–7.15 (m, 4H, 4 × CH_Ar_), 7.08–7.03 (m, 2H, 2 × CH_Ar_), 6.93 (ddd, ^3^*J*_HH_ = 7.8 Hz, ^4^*J*_HH_ = 2.1 Hz, ^4^*J*_HH_ = 1.2 Hz, 2H, 2 × CH_Ar_), 6.70 (s, 1H, NH), 6.11 (d, ^3^*J*_HH_ = 2.6 Hz, 1H, = CH), 5.66 (d, ^3^*J*_HH_ = 2.6 Hz, 1H, CHN) ppm. ^13^C {^1^H} NMR (101 MHz, CDCl_3_) δ 167.0 (C=O), 142.3 (C_quat_), 138.3 (C_quat_), 136.6 (C_quat_), 135.1 (C_quat_), 134.6 (C_quat_), 131.4 (C_quat_), 130.4 (CH_Ar_), 129.9 (CH_Ar_), 129.2 (2 × CH_Ar_), 128.5 (CH_Ar_), 126.6 (2 × CH_Ar_), 125.0 (CH_Ar_), 121.4 (CH_Ar_), 121.3 (CH_Ar_), 119.1 (CH_Ar_), 116.4 (CH_Ar_), 115.0 (CH_Ar_), 109.4 (=CH), 64.2 (CHN) ppm. FTIR (neat) *n*_max_: 3325 (NH_st_), 1695 (C=O_st_), 1612 (C=C_st_) cm^–1^. HRMS (ESI-TOF) *m*/*z*: [M + H]^+^, calcd for C_22_H_17_Cl_2_N_2_O 395.0718; found, 395.0714.

#### 1-(2-Fluorophenyl)-3-((2-fluorophenyl)amino)-5-phenyl-1,5-dihydro-2*H*-pyrrol-2-one (**8h**)

The general procedure was followed using *o*-fluoroaniline (0.193 mL, 2 mmol, 2 equiv), benzaldehyde (0.102 mL, 1 mmol, 1 equiv), and ethyl pyruvate (0.335 mL, 3 mmol, 3 equiv), affording 0.279 g (77%) of **8h** as a white solid after crystallization (Et_2_O). mp (Et_2_O): 176–178 °C. ^1^H NMR (400 MHz, CDCl_3_) δ 7.33–7.19 (m, 6H, 6× CH_Ar_), 7.17–7.03 (m, 5H, 5× CH_Ar_), 6.94–6.84 (m, 2H, 2 × CH_Ar_), 6.21 (d, ^3^*J*_HH_ = 2.6 Hz, 1H, = CH), 5.73 (d, ^3^*J*_HH_ = 2.6 Hz, 1H, CHN) ppm. ^13^C {^1^H} NMR (101 MHz, CDCl_3_) δ 167.0 (C=O), 157.4 (d, ^1^*J*_FC_ = 250.3 Hz, C_quat_), 152.5 (d, ^1^*J*_FC_ = 243.6 Hz, C_quat_), 136.6 (C_quat_), 132.1 (C_quat_), 130.1 (d, ^2^*J*_FC_ = 10.9 Hz, C_quat_), 128.9 (2 × CH_Ar_), 128.7 (CH_Ar_), 128.6 (d, ^3^*J*_FC_ = 8.0 Hz, CH_Ar_), 128.5 (d, ^4^*J*_FC_ = 1.6 Hz, CH_Ar_), 127.5 (2 × CH_Ar_), 124.6 (d, ^3^*J*_FC_ = 6.6 Hz, CH_Ar_), 124.5 (d, ^3^*J*_FC_ = 6.6 Hz, CH_Ar_), 124.1 (d, ^2^*J*_FC_ = 11.6 Hz, C_quat_), 121.4 (d, ^3^*J*_FC_ = 7.2 Hz, CH_Ar_), 116.7 (d, ^4^*J*_FC_ = 1.7 Hz, CH_Ar_), 116.6 (d, ^2^*J*_FC_ = 20.3 Hz, CH_Ar_), 115.5 (d, ^2^*J*_FC_ = 18.7 Hz, CH_Ar_), 109.8 (=CH), 65.41 (d, ^4^*J*_FC_ = 4.3 Hz, CHN) ppm. ^19^F {^1^H} NMR (282 MHz, CDCl_3_) δ −120.6, −132.1 ppm. FTIR *n*_max_ 3323 (NH_st_), 1693 (C=O_st_), 1657 (C=C_st_), 1112 (C–F_st_) cm^–1^.

#### 5-Phenyl-1-(3-(trifluoromethyl)phenyl)-3-((3-(trifluoromethyl)phenyl)amino)-1,5-dihydro-2*H*-pyrrol-2-one (**8i**)

The general procedure was followed using *m*-(trifluoromethyl)aniline (0.250 g, 2 mmol, 2 equiv), benzaldehyde (0.102 mL, 1 mmol, 1 equiv), and ethyl pyruvate (0.335 mL, 3 mmol, 3 equiv), affording 0.301 g (65%) of **8i** as a white solid after flash column chromatography (hexanes/AcOEt 8:2). mp (Et_2_O): 198–200 °C. ^1^H NMR (400 MHz, CDCl_3_) δ 7.93 (s, 1H, CH_Ar_), 7.72 (d, ^3^*J*_HH_ = 8.3 Hz, 1H, CH_Ar_), 7.44–7.37 (m, 2H, 2 × CH_Ar_), 7.35–7.27 (m, 5H, 5× CH_Ar_), 7.24 (d, ^3^*J*_HH_ = 1.8 Hz, 2H, 2 × CH_Ar_), 7.23–7.19 (m, 2H, 2 × CH_Ar_), 6.85 (s, 1H, NH), 6.15 (d, ^3^*J*_HH_ = 2.6 Hz, 1H, = CH), 5.73 (d, ^3^*J*_HH_ = 2.6 Hz, 1H, CHN) ppm. ^13^C {^1^H} NMR (101 MHz, CDCl_3_) δ 167.2 (C=O), 141.7 (C_quat_), 137.8 (C_quat_), 136.4 (C_quat_), 132.0 (q, ^2^*J*_FC_ = 32.4 Hz, C_quat_), 131.6 (C_quat_), 131.5 (q, ^2^*J*_FC_ = 32.4 Hz, C_quat_), 130.1 (CH_Ar_), 129.6 (CH_Ar_), 129.4 (2 × CH_Ar_), 128.8 (CH_Ar_), 126.8 (2 × CH_Ar_), 124.2 (CH_Ar_), 124.0 (q, ^1^*J*_FC_ = 272.4 Hz, CF_3_), 123.9 (q, ^1^*J*_FC_ = 272.5 Hz, CF_3_), 121.61 (q, ^3^*J*_FC_ = 3.8 Hz, CH_Ar_), 119.9 (CH_Ar_), 118.1 (q, ^3^*J*_FC_ = 3.8 Hz, CH_Ar_), 118.0 (q, ^3^*J*_FC_ = 4.0 Hz, CH_Ar)_, 113.1 (q, ^3^*J*_FC_ = 3.9 Hz, CH_Ar_), 109.7 (=CH), 64.3 (CHN) ppm. ^19^F {^1^H} NMR (282 MHz, CDCl3) δ −63.3, −63.2 ppm. FTIR (neat) *n*_max_: 3323 (NH_st_), 1675 (C=O_st_), 1663 (C=C_st_), 1201 (C–F_st_) cm^–1^. HRMS (ESI-TOF) *m*/*z* calcd for C_24_H_17_F_6_N_2_O [M + H]^+^, 463.1245; found, 463.1247.

### General Procedure for the Functionalization of γ-Lactams **1** and **8** with Eschenmoser’s Salt

The corresponding 3-amino-3-pyrrolidin-2-one **1** or **8** (1 mmol, 1 equiv) was stirred overnight with 1.5 equiv of *N*,*N*-dimethylmethyleneiminium iodide (0.278 g, 1.5 mmol, 1.5 equiv) in the presence of freshly distilled triethylamine (0.279 mL, 2.0 mmol, 2 equiv) in refluxing chloroform (3 mL) under a N_2_ atmosphere. The reaction crude was acidified with 0.5 M HCl aqueous solution and extracted with dichloromethane (2 × 20 mL). The combined organic layers were dried with MgSO_4_ and purified by flash column chromatography, affording the corresponding pure functionalized γ-lactams **2** or **9**. In some cases, other purification processes were necessary as detailed for each compound.

#### 4-((Dimethylamino)methyl)-1-(*p*-tolyl)-3-(*p*-tolylamino)-1,5-dihydro-2*H*-pyrrol-2-one (**2**)

The general procedure was followed using 1-(*p*-tolyl)-3-(*p*-tolylamino)-1,5-dihydro-2*H*-pyrrol-2-one (0.278 g, 1 mmol, 1 equiv) **1** to afford 0.242 g (72%) of **2** as red crystals after crystallization (hexanes/CH_2_Cl_2_ 3:1). mp (hexanes/CH_2_Cl_2_) = 106–107 °C. ^1^H NMR (400 MHz, CDCl_3_) δ 7.68 (d, ^3^*J*_HH_ = 8.5 Hz, 2H, 2 × CH_Ar_), 7.19 (d, ^3^*J*_HH_ = 8.5 Hz, 2H, 2 × CH_Ar_), 7.08 (d, ^3^*J*_HH_ = 8.2 Hz, 2H, 2 × CH_Ar_), 6.85 (d, ^3^*J*_HH_ = 8.2 Hz, 2H, 2 × CH_Ar_), 6.05 (s, 1H, NH), 4.35 (s, 2H, CH_2_), 3.11 (s, 2H, CH_2_NMe_2_), 2.34 (s, 3H, CH_3Tol_), 2.31 (s, 3H, CH_3Tol_), 2.19 (s, 6H, 2 × NCH_3_) ppm. ^13^C {^1^H} NMR (101 MHz, CDCl_3_) δ 167.1 (C=O), 139.8 (C_quat_), 136.9 (C_quat_), 133.6 (C_quat_), 132.6 (C_quat_), 131.4 (C_quat_), 129.7 (2 × CH_Ar_), 129.6 (2 × CH_Ar_), 119.5 (2 × CH_Ar_), 118.9 (C_quat_), 118.4 (2 × CH_Ar_), 56.6 (CH_2_), 51.0 (CH_2_), 45.6 (2 × NCH_3_), 20.9 (CH_3Tol_), 20.8 (CH_3Tol_) ppm. **FTIR** (neat) *n*_max_: 3031 (=CH_st_), 1686 (C=O_st_), 1615 (C=C_st_) cm^–1^. **HRMS** (ESI-TOF) *m*/*z*: [M–Me_2_N]^+^, calcd for C_19_H_19_N_2_O 291.1497; found, 291.1495.

#### 4-((Dimethylamino)methyl)-5-phenyl-1-(*p*-tolyl)-3-(*p*-tolylamino)-1,5-dihydro-2*H*-pyrrol-2-one (**9a**)

The general procedure was followed using 5-phenyl-1-(*p*-tolyl)-3-(*p*-tolylamino)-1,5-dihydro-2*H*-pyrrol-2-one **8a** (0.354 g, 1 mmol, 1 equiv), affording 0.342 g (83%) of **9a** as red crystals after flash column chromatography (hexanes/AcOEt 8:2) followed by crystallization (pentane/Et_2_O 3:1). mp (pentane/Et_2_O) = 98–100 °C. ^1^H NMR (400 MHz, CDCl_3_) δ 7.49 (d, ^3^*J*_HH_ = 8.5 Hz, 2H, 2 × CH_Ar_), 7.33–7.29 (m, 4H, 4 × CH_Ar_), 7.25 (m, 1H, CH_Ar_), 7.06 (d, ^3^*J*_HH_ = 8.2 Hz, 2H, 2 × CH_Ar_), 7.02 (d, ^3^*J*_HH_ = 8.2 Hz, 2H, 2 × CH_Ar_), 6.76 (d, ^3^*J*_HH_ = 8.5 Hz, 2H, 2 × CH_Ar_), 6.12 (s, 1H, NH), 5.69 (s, 1H, CH), 2.78 (d, ^2^*J*_HH_ = 13.9 Hz, 1H, CH_A_CH_B_), 2.67 (d, ^2^*J*_HH_ = 13.9 Hz, 1H, CH_A_CH_B_), 2.28 (s, 3H, CH_3Tol_), 2.24 (s, 3H, CH_3Tol_), 2.11 (s, 6H, 2 × NCH_3_) ppm. ^13^C {^1^H} NMR (101 MHz, CDCl_3_) δ 167.7 (C=O), 140.1 (C_quat_), 137.8 (C_quat_), 135.4 (C_quat_), 134.0 (C_quat_), 131.3 (C_quat_), 131.3 (C_quat_), 129.6 (2 × CH_Ar_), 129.5 (2 × CH_Ar_), 128.9 (2 × CH_Ar_), 128.0 (2 × CH_Ar_), 127.2 (C_quat_), 127.0 (2 × CH_Ar_), 120.1 (2 × CH_Ar_), 119.1 (CH_Ar_), 64.6 (CH), 55.3 (CH_2_), 45.5 (2 × NCH_3_), 20.9 (CH_3Tol_), 20.8 (CH_3Tol_) ppm. FTIR (neat) *n*_max_: 3331 (NH_st_), 1689 (C=O_st_), 1614 (C=C_st_) cm^–1^. HRMS (ESI-TOF) *m*/*z*: [M–Me_2_N]^+^, calcd for C_25_H_23_N_2_O 367.1810; found, 367.1806.

#### 4-((Dimethylamino)methyl)-1,5-diphenyl-3-(phenylamino)-1,5-dihydro-2*H*-pyrrol-2-one (**9b**)

The general procedure was followed using 1,5-diphenyl-3-(phenylamino)-1,5-dihydro-2*H*-pyrrol-2-one (0.326 g, 1 mmol, 1 equiv) **8b** to afford 0.314 g (82%) of **9b** as white crystals after flash column chromatography (hexanes/AcOEt 8:2) followed by crystallization (pentane/Et_2_O 3:1). mp (pentane/Et_2_O) = 140–142 °C. ^1^H NMR (400 MHz, CDCl_3_) δ 7.56 (dd, ^3^*J*_HH_ = 8.8 Hz, ^4^*J*_HH_ = 1.2 Hz, 2H, 2 × CH_Ar_), 7.28–7.32 (m, 4H, 4 × CH_Ar_), 7.21–7.16 (m, 3H, 3 × CH_Ar_), 7.15–7.11 (m, 2H, 2 × CH_Ar_), 6.99–6.94 (m, 1H, 1 × CH_Ar_), 6.84 (tt, ^3^*J*_HH_ = 7.3 Hz, ^4^*J*_HH_ = 1.2 Hz, 1H, CH_Ar_), 6.75 (dd, ^3^*J*_HH_ = 8.8 Hz, ^4^*J*_HH_ = 1.2 Hz, 2H, 2 × CH_Ar_), 6.17 (s, 1H, NH), 5.67 (s, 1H, CH), 3.05–2.36 (m, 2H, CH_2_), 2.04 (s, 6H, 2 × NCH_3_) ppm. ^13^C {^1^H} NMR (101 MHz, CDCl_3_) δ 167.8 (C=O), 142.8 (C_quat_), 137.9 (C_quat_), 137.5 (C_quat_), 130.8 (C_quat_), 129.4 (CH_Ar_), 129.1 (2 × CH_Ar_), 129.0 (2 × CH_Ar_), 128.9 (2 × CH_Ar_), 128.1 (CH_Ar_), 126.9 (2 × CH_Ar_), 124.4 (CH_Ar_), 121.5 (CH_Ar_), 120.6 (2 × CH_Ar_), 118.3 (2 × CH_Ar_), 64.5 (CH), 55.5 (CH_2_), 45.5 (2 × NCH_3_) ppm. FTIR (neat) ν_max_: 3039 (=CH_st_), 1691 (C=O_st_), 1610 (C=C_st_) cm^–1^. HRMS (ESI-TOF) *m*/*z*: [M + H]^+^, calcd for C_25_H_26_N_3_O 384.2076; found, 384.2074.

#### 4-((Dimethylamino)methyl)-1-(4-methoxyphenyl)-3-((4-methoxyphenyl)amino)-5-phenyl-1,5-dihydro-2*H*-pyrrol-2-one (**9c**)

The general procedure was followed using 1-(4-methoxyphenyl)-3-((4-methoxyphenyl)amino)-5-phenyl-1,5-dihydro-2*H*-pyrrol-2-one (0.386 g, 1 mmol, 1 equiv) **8c** to afford 0.310 g (70%) of **9c** as an orange oil after flash column chromatography (hexanes/AcOEt 8:2). ^1^H NMR (400 MHz, CDCl_3_) δ 7.46 (d, ^3^*J*_HH_ = 9.2 Hz, 2H, 2 × CH_Ar_), 7.31–7.27 (m, 5H, 5 × CH_Ar_), 6.89 (d, ^3^*J*_HH_ = 8.9 Hz, 2H, 2 × CH_Ar_), 6.79 (d, ^3^*J*_HH_ = 8.9 Hz, 2H, 2 × CH_Ar_), 6.78 (d, ^3^*J*_HH_ = 9.2 Hz, 2H, 2 × CH_Ar_), 6.07 (s, 1H, NH), 5.63 (s, 1H, CH), 3.76 (s, 3H, OCH_3_), 3.71 (s, 3H, OCH_3_), 2.69 (d, ^2^*J*_HH_ = 13.8 Hz, 1H, CH_A_CH_B_), 2.61 (d, ^2^*J*_HH_ = 13.8 Hz, 1H, CH_A_CH_B_), 2.08 (s, 6H, 2 × NCH_3_) ppm. ^13^C {^1^H} NMR (101 MHz, CDCl_3_) δ 167.6 (C=O), 156.6 (C_quat_), 155.6 (C_quat_), 137.7 (C_quat_), 135.4 (C_quat_), 132.3 (C_quat_), 131.5 (C_quat_), 131.0 (C_quat_), 128.9 (2 × CH_Ar_), 128.0 (2 × CH_Ar_), 127.2 (2 × CH_Ar_), 122.9 (2 × CH_Ar_), 122.4 (CH_Ar_), 114.4 (2 × CH_Ar_), 114.2 (2 × CH_Ar_), 65.1 (CH), 55.6 (OCH_3_), 55.4 (OCH_3_), 54.7 (CH_2_), 45.3 (2 × NCH_3_) ppm. FTIR (neat) *n*_max_: 3035 (=CH_st_), 1688 (C=O_st_), 1616 (C=C_st_) cm^–1^. HRMS (ESI-TOF) *m*/*z*: [M + H]^+^, calcd for C_27_H_30_N_3_O_3_ 444.2287; found, 444.2277.

#### 1-(4-Bromophenyl)-3-((4-bromophenyl)amino)-4-((dimethylamino)methyl)-5-phenyl-1,5-dihydro-2*H*-pyrrol-2-one (**9d**)

The general procedure was followed using 1-(4-bromophenyl)-3-((4-bromophenyl)amino)-5-phenyl-1,5-dihydro-2*H*-pyrrol-2-one **8d** (0.484 g, 1 mmol, 1 equiv), affording 0.504 g (93%) of **9d** as orange crystals after flash column chromatography (hexanes/AcOEt 7:3) followed by crystallization (pentane/Et_2_O 3:1). mp (pentane/Et_2_O) = 139–141 °C. ^1^H NMR (400 MHz, CDCl_3_) δ 7.53 (d, ^3^*J*_HH_ = 7.6 Hz, 2H, 2 × CH_Ar_), 7.37–7.25 (m, 9H, 9 × CH_Ar_), 6.68 (d, ^3^*J*_HH_ = 7.6 Hz, 2H, 2 × CH_Ar_), 6.38 (s, 1H, NH), 5.66 (s, 1H, CH), 2.76 (d, ^2^*J*_HH_ = 14.5 Hz, 1H, CH_A_CH_B_), 2.72 (d, ^2^*J*_HH_ = 14.5 Hz, 1H, CH_A_CH_B_), 2.11 (s, 6H, 2 × NCH_3_) ppm. ^13^C {^1^H} NMR (101 MHz, CDCl_3_) δ 167.5 (C=O), 141.8 (C_quat_), 136.8 (C_quat_), 136.8 (C_quat_), 132.0 (2 × CH_Ar_), 131.9 (2 × CH_Ar_), 130.7 (C_quat_), 130.6 (C_quat_), 129.2(2 × CH_Ar_), 128.5 (CH_Ar_), 126.8 (2 × CH_Ar_), 122.0 (2 × CH_Ar_), 119.7 (2 × CH_Ar_), 117.3 (C_quat_), 113.6 (C_quat_), 64.4 (CH), 55.5 (CH_2_), 45.5 (2 × NCH_3_) ppm. FTIR (neat) *n*_max_: 3321 (NH_st_), 1689 (C=O_st_), 1604 (C=C_st_) cm^–1^. HRMS (ESI-TOF) *m*/*z*: [M + H]^+^, calcd for C_25_H_24_Br_2_N_3_O 542.0286; found, 542.0269.

#### 1-(*p*-Chlorophenyl)-3-((*p*-chlorophenyl)amino)-4-((dimethylamino)methyl)-5-phenyl-1,5-dihydro-2*H*-pyrrol-2-one (**9e**)

The general procedure was followed using 1-(*p*-chlorophenyl)-3-((*p*-chlorophenyl)amino)-5-phenyl-1,5-dihydro-2*H*-pyrrol-2-one (0.395 g, 1 mmol, 1 equiv) **8e** to afford 0.281 g (62%) of **9e** as a red solid after flash column chromatography (hexanes/AcOEt 8:2). ^1^H NMR (400 MHz, CDCl_3_) δ 7.54 (d, ^3^*J*_HH_ = 9.1 Hz, 2H, 2 × CH_Ar_), 7.34–7.23 (m, 5H, 5 × CH_Ar_), 7.18 (d, ^3^*J*_HH_ = 9.0 Hz, 2H, 2 × CH_Ar_), 7.14 (d, ^3^*J*_HH_ = 8.9 Hz, 2H, 2 × CH_Ar_), 6.72 (d, ^3^*J*_HH_ = 8.9 Hz, 2H, 2 × CH_Ar_), 6.25 (s, 1H, NH), 5.62 (s, 1H, CH), 2.73 (d, ^2^*J*_HH_ = 12.5 Hz, 1H, CH_A_CH_B_), 2.59 (d, ^2^*J*_HH_ = 12.5 Hz, 1H, CH_A_CH_B_), 2.08 (s, 6H, 2 × NCH_3_) ppm. ^13^C {^1^H} NMR (101 MHz, CDCl_3_) δ 167.5 (C=O), 141.3 (C_quat_), 136.9 (C_quat_), 136.4 (C_quat_), 130.8 (C_quat_), 130.2 (C_quat_), 129.6 (C_quat_), 129.2 (2 × CH_Ar_), 129.1 (2 × CH_Ar_), 129.0 (2 × CH_Ar_), 128.5 (CH_Ar_), 126.9 (2 × CH_Ar_), 126.5 (C_quat_), 121.7 (2 × CH_Ar_), 119.6 (2 × CH_Ar_), 64.6 (CH), 55.6 (CH_2_), 45.5 (2 × NCH_3_) ppm. FTIR (neat) ν_max_: 3399 (N–H_st_), 1695 (C=O_st_), 1613 (C=C_st_) cm^–1^. HRMS (ESI-TOF) *m*/*z*: [M + H]^+^, calcd for C_25_H_24_Cl_2_N_3_O 452.1218; found, 452.1228.

#### 4-((Dimethylamino)methyl)-1-(*p*-fluorophenyl)-3-((*p*-fluorophenyl)amino)-5-phenyl-1,5-dihydro-2*H*-pyrrol-2-one (**9f**)

The general procedure was followed using 1-(*p*-fluorophenyl)-3-((*p*-fluorophenyl)amino)-5-phenyl-1,5-dihydro-2*H*-pyrrol-2-one (0.362 g, 1 mmol, 1 equiv) **8f** to afford 0.248 g (59%) of **9f** as a white solid after flash column chromatography (hexanes/AcOEt 8:2). ^1^H NMR (400 MHz, CDCl_3_) δ 7.56–7.48 (m, 2H, 2 × CH_Ar_), 7.33–7.20 (m, 5H, 5 × CH_Ar_), 6.98–6.86 (m, 4H, 4 × CH_Ar_), 6.82 (dd, ^3^*J*_HH_ = 9.1 Hz, ^4^*J*_HH_ = 4.7 Hz, 2H, 2 × CH_Ar_), 6.24 (s, 1H, NH), 5.61 (s, 1H, CH), 2.66 (m, 2H, CH_2_), 2.06 (s, 6H, 2 × NCH_3_) ppm. ^13^C {^1^H} NMR (101 MHz, CDCl_3_) δ 167.6 (C=O), 159.5 (d, ^1^*J*_FC_ = 244.2 Hz, C_quat_), 158.4 (d, ^1^*J*_FC_ = 240.5 Hz, C_quat_), 138.5 (d, ^4^*J*_FC_ = 2.6 Hz, C_quat_), 137.1 (C_quat_), 133.9 (d, ^4^*J*_FC_ = 2.6 Hz, C_quat_), 134.5 (C_quat_), 129.1 (2 × CH_Ar_), 128.3 (CH_Ar_) 127.3 (C_quat_), 127.0 (2 × CH_Ar_), 122.6 (d, ^4^*J*_FC_ = 8.1 Hz, 2 × CH_Ar_), 120.9 (d, ^3^*J*_FC_ = 7.9 Hz, 2 × CH_Ar_), 115.8 (d, ^3^*J*_FC_ = 1.3 Hz, 2 × CH_Ar_), 115.6 (d, ^3^*J*_FC_ = 1.4 Hz, 2 × CH_Ar_), 64.9 (CH), 55.2 (CH_2_), 45.5 (2 × NCH_3_) ppm. ^19^F {^1^H} NMR (282 MHz, CDCl_3_) δ −117.7, −121.5 ppm. FTIR (neat) ν_max_: 3297 (N–H_st_), 1699 (C=O_st_), 1601 (C=C_st_) cm^–1^. HRMS (ESI-TOF) *m*/*z*: [M + H]^+^, calcd for C_25_H_24_F_2_N_3_O 420.1809; found, 420.1819.

#### 1-(3-Chlorophenyl)-3-((3-chlorophenyl)amino)-4-((dimethylamino)methyl)-5-phenyl-1,5-dihydro-2*H*-pyrrol-2-one (**9g**)

The general procedure was followed using 1-(3-chlorophenyl)-3-((3-chlorophenyl)amino)-5-phenyl-1,5-dihydro-2*H*-pyrrol-2-one **8g** (0.395 g, 1 mmol, 1 equiv), affording 0.402 g (89%) of **9g** as yellow crystals after chromatography (hexanes/AcOEt 7:3) followed by crystallization (pentane/Et_2_O 3:1). mp (pentane/Et_2_O) = 128–130 °C. ^1^H NMR (400 MHz, CDCl_3_) δ 7.79 (t, ^4^*J*_HH_ = 2.1 Hz, 1H, CH_Ar_), 7.45 (ddd, ^3^*J*_HH_ = 8.3 Hz, ^4^*J*_HH_ = 2.3 Hz, ^4^*J*_HH_ = 1.0 Hz, 1H, CH_Ar_), 7.39–7.24 (m, 5H, 5 × CH_Ar_), 7.16 (t, ^3^*J*_HH_ = 8.0 Hz, 1H, CH_Ar_), 7.11 (t, ^3^*J*_HH_ = 8.0 Hz, 1H, CH_Ar_), 7.01 (ddd, ^3^*J*_HH_ = 8.0 Hz, ^4^*J*_HH_ = 2.0 Hz, ^4^*J*_HH_ = 0.9 Hz, 1H, CH_Ar_), 6.87 (ddd, ^3^*J*_HH_ = 8.0 Hz, ^4^*J*_HH_ = 2.0 Hz, ^4^*J*_HH_ = 0.9 Hz, 1H, CH_Ar_), 6.81 (t, ^4^*J*_HH_ = 2.1 Hz, 1H, CH_Ar_), 6.67 (ddd, ^3^*J*_HH_ = 8.3 Hz, ^4^*J*_HH_ = 2.3 Hz, ^4^*J*_HH_ = 1.0 Hz, 1H, CH_Ar_), 6.51 (s, 1H, NH), 5.69 (s, 1H, CH), 2.81 (d, ^2^*J*_HH_ = 14.3 Hz, 1H, CH_A_CH_B_), 2.74 (d, ^2^*J*_HH_ = 14.3 Hz, 1H, CH_A_CH_B_), 2.13 (s, 6H, 2 × NCH_3_) ppm. ^13^C {^1^H} NMR (101 MHz, CDCl_3_) δ 167.5 (C=O), 144.1 (C_quat_), 138.9 (C_quat_), 136.6 (C_quat_), 134.7 (C_quat_), 134.6 (C_quat_), 132.2 (C_quat_), 130.5 (C_quat_), 130.1 (CH_Ar_), 129.9 (CH_Ar_), 129.2 (2 × CH_Ar_), 128.5 (CH_Ar_), 126.9 (2 × CH_Ar_), 124.5 (CH_Ar_), 121.3 (CH_Ar_), 120.6 (CH_Ar_), 118.4 (CH_Ar_), 118.0 (CH_Ar_), 115.9 (CH_Ar_), 64.6 (CH), 55.5 (CH_2_), 45.5 (2 × NCH_3_) ppm. FTIR (neat) *n*_max_: 3315 (NH_st_), 1694 (C=O_st_), 1602 (C=C_st_) cm^–1^. HRMS (ESI-TOF) *m*/*z*: [M + H]^+^, calcd for C_25_H_24_Cl_2_N_3_O 452.1296; found, 452.1303.

#### 4-((Dimethylamino)methyl)-1-(2-fluorophenyl)-3-((2-fluorophenyl)amino)-5-phenyl-1,5-dihydro-2*H*-pyrrol-2-one (**9h**)

The general procedure was followed using 1-(2-fluorophenyl)-3-((2-fluorophenyl)amino)-5-phenyl-1,5-dihydro-2*H*-pyrrol-2-one **8h** (0.362 g, 1 mmol, 1 equiv), affording 0.403 g (96%) of **9h** as yellow crystals after chromatography (hexanes/AcOEt 8:2) followed by crystallization (pentane/Et_2_O 3:1). mp (pentane/Et_2_O) = 150–152 °C. ^1^H NMR (400 MHz, CDCl_3_) δ 7.35–7.20 (m, 6H, 6 × CH_Ar_), 7.13–6.93 (m, 6H, 6 × CH_Ar_), 6.90–6.82 (m, 1H, CH_Ar_), 6.57 (s, 1H, NH), 5.67 (s, 1H, CH), 2.85 (d, ^2^*J*_HH_ = 14.5 Hz, 1H, CH_A_CH_B_), 2.80 (d, ^2^*J*_HH_ = 14.5 Hz, 1H, CH_A_CH_B_), 2.11 (s, 6H, 2 × NCH_3_) ppm. ^13^C {^1^H} NMR 167.3 (C=O), 157.2 (d, ^1^*J*_FC_ = 250.0 Hz, C_quat_), 153.8 (d, ^1^*J*_FC_ = 243.0 Hz, C_quat_), 136.2 (C_quat_), 131.9 (C_quat_), 131.2 (C_quat_), 131.1 (d, ^2^*J*_FC_ = 11.2 Hz, C_quat_), 128.9 (2 × CH_Ar_), 128.5 (CH_Ar_), 128.4 (d, ^4^*J*_FC_ = 1.1 Hz, CH_Ar_), 128.2 (d, ^3^*J*_FC_ = 8.0 Hz, CH_Ar_), 127.6 (2 × CH_Ar_), 124.5 (d, ^2^*J*_FC_ = 11.5 Hz, C_quat_), 124.4 (d, ^3^*J*_FC_ = 3.6 Hz, CH_Ar_), 124.1 (d, ^3^*J*_FC_ = 3.6 Hz, CH_Ar_), 121.5 (d, ^3^*J*_FC_ = 7.2 Hz, CH_Ar_), 119.0 (d, ^4^*J*_FC_ = 2.1 Hz, CH_Ar_), 116.6 (d, ^2^*J*_FC_ = 20.3 Hz, CH_Ar_), 115.4 (d, ^2^*J*_CF_ = 19.0 Hz, CH_Ar_), 66.2 (d, ^4^*J*_FC_ = 4.5 Hz, CH), 55.8 (CH_2_), 45.5 (2 × NCH_3_) ppm. ^19^F {^1^H} NMR (282 MHz, CDCl_3_) δ −120.3, −131.0 ppm. FTIR (neat) *n*_max_: 3323 (NH_st_), 1685 (C=O_st_), 1600 (C=C_st_) cm^–1^. HRMS (ESI-TOF) *m*/*z*: [M + H]^+^, calcd for C_25_H_24_F_2_N_3_O 420.1887; found, 420.1889.

#### 4-((Dimethylamino)methyl)-5-phenyl-1-(3-(trifluoromethyl)phenyl)-3-((3-(trifluoromethyl)phenyl)amino)-1,5-dihydro-2*H*-pyrrol-2-one (**9i**)

The general procedure was applied using 5-phenyl-1-(3-(trifluoromethyl)phenyl)-3-((3-(trifluoromethyl)phenyl)amino)-1,5-dihydro-2*H*-pyrrol-2-one (0.462 g, 1 mmol, 1 equiv) **8i** to afford 0.478 g (92%) of **9i** as white crystals after flash column chromatography (hexanes/AcOEt 7:3) followed by crystallization (Et_2_O). mp (Et_2_O) = 114–116 °C. ^1^H NMR (400 MHz, CDCl_3_) δ 7.99 (s, 1H, CH_Ar_), 7.81 (d, ^3^*J*_HH_ = 8.2 Hz, 1H, CH_Ar_), 7.41–7.24 (m, 8H, 8 × CH_Ar_), 7.15 (d, ^3^*J*_HH_ = 7.7 Hz, 1H, CH_Ar_), 7.05 (s, 1H, CH_Ar_), 6.97 (d, ^3^*J*_HH_ = 8.1 Hz, 1H, CH_Ar_), 6.62 (s, 1H, NH), 5.74 (s, 1H, CH), 2.80 (d, ^2^*J*_HH_ = 14.7 Hz, 1H, CH_A_CH_B_), 2.76 (d, ^2^*J*_HH_ = 14.7 Hz, 1H, CH_A_CH_B_), 2.12 (s, 6H, 2 × NCH_3_) ppm. ^13^C {^1^H} NMR (101 MHz, CDCl_3_) δ 167.6 (C=O), 143.4 (C_quat_), 138.3 (C_quat_), 136.3 (C_quat_), 133.1 (C_quat_), 131.5 (q, ^2^*J*_FC_ = 32.1 Hz, C_quat_), 131.3 (q, ^2^*J*_FC_ = 32.5 Hz, C_quat_), 130.5 (C_quat_), 129.6 (CH_Ar_), 129.5 (CH_Ar_), 129.3 (2 × CH_Ar_), 128.7 (CH_Ar_), 124.1 (q, ^1^*J*_FC_ = 275.5 Hz, C_quat_), 123.9 (q, ^1^*J*_FC_ = 275.5 Hz, C_quat_), 126.9 (2 × CH_Ar_), 123.4 (CH_Ar_), 121.0 (q, ^3^*J*_FC_ = 3.8 Hz, CH_Ar_), 120.67 (CH_Ar_), 117.8 (q, ^3^*J*_FC_ = 3.8 Hz, CH_Ar_), 117.1 (q, ^3^*J*_FC_ = 4.0 Hz, CH_Ar_), 114.3 (q, ^3^*J*_FC_ = 3.9 Hz, CH_Ar_), 64.7 (CH), 55.6 (CH_2_), 45.5 (2 × NCH_3_) ppm. ^19^F {^1^H} NMR (282 MHz, CDCl_3_) δ −63.2, −63.3 ppm. FTIR (neat) *n*_max_: 3334 (NH_st_), 1688 (C=O_st_), 1611 (C=C_st_) cm^–1^. HRMS (ESI-TOF) *m*/*z*: [M + H]^+^, calcd for C_27_H_24_F_6_N_3_O 520.1824; found, 520.1834.

### General Procedure for the Synthesis and Isolation of Acetylated Lactam **10**

To a solution of 4-((dimethylamino)methyl)-5-phenyl-1-(*p*-tolyl)-3-(*p*-tolylamino)-1,5-dihydro-2*H*-pyrrol-2-one **9a** (0.367 g, 1 mmol, 1 equiv) in chloroform (3 mL), 12 equiv of acetic anhydride (1.1 mL) was added at room temperature. After 5 min, the solvent was evaporated and the obtained residue was dried in a vacuum pump, where the product crystallized spontaneously. The red crystals were washed with Et_2_O, affording pure **10**.

#### (5-Oxo-2-phenyl-1-(*p*-tolyl)-4-(*p*-tolylamino)-2,5-dihydro-1*H*-pyrrol-3-yl)methyl Acetate (**10**)

The general procedure was followed, affording 0.397 g (93%) of **10** as red crystals. mp (Acetic acid) = 162–164 °C. ^1^H NMR (400 MHz, CDCl_3_) δ 7.39 (d, ^3^*J*_HH_ = 8.5 Hz, 2H, 2 × CH_Ar_), 7.32–7.19 (m, 5H, 5 × CH_Ar_), 7.07 (d, ^3^*J*_HH_ = 8.3 Hz, 2H, 2 × CH_Ar_), 7.07 (d, ^3^*J*_HH_ = 8.5 Hz, 2H, 2 × CH_Ar_), 6.96 (d, ^3^*J*_HH_ = 8.3 Hz, 2H, 2 × CH_Ar_), 6.34 (s, 1H, NH), 5.57 (s, 1H, CH), 4.62 (d, ^2^*J*_HH_ = 13.3 Hz, 1H, CH_A_CH_B_), 4.22 (d, ^2^*J*_HH_ = 13.3 Hz, 1H, CH_A_CH_B_), 2.29 (s, 3H, CH_3Tol_), 2.25 (s, 3H, CH_3Tol_), 1.88 (s, 3H, CH_3_). ^13^C {^1^H} NMR (101 MHz, CDCl_3_) δ 170.6 (C=O), 167.0 (C=O), 138.2 (C_quat_), 136.7 (C_quat_), 134.8 (C_quat_), 134.6 (C_quat_), 133.1 (C_quat_), 132.6 (C_quat_), 129.8 (2 × CH_Ar_), 129.6 (2 × CH_Ar_), 129.1 (2 × CH_Ar_), 128.5 (CH_Ar_), 127.3 (2 × CH_Ar_), 121.9 (2 × CH_Ar_), 120.9 (2 × CH_Ar_), 115.3 (C_quat_), 65.4 (CH), 58.1 (CH_2_), 21.0 (CH_3Tol_), 20.9 (CH_3Tol_), 20.7 (CH_3_) ppm. FTIR (neat) *n*_max_: 3388 (NH_st_), 1739 (C=O_st_), 1675 (C=O_st_), 1615 (C=C_st_) cm^–1^. HRMS (ESI-TOF) *m*/*z*: [M + H]^+^, calcd for C_27_H_27_N_2_O_3_ 427.2022; found, 427.2025.

### General Procedure for the Synthesis and Isolation of Spirocyclic Dihydropyridines **5** and **5**′

A solution of the corresponding functionalized γ-lactam **2** or **9a** (1 mmol, 1 equiv) in chloroform (3 mL) was stirred at room temperature under the presence of 12 equiv of acetic anhydride. After 5 min, the formation of the acetylated intermediate **3** or **10a** was detected by NMR. Then, acetic acid was removed under low pressure and freshly distilled triethylamine (0.167 mL, 1.2 equiv) and CHCl_3_ (3 mL) were added to the reaction. The mixture was heated at 55 °C overnight. The reaction crude was acidified with 0.5 M HCl aqueous solution and extracted with dichloromethane (2 × 20 mL). The combined organic layers were dried with MgSO_4_ and purified by flash column chromatography, affording the corresponding spirocyclic dihydropyridine **5** or **5′**.

#### (*Z*)-1,1′,6′-Tri*p*-tolyl-4-(*p*-tolylimino)-3′,4′,5′,6′-tetrahydrospiro[pyrrolidine-3,2′-pyrrolo[3,4-*b*]pyridine]-5,7′(1′*H*)-dione (**5**)

The general procedure was followed, affording 0.476 g (82%) of **5** as a yellow solid after chromatography (hexanes/AcOEt 85:15). ^1^H NMR (400 MHz, CDCl_3_) δ 7.56 (d, ^3^*J*_HH_ = 8.5 Hz, 2H, 2 × CH_Ar_), 7.48 (d, ^3^*J*_HH_ = 8.6 Hz, 2H, 2 × CH_Ar_), 7.21–6.96 (m, 10H, 5 × CH_Ar_), 6.47 (d, ^3^*J*_HH_ = 8.3 Hz, H, CH_Ar_), 4.33 (s, 2H, CH_2_), 4.24 (d, ^2^*J*_HH_ = 10.2 Hz, 1H, CH_A_CH_B_), 3.87 (d, ^2^*J*_HH_ = 10.2 Hz, 1H, CH_A_CH_B_), 2.99–2.76 (m, 1H, CH_A_CH_B_), 2.70–2.44 (m, 2H, CH_2_), 2.33 (s, 3H, CH_3_), 2.32 (s, 3H, CH_3_), 2.30 (s, 3H, CH_3_), 2.29 (s, 3H, CH_3_), 2.23–2.13 (m, 1H, CH_A_CH_B_) ppm. ^13^C {^1^H} NMR (101 MHz, CDCl_3_) δ 165.0 (C=O), 161.7 (C=O), 156.7 (C_quat_), 146.2 (C_quat_), 139.8 (C_quat_), 137.2 (C_quat_), 136.6 (C_quat_), 136.2 (C_quat_), 136.2 (C_quat_), 135.8 (C_quat_), 134.0 (C_quat_), 133.2 (C_quat_), 129.7 (2 × CH_Ar_), 129.5 (4 × CH_Ar_), 128.9 (4 × CH_Ar_), 122.5 (C_quat_), 119.8 (2 × CH_Ar_), 118.4 (2 × CH_Ar_), 118.0 (2 × CH_Ar_), 61.5 (C_quat_), 56.0 (CH_2_), 50.8 (CH_2_), 29.8 (CH_2_), 21.2 (CH_3_), 21.1 (CH_3_), 21.0 (CH_3_), 20.8 (CH_3_), 19.5 (CH_2_) ppm. FTIR (neat) *n*_max_: 1696 (C=O_st_), 1669 (C=N_st_), 1627 (C=C_st_) cm^–1^. HRMS (ESI-TOF) *m*/*z*: [M + H]^+^, calcd for C_38_H_37_N_4_O_2_ 581.2917; found, 581.2912.

#### (2R*,3R*,5′R*,*Z*)-2,5′-Diphenyl-1,1′,6′-tri*p*-tolyl-4-(*p*-tolylimino)-3′,4′,5′,6′-tetrahydrospiro[pyrrolidine-3,2′-pyrrolo[3,4-*b*]pyridine]-5,7′(1′*H*)-dione (**5**′)

The general procedure was applied starting from (5-oxo-2-phenyl-1-(*p*-tolyl)-4-(*p*-tolylamino)-2,5-dihydro-1*H*-pyrrol-3-yl)methyl acetate **9a** (0.451 g, 1 mmol) to afford 0.322 g (88%) of **5′** as a yellow oil after chromatography (hexanes/AcOEt 9:1). ^1^H NMR (400 MHz, CDCl_3_) δ 7.68–6.80 (m, 24H, 24 × CH_Ar_), 6.30 (d, ^3^*J*_HH_ = 8.2 Hz, 2H, 2 × CH_Ar_), 5.52 (s, 1H, CH), 4.94 (s, 1H, CH), 2.54–2.05 (m, 2H, CH_2_), 2.35 (s, 3H, CH_3Tol_), 2.28 (s, 3H, CH_3Tol_), 2.24 (s, 3H, CH_3Tol_), 2.20 (s, 3H, CH_3Tol_), 1.67 (m, 1H, CH_A_CH_B_), 1.47 (m, 1H, CH_A_CH_B_) ppm. ^13^C NMR {^1^H} (101 MHz, CDCl_3_) δ 166.2 (C_quat_), 161.2 (C_quat_), 157.6 (C_quat_), 146.2 (C_quat_), 140.4 (C_quat_), 137.8 (C_quat_), 136.8 (C_quat_), 136.5 (C_quat_), 135.9 (C_quat_), 135.7 (C_quat_), 135.5 (C_quat_), 134.0 (C_quat_), 133.9 (C_quat_), 133.4 (C_quat_), 132.6 (C_quat_), 129.9 (2 × CH_Ar_), 129.8 (2 × CH_Ar_), 129.5 (CH_Ar_), 129.4 (2 × CH_Ar_), 129.3 (2 × CH_Ar_), 129.2 (2 × CH_Ar_), 129.1 (CH_Ar_), 128.8 (2 × CH_Ar_), 128.7 (2 × CH_Ar_), 128.2 (2 × CH_Ar_), 126.1 (2 × CH_Ar_), 121.6 (2 × CH_Ar_), 120.1 (2 × CH_Ar_), 117.6 (2 × CH_Ar_), 72.3 (CH), 66.9 (C_quat_), 65.0 (CH), 24.5 (CH_2_), 21.1 (CH_3_Tol), 21.0 (CH_3_Tol), 20.9 (CH_3_Tol), 20.7 (CH_3_Tol), 18.1 (CH_2_) ppm. FTIR (neat) *n*_max_: 1698 (C=O_st_), 1669 (C=N_st_), 1628 (C=C_st_) cm^–1^. HRMS (ESI-TOF) *m*/*z*: [M + H]^+^, calcd for C_50_H_45_N_4_O_2_ 733.3543; found, 733.3537.

### General Procedure for the Synthesis and Isolation of Bicycle **7**

A solution of the corresponding functionalized γ-lactam **2** (1 mmol, 1 equiv) in chloroform (3 mL) was stirred at room temperature under the presence of 12 equiv of acetic anhydride. After 5 min, the formation of the acetylated intermediate **3** was detected by NMR. Then, acetic acid was removed under low pressure and freshly distilled triethylamine (0.167 mL, 1.2 equiv), *N*-*p*-tolylmethanimine (0.273 mL, 2 equiv) and CHCl_3_ (3 mL) were added to the reaction. The mixture was heated at 55 °C overnight. The reaction crude was acidified with 0.5 M HCl aqueous solution and extracted with dichloromethane (2 × 20 mL). The combined organic layers were dried with MgSO_4_ and purified by flash column chromatography (hexanes/AcOEt 8:2), affording the corresponding bicycle **7**.

#### 1,3,6-Tri*p*-tolyl-1,2,3,4,5,6-hexahydro-7*H*-pyrrolo[3,4-*d*]pyrimidin-7-one (**7**)

The general procedure was followed, affording 0.172 g (42%) of **7** as a white solid. ^1^H NMR (400 MHz, CDCl_3_) δ 7.62 (d, ^3^*J*_HH_ = 8.6 Hz, 2H, 2 × CH_Ar_), 7.15 (d, ^3^*J*_HH_ = 8.4 Hz, 2H, 2 × CH_Ar_), 7.09 (d, ^3^*J*_HH_ = 8.4 Hz, 2H, 2 × CH_Ar_), 7.00 (d, ^3^*J*_HH_ = 8.6 Hz, 2H, 2 × CH_Ar_), 6.95 (d, ^3^*J*_HH_ = 8.4 Hz, 2H, 2 × CH_Ar_), 6.72 (d, ^3^*J*_HH_ = 8.6 Hz, 2H, 2 × CH_Ar_), 4.83 (s, 1H, CH), 4.41 (d, ^4^*J*_HH_ = 1.2 Hz, 2H, CH_2_), 4.20 (d, ^4^*J*_HH_ = 1.2 Hz, 2H, CH_2_), 2.32 (s, 3H, CH_3_), 2.30 (s, 3H, CH_3_), 2.23 (s, 3H, CH_3_) ppm. ^13^C {^1^H} NMR (101 MHz, CDCl_3_) δ 165.1 (C=O), 146.5 (C_quat_), 143.3 (C_quat_), 137.5 (C_quat_), 136.7 (C_quat_), 133.9 (C_quat_), 133.5 (C_quat_), 130.3 (C_quat_), 130.2 (2 × CH_Ar_), 130.0 (4 × CH_Ar_), 127.6 (C_quat_), 122.5 (2 × CH_Ar_), 118.9 (2 × CH_Ar_), 117.4 (2 × CH_Ar_), 71.5 (CH_2_), 50.3 (CH_2_), 48.3 (CH_2_), 21.3 (CH_3_), 21.2 (CH_3_), 20.9 (CH_3_) ppm. FTIR (neat) *n*_max_: 1687 (C=O_st_), 1631 (C=C_st_) cm^–1^. HRMS (ESI-TOF) *m*/*z*: [M + H]^+^, (and the scission of the tetrahydropyrimidine) calcd for C_19_H_19_N_2_O 291.1497; found, 291.1493.

### General Procedure for the Synthesis of Spiro Bicyclo[2.2.1]heptane Pyrrolidines **6** and **12**

A solution of the corresponding functionalized γ-lactam **2** or **9** (1 mmol, 1 equiv) in chloroform (3 mL) was stirred at room temperature under the presence of 12 equiv of acetic anhydride. After 5 min, the formation of the acetylated intermediate **3** or **10** was detected by NMR. Then, acetic acid was removed under low pressure and freshly distilled triethylamine (0.167 mL, 1.2 equiv), cyclopentadiene (0.5 mL, 6 equiv), and CHCl_3_ (3 mL) were added to the reaction. The mixture was heated at 55 °C overnight. The reaction crude was acidified with HCl 0.5 M aqueous solution and extracted with dichloromethane (2 × 20 mL). The combined organic layers were dried with MgSO_4_ and purified by chromatography, affording the corresponding spiro bicyclo[2.2.1]heptane pyrrolidines **6** or **12**.

#### (1S*,2R*,4S*,*Z*)-1′-(*p*-Tolyl)-4′-(*p*-tolylimino)spiro[bicyclo[2.2.1]heptane-2,3′-pyrrolidin]-5-en-5′-one (**6**)

The general procedure was applied using 4-((dimethylamino)methyl)-1-(*p*-tolyl)-3-(*p*-tolylamino)-1,5-dihydro-2*H*-pyrrol-2-one (0.335 g, 1 mmol, 1 equiv) **2** to afford 0.260 g (73%) of **6** as a yellow oil after flash column chromatography (hexanes/AcOEt 9:1). ^1^H NMR (400 MHz, CDCl_3_) δ 7.61 (d, ^3^*J*_HH_ = 8.6 Hz, 2H, 2 × CH_Ar_), 7.11 (d, ^3^*J*_HH_ = 8.6 Hz, 2H, 2 × CH_Ar_), 7.08 (d, ^3^*J*_HH_ = 8.6 Hz, 2H, 2 × CH_Ar_), 6.93 (d, ^3^*J*_HH_ = 8.4 Hz, 2H, 2 × CH_Ar_), 5.98 (ddt, ^3^*J*_HH_ = 5.6 Hz, ^3^*J*_HH_ = 2.7 Hz, ^4^*J*_HH_ = 1.3 Hz, 1H, = CH), 5.81 (dtd, ^3^*J*_HH_ = 5.7 Hz, ^3^*J*_HH_ = 3.0 Hz, ^4^*J*_HH_ = 2.0 Hz, 1H, = CH), 4.68 (ddq, ^3^*J*_HH_ = 6.1 Hz, ^3^*J*_HH_ = 2.9 Hz, ^4^*J*_HH_ = 1.5 Hz, 1H, CH), 4.25 (q, ^4^*J*_HH_ = 1.6 Hz, 2H, CH_2_), 2.78–2.64 (m, 1H, CH), 2.59 (ddtd, ^2^*J*_HH_ = 15.6 Hz, ^3^*J*_HH_ = 5.8 Hz, ^3^*J*_HH_ = 2.9 Hz, ^4^*J*_HH_ = 1.9 Hz, 1H, CH), 2.45 (dd, ^2^*J*_HH_ = 17.8 Hz, ^3^*J*_HH_ = 7.1 Hz, 1H, CH), 2.30 (s, 6H, 2 × CH_3_), 2.22–2.11 (m, 2H, CH_2_) ppm. ^13^C {^1^H} NMR (101 MHz, CDCl_3_) δ 165.3 (C=O), 144.1 (C_quat_), 137.6 (C_quat_), 135.8 (CH), 134.3 (C_quat_), 133.0 (C_quat_), 132.2 (C_quat_), 130.4 (CH), 129.5 (2 × CH_Ar_), 129.3 (2 × CH_Ar_), 125.9 (C_quat_), 122.0 (2 × CH_Ar_), 118.4 (2 × CH_Ar_), 70.0 (CH), 51.1 (CH_2_), 39.2 (CH_2_), 35.2 (CH), 25.9 (CH_2_), 21.0 (CH_3_), 20.9 (CH_3_) ppm. **FTIR** (neat) *n*_max_: 3027 (=CH_st_), 1704 (C=O_st_), 1678 (C=C_st_) cm^–1^. **HRMS** (ESI-TOF) *m*/*z*: [M + H]^+^, calcd for C_24_H_25_N_2_O 357.1967; found, 357.1965.

#### (1S*,2R*,2′R*,4S*,*Z*)-2′-Phenyl-1′-(*p*-tolyl)-4′-(*p*-tolylimino)spiro[bicyclo[2.2.1]heptane-2,3′-pyrrolidin]-5-en-5′-one (**12a**)

The general procedure was applied using 4-((dimethylamino)methyl)-5-phenyl-1-(*p*-tolyl)-3-(*p*-tolylamino)-1,5-dihydro-2*H*-pyrrol-2-one (0.412 g, 1 mmol, 1 equiv) **9a** to afford 0.359 g (83%) of **12a** as a yellow oil after flash column chromatography (hexanes/AcOEt 98:02). ^1^H NMR (400 MHz, CDCl_3_) δ 7.42 (d, ^3^*J*_HH_ = 8.6 Hz, 2H, 2 × CH_Ar_), 7.38–7.29 (m, 2H, 2 × CH_Ar_), 7.28 (d, ^3^*J*_HH_ = 7.3 Hz, 1H, CH_Ar_), 7.15 (d, ^3^*J*_HH_ = 8.5 Hz, 2H, 2 × CH_Ar_), 7.08 (d, ^3^*J*_HH_ = 8.5 Hz, 2H, 2 × CH_Ar_), 7.08 (d, ^3^*J*_HH_ = 8.2 Hz, 2H, 2 × CH_Ar_), 7.02 (d, ^3^*J*_HH_ = 8.2 Hz, 2H, 2 × CH_Ar_), 6.55 (dd, ^3^*J*_HH_ = 5.7 Hz, ^3^*J*_HH_ = 3.1 Hz, 1H, = CH), 6.42 (dd, ^3^*J*_HH_ = 5.7 Hz, ^3^*J*_HH_ = 3.1 Hz, 1H, = CH), 4.85 (s, 1H, CH), 3.12 (s, 1H, CH), 2.89 (s, 1H, CH), 2.35 (s, 3H, CH_3_), 2.24 (s, 3H, CH_3_), 2.22–2.13 (m, 2H, CH_2_), 1.48 (m, 1H, CH_A_CH_B_), 0.84 (dd, ^2^*J*_HH_ = 12.4 Hz, ^3^*J*_HH_ = 2.9 Hz, 1H, CH_A_CH_B_) ppm. ^13^C {^1^H} NMR (101 MHz, CDCl_3_) δ 165.3 (C=O), 159.0 (C_quat_), 147.4 (C_quat_), 142.6 (CH), 139.5 (C_quat_), 136.1 (C_quat_), 135.5 (C_quat_), 134.3 (CH), 133.4 (C_quat_), 129.4 (2 × CH_Ar_), 129.3 (2 × CH_Ar_), 129.2 (2 × CH_Ar_), 128.2 (CH), 126.7 (2 × CH_Ar_), 121.0 (2 × CH_Ar_), 118.2 (2 × CH_Ar_), 69.6 (CH), 58.1 (C_quat_), 51.9 (CH), 46.1 (CH_2_), 43.0 (CH), 34.9 (CH_2_), 21.2 (CH_3_), 21.0 (CH_3_) ppm. FTIR (neat) *n*_max_: 3031 (=CH_st_), 1701 (C=O_st_), 1675 (C=C_st_) cm^–1^. HRMS (ESI-TOF) *m*/*z*: [M + H]^+^, (and the scission of the spirocycle) calcd for C_25_H_23_N_2_O 367.1810; found, 367.1807.

#### (1S*,2R*,2′R*,4S*,*Z*)-1′,2′-Diphenyl-4′-(phenylimino)spiro[bicyclo[2.2.1]heptane-2,3′-pyrrolidin]-5-en-5′-one (**12b**)

The general procedure was applied using 4-((dimethylamino)methyl)-1,5-diphenyl-3-(phenylamino)-1,5-dihydro-2*H*-pyrrol-2-one **9b** (0.383 g, 1 mmol, 1 equiv) to afford 0.309 g (76%) of **12b** as a yellow oil after flash column chromatography (hexanes/AcOEt 98:2). ^1^H NMR (400 MHz, CDCl_3_) δ 7.44 (d, ^3^*J*_HH_ = 7.8 Hz, 2H, 2 × CH_Ar_), 7.26 (t, ^3^*J*_HH_ = 7.8 Hz, 4H, 4 × CH_Ar_), 7.20 (d, ^3^*J*_HH_ = 7.0 Hz, 1H, CH_Ar_), 7.17–7.09 (m, 2H, 2 × CH_Ar_), 7.06–6.98 (m, 4H, 4 × CH_Ar_), 6.82 (d, ^3^*J*_HH_ = 7.4 Hz, 2H, 2 × CH_Ar_), 6.47 (dd, ^3^*J*_HH_ = 5.7 Hz, ^3^*J*_HH_ = 3.1 Hz, 1H, = CH), 6.34 (dd, ^3^*J*_HH_ = 5.7 Hz, ^3^*J*_HH_ = 3.1 Hz, 1H, = CH), 4.79 (s, 1H, CH), 3.05 (s, 1H, CH), 2.82 (s, 1H, CH), 2.33–2.10 (m, 1H, CH), 2.08 (d, ^3^*J*_HH_ = 3.7 Hz, 1H, CH), 1.71–1.15 (m, 1H, CH_A_CH_B_), 0.78 (dd, ^2^*J*_HH_ = 12.4 Hz, ^3^*J*_HH_ = 3.0 Hz, 1H, CH_A_CH_B_) ppm. ^13^C {^1^H} NMR (101 MHz, CDCl_3_) δ 165.5 (C=O), 159.1 (C_quat_), 150.1 (C_quat_), 142.7 (CH), 139.5 (C_quat_), 138.5 (C_quat_), 134.3 (CH), 129.4 (2 × CH_Ar_), 128.9 (2 × CH_Ar_), 128.6 (2 × CH_Ar_), 128.4 (CH), 126.7 (2 × CH_Ar_), 125.8 (CH), 124.0 (CH), 121.2 (2 × CH_Ar_), 118.0 (2 × CH_Ar_), 69.6 (CH), 58.1 (C_quat_), 52.0 (CH), 46.1 (CH_2_), 43.0 (CH), 34.9 (CH_2_) ppm. FTIR (neat) *n*_max_: 3061 (=CH_st_), 1699 (C=O_st_), 1682 (C=C_st_) cm^–1^. HRMS (ESI-TOF) *m*/*z*: [M + H]^+^, calcd for C_28_H_25_N_2_O 405.1967; found, 405.1965.

#### (1S*,2R*,2′R*,4S*,*Z*)-1′-(4-Methoxyphenyl)-4′-((4-methoxyphenyl)imino)-2′-phenylspiro[bicyclo[2.2.1]heptane-2,3′-pyrrolidin]-5-en-5′-one (**12c**)

The general procedure was applied using 4-((dimethylamino)methyl)-1-(4-methoxyphenyl)-3-((4-methoxyphenyl)amino)-5-phenyl-1,5-dihydro-2H-pyrrol-2-one (0.444 g, 1 mmol, 1 equiv) **9c** to afford 0.218 g (47%) of **12c** as yellow crystals after flash column chromatography (hexanes/AcOEt 98:2) followed by crystallization (hexanes/CHCl_3_ 3:1). mp (hexanes/CHCl_3_) = 124–126 °C. ^1^H NMR (400 MHz, CDCl_3_) δ 7.43–7.40 (m, 2H, 2 × CH_Ar_), 7.36–7.31 (m, 2H, 2 × CH_Ar_), 7.29–7.24 (m, 1H, CH_Ar_), 7.08 (dd, ^3^*J*_HH_ = 8.3 Hz, ^3^*J*_HH_ = 1.4 Hz, 2H, 2 × CH_Ar_), 6.99–6.87 (m, 4H, 4 × CH_Ar_), 6.79–6,72 (m, 2H, 2 × CH_Ar_), 6.55 (dd, ^3^*J*_HH_ = 5.7 Hz, ^3^*J*_HH_ = 2.9 Hz, 1H, = CH), 6.43 (dd, ^3^*J*_HH_ = 5.7 Hz, ^3^*J*_HH_ = 2.9 Hz, 1H, = CH), 4.81 (s, 1H, CH), 3.82 (s, 3H, OCH_3_), 3.72 (s, 3H, OCH_3_), 3.12 (s, 1H, CH), 2.90 (s, 1H, CH), 2.23–2.13 (m, 2H, CH_2_), 1.52–1.46 (m, 1H, CH_A_CH_B_), 0.83 (dd, ^2^*J*_HH_ = 12.3 Hz, ^3^*J*_HH_ = 2.9 Hz, 1H, CH_A_CH_B_) ppm. ^13^C {^1^H} NMR (101 MHz, CDCl_3_) δ 165.0 (C=O), 159.1 (C_quat_), 157.5 (C_quat_), 156.8 (C_quat_), 142.7 (C_quat_), 142.6 (CH), 139.6 (C_quat_), 134.4 (CH), 131.7 (C_quat_), 129.3 (2 × CH_Ar_), 128.8 (CH), 126.8 (2 × CH_Ar_), 123.0 (2 × CH_Ar_), 120.3 (2 × CH_Ar_), 114.1 (2 × CH_Ar_), 113.1 (2 × CH_Ar_), 70.2 (CH), 58.2 (C_quat_), 55.5 (OCH_3_), 55.4 (OCH_3_), 52.0 (CH), 46.1 (CH_2_), 43.0 (CH), 34.9 (CH_2_) ppm. FTIR (neat) *n*_max_: 3056 (=CH_st_), 1690 (C=O_st_), 1645 (C=C_st_) cm^–1^. HRMS (ESI-TOF) *m*/*z*: [M + H]^+^ calcd for C_30_H_29_N_2_O_3_ 465.2178; found, 465.2166.

#### (1S*,2R*,2′R*,4S*,*Z*)-1′-(4-Bromophenyl)-4′-((4-bromophenyl)imino)-2′-phenylspiro[bicyclo[2.2.1]heptane-2,3′-pyrrolidin]-5-en-5′-one (**12d**)

The general procedure was applied using 1-(4-bromophenyl)-3-((4-bromophenyl)amino)-4-((dimethylamino)methyl)-5-phenyl-1,5-dihydro-2*H*-pyrrol-2-one (0.541 g, 1 mmol, 1 equiv) **9d** to afford 0.427 g (76%) of **12d** as yellow crystals after flash column chromatography (hexanes/AcOEt 95:5) followed by crystallization (hexanes/CHCl_3_ 3:1). mp (hexanes/CHCl_3_) = 200–201 °C. ^1^H NMR (400 MHz, CDCl_3_) δ 7.45 (d, ^3^*J*_HH_ = 8.9 Hz, 2H, 2 × CH_Ar_), 7.44 (d, ^3^*J*_HH_ = 8.9 Hz, 2H, 2 × CH_Ar_), 7.36 (d, ^3^*J*_HH_ = 7.0 Hz, 2H, 2 × CH_Ar_), 7.35–7.32 (m, 2H, 2 × CH_Ar_), 7.30 (d, ^3^*J*_HH_ = 7.0 Hz, 1H, CH_Ar_), 7.03 (d, ^3^*J*_HH_ = 8.6 Hz, 2H, 2 × CH_Ar_), 6.78 (d, ^3^*J*_HH_ = 8.6 Hz, 2H, 2 × CH_Ar_), 6.58 (dd, ^3^*J*_HH_ = 5.6 Hz, ^3^*J*_HH_ = 2.9 Hz, 1H, = CH), 6.42 (dd, ^3^*J*_HH_ = 5.6 Hz, ^3^*J*_HH_ = 2.9 Hz, 1H, = CH), 4.85 (s, 1H, CH), 3.11 (s, 1H, CH), 2.91 (s, 1H, CH), 2.22–2.02 (m, 2H, CH_2_), 1.53 (d, ^3^*J*_HH_ = 3.3 Hz, 1H, CH_A_CH_B_), 0.87 (dd, ^2^*J*_HH_ = 12.5 Hz, ^3^*J*_HH_ = 3.3 Hz, 1H, CH_A_CH_B_) ppm. ^13^C {^1^H} NMR (101 MHz, CDCl_3_) δ 165.9 (C=O), 158.9 (C_quat_), 148.9 (C_quat_), 142.9 (CH), 138.8 (C_quat_), 137.4 (C_quat_), 134.1 (CH), 132.1 (2 × CH_Ar_), 131.7 (2 × CH_Ar_), 129.6 (2 × CH_Ar_), 128.7 (CH), 126.6 (2 × CH_Ar_), 122.5 (2 × CH_Ar_), 119.9 (2 × CH_Ar_), 119.2 (C_quat_), 117.2 (C_quat_), 69.5 (CH), 58.1 (C_quat_), 52.2 (CH), 46.2 (CH_2_), 43.0 (CH), 35.1 (CH_2_) ppm. FTIR (neat) *n*_max_: 3048 (=CH_st_), 1696 (C=O_st_), 1672 (C=C_st_) cm^–1^. HRMS (ESI-TOF) *m*/*z*: [M + H]^+^, calcd for C_28_H_23_Br_2_N_2_O 563.0157; found, 563.0149.

#### (1S*,2R*,2′R*,4S*,*Z*)-1′-(4-Chlorophenyl)-4′-((4-chlorophenyl)imino)-2′-phenylspiro[bicyclo[2.2.1]heptane-2,3′-pyrrolidin]-5-en-5′-one (**12e**)

The general procedure was applied using 4-((dimethylamino)methyl)-1-(4-chlorophenyl)-3-((4-chlorophenyl)amino)-5-phenyl-1,5-dihydro-2*H*-pyrrol-2-one (0.452 g, 1 mmol, 1 equiv) **9e** to afford 0.307 g (68%) of **12e** as a yellow oil after flash column chromatography (pentane/Et_2_O 98:2). ^1^H NMR (400 MHz, CDCl_3_) δ 7.53–7.44 (m, 2H, 2 × CH_Ar_), 7.40–7.27 (m, 5H, 5 × CH_Ar_), 7.22–7.17 (m, 2H, 2 × CH_Ar_), 7.05–7.03 (m, 2H, 2 × CH_Ar_), 6.86–6.82 (m, 2H, 2 × CH_Ar_), 6.58 (dd, ^3^*J*_HH_ = 5.7 Hz, ^4^*J*_HH_ = 3.1 Hz, 1H, = CH), 6.43 (dd, ^3^*J*_HH_ = 5.7 Hz, ^4^*J*_HH_ = 3.0 Hz, 1H, = CH), 4.85 (s, 1H, CH), 3.11 (s, 1H, CH), 2.91 (s, 1H, CH), 2.14 (dd, ^3^*J*_HH_ = 8.5 Hz, ^3^*J*_HH_ = 4.0 Hz, 2H, CH_2_), 1.55–1.46 (m, 1H, CH_A_CH_B_), 0.87 (dd, ^2^*J*_HH_ = 12.5 Hz, ^4^*J*_HH_ = 3.0 Hz, 1H, CH_A_CH_B_) ppm. ^13^C {^1^H} NMR (101 MHz, CDCl_3_) δ 165.9 (C=O), 159.0 (C_quat_), 148.4 (C_quat_), 151.4 (C_quat_), 143.0 (CH), 138.9 (C_quat_), 137.0 (C_quat_), 134.1 (CH), 131.4 (C_quat_), 129.5 (2 × CH_Ar_), 129.4 (C_quat_), 129.1 (2 × CH_Ar_), 128.7 (2 × CH_Ar_), 128.6 (CH_Ar_), 126.6 (2 × CH_Ar_), 122.3 (2 × CH_Ar_), 119.6 (2 × CH_Ar_), 69.6 (CH_2_), 58.1 (C_quat_), 52.2 (CH_2_), 46.2 (CH), 43.0 (CH_2_), 35.1 (CH) ppm. FTIR (neat) *n*_max_: 3057 (=CH_st_), 1708 (C=O_st_), 1682 (C=C_st_) cm^–1^. HRMS (ESI-TOF) *m*/*z*: [M + H]^+^, calcd for C_28_H_23_Cl_2_N_2_O 473.1187; found, 473.1178.

#### (1S*,2R*,2′R*,4S*,*Z*)-1′-(4-Fluorophenyl)-4′-((4-fluorophenyl)imino)-2′-phenylspiro[bicyclo[2.2.1]heptane-2,3′-pyrrolidin]-5-en-5′-one (**12f**)

The general procedure was applied using 4-((dimethylamino)methyl)-1-(4-fluorophenyl)-3-((4-fluorophenyl)amino)-5-phenyl-1,5-dihydro-2*H*-pyrrol-2-one (0.419 g, 1 mmol, 1 equiv) **9f** to afford 0.216 g (49%) of **12f** as a yellow oil after flash column chromatography (pentane/Et_2_O 98:2). ^1^H NMR (400 MHz, CDCl_3_) δ 7.50–7.42 (m, 2H, 2 × CH_Ar_), 7.40–7.28 (m, 3H, 3 × CH_Ar_), 7.10–7.00 (m, 4H, 4 × CH_Ar_), 6.97–6.80 (m, 4H, 4 × CH_Ar_), 6.57 (dd, ^3^*J*_HH_ = 5.7 Hz, ^4^*J*_HH_ = 3.1 Hz, 1H, = CH), 6.43 (dd, ^3^*J*_HH_ = 5.7 Hz, ^4^*J*_HH_ = 3.1 Hz, 1H, = CH), 4.82 (s, 1H, CH), 3.12 (s, 1H, CH), 2.91 (s, 1H, CH), 2.23–1.98 (m, 2H, CH_2_), 1.53–1.49 (m, 1H, CH_A_CH_B_), 0.85 (dd, ^2^*J*_HH_ = 12.4 Hz, ^4^*J*_HH_ = 3.0 Hz, 1H, CH_A_CH_B_) ppm. ^13^C {^1^H} NMR (101 MHz, CDCl_3_) δ 166.2 (C=O), 160.7 (d, ^1^*J*_FC_ = 246.7 Hz, C_quat_), 160.4 (d, ^1^*J*_FC_ = 242.0 Hz, C_quat_), 159.4 (C_quat_), 146.0 (d, ^4^*J*_FC_ = 2.9 Hz, C_quat_), 143.2 (CH), 139.4 (C_quat_), 134.8 (d, ^4^*J*_FC_ = 3.0 Hz, C_quat_), 134.5 (CH), 129.8 (2 × CH_Ar_), 128.9 (2 × CH_Ar_), 127.0 (CH_Ar_), 123.6 (d, ^3^*J*_FC_ = 8.0 Hz, 2 × CH_Ar_), 120.1 (d, ^3^*J*_FC_ = 8.1 Hz, 2 × CH_Ar_), 116.1 (d, ^2^*J*_FC_ = 22.5 Hz, 2 × CH_Ar_), 115.6 (d, ^2^*J*_FC_ = 22.6 Hz, 2 × CH_Ar_), 70.4 (CH), 58.5 (C_quat_), 52.4 (CH), 46.4 (CH_2_), 43.4 (CH), 35.4 (CH_2_) ppm. ^19^F {^1^H} NMR (282 MHz, CDCl_3_) δ −115.4, −120.0 ppm. FTIR (neat) *n*_max_: 3057 (=CH_st_), 1704 (C=O_st_), 1673 (C=C_st_) cm^–1^. **HRMS** (ESI-TOF) *m*/*z*: [M + H]^+^, calcd for C_28_H_23_F_2_N_2_O 441.1778; found, 441.1765.

#### (1S*,2R*,2′R*,4S*,*Z*)-1′-(3-Chlorophenyl)-4′-((3-chlorophenyl)imino)-2′-phenylspiro[bicyclo[2.2.1]heptane-2,3′-pyrrolidin]-5-en-5′-one (**12g**)

The general procedure was applied using 1-(3-chlorophenyl)-3-((3-chlorophenyl)amino)-4-((dimethylamino)methyl)-5-phenyl-1,5-dihydro-2H-pyrrol-2-one (0,452 g, 1 mmol, 1 equiv) **9g** to afford 0.123 g (26%) of **12g** as yellow crystals after flash column chromatography (pentane/Et_2_O 8:2) followed by crystallization (pentane/Et_2_O 3:1). mp (pentane/Et_2_O) = 189–191 °C. ^1^H NMR (400 MHz, CDCl_3_) δ 7.76 (t, ^4^*J*_HH_ = 2.1 Hz, 1H, CH_Ar_), 7.37 (t, ^3^*J*_HH_ = 7.5 Hz, 2H, 2 × CH_Ar_), 7.35–7.27 (m, 1H, CH_Ar_), 7.29 (d, ^3^*J*_HH_ = 7.5 Hz, 2H, 2 × CH_Ar_), 7.13 (d, ^3^*J*_HH_ = 8. 0 Hz, 2H, 2 × CH_Ar_), 7.08 (d, ^4^*J*_HH_ = 1.2 Hz, 2H, 2 × CH_Ar_), 7.05 (s, 1H, CH_Ar_), 6.90 (d, ^4^*J*_HH_ = 2.1 Hz, 1H, CH_Ar_), 6.77 (ddd, ^3^*J*_HH_ = 8.0 Hz, ^4^*J*_HH_ = 2.1 Hz, ^4^*J*_HH_ = 1.2 Hz, 1H, CH_Ar_), 6.59 (dd, ^3^*J*_HH_ = 5.8 Hz, ^3^*J*_HH_ = 3.1 Hz, 1H, = CH), 6.44 (dd, ^3^*J*_HH_ = 5.8 Hz, ^3^*J*_HH_ = 3.1 Hz, 1H, = CH), 4.87 (s, 1H, CH), 3.12 (s, 1H, CH), 2.92 (s, 1H, CH), 2.17–2.11 (m, 2H, CH_2_), 1.54–1.50 (m, 1H, CH_A_CH_B_), 0.87 (dd, ^2^*J*_HH_ = 12.5 Hz, ^3^*J*_HH_ = 3.0 Hz, 1H, CH_A_CH_B_) ppm. ^13^C {^1^H} NMR (101 MHz, CDCl_3_) δ 166.0 (C=O), 158.7 (C_quat_), 151.1 (C_quat_), 142.8 (CH), 139.3 (C_quat_), 138.6 (C_quat_), 134.7 (C_quat_), 134.3 (C_quat_), 134.0 (CH), 129.8 (CH), 129.6 (CH), 129.5 (2 × CH_Ar_), 128.6 (CH), 126.4 (2 × CH_Ar_), 125.9 (CH), 123.9 (CH), 121.1 (CH), 118.5 (CH), 118,1 (CH), 116.0 (CH), 69.4 (CH), 58.0 (C_quat_), 52.0 (CH), 46.0 (CH_2_), 42.9 (CH), 35.0 (CH_2_) ppm. FTIR (neat) *n*_max_: 3025 (=CH_st_), 1703 (C=O_st_), 1677 (C=C_st_) cm^–1^. HRMS (ESI-TOF) *m*/*z*: [M + H]^+^, calcd for C_28_H_23_Cl_2_N_2_O 473.1187; found, 473.1178.

#### (1S*,2R*,2′R*,4S*,*Z*)-1′-(2-Fluorophenyl)-4′-((2-fluorophenyl)imino)-2′-phenylspiro[bicyclo[2.2.1]heptane-2,3′-pyrrolidin]-5-en-5′-one (**12h**)

The general procedure was applied using 4-((dimethylamino)methyl)-1-(2-fluorophenyl)-3-((2-fluorophenyl)amino)-5-phenyl-1,5-dihydro-2*H*-pyrrol-2-one (0.419 g, 1 mmol, 1 equiv) **9h** to afford 0.238 g (54%) of **12h** as yellow crystals after flash column chromatography (hexanes/AcOEt 95:5) followed by crystallization (hexanes/CHCl_3_ 3:1). mp (hexanes/CHCl_3_) = 166–168 °C. ^1^H NMR (400 MHz, CDCl_3_) δ 7.49–7.41 (m, 4H, 4 × CH_Ar_), 7.37–7.33 (m, 1H, CH_Ar_), 7.29–7.23 (m, 5H, 5 × CH_Ar_), 7.19 (d, ^3^*J*_HH_ = 7.5 Hz, 2H, 2 × CH_Ar_), 7.13 (t, ^3^*J*_HH_ = 7.5 Hz, 1H, CH_Ar_), 6.67 (3, 2H, = CH), 5.02 (s, 1H, CH), 3.48 (s, 1H, CH), 3.09 (s, 1H, CH), 2.49–2.41 (m, 2H, CH_2_), 1.69 (d, ^3^*J*_HH_ = 8.9 Hz, 1H, CH_A_CH_B_), 0.98 (d, ^2^*J*_HH_ = 12.4 Hz, 1H, CH_A_CH_B_) ppm. ^13^C {^1^H} NMR (101 MHz, CDCl_3_) δ 168.3 (C=O), 159.2 (C_quat_), 157.4 (d, ^1^*J*_FC_ = 250.7 Hz, C_quat_), 151.4 (d, ^1^*J*_FC_ = 243.4 Hz, C_quat_), 142.5 (CH), 138.8 (C_quat_), 137.5 (d, ^2^*J*_FC_ = 13.4 Hz, C_quat_), 134.6 (CH), 129.5 (d, ^3^*J*_FC_ = 8.0 Hz, CH_Ar_), 129.1 (2 × CH_Ar_), 128.73 (CH_Ar_), 128.4 (2 × CH_Ar_), 127.5 (CH_Ar_), 125.0 (d, ^3^*J*_FC_ = 7.6 Hz, CH_Ar_), 124.8 (d, ^2^*J*_FC_ = 11.6 Hz, C_quat_), 124.5 (d, ^3^*J*_FC_ = 3.6 Hz, CH_Ar_), 124.1 (d, ^3^*J*_FC_ = 3.6 Hz, CH_Ar_), 121.3 (d, ^4^*J*_FC_ = 2.2 Hz, CH_Ar_), 116.6 (d, ^2^*J*_FC_ = 19.6 Hz, CH_Ar_), 115.4 (d, ^2^*J*_FC_ = 20.3 Hz, CH_Ar_), 71.1 (d, ^4^*J*_FC_ = 3.6 Hz, CH), 58.2 (C_quat_), 52.5 (CH), 45.9 (CH_2_), 43.3 (CH), 35.7 (CH_2_) ppm. ^19^F {^1^H} NMR (282 MHz, CDCl_3_) δ −119.9, −127.7 ppm. FTIR (neat) *n*_max_: 3056 (=CH_st_), 1717 (C=O_st_), 1681 (C=C_st_) cm^–1^. HRMS (ESI-TOF) *m*/*z*: [M + H]^+^, calcd for C_28_H_23_F_2_N_2_O 441.1778; found, 441.1775.

#### (1S*,2R*,2′R*,4S*,*Z*)-2′-Phenyl-1′-(3-(trifluoromethyl)phenyl)-4′-((3-(trifluoromethyl)phenyl)imino)spiro[bicyclo[2.2.1]heptane-2,3′-pyrrolidin]-5-en-5′-one (**12i**)

The general procedure was applied using 4-((dimethylamino)methyl)-5-phenyl-1-(3-(trifluoromethyl)phenyl)-3-((3-(trifluoromethyl)phenyl)amino)-1,5-dihydro-2H-pyrrol-2-one (0.519 g, 1 mmol, 1 equiv) **9i** to afford 0.222 g (41%) of **12i** as a yellow solid after flash column chromatography (Pentane/Et_2_O 8:2). ^1^H NMR (400 MHz, CDCl_3_) δ 7.89 (s, 1H, CH_Ar_), 7.68–7.63 (m, 1H, CH_Ar_), 7.46 (t, ^3^*J*_HH_ = 7.8 Hz, 1H, CH_Ar_), 7.41–7.30 (m, 6H, 6 × CH_Ar_), 7.15 (s, 1H, CH_Ar_), 7.07 (dt, ^3^*J*_HH_ = 8.3 Hz, ^3^*J*_HH_ = 2.5 Hz, 3H, 3 × CH_Ar_), 6.60 (dd, ^3^*J*_HH_ = 5.6 Hz, ^3^*J*_HH_ = 3.1 Hz, 1H, = CH), 6.46 (dd, ^3^*J*_HH_ = 5.6 Hz, ^3^*J*_HH_ = 3.1 Hz, 1H, = CH), 4.91 (s, 1H, CH), 3.17 (s, 1H, CH), 2.94 (s, 1H, CH), 2.21–2.14 (m, 2H, CH_2_), 1.54 (s, 1H, CH_A_CH_B_), 0.91 (dd, ^2^*J*_HH_ = 12.4 Hz, ^3^*J*_HH_ = 3.0 Hz, 1H, CH_A_CH_B_) ppm. ^13^C {^1^H} NMR (101 MHz, CDCl_3_) δ 166.7 (C=O), 159.2 (C_quat_), 150.5 (C_quat_), 143.4 (=CH), 139.2 (C_quat_),138.9 (C_quat_), 134.1 (=CH), 131.8 (q, ^2^*J*_FC_ = 37.4 Hz, C_quat_), 131.5 (q, ^2^*J*_FC_ = 37.4 Hz, C_quat_), 130.0 (2 × CH_Ar_), 129.9 (2 × CH_Ar_), 129.4 (CH_Ar_), 129.1 (CH_Ar_), 126.9 (CH_Ar_), 124.2 (CH_Ar_), 122.9 (d, ^3^*J*_FC_ = 4.0 Hz, CH_Ar_), 121.6 (CH_Ar_), 121.2 (d, ^3^*J*_FC_ = 4.0 Hz, CH_Ar_), 118.1 (d, ^3^*J*_FC_ = 4.0 Hz, CH_Ar_), 115.6 (d, ^3^*J*_FC_ = 4.0 Hz, CH_Ar_), 70.0 (CH), 58.5 (C_quat_), 52.6 (CH), 46.5 (CH_2_), 43.4 (CH), 35.6 (CH_2_) ppm. ^19^F {^1^H} NMR (282 MHz, CDCl_3_) δ −62.5, −62.8 ppm. FTIR (neat) *n*_max_: 3045 (=CH_st_), 1699 (C=O_st_), 1664 (C=C_st_) cm^–1^. HRMS (ESI-TOF) *m*/*z*: [M + H]^+^, calcd for C_30_H_23_F_6_N_2_O 541.1715; found, 541.1711.

## Data Availability

The data underlying this study are available in the published article and its Supporting Information.

## Supporting Information Available

Copies of ^1^H, ^13^C, and ^19^F NMR spectra for compounds **1**, **2**, **5**, **5′**, **6**, **7**, **8**, **9**, **10**, and **12**, 2D NMR spectra for compounds **5**, **7**, **9a**, **12a**, and **12h**, and cif file and thermal ellipsoid plot for **12d** and **12h**; and Cartesian coordinates, energies, and number of imaginary frequencies of local minima and transition structures discussed in this work computed at the DFT level (PDF)
