# Supplementary material for: Exploring the Reactivity of Rigid 1-Azadienes Derived from Methylene γ-Lactams. Applications to the Stereoselective Synthesis of Spiro-γ-Lactams
Source: J Org Chem. 2024 Jun 19;89(13):9502–15. doi: 10.1021/acs.joc.4c00822 (PMC11232019; doi:10.1021/acs.joc.4c00822)
Supplement: Supplementary file 1 — jo4c00822_si_001.pdf [file jo4c00822_si_001.pdf]

Supporting Information for

Exploring the Reactivity of Rigid 1-Azadienes Derived from Methylene γ-Lactams. Applications to the Stereoselective Synthesis of Spiro-γ-Lactams.

Adrián López-Francés,[a] Zuriñe Serna-Burgos,[a] Xabier del Corte,[a] Jesús M. de los Santos,[a] Abel de Cózar[b,c]* and Javier Vicario[a]*

[a] Department of Organic Chemistry I, Faculty of Pharmacy and Lascaray Research Center, University of the Basque Country, UPV/EHU. Paseo de la Universidad 7, 01006 Vitoria-Gasteiz, Spain.

[b] Department of Organic Chemistry I, Donostia International Physics Center (DIPC), University of the Basque Country, UPV/EHU. Paseo Manuel de Lardizabal, 3, 20018 Donostia-San Sebastián, Spain.

[c] Ikerbasque, Basque Foundation for Science, Plaza Euskadi 5, 48009, Bilbao, Spain.

Table of Contents

I. NMR spectra copies of **1**, **2**, **5**, **6**, **7**, **8**, **9**, **10** and **12**................................ S1

II. 2D NMR spectra copies of **9a**, **5**, **7**, **12a** and **12h** S43

III. Crystallography of **12d** and **12h** S53

IV. Computational methods S59

I. NMR Spectra Copies

1-(*p*-Tolyl)-3-(*p*-tolylamino)-1,5-dihydro-2*H*-pyrrol-2-one (1)

^{1}H NMR (400 MHz, $CDCl_3$)

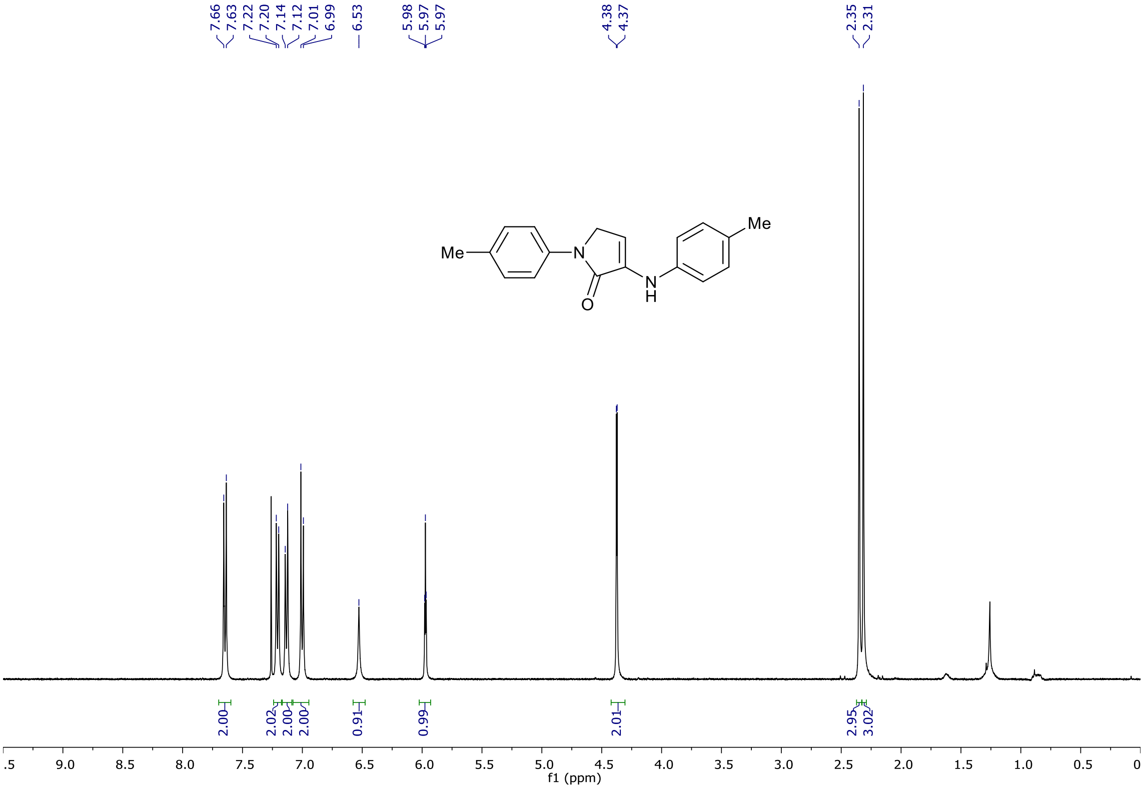

^{13}C {^{1}H} NMR (101 MHz, $CDCl_3$)

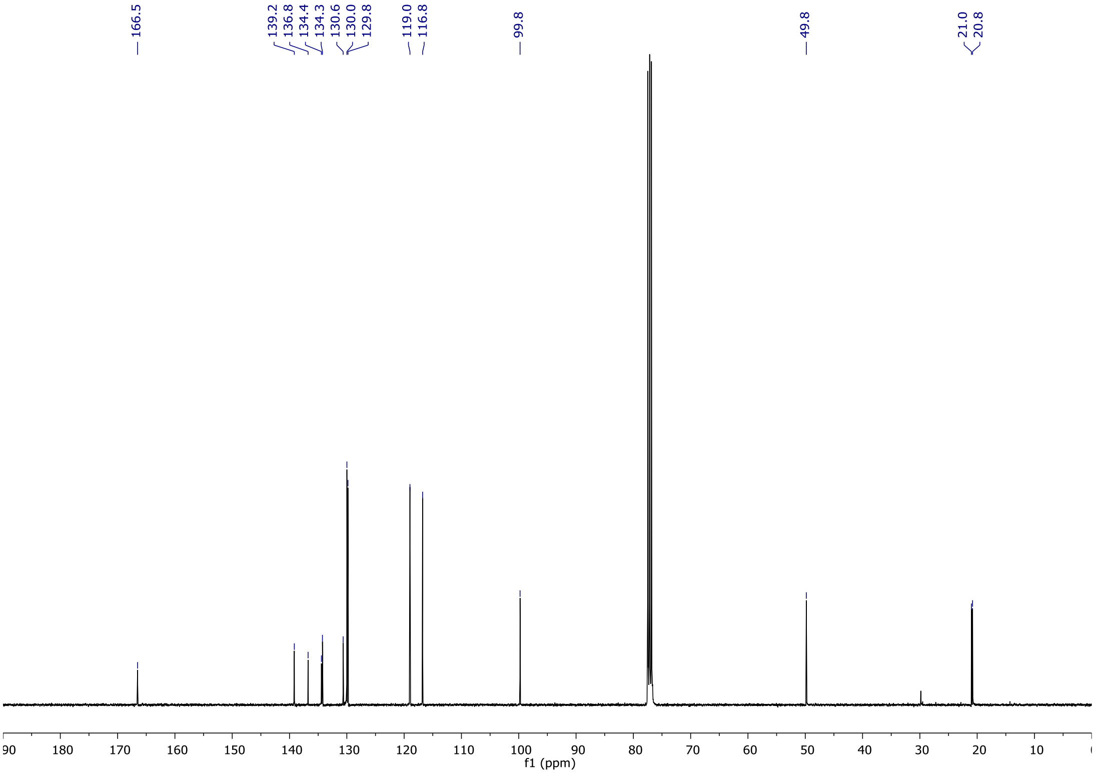

5-Phenyl-1-(*p*-tolyl)-3-(*p*-tolylamino)-1,5-dihydro-2*H*-pyrrol-2-one (8a).

^{1}H NMR (400 MHz, $CDCl_3$)

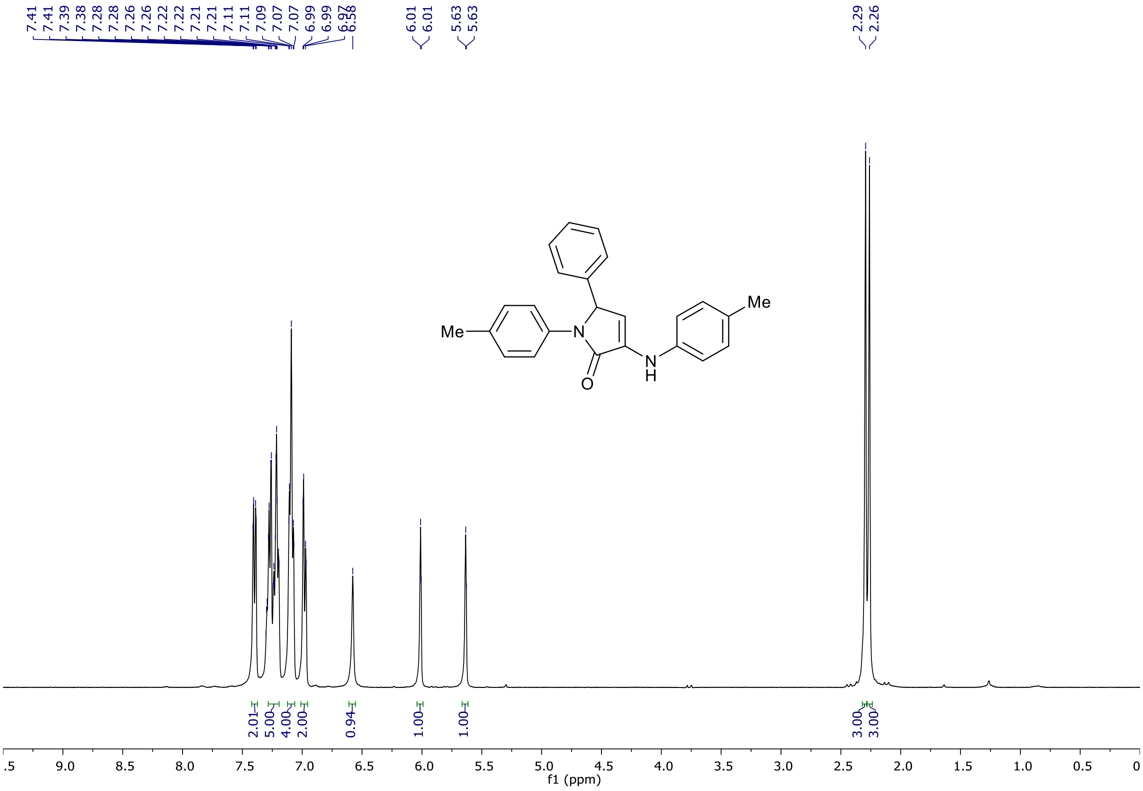

^{13}C {^{1}H} NMR (101 MHz, $CDCl_3$)

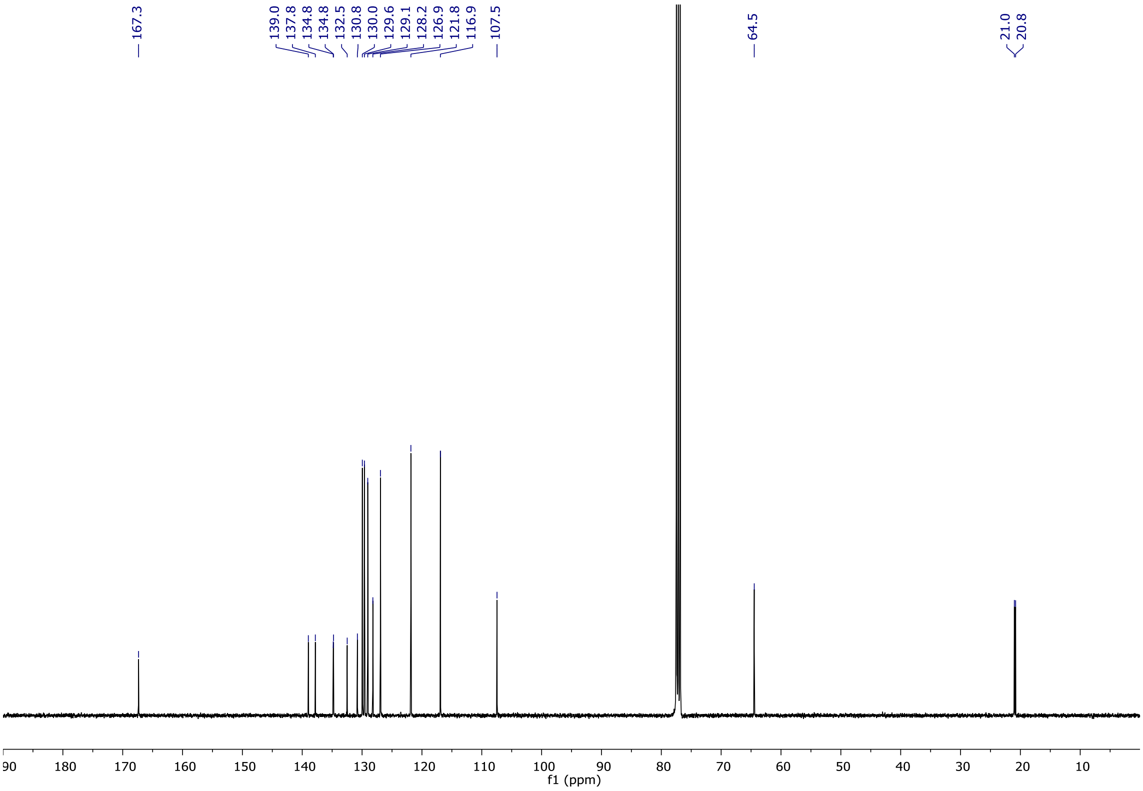

1,5-Diphenyl-3-(phenylamino)-1,5-dihydro-2*H*-pyrrol-2-one (8b)

^{1}H NMR (400 MHz, $CDCl_3$)

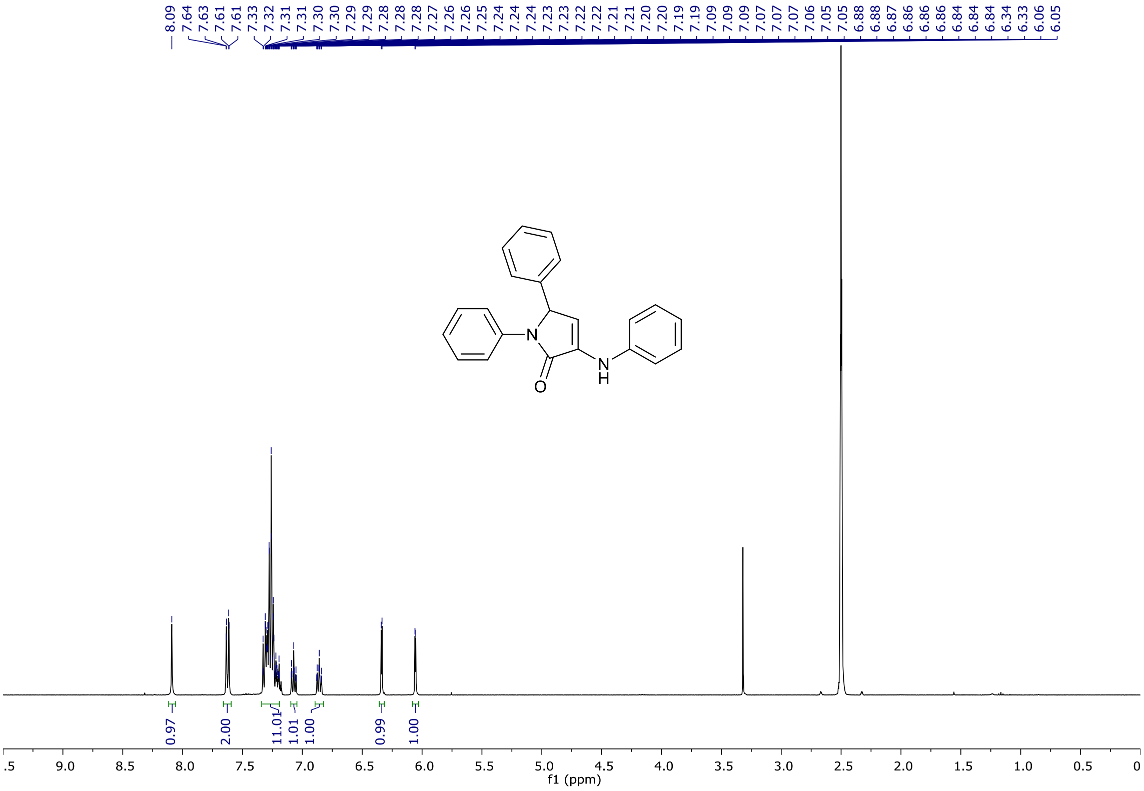

^{13}C {^{1}H} NMR (101 MHz, $CDCl_3$)

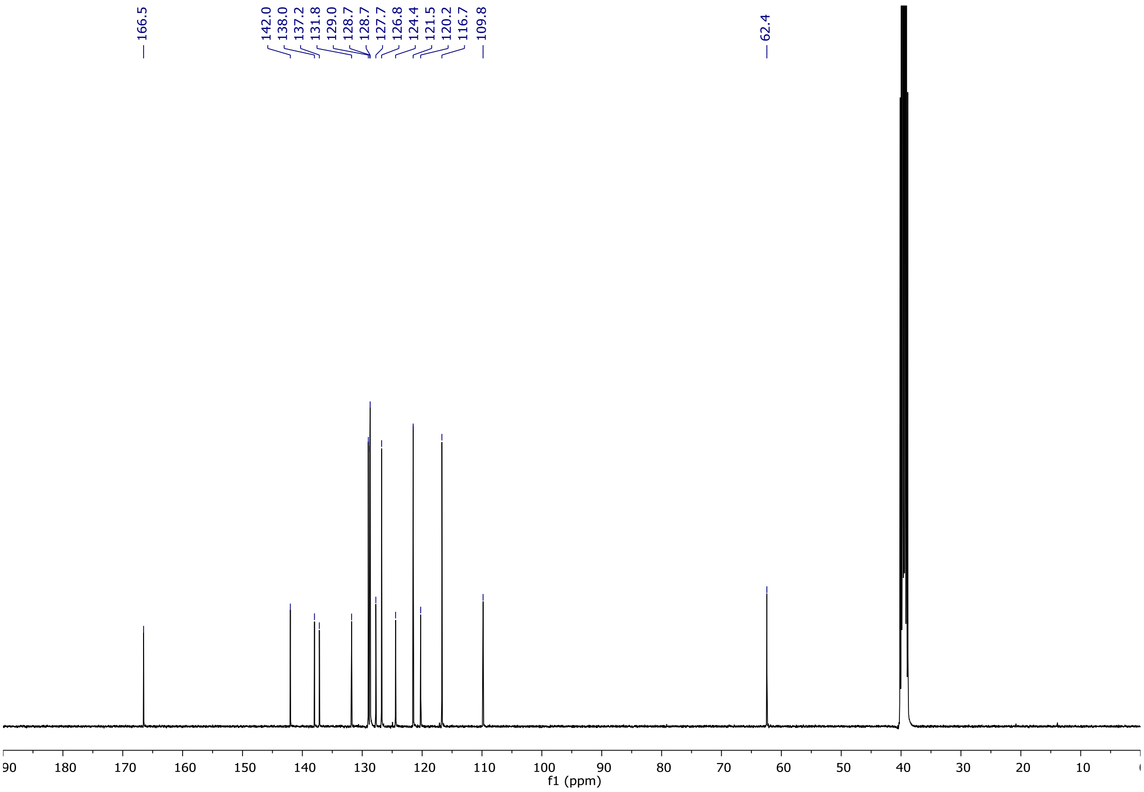

1-(4-Methoxyphenyl)-3-((4-methoxyphenyl)amino)-5-phenyl-1,5-dihydro-2*H*-pyrrol-2-one (8c)

^{1}H NMR (300 MHz, $CDCl_3$)

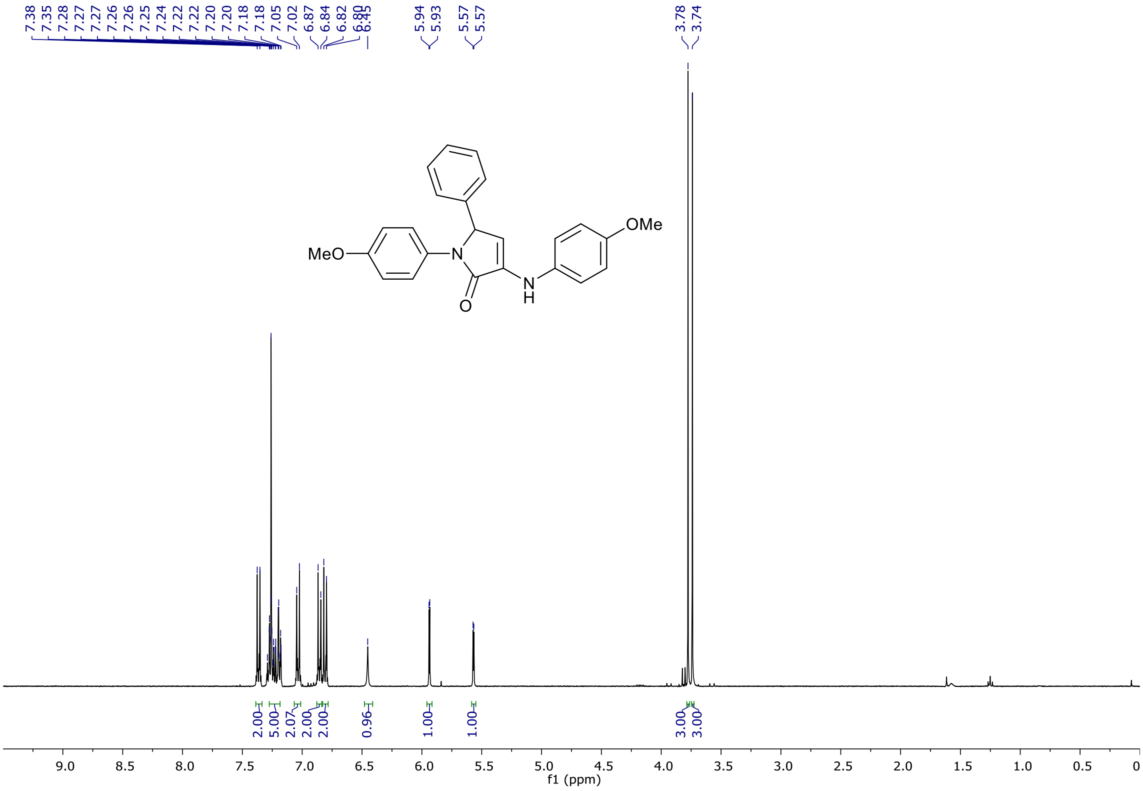

^{13}C {^{1}H} NMR (75 MHz, $CDCl_3$)

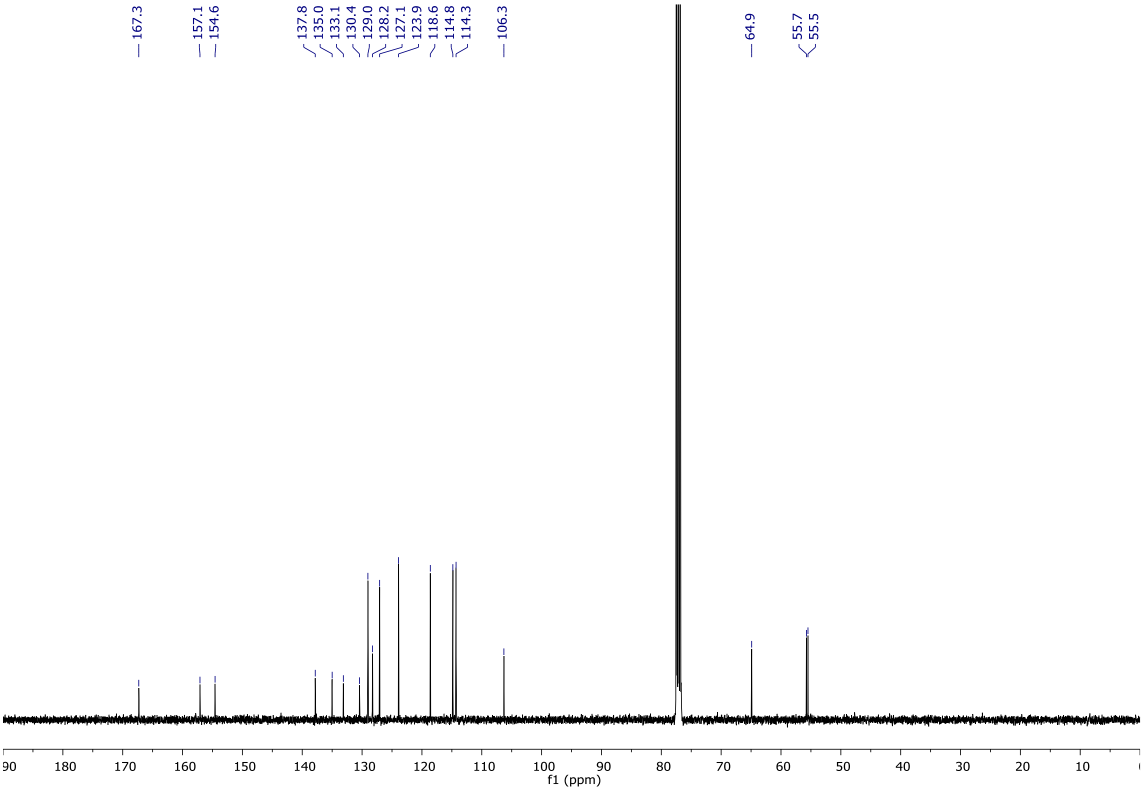

1-(4-Bromophenyl)-3-((4-bromophenyl)amino)-5-phenyl-1,5-dihydro-2*H*-pyrrol-2-one (8d)

^{1}H NMR (400 MHz, $CDCl_3$)

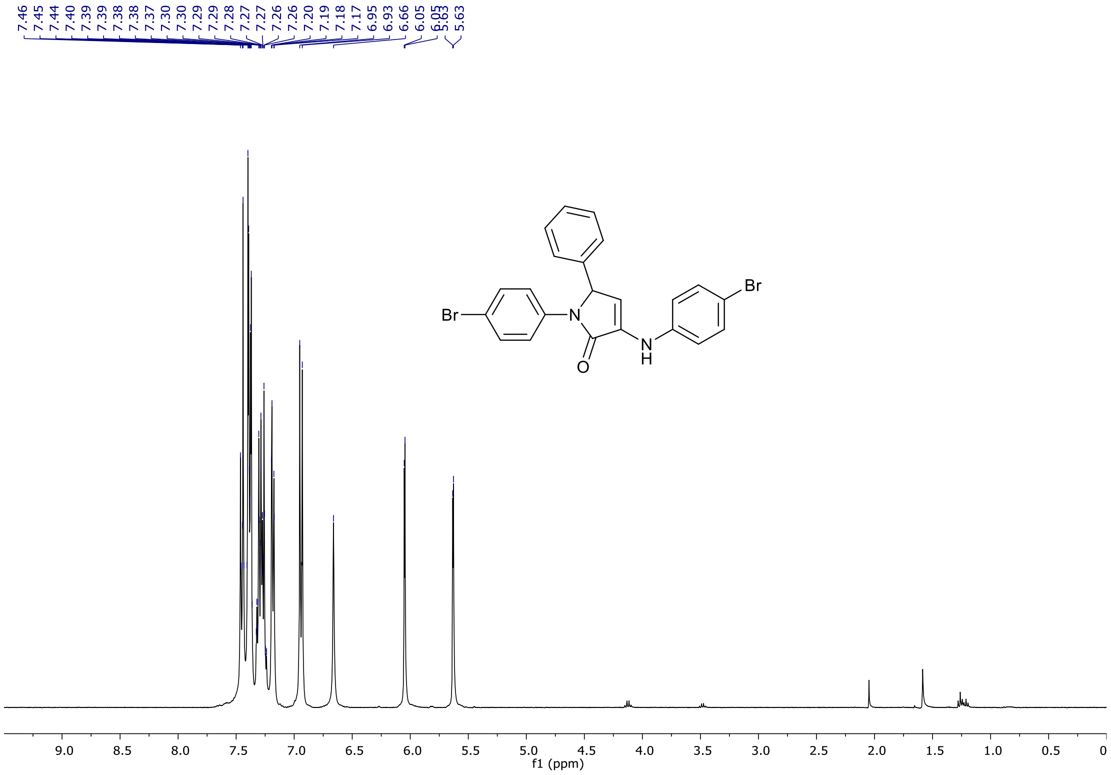

^{13}C {^{1}H} NMR (101 MHz, $CDCl_3$)

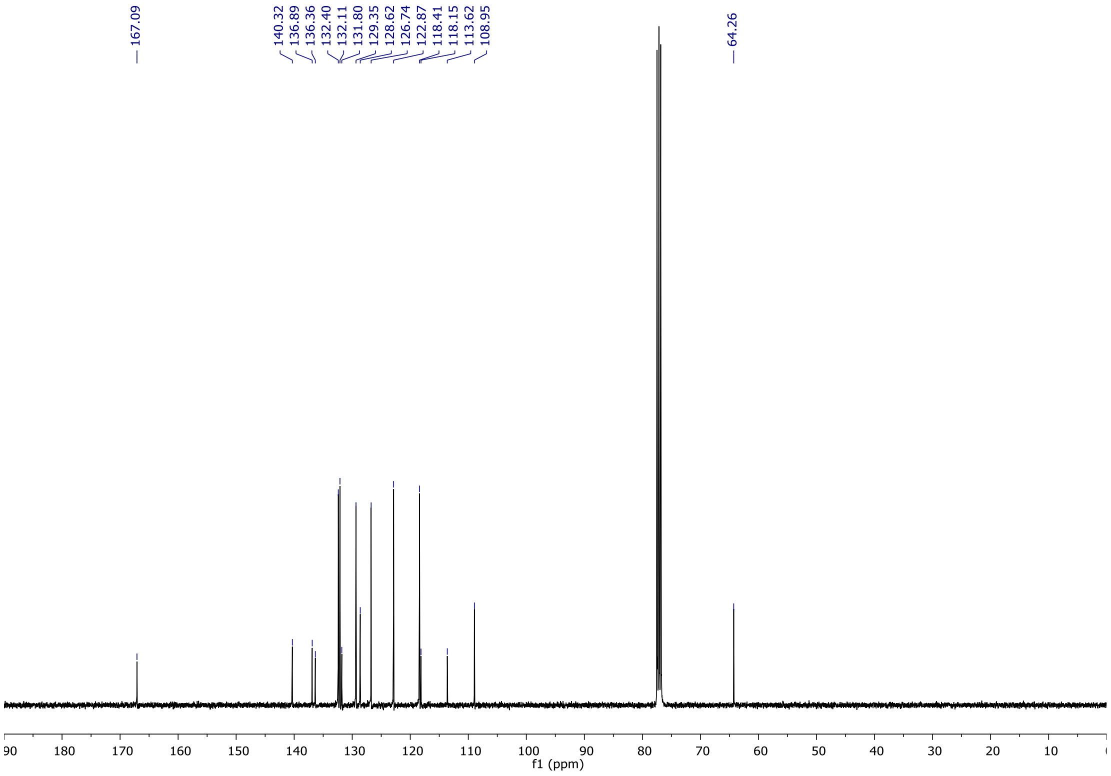

1-(4-Chlorophenyl)-3-((4-chlorophenyl)amino)-5-phenyl-1,5-dihydro-2*H*-pyrrol-2-one (8e)

^{1}H NMR (400 MHz, $CDCl_3$)

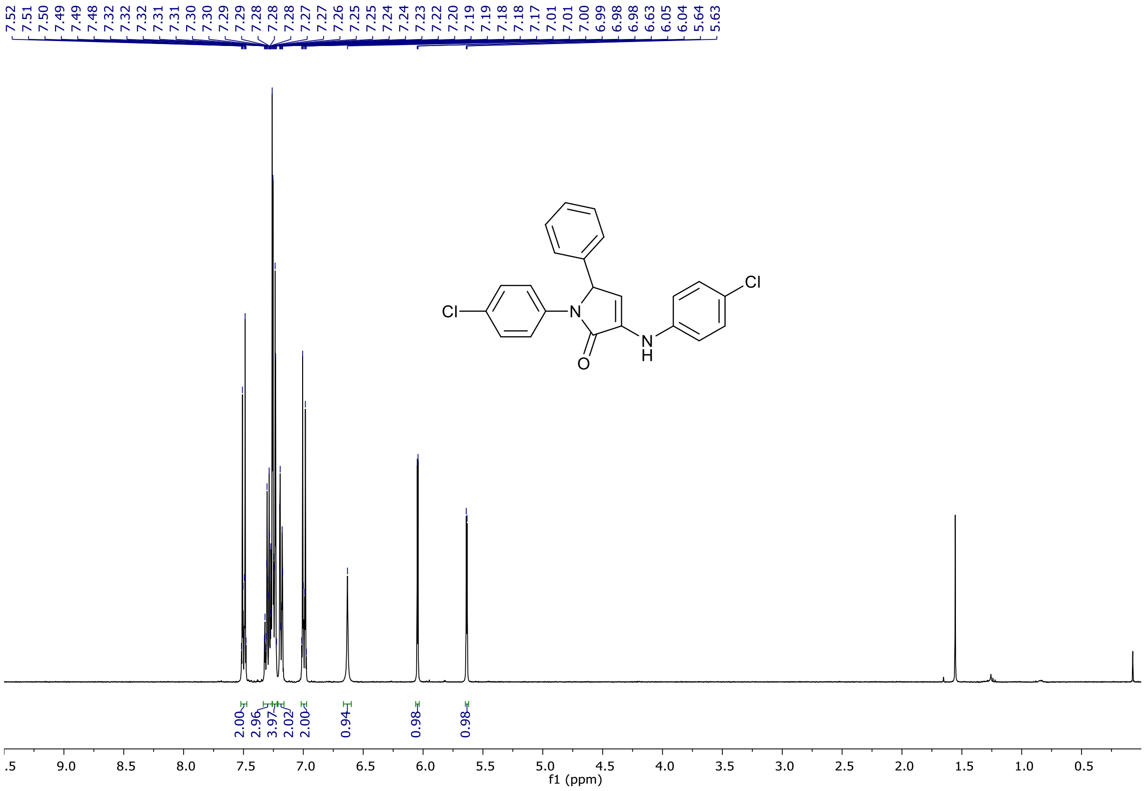

^{13}C {^{1}H} NMR (101 MHz, $CDCl_3$)

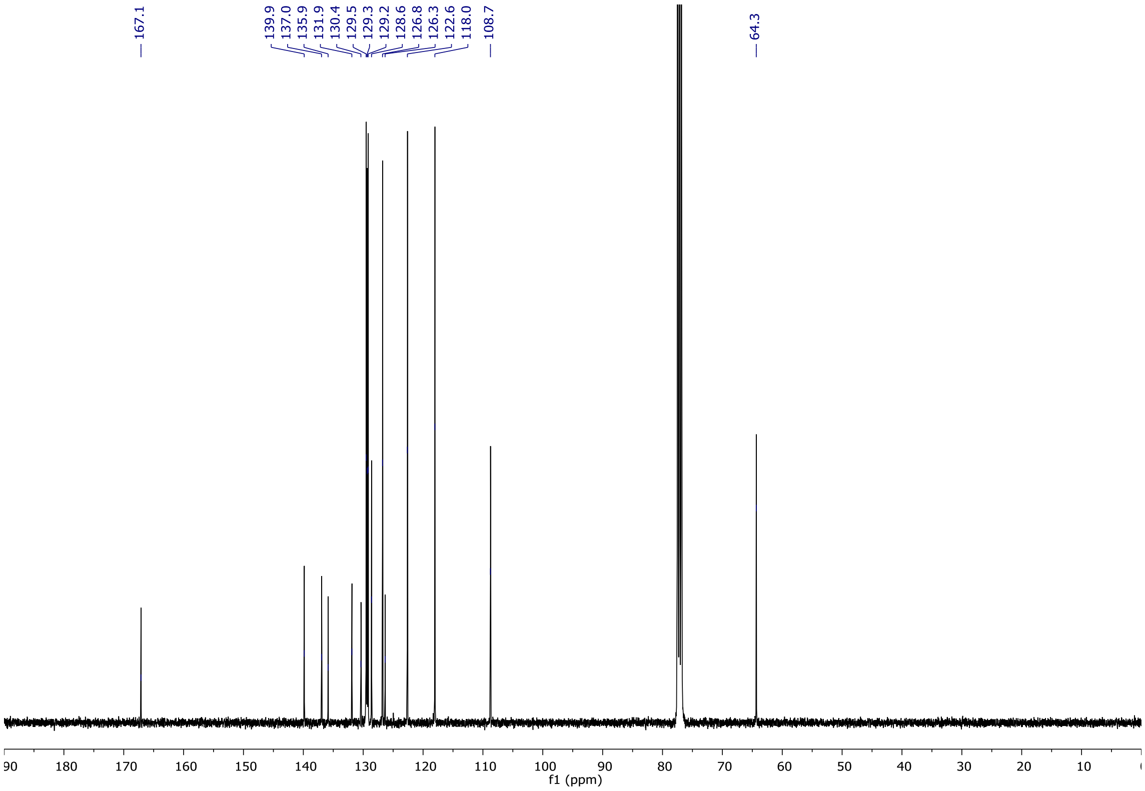

1-(4-Fluorophenyl)-3-((4-fluorophenyl)amino)-5-phenyl-1,5-dihydro-2*H*-pyrrol-2-one (8f)

^{1}H NMR (400 MHz, $CDCl_3$)

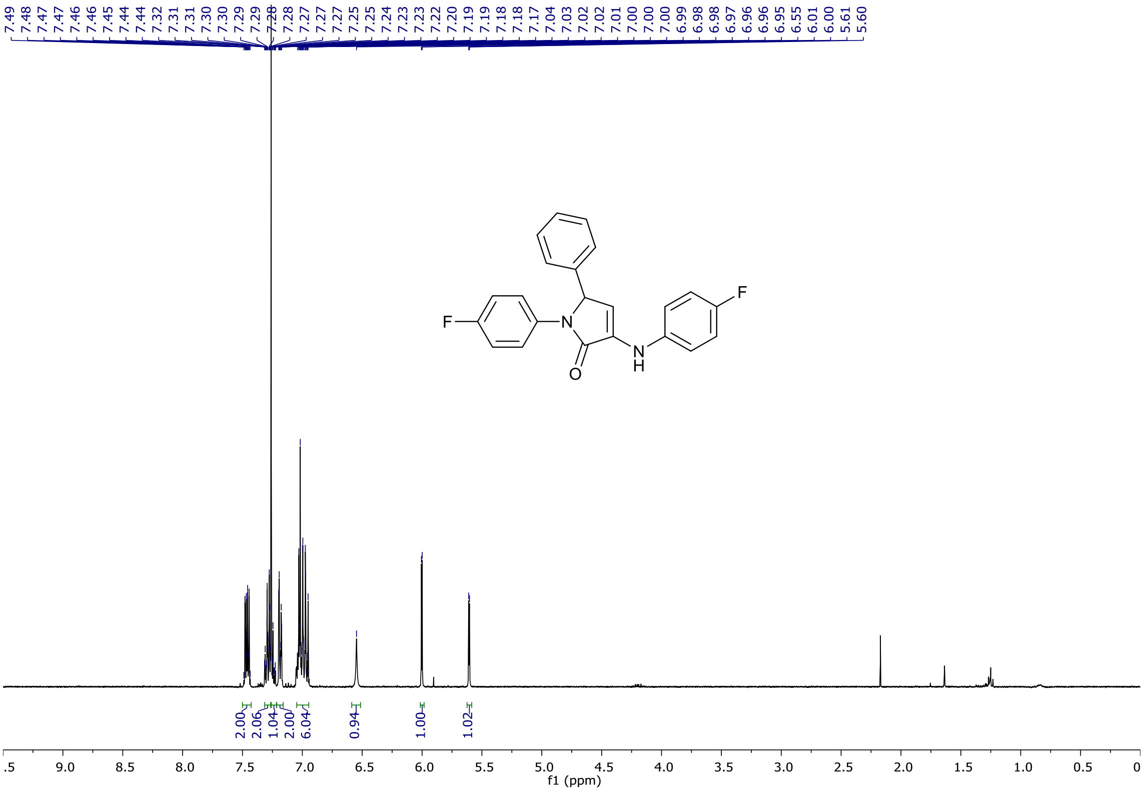

^{13}C {^{1}H} NMR (101 MHz, $CDCl_3$)

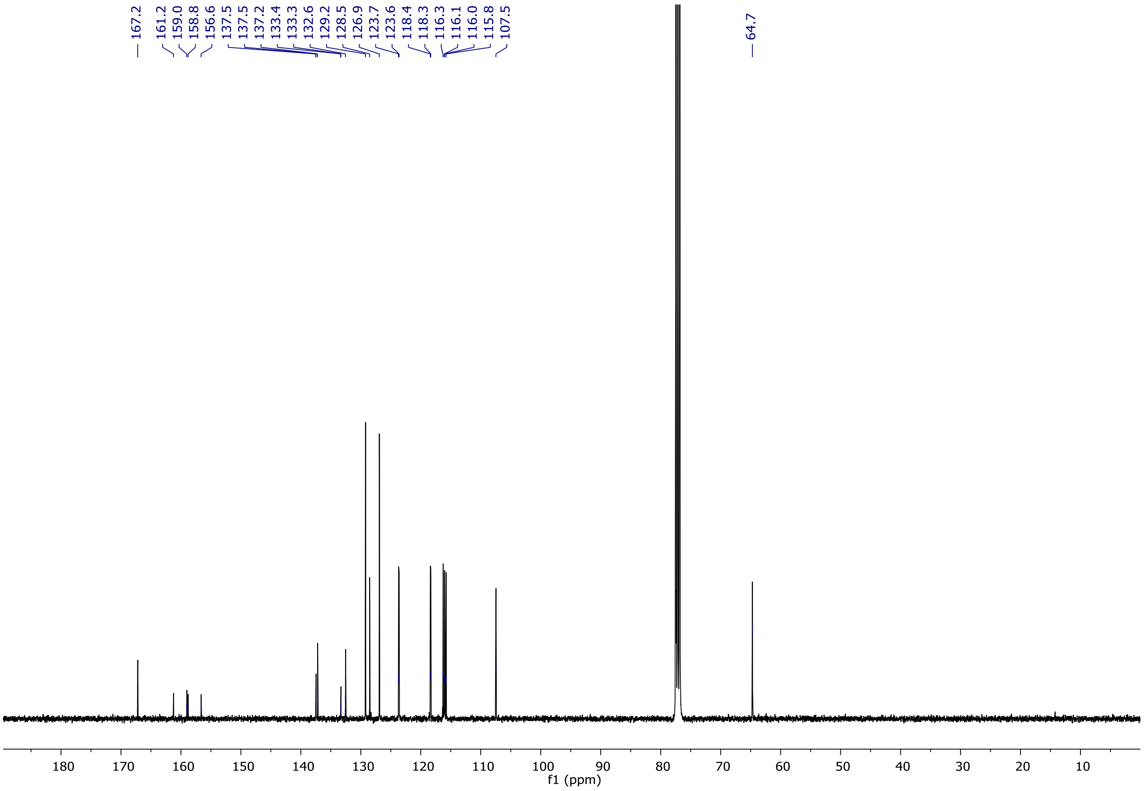

^{19}F {^{1}H} NMR (282 MHz, $CDCl_3$)

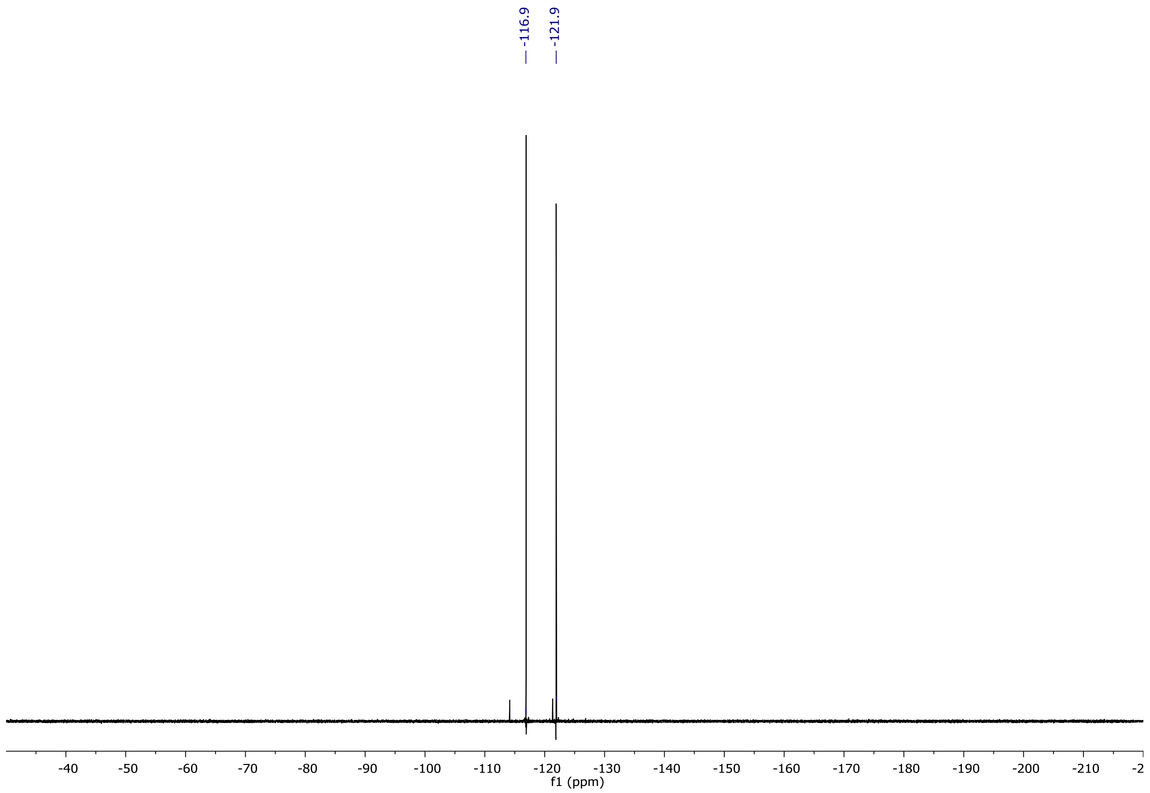

1-(3-Chlorophenyl)-3-((3-chlorophenyl)amino)-5-phenyl-1,5-dihydro-2*H*-pyrrol-2-one (8g)

^{1}H NMR (400 MHz, $CDCl_3$)

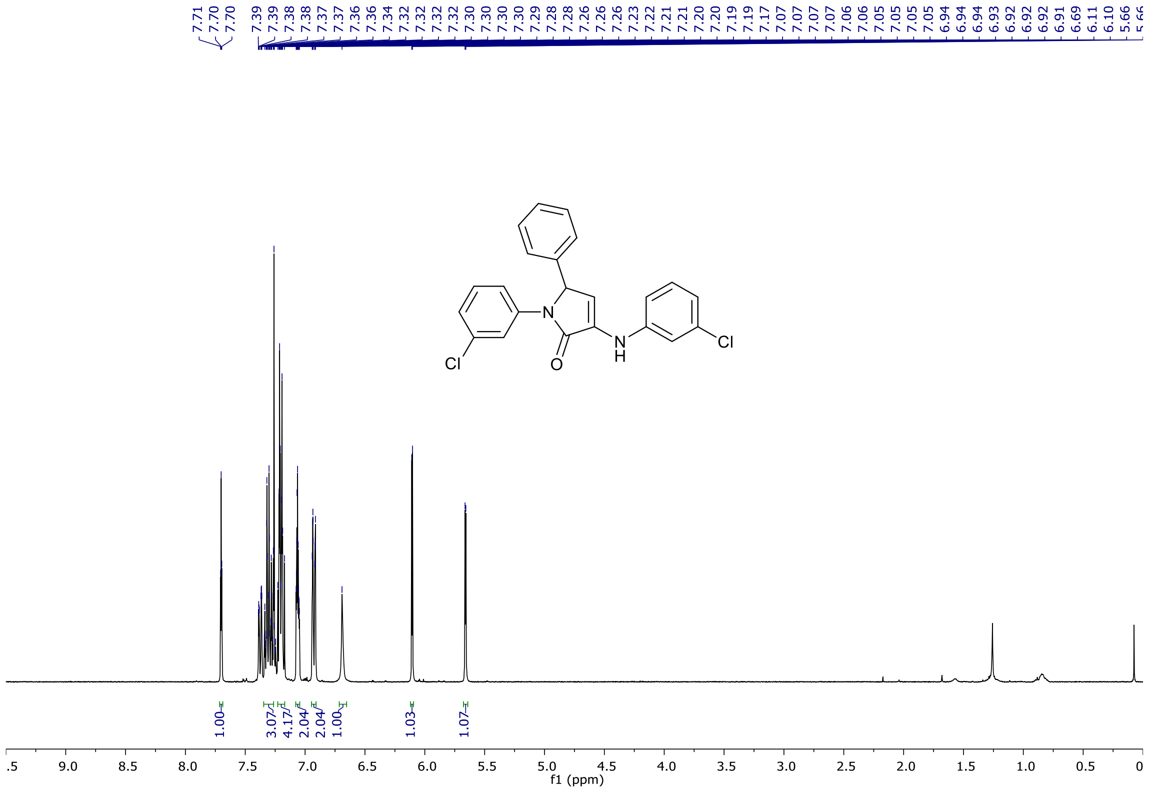

^{13}C {^{1}H} NMR (101 MHz, $CDCl_3$)

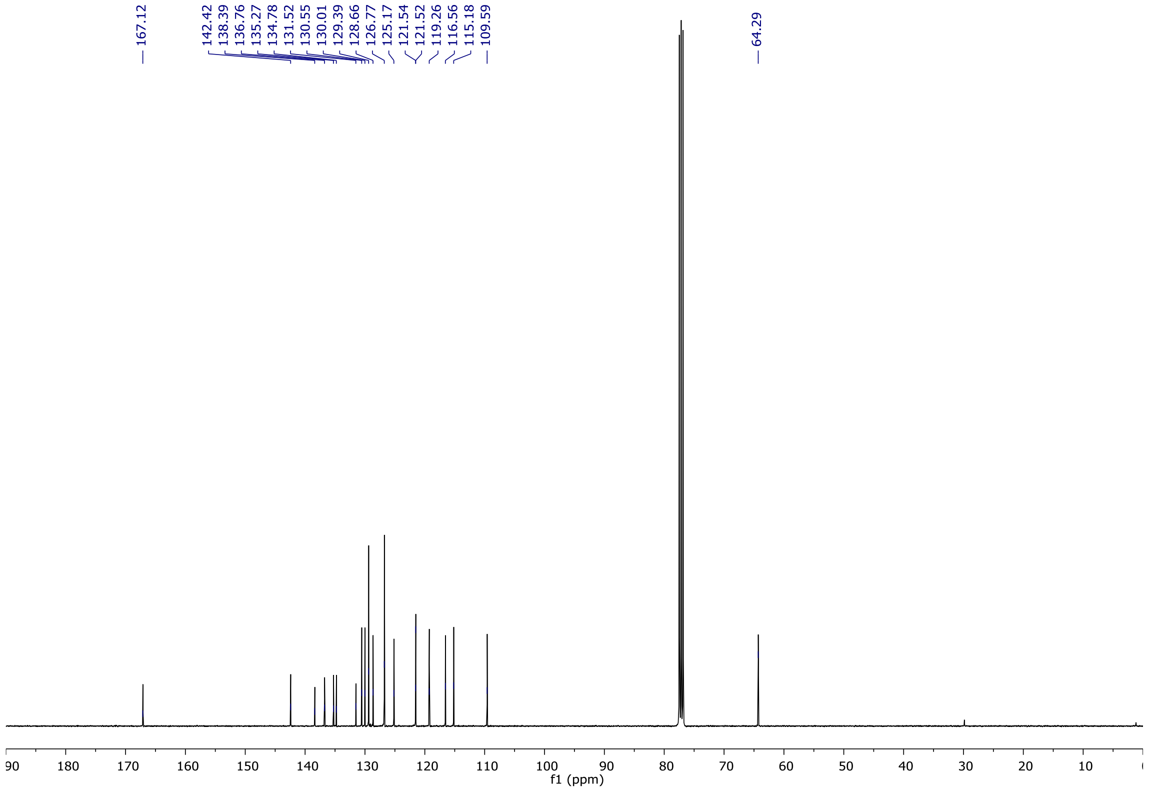

1-(2-Fluorophenyl)-3-((2-fluorophenyl)amino)-5-phenyl-1,5-dihydro-2*H*-pyrrol-2-one (8h)

^{1}H NMR (400 MHz, $CDCl_3$)

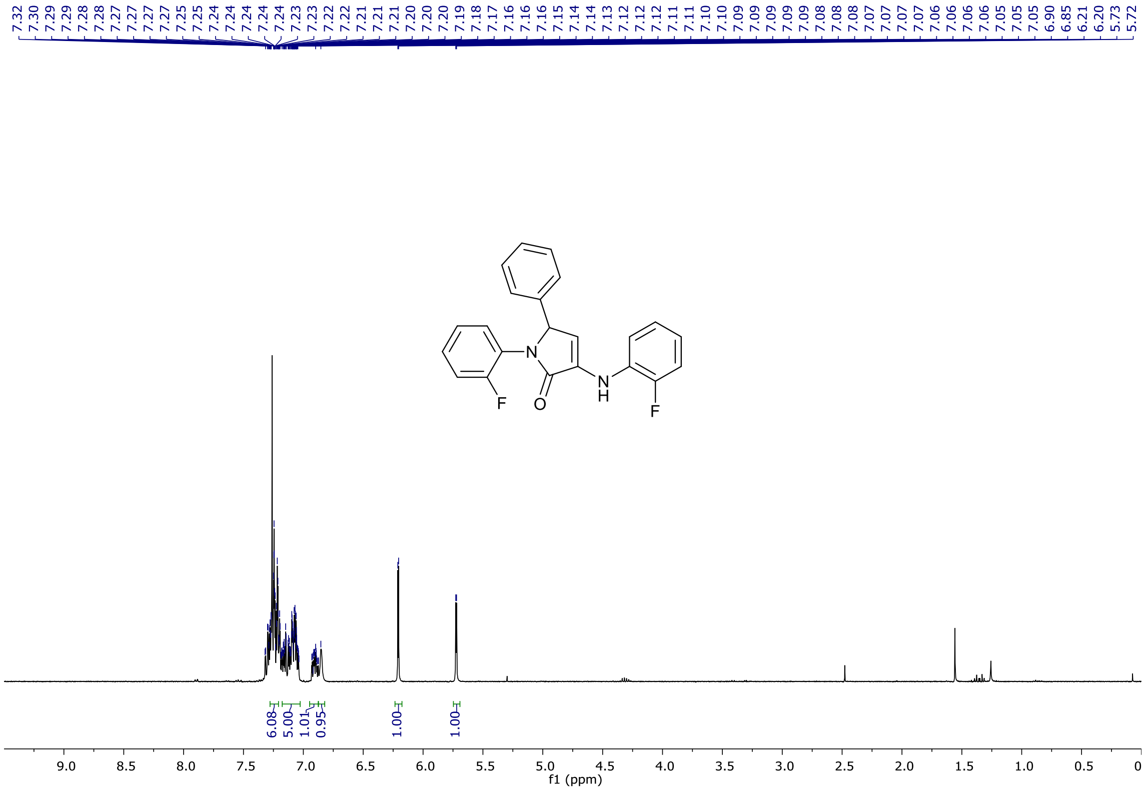

^{13}C {^{1}H} NMR (101 MHz, $CDCl_3$)

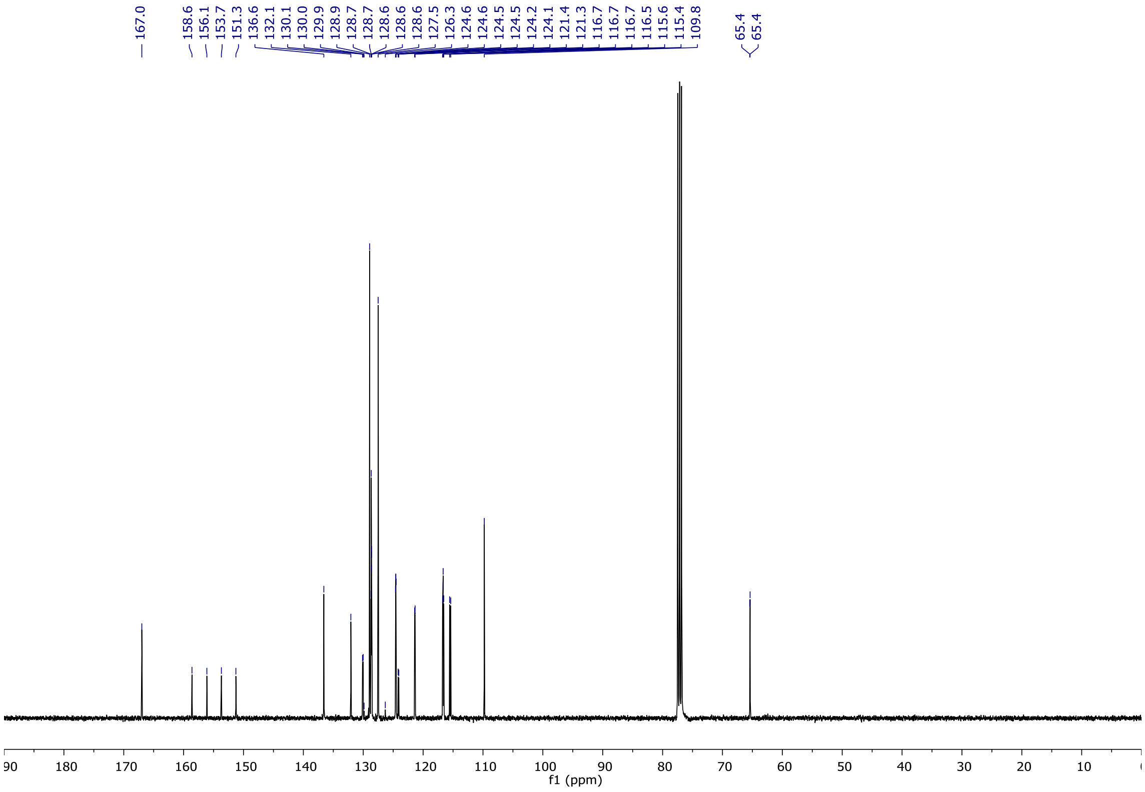

^{19}F {^{1}H} NMR (282 MHz, $CDCl_3$)

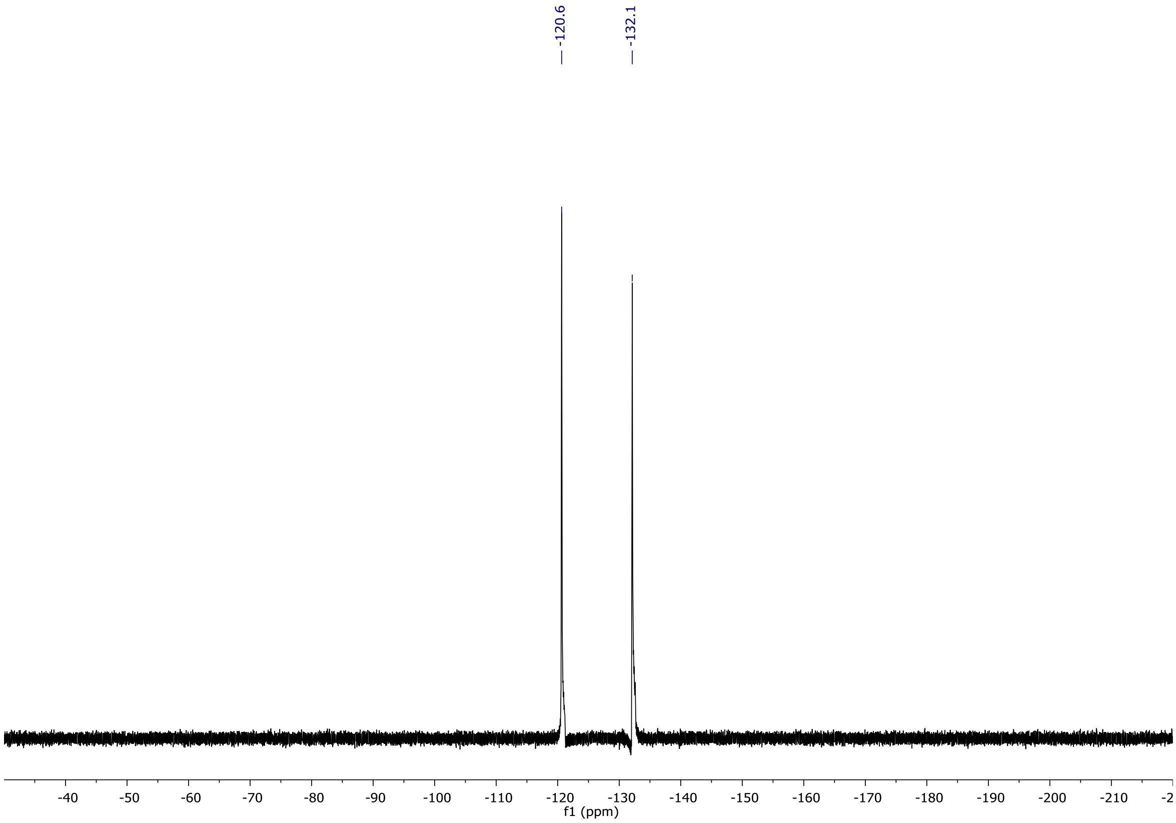

5-Phenyl-1-(3-(trifluoromethyl)phenyl)-3-((3-(trifluoromethyl)phenyl)amino)-1,5-dihydro-2*H*-pyrrol-2-one (8i)

^{1}H NMR (400 MHz, $CDCl_3$)

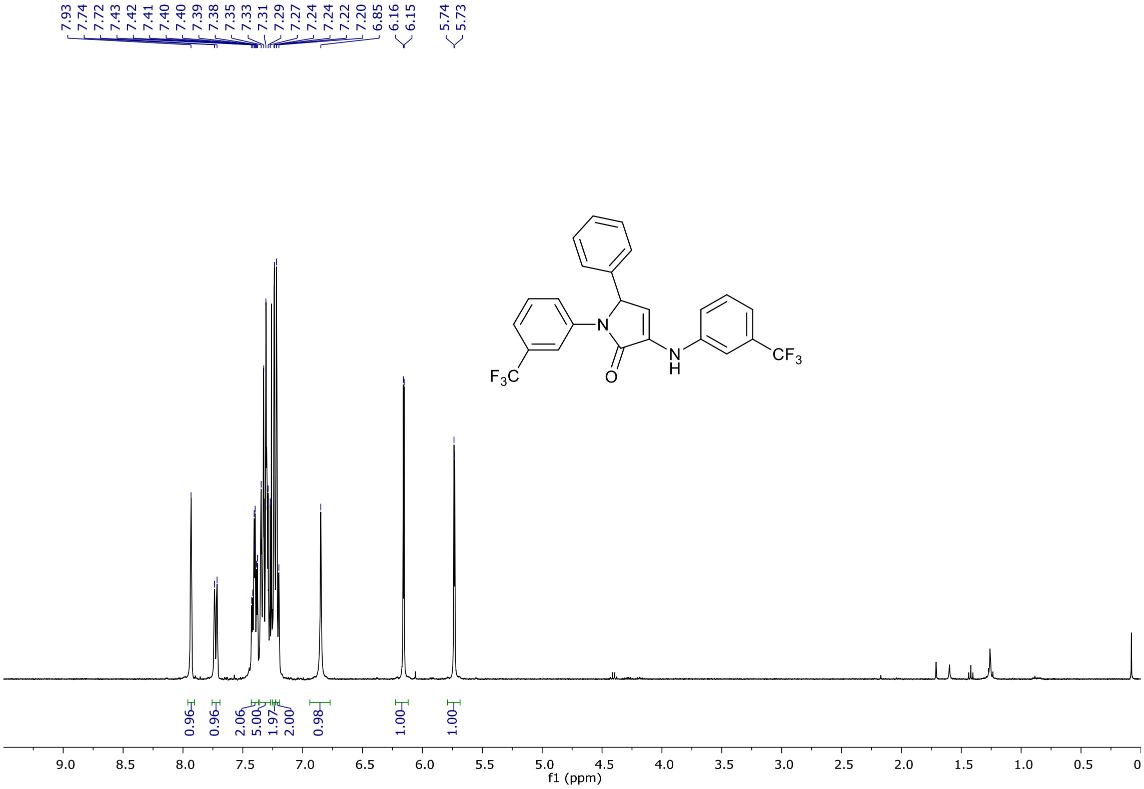

^{13}C {^{1}H} NMR (101 MHz, $CDCl_3$)

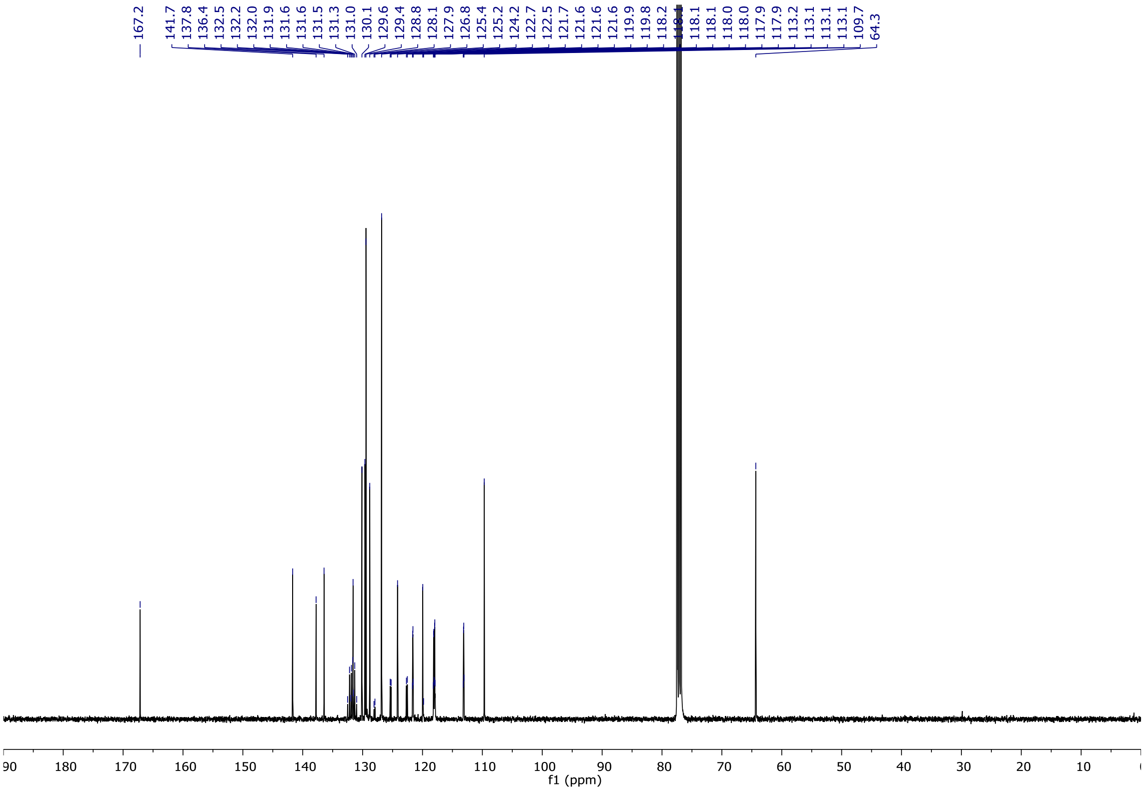

^{19}F {^{1}H} NMR (282 MHz, $CDCl_3$)

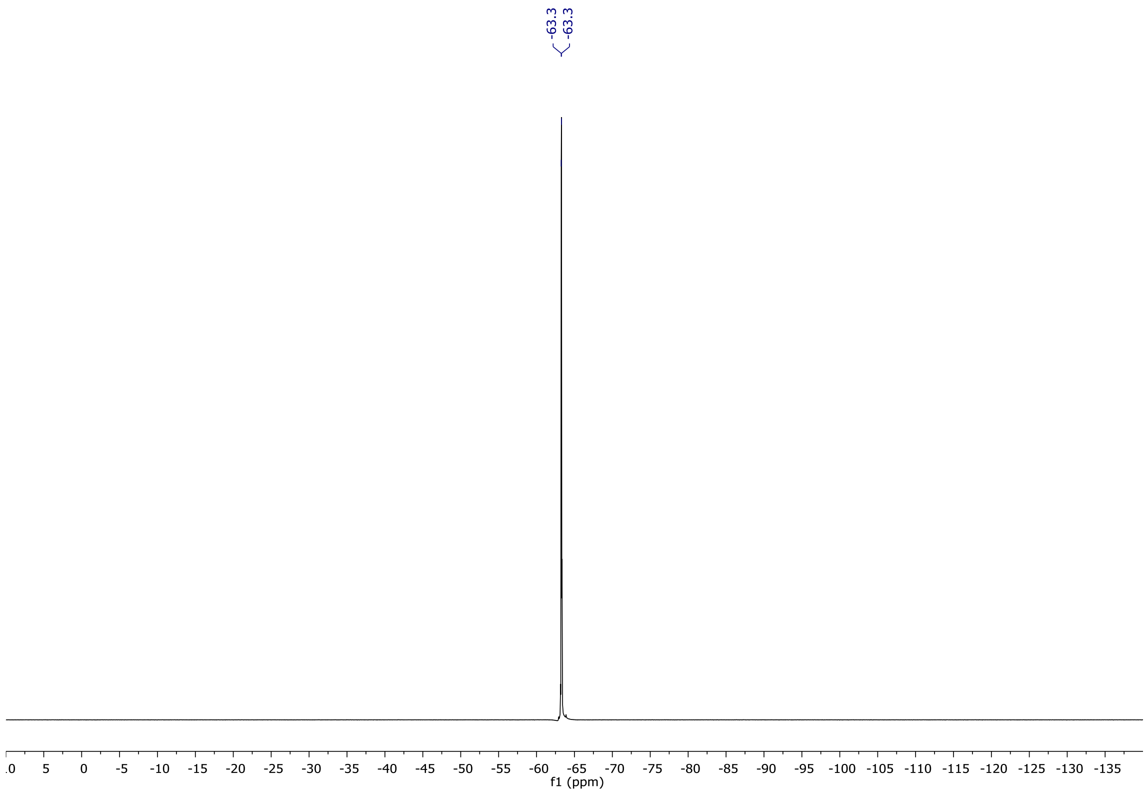

4-((Dimethylamino)methyl)-1-(*p*-tolyl)-3-(*p*-tolylamino)-1,5-dihydro-2*H*-pyrrol-2-one (2)

^{1}H NMR (400 MHz, $CDCl_3$)

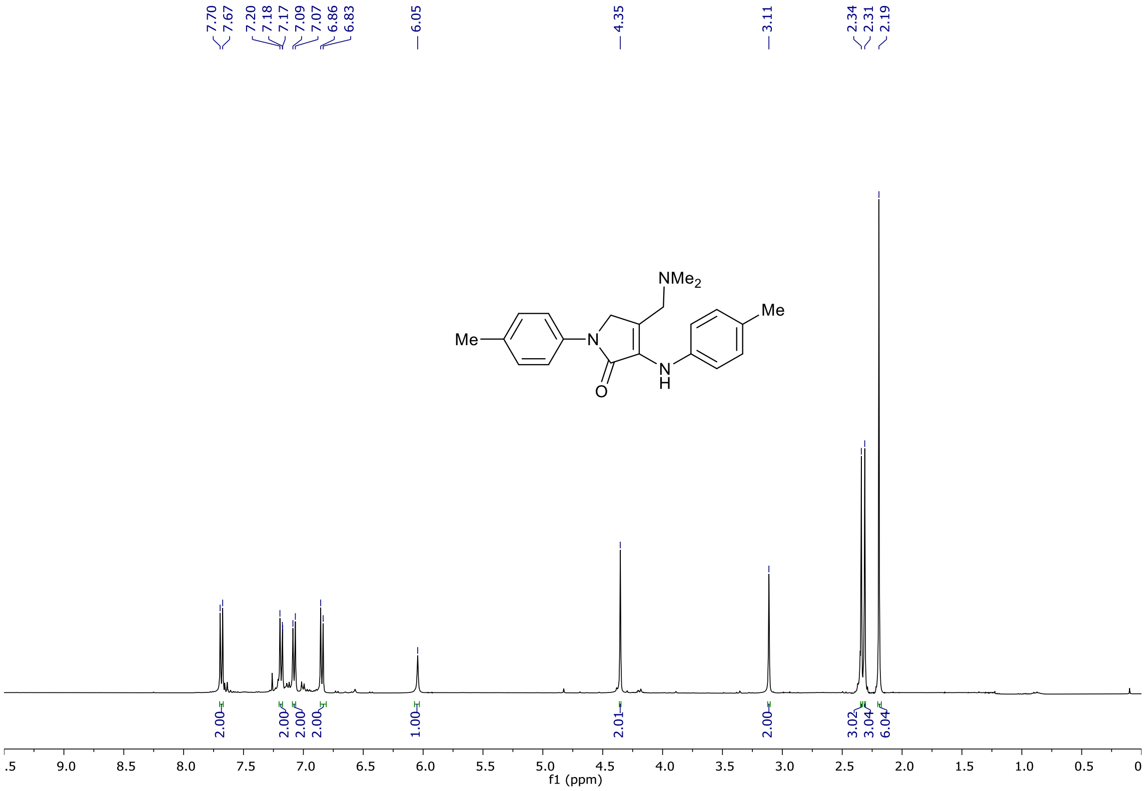

^{13}C {^{1}H} NMR (101 MHz, $CDCl_3$)

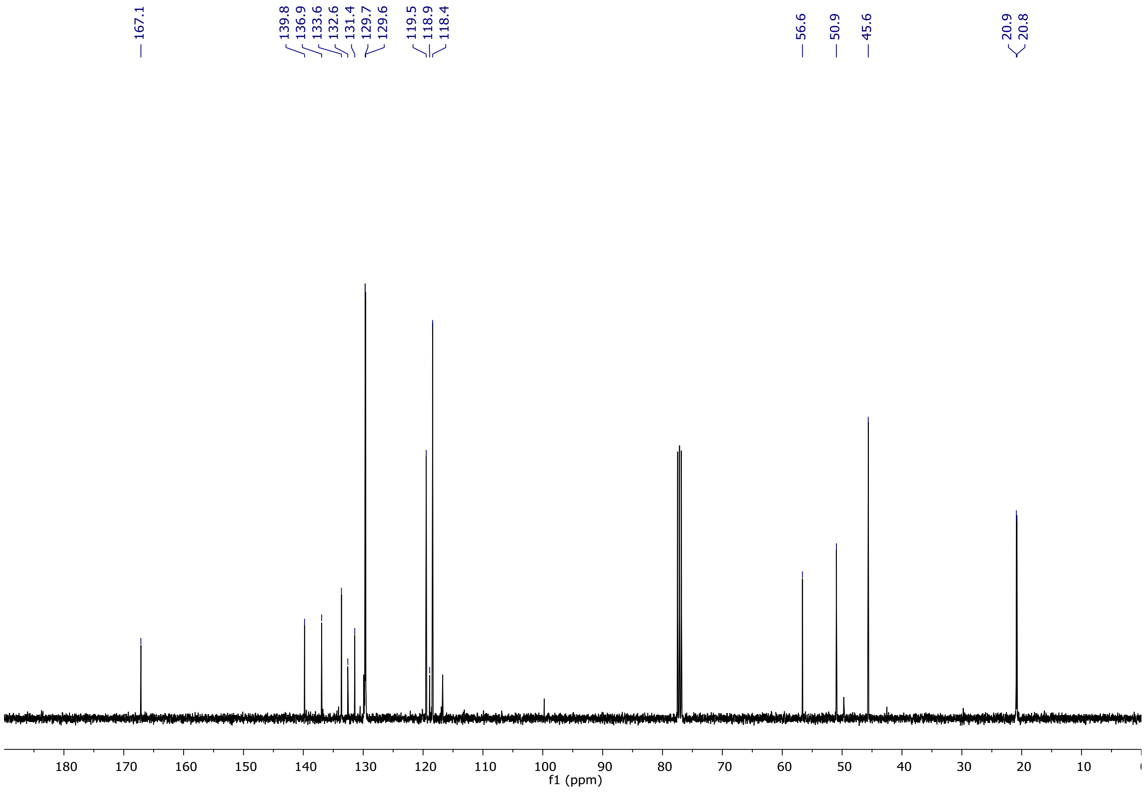

4-((Dimethylamino)methyl)-5-phenyl-1-(*p*-tolyl)-3-(*p*-tolylamino)-1,5-dihydro-2*H*-pyrrol-2-one (9a)

^{1}H NMR (400 MHz, $CDCl_3$)

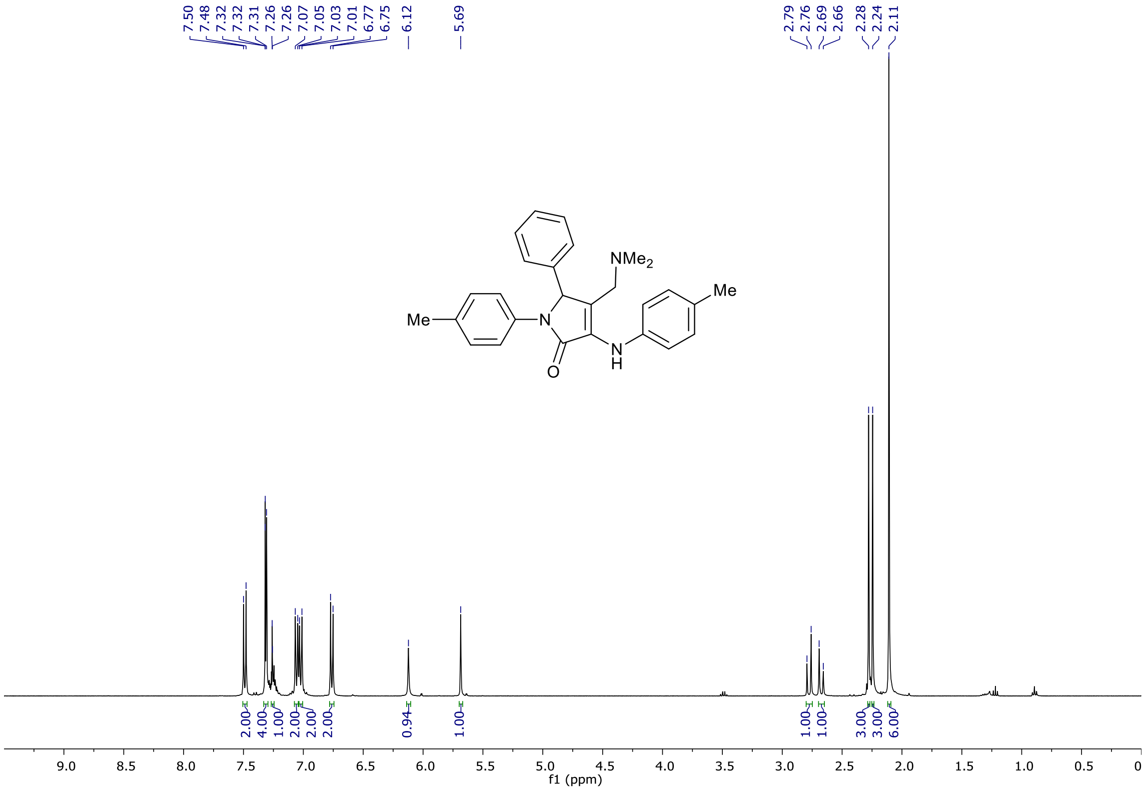

^{13}C {^{1}H} NMR (101 MHz, $CDCl_3$)

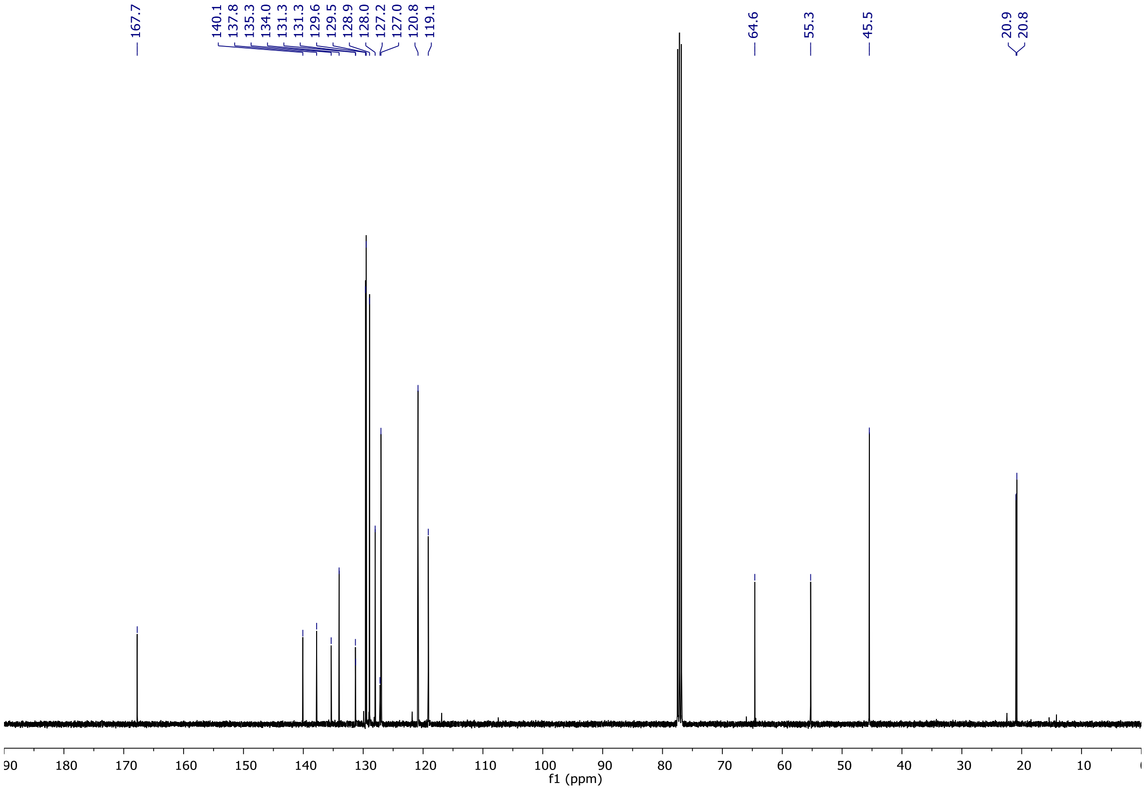

4-((Dimethylamino)methyl)-1,5-diphenyl-3-(phenylamino)-1,5-dihydro-2*H*-pyrrol-2-one (9b)

^{1}H NMR (400 MHz, $CDCl_3$)

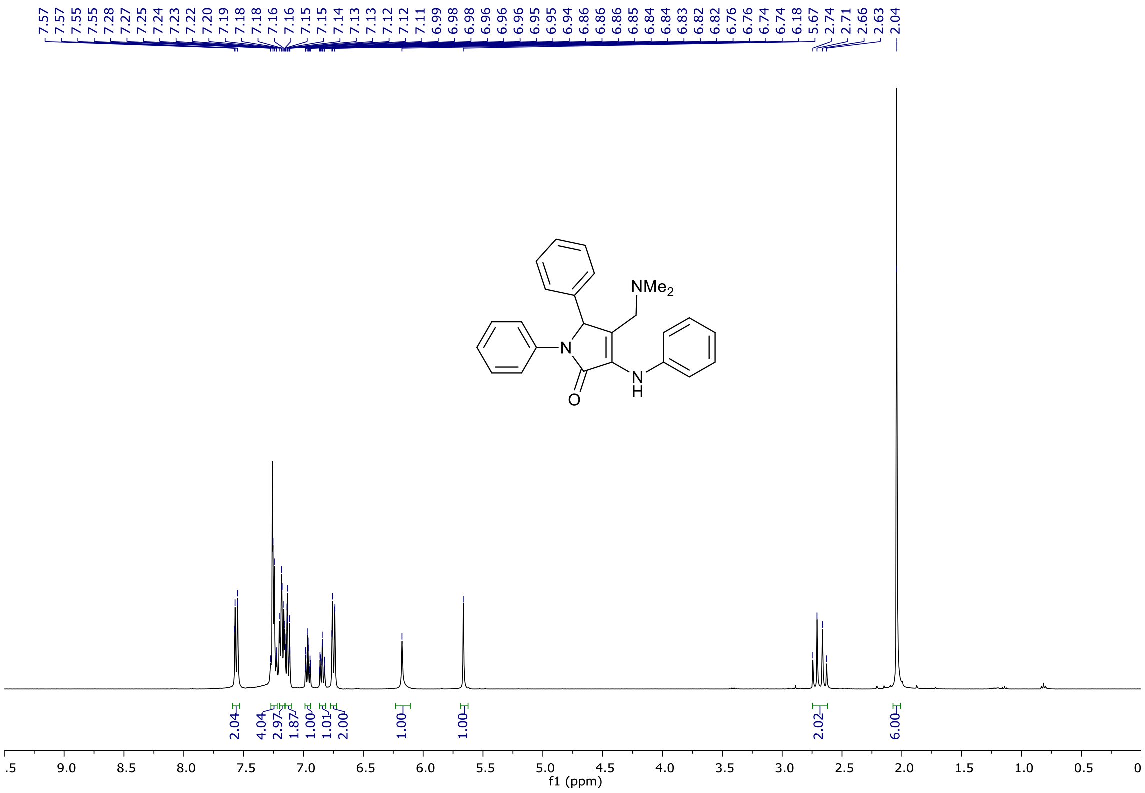

^{13}C {^{1}H} NMR (101 MHz, $CDCl_3$)

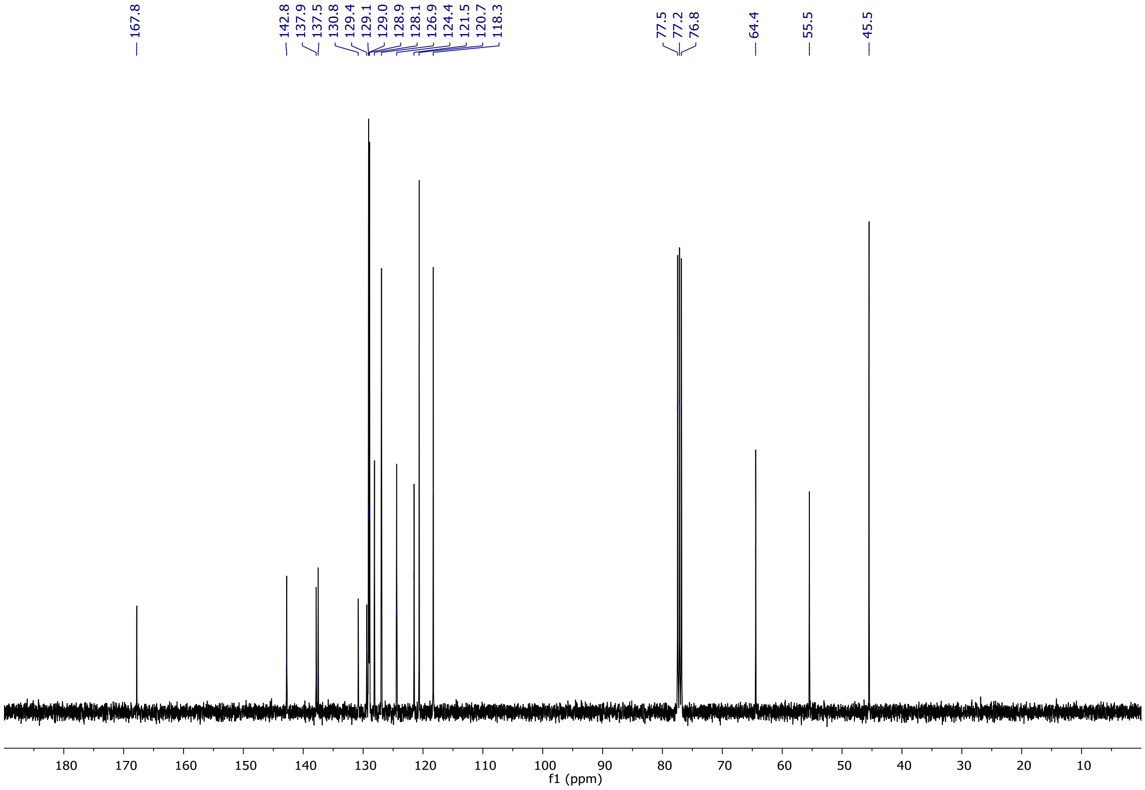

4-((Dimethylamino)methyl)-1-(4-methoxyphenyl)-3-((4-methoxyphenyl)amino)-5-phenyl-1,5-dihydro-2*H*-pyrrol-2-one (9c)

^{1}H NMR (400 MHz, $CDCl_3$)

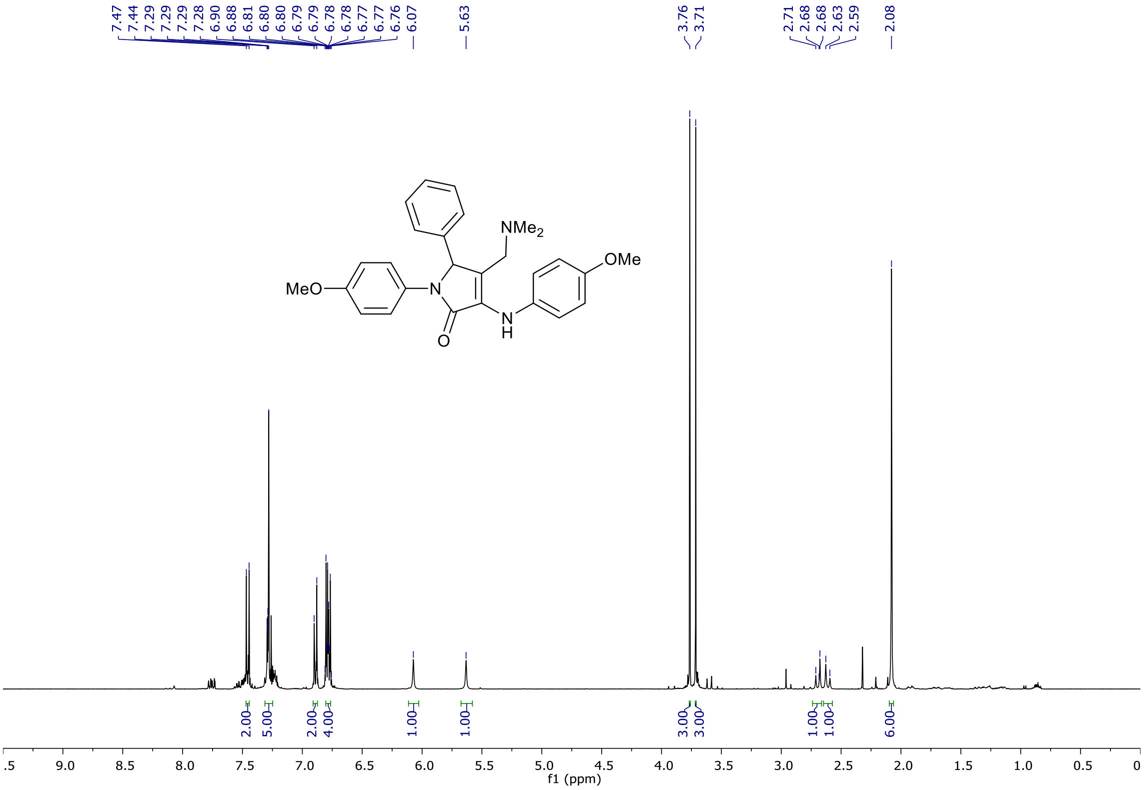

^{13}C {^{1}H} NMR (101 MHz, $CDCl_3$)

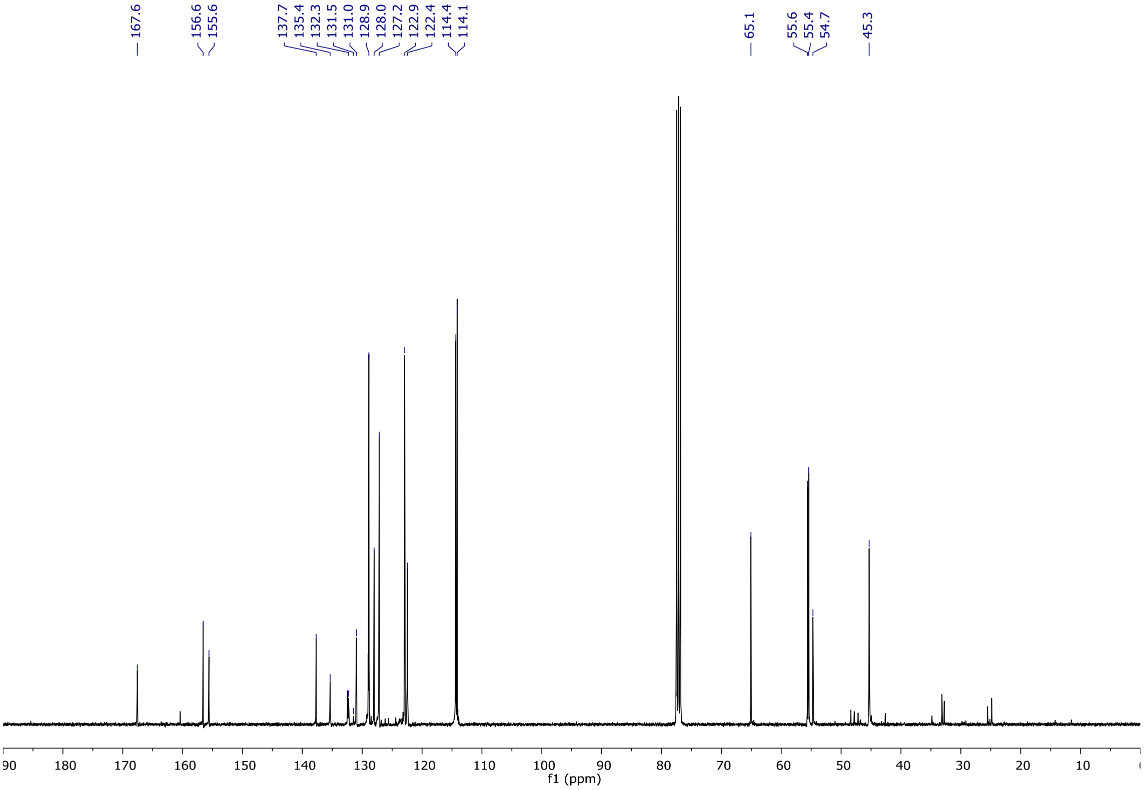

1-(4-Bromophenyl)-3-((4-bromophenyl)amino)-4-((dimethylamino)methyl)-5-phenyl-1,5-dihydro-2*H*-pyrrol-2-one (9d)

^{1}H NMR (400 MHz, $CDCl_3$)

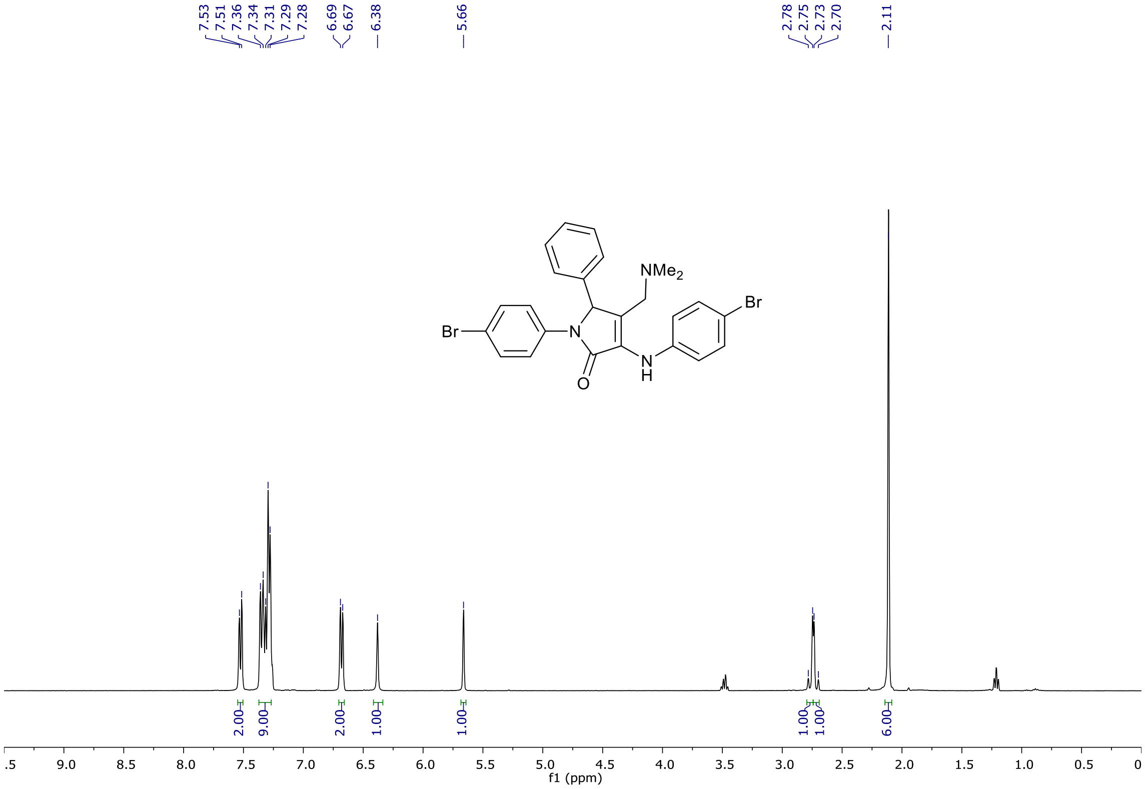

^{13}C {^{1}H} NMR (101 MHz, $CDCl_3$)

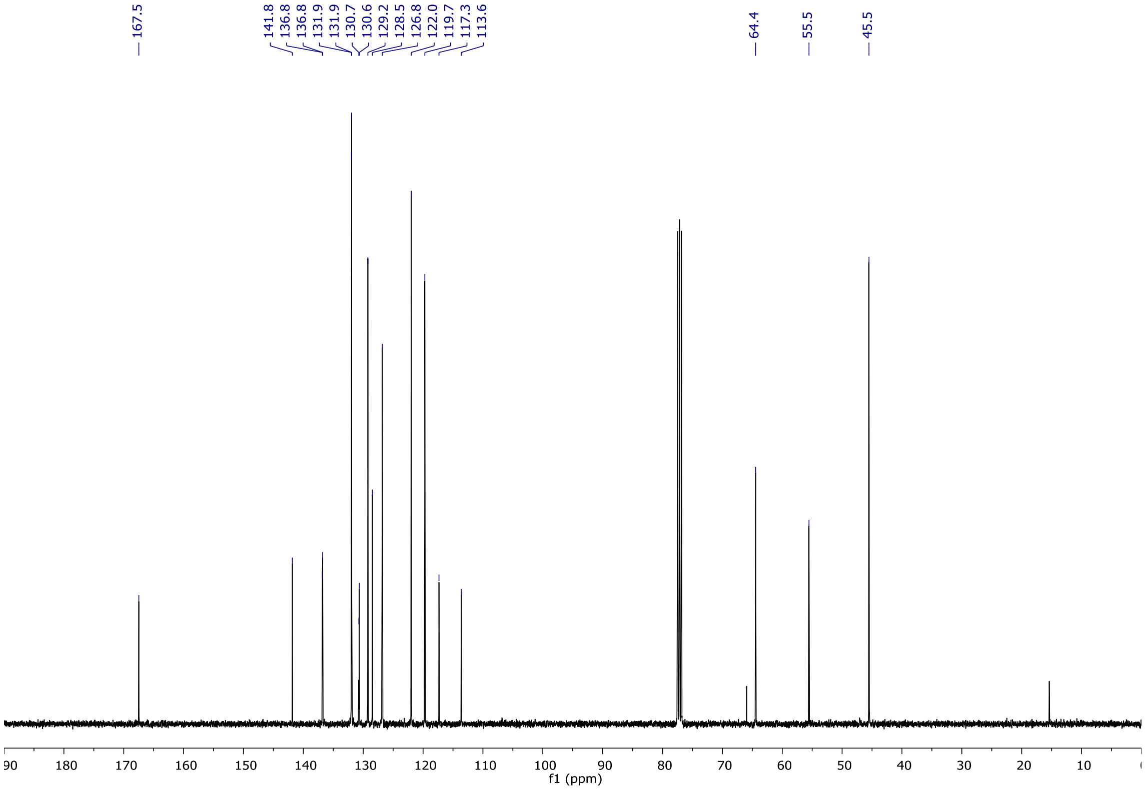

1-(4-Chlorophenyl)-3-((4-chlorophenyl)amino)-4-((dimethylamino)methyl)-5-phenyl-1,5-dihydro-2*H*-pyrrol-2-one (9e)

^{1}H NMR (400 MHz, $CDCl_3$)

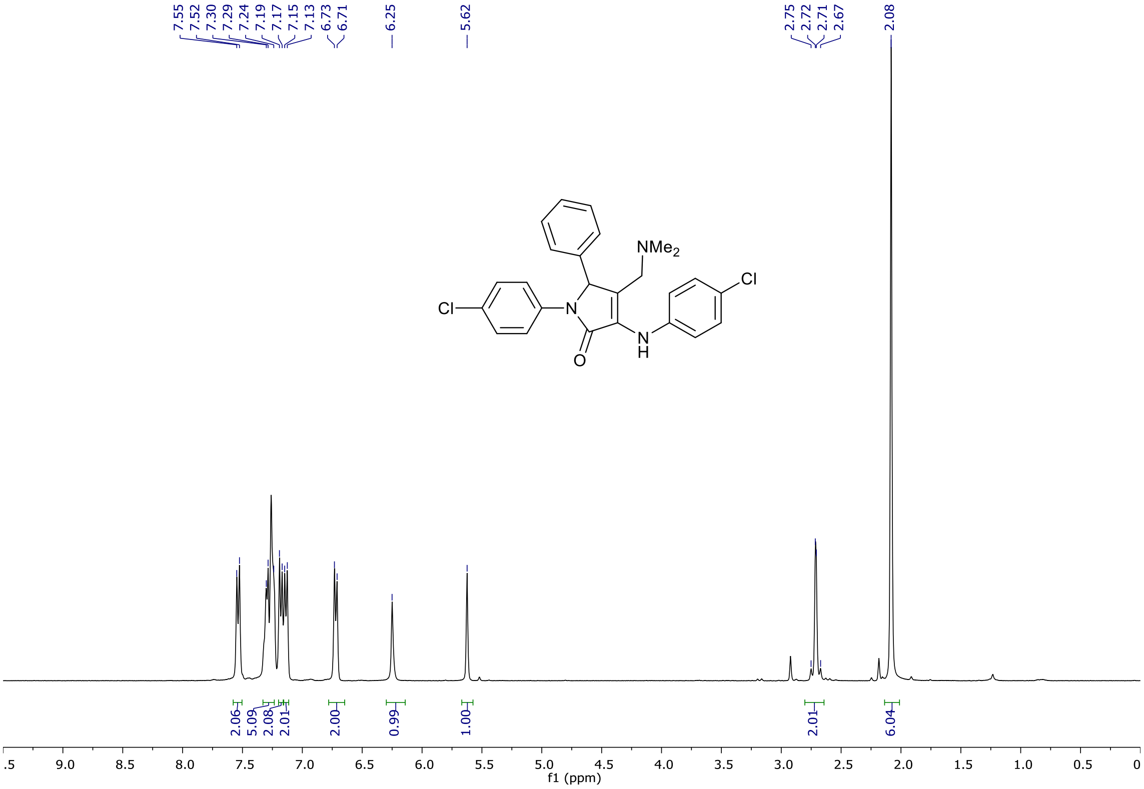

^{13}C NMR{^{1}H} (101 MHz, $CDCl_3$)

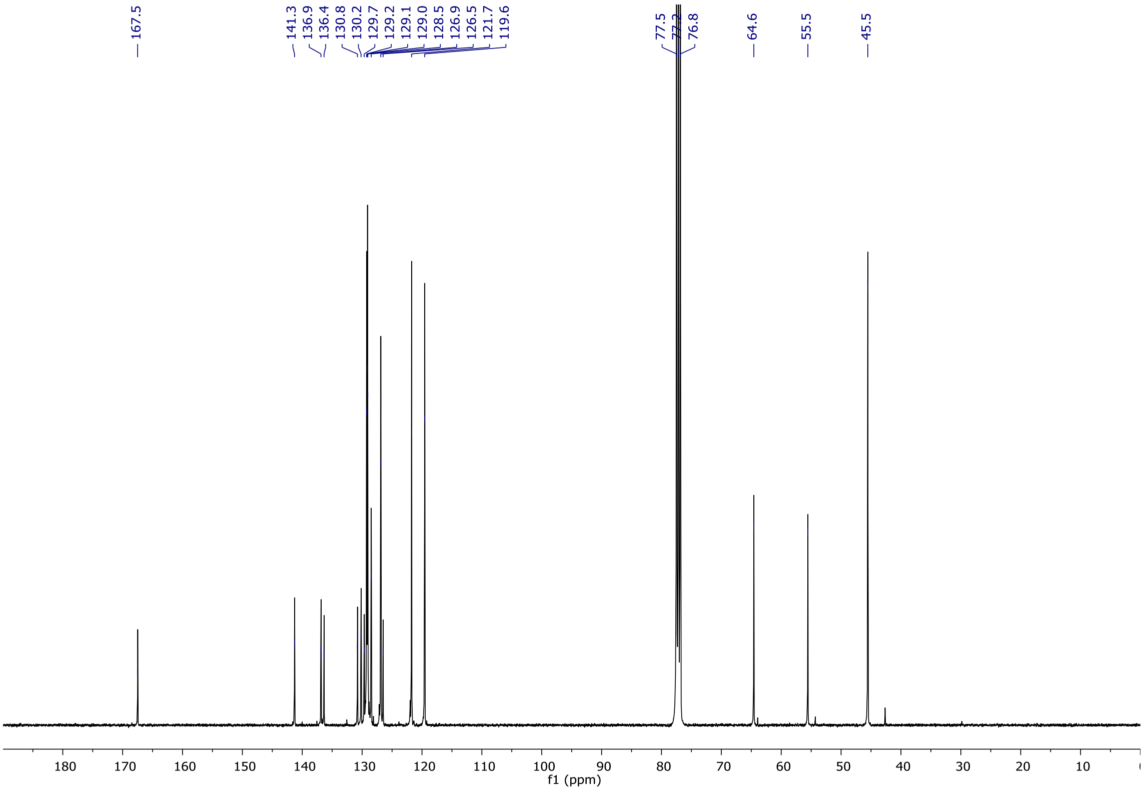

4-((Dimethylamino)methyl)-1-(4-fluorophenyl)-3-((4-fluorophenyl)amino)-5-phenyl-1,5-dihydro-2*H*-pyrrol-2-one (9f)

^{1}H NMR (400 MHz, $CDCl_3$)

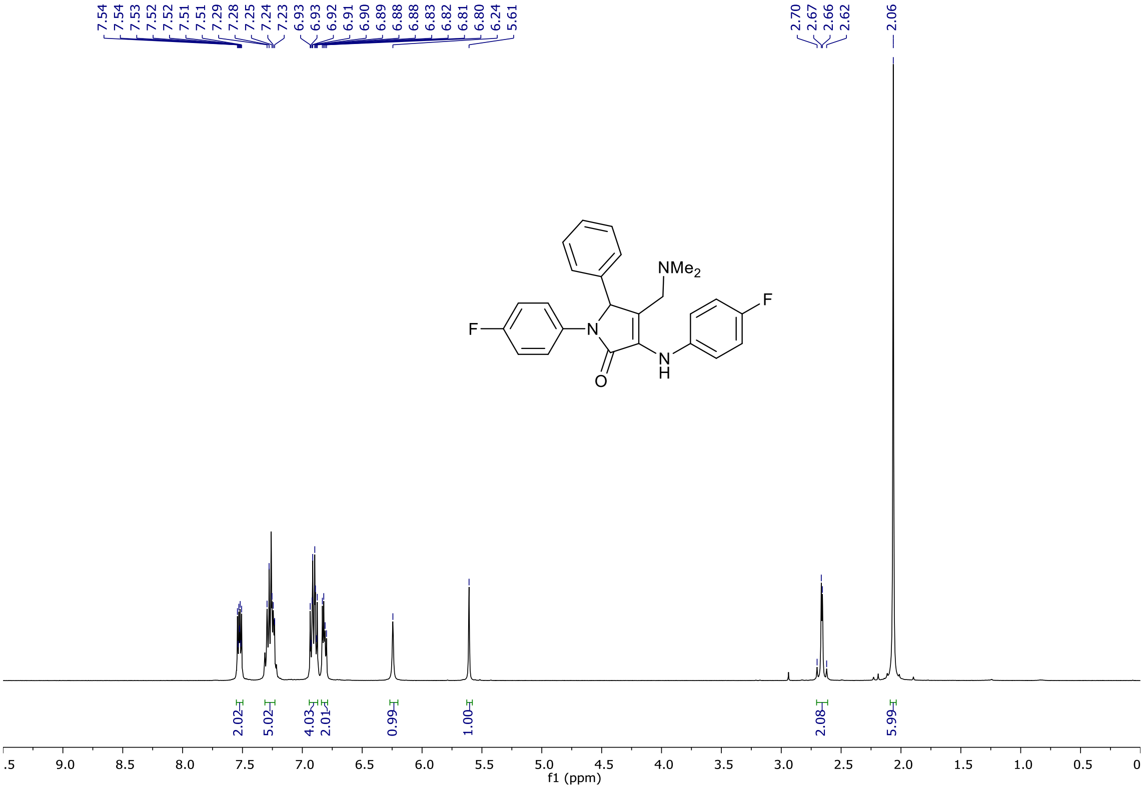

^{13}C {^{1}H} NMR (101 MHz, $CDCl_3$)

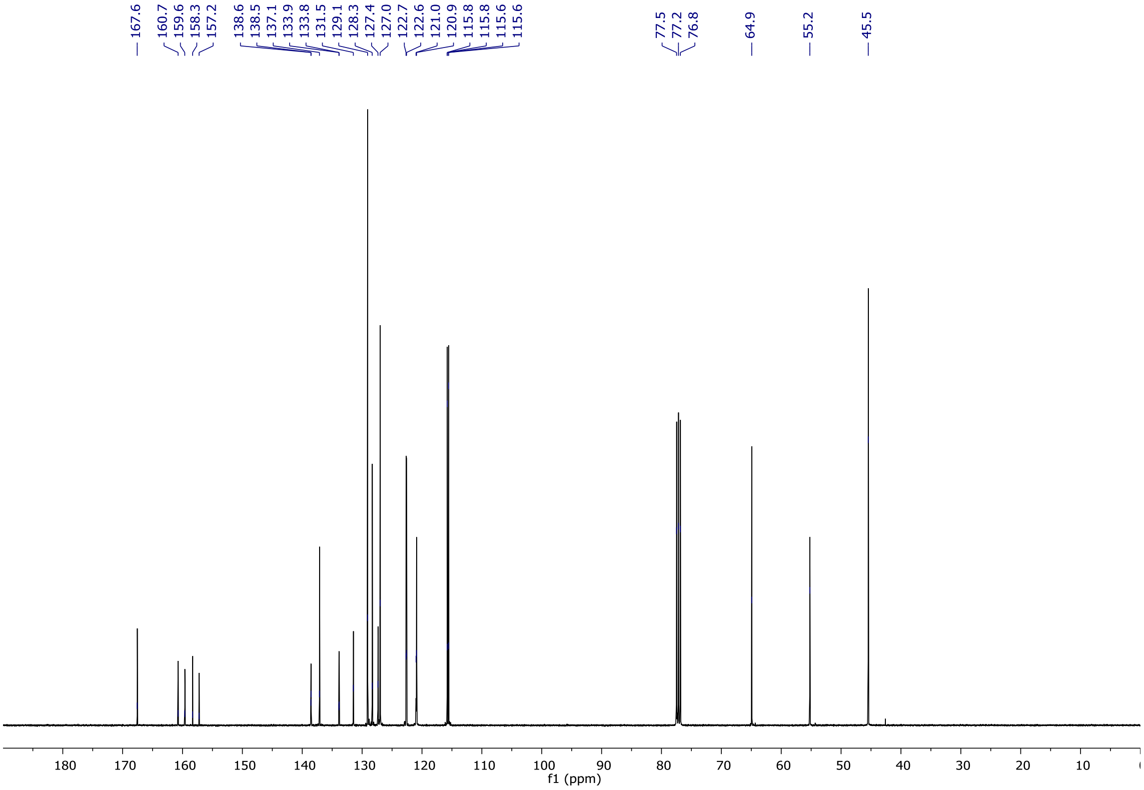

^{19}F {^{1}H} NMR (282 MHz, $CDCl_3$)

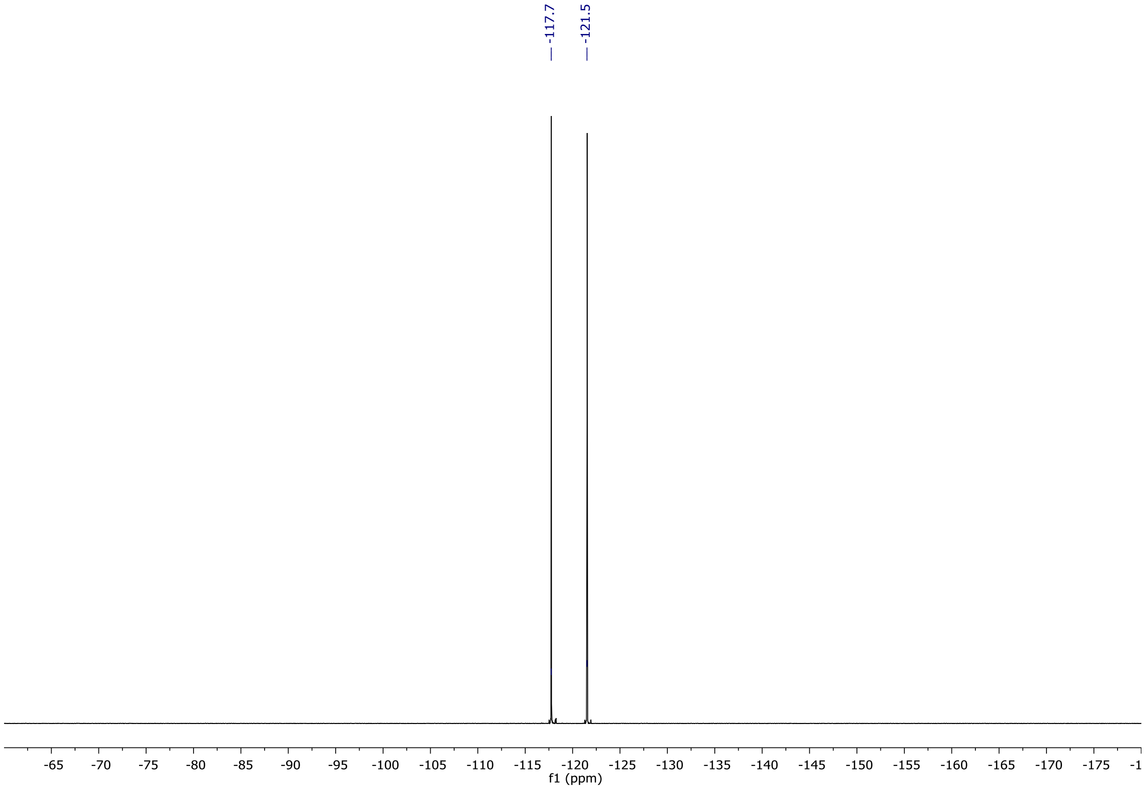

1-(3-Chlorophenyl)-3-((3-chlorophenyl)amino)-4-((dimethylamino)methyl)-5-phenyl-1,5-dihydro-2*H*-pyrrol-2-one (9g)

^{1}H NMR (400 MHz, $CDCl_3$)

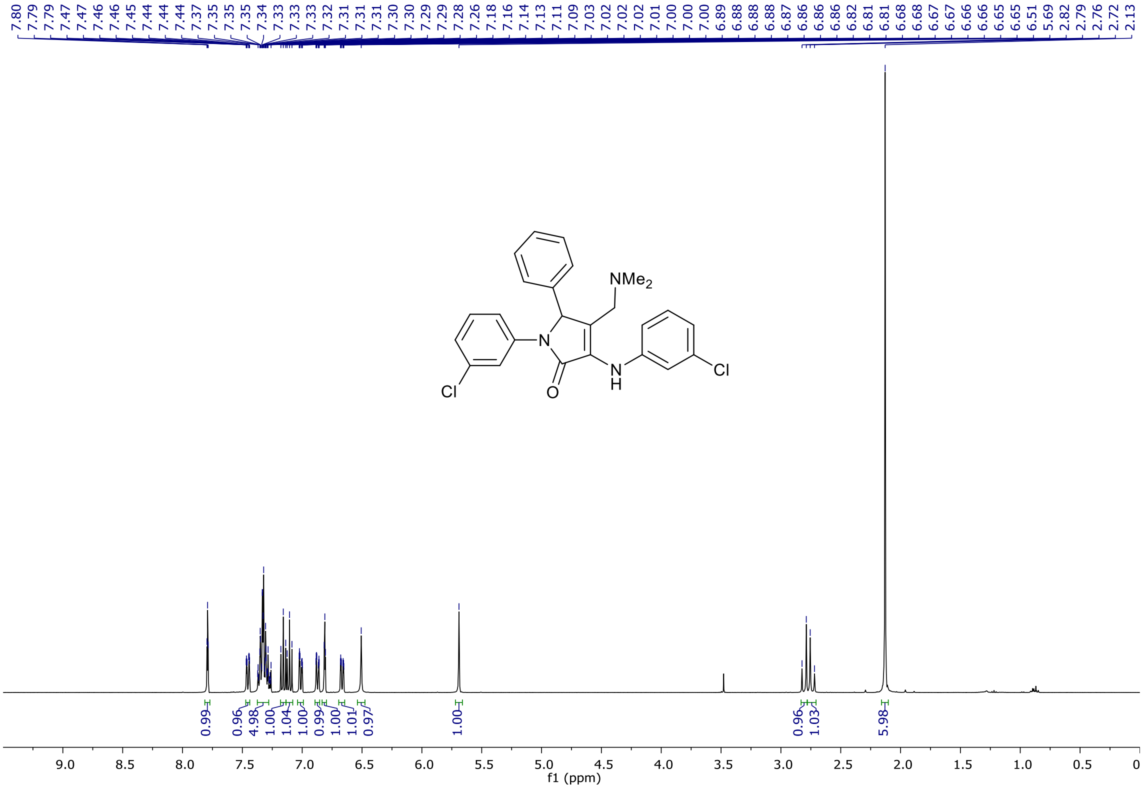

^{13}C {^{1}H} NMR (101 MHz, $CDCl_3$)

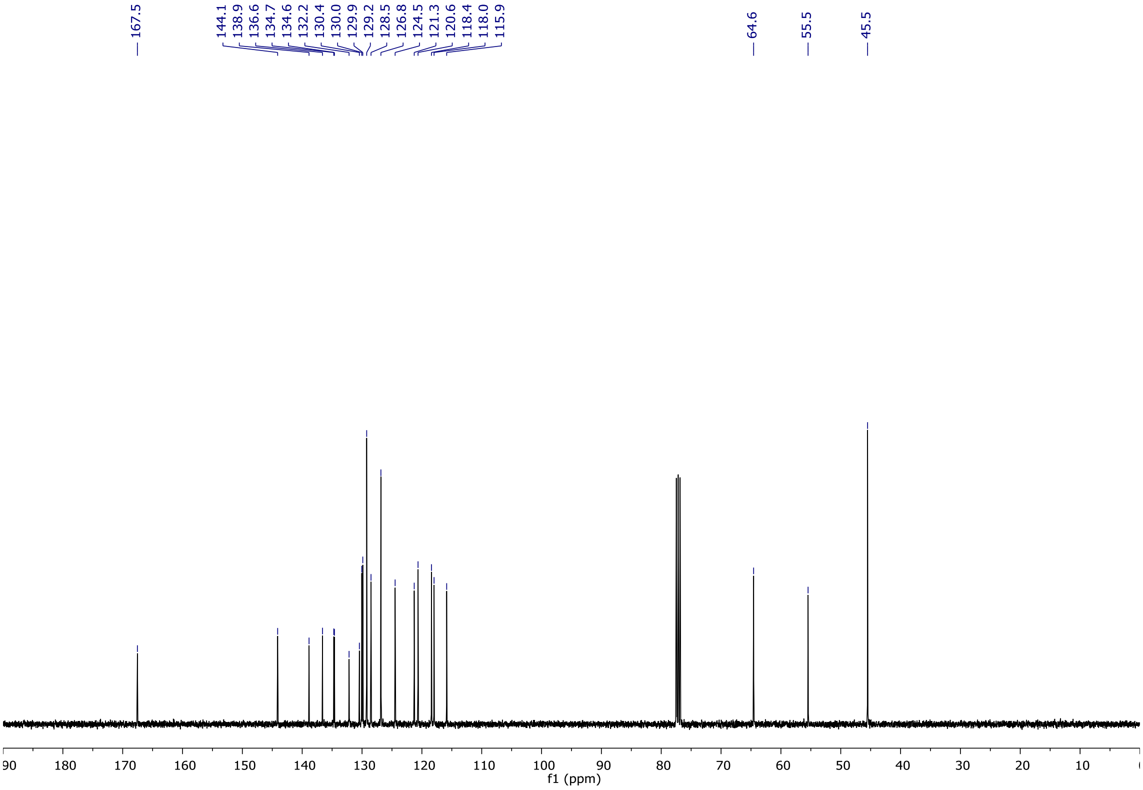

4-((Dimethylamino)methyl)-1-(2-fluorophenyl)-3-((2-fluorophenyl)amino)-5-phenyl-1,5-dihydro-2*H*-pyrrol-2-one (9h)

^{1}H NMR (400 MHz, $CDCl_3$)

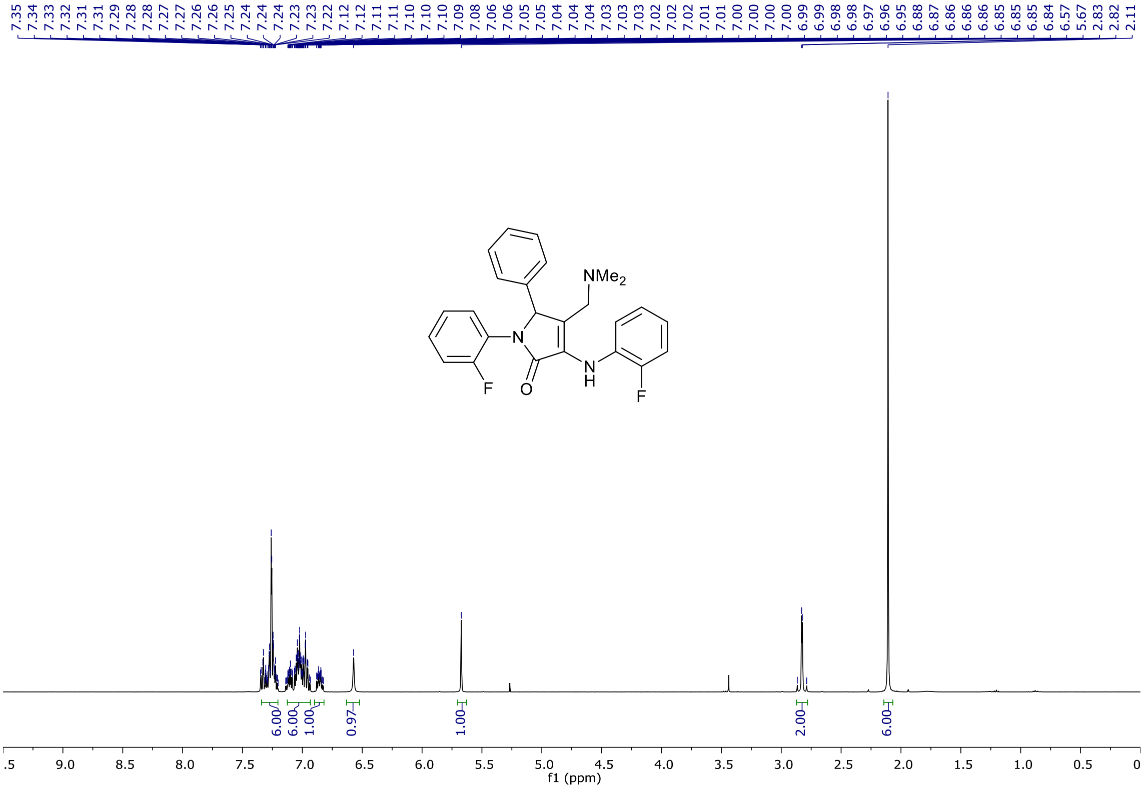

^{13}C {^{1}H} NMR (101 MHz, $CDCl_3$)

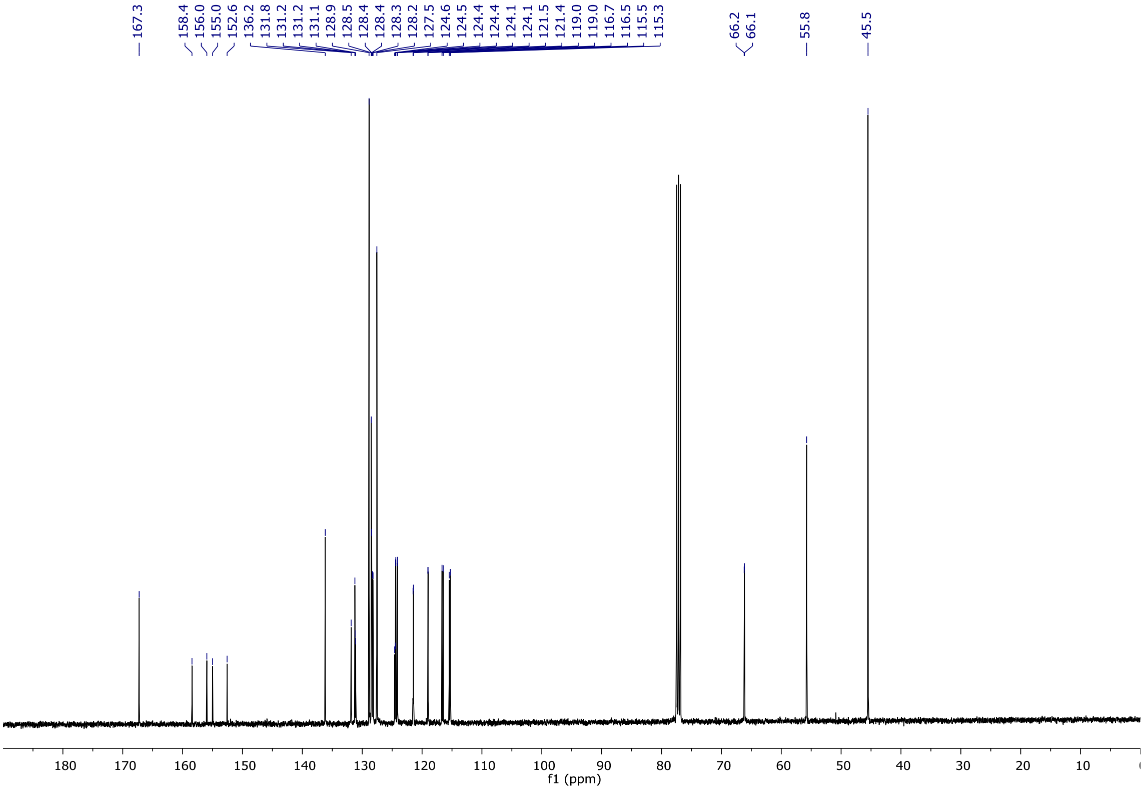

^{19}F {^{1}H} NMR (282 MHz, $CDCl_3$)

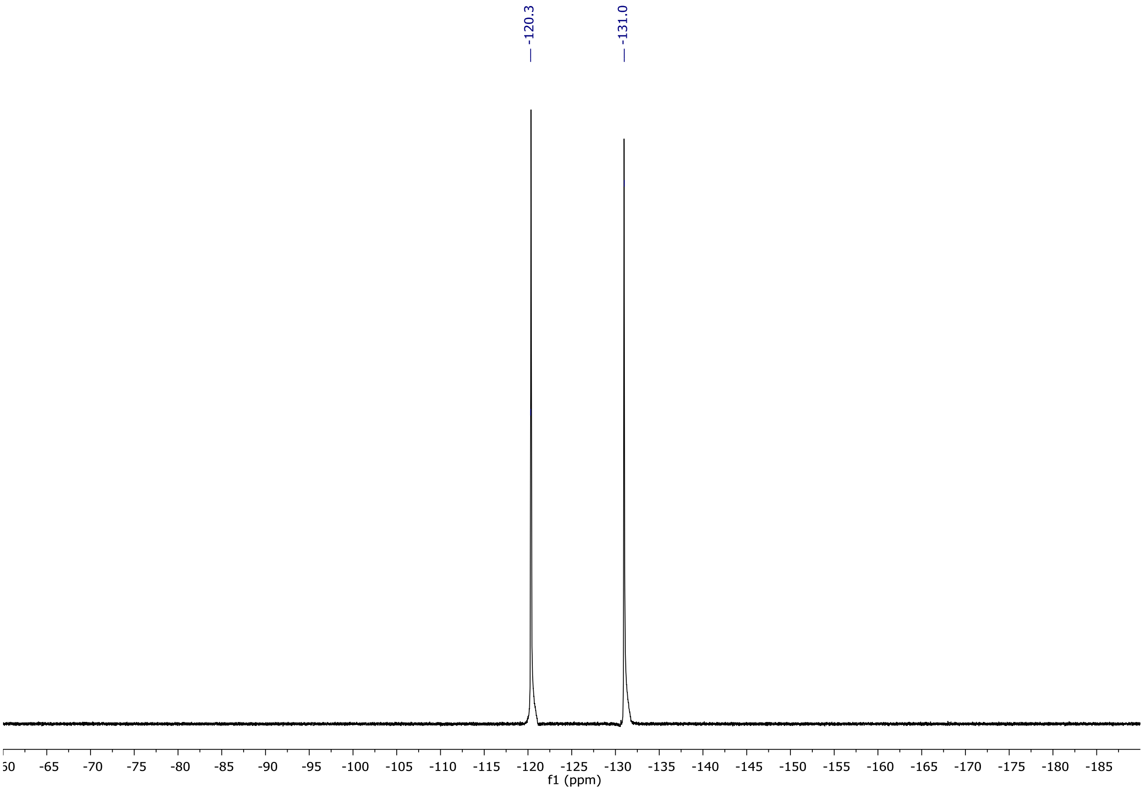

4-((Dimethylamino)methyl)-5-phenyl-1-(3-(trifluoromethyl)phenyl)-3-((3-(trifluoromethyl)phenyl) amino)-1,5-dihydro-2*H*-pyrrol-2-one (9i)

^{1}H NMR (400 MHz, $CDCl_3$)

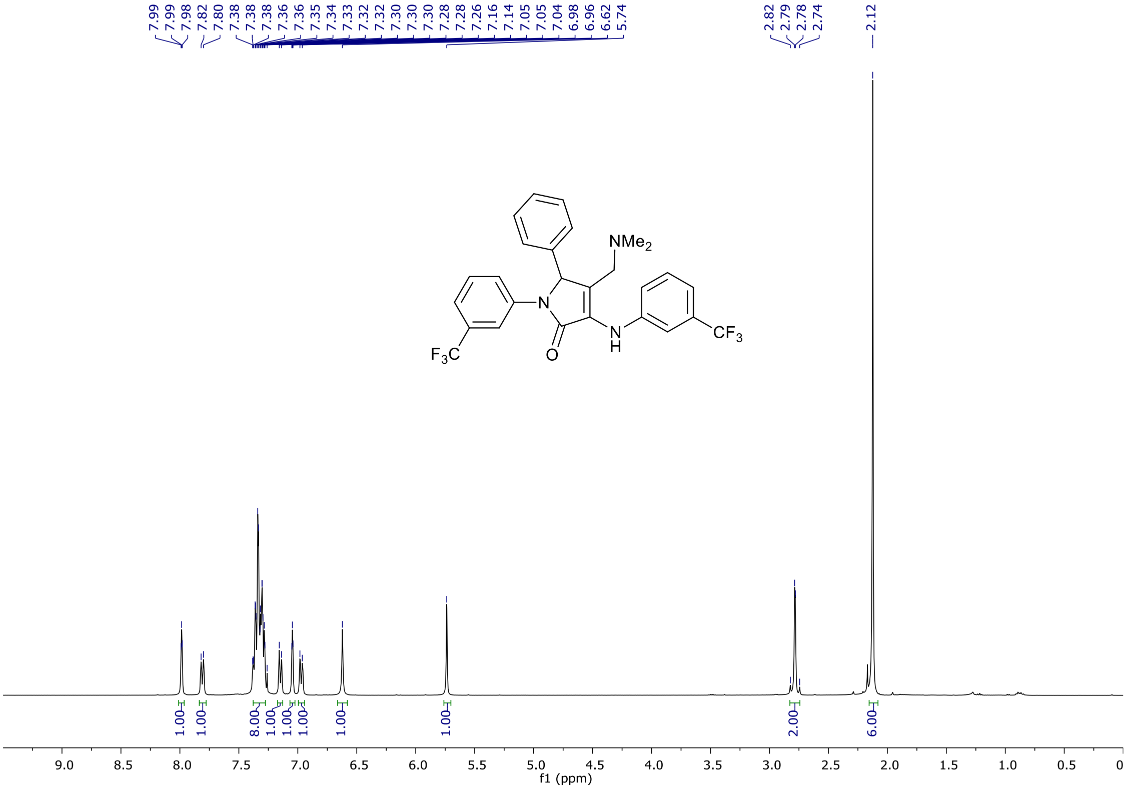

^{13}C {^{1}H} NMR (101 MHz, $CDCl_3$)

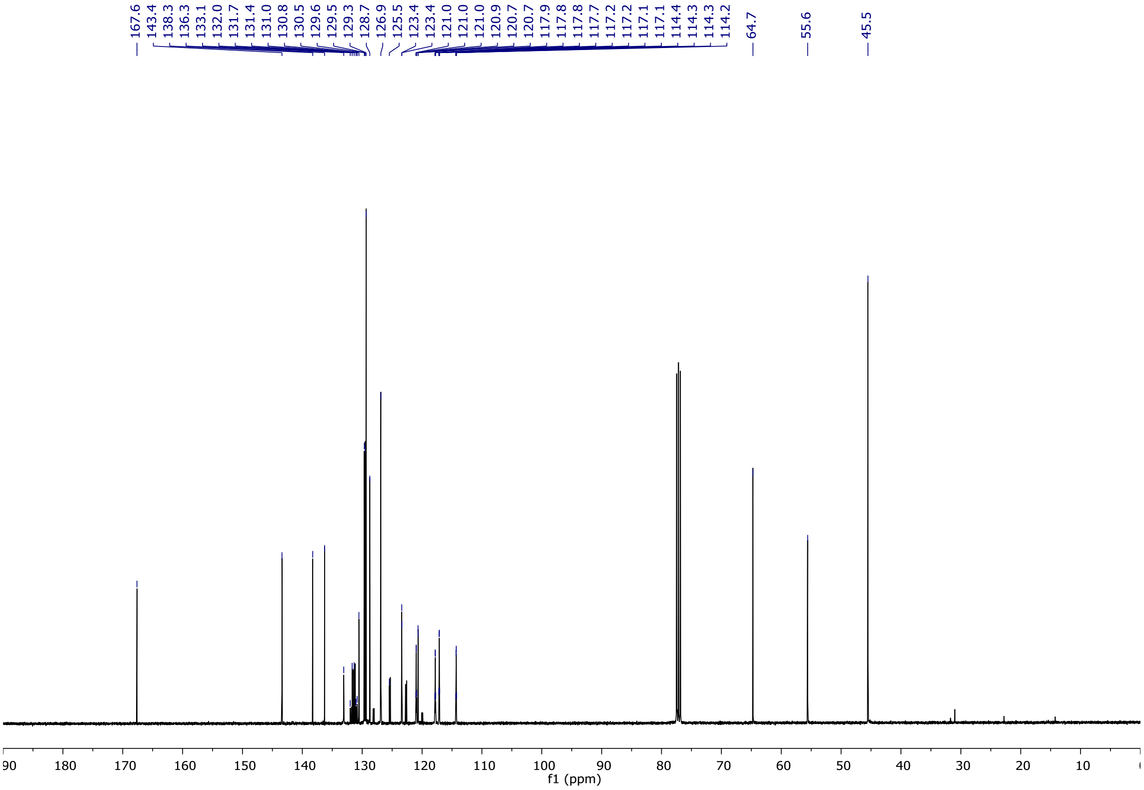

^{19}F {^{1}H} NMR (282 MHz, $CDCl_3$)

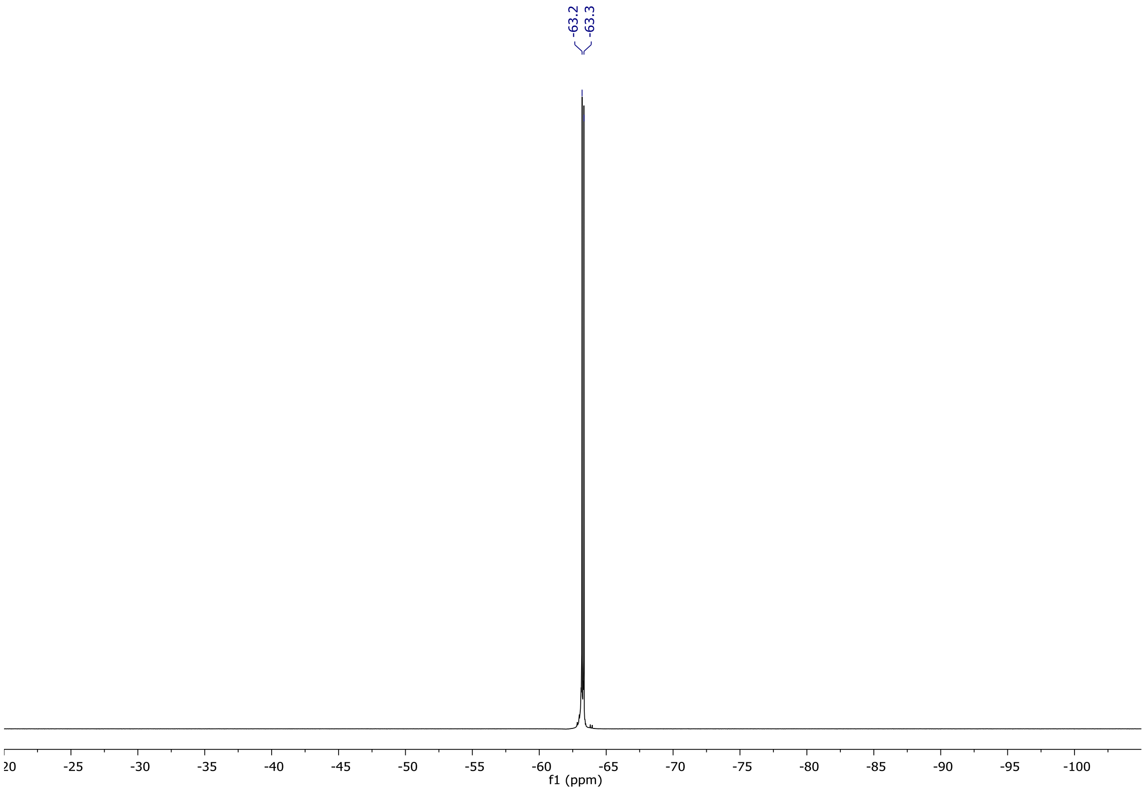

(5-Oxo-2-phenyl-1-(*p*-tolyl)-4-(*p*-tolylamino)-2,5-dihydro-1*H*-pyrrol-3-yl)methyl acetate (10a)

^{1}H NMR (400 MHz, $CDCl_3$)

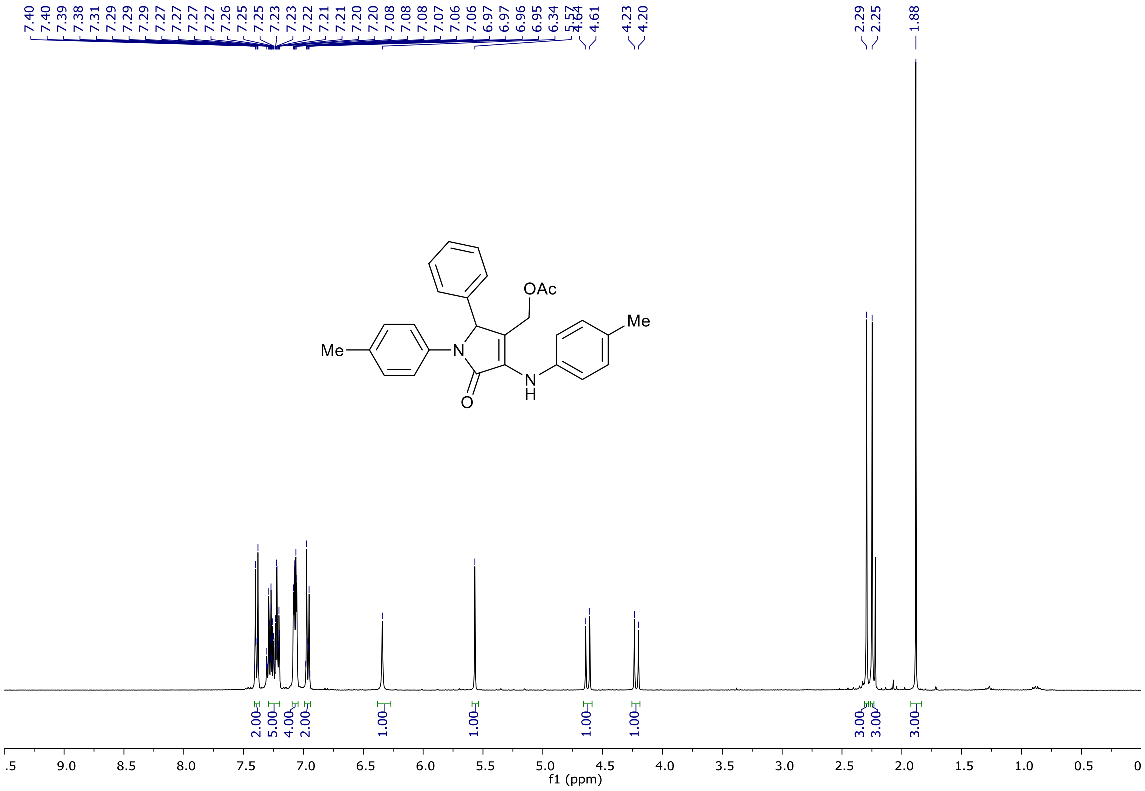

^{13}C {^{1}H} NMR (101 MHz, $CDCl_3$)

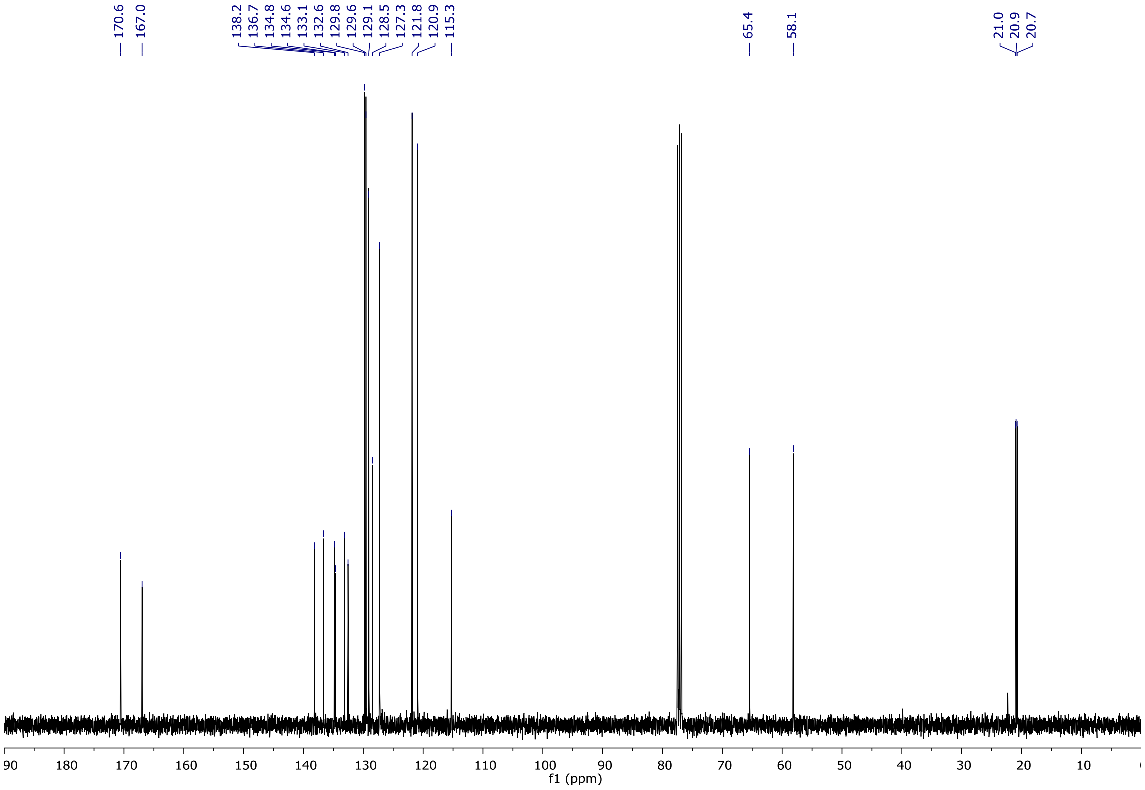

(*Z*)-1,1',6'-Tri-*p*-tolyl-4-(*p*-tolylimino)-3',4',5',6'-tetrahydrospiro[pyrrolidine-3,2'-pyrrolo[3,4-*b*]pyridine]-5,7'(1'*H*)-dione (5)

^{1}H NMR (400 MHz, $CDCl_3$)

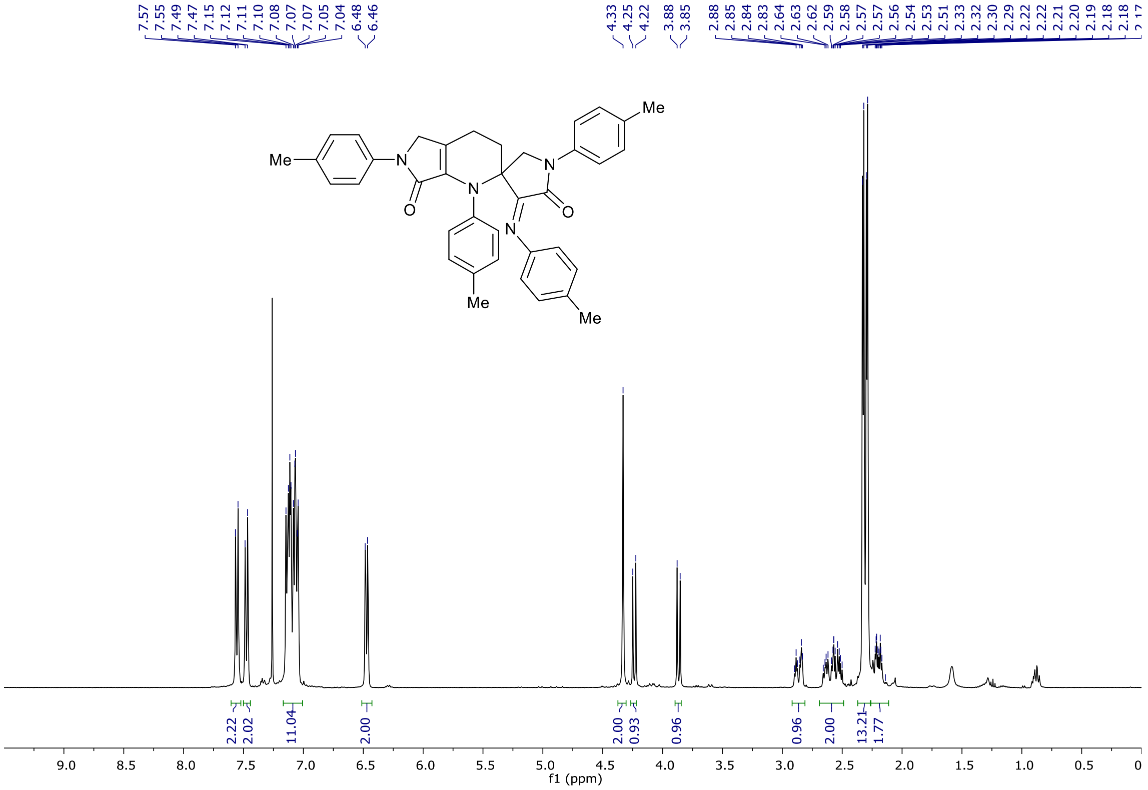

^{13}C {^{1}H} NMR (101 MHz, $CDCl_3$)

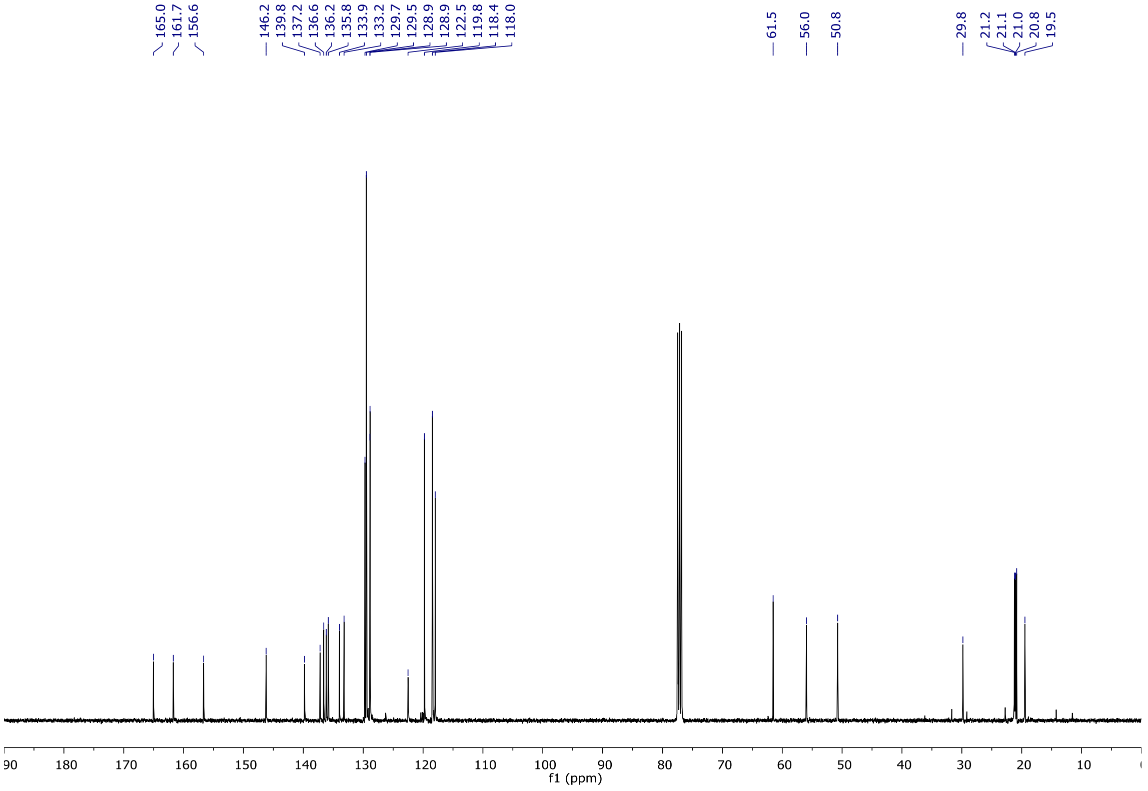

(2*S*,3*S*,5'*S*,*Z*)-2,5'-Diphenyl-1,1',6'-tri-*p*-tolyl-4-(*p*-tolylimino)-3',4',5',6'-tetrahydrospiro[pyrrolidine-3,2'-pyrrolo[3,4-*b*]pyridine]-5,7'(1'*H*)-dione (5´)

^{1}H NMR (400 MHz, $CDCl_3$)

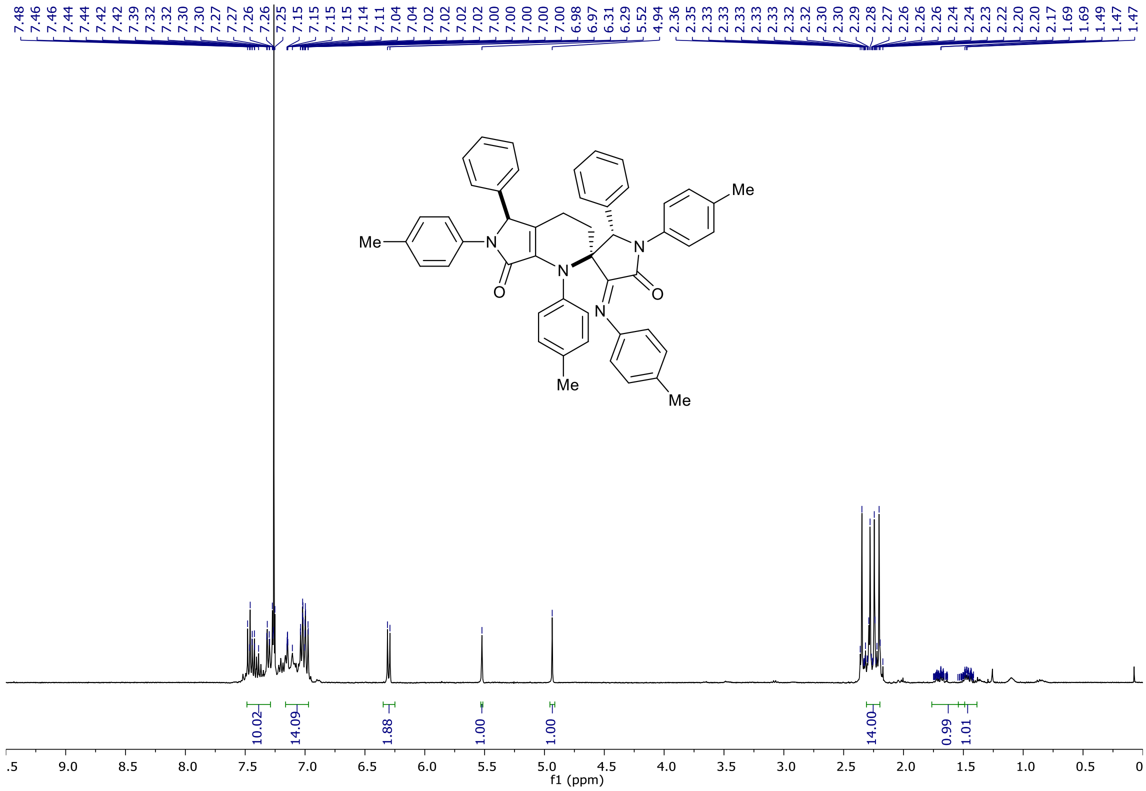

^{13}C NMR {^{1}H} (101 MHz, $CDCl_3$)

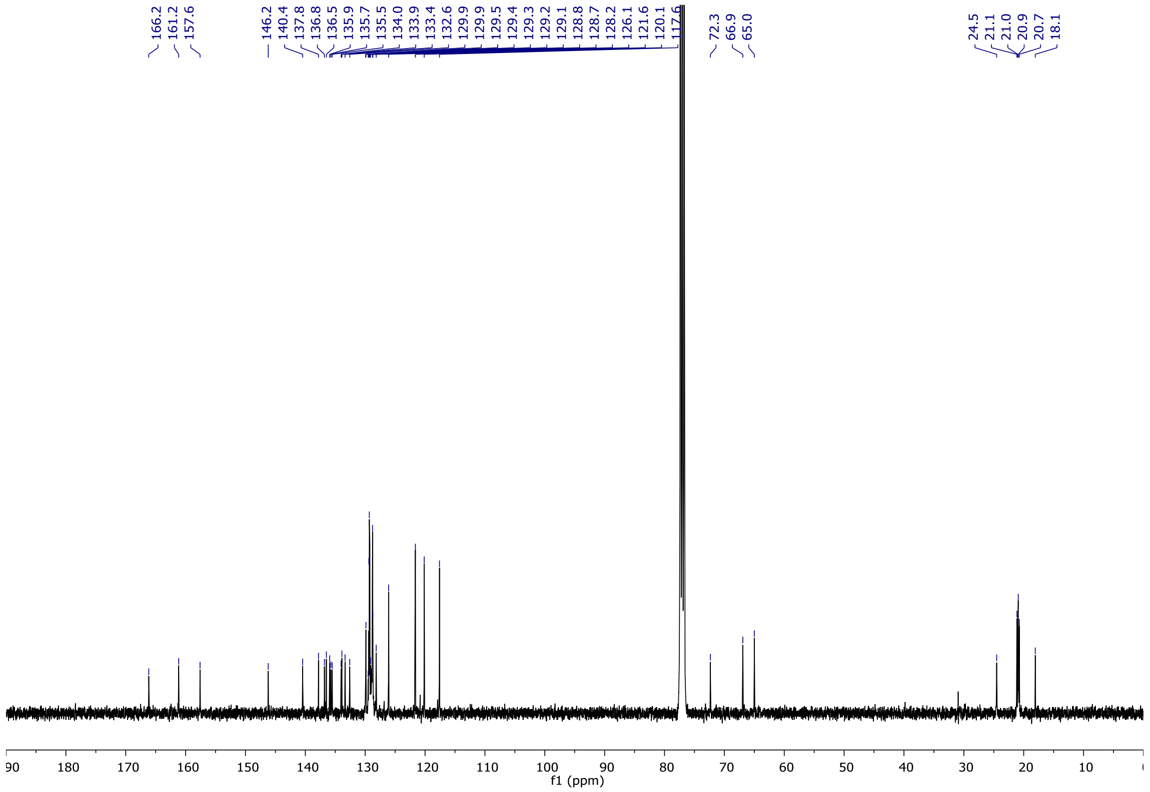

1,3,6-Tri-*p*-tolyl-1,2,3,4,5,6-hexahydro-7*H*-pyrrolo[3,4-*d*]pyrimidin-7-one (7).

^{1}H NMR (400 MHz, $CDCl_3$)

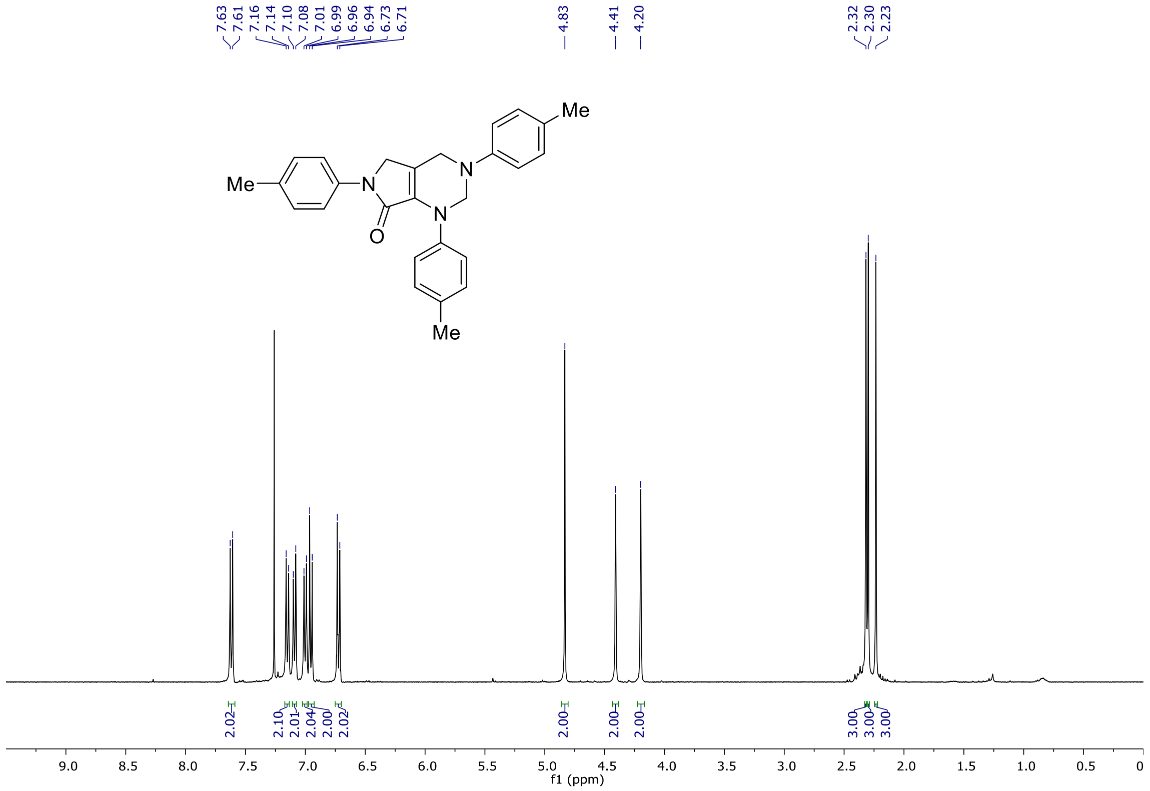

^{13}C {^{1}H} NMR (101 MHz, $CDCl_3$)

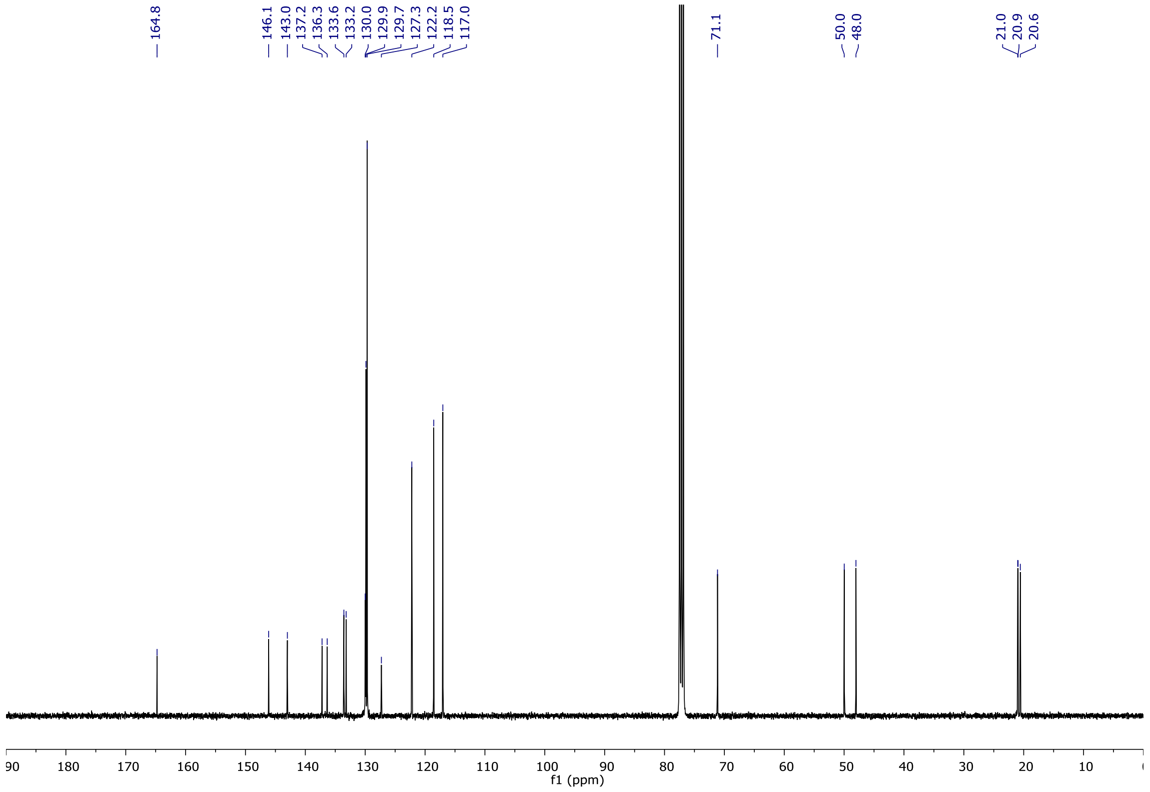

(1*S,2*R**,4*S**,*Z*)-1'-(*p*-Tolyl)-4'-(*p*-tolylimino)spiro[bicyclo[2.2.1]heptane-2,3'-pyrrolidin]-5-en-5'-one (6).**

^{1}H NMR (400 MHz, $CDCl_3$)

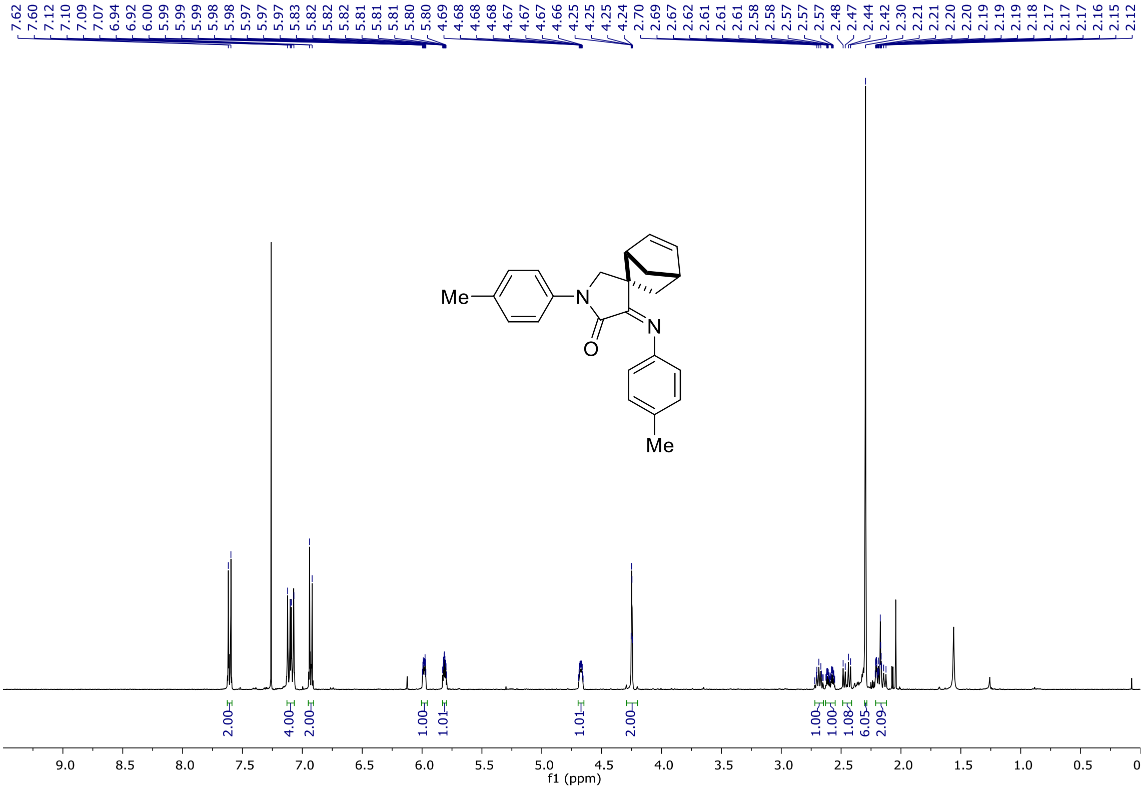

^{13}C {^{1}H} NMR (101 MHz, $CDCl_3$)

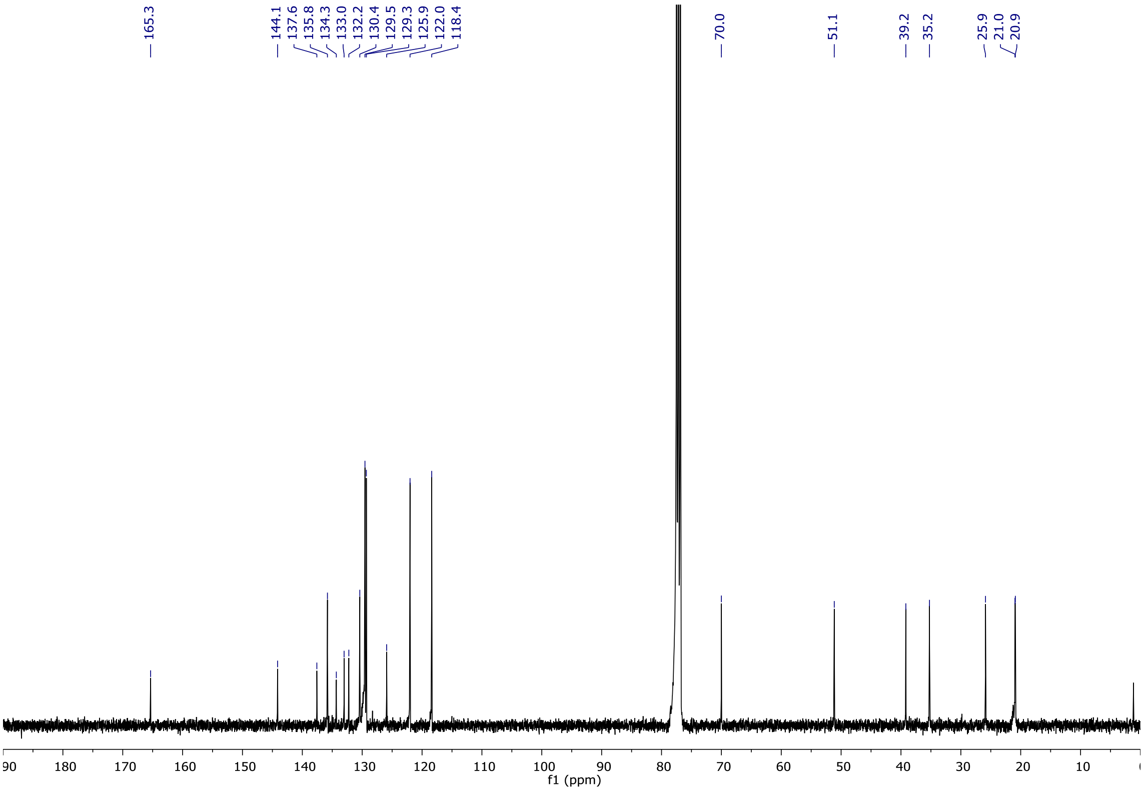

(1S*,2*R,2'*R**,4*S**,*Z*)-2'-Phenyl-1'-(*p*-tolyl)-4'-(*p*-tolylimino)spiro[bicyclo[2.2.1]heptane-2,3'-pyrrolidin]-5-en-5'-one (12a)**

^{1}H NMR (400 MHz, $CDCl_3$)

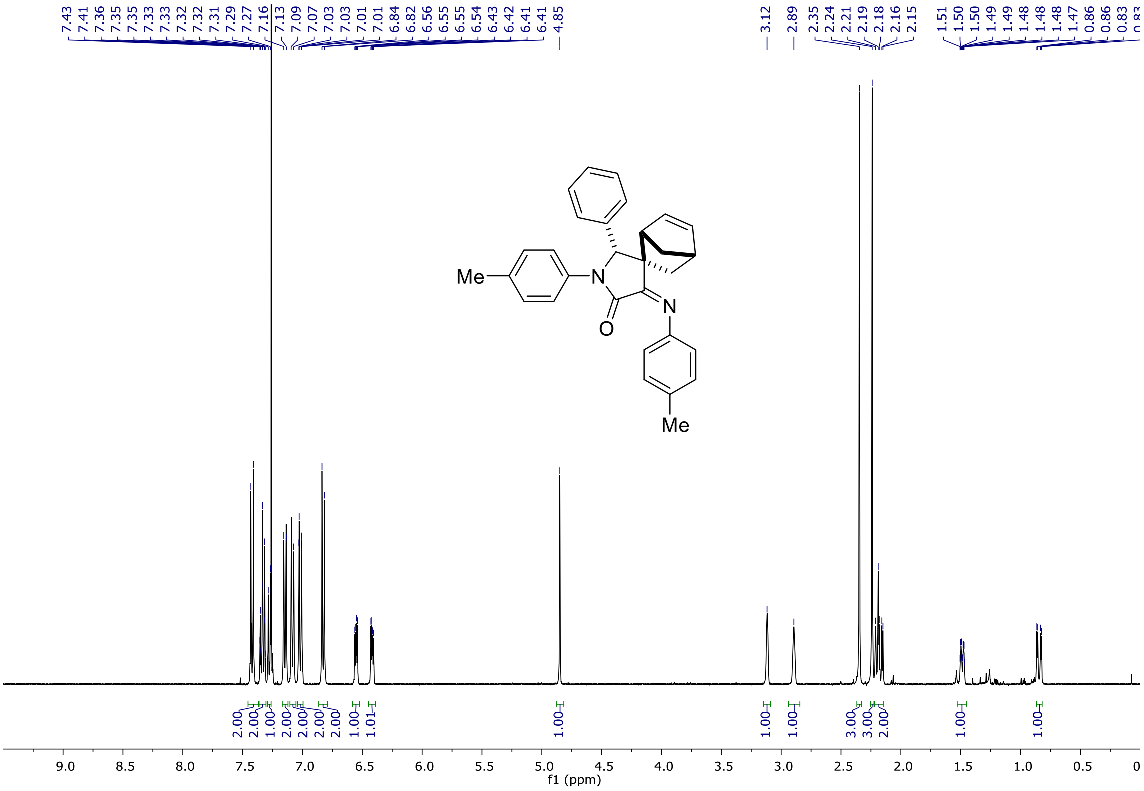

^{13}C {^{1}H} NMR (101 MHz, $CDCl_3$)

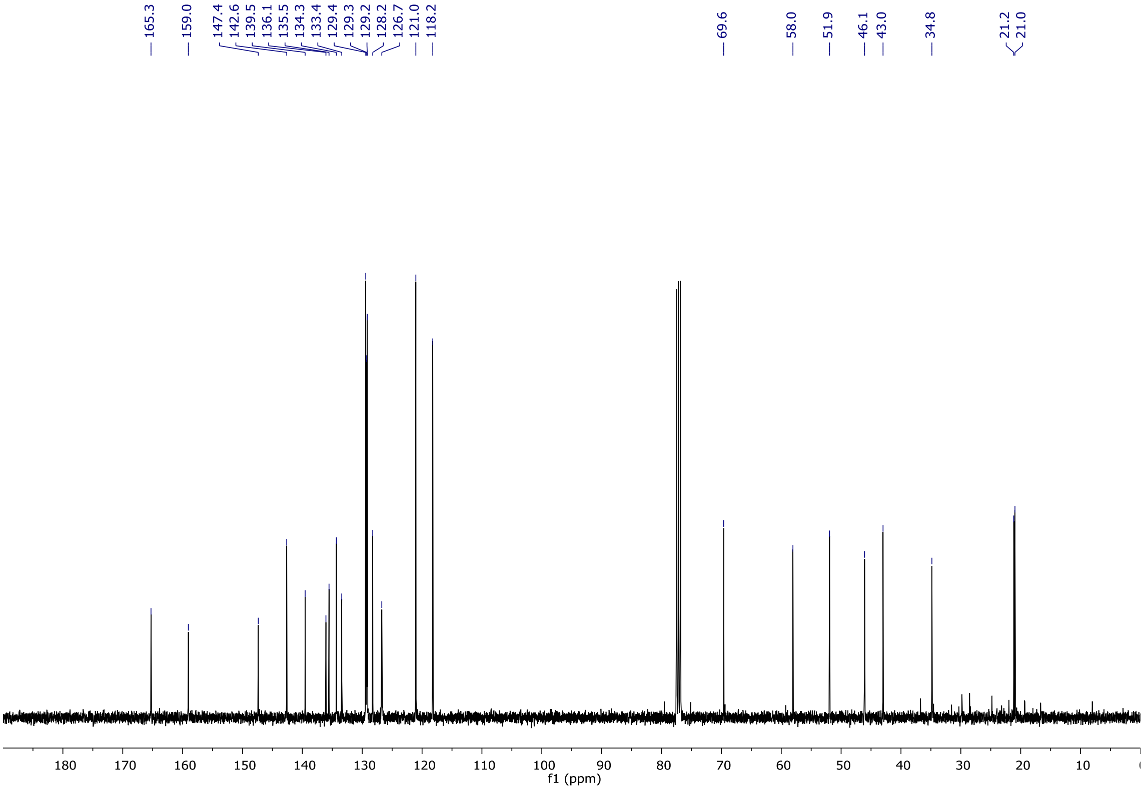

(1S*,2*R,2'*R**,4*S**,*Z*)-1',2'-Diphenyl-4'-(phenylimino)spiro[bicyclo[2.2.1]heptane-2,3'-pyrrolidin]-5-en-5'-one (12b)**

^{1}H NMR (400 MHz, $CDCl_3$)

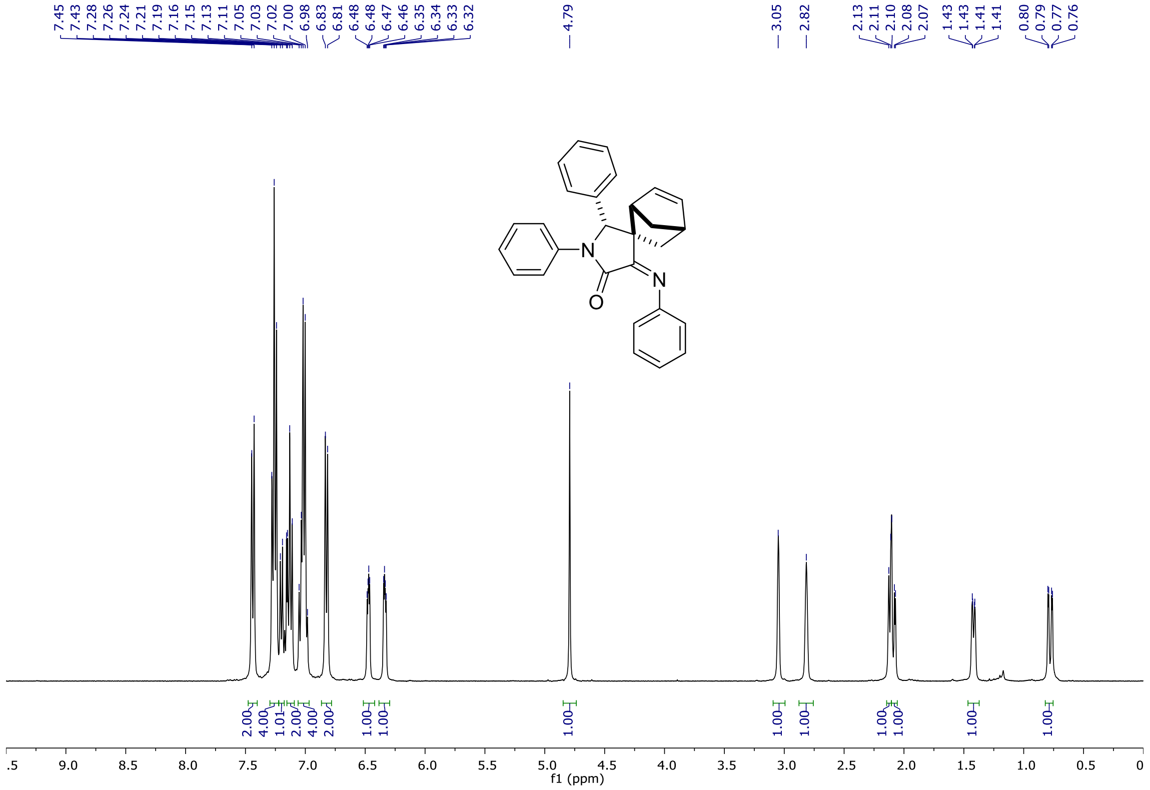

^{13}C {^{1}H} NMR (101 MHz, $CDCl_3$)

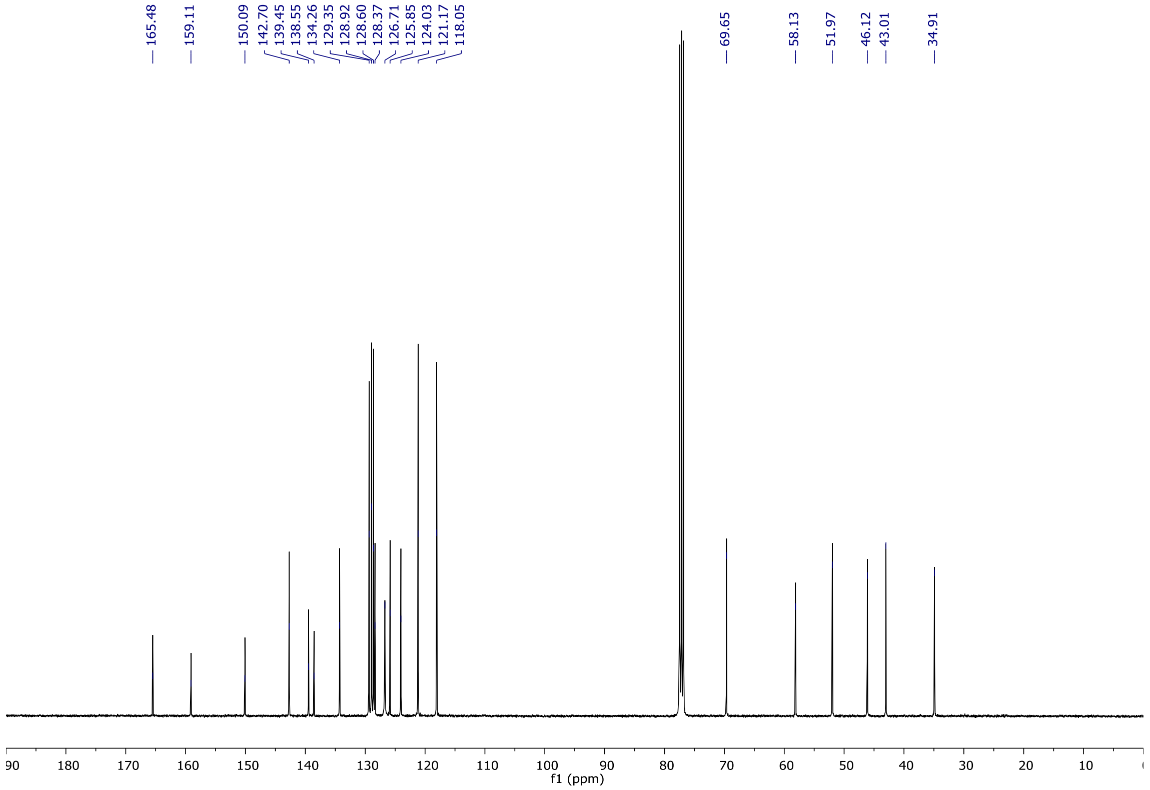

(1S*,2*R,2'*R**,4*S**,*Z*)-1'-(4-Methoxyphenyl)-4'-((4-methoxyphenyl)imino)-2'-phenylspiro[bicyclo[2.2.1]heptane-2,3'-pyrrolidin]-5-en-5'-one (12c)**

^{1}H NMR (400 MHz, $CDCl_3$)

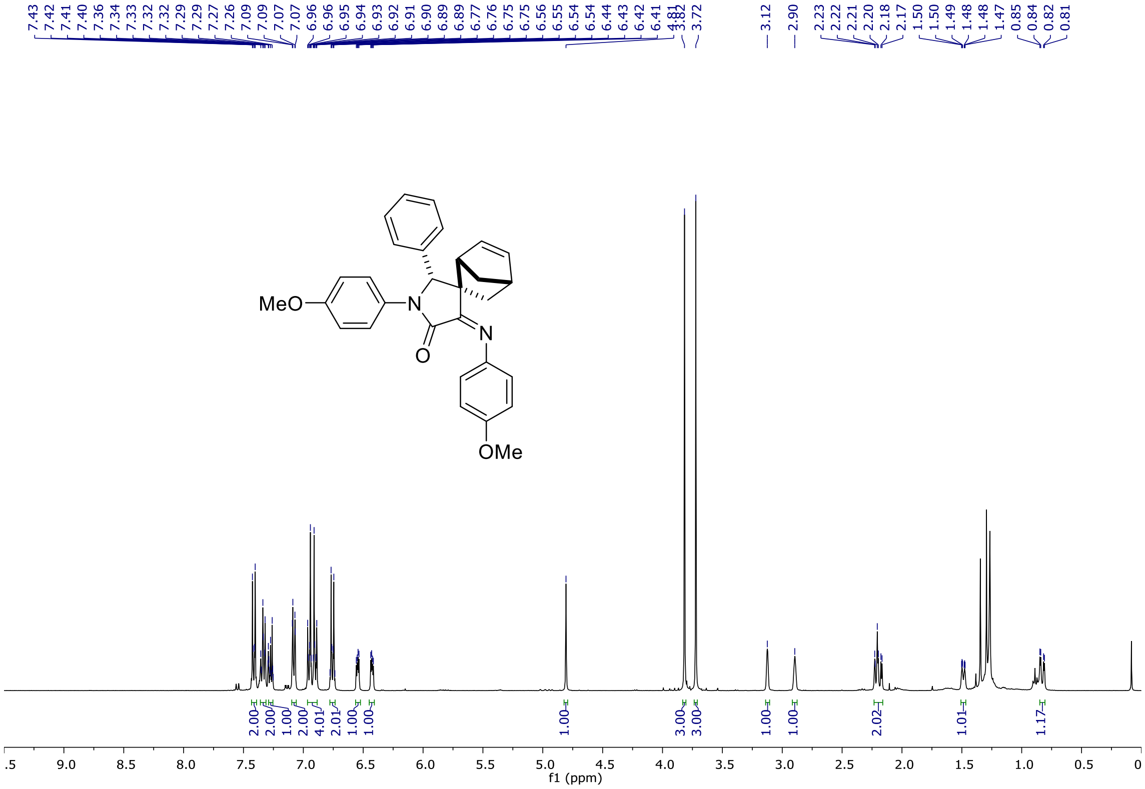

^{13}C {^{1}H} NMR (101 MHz, $CDCl_3$)

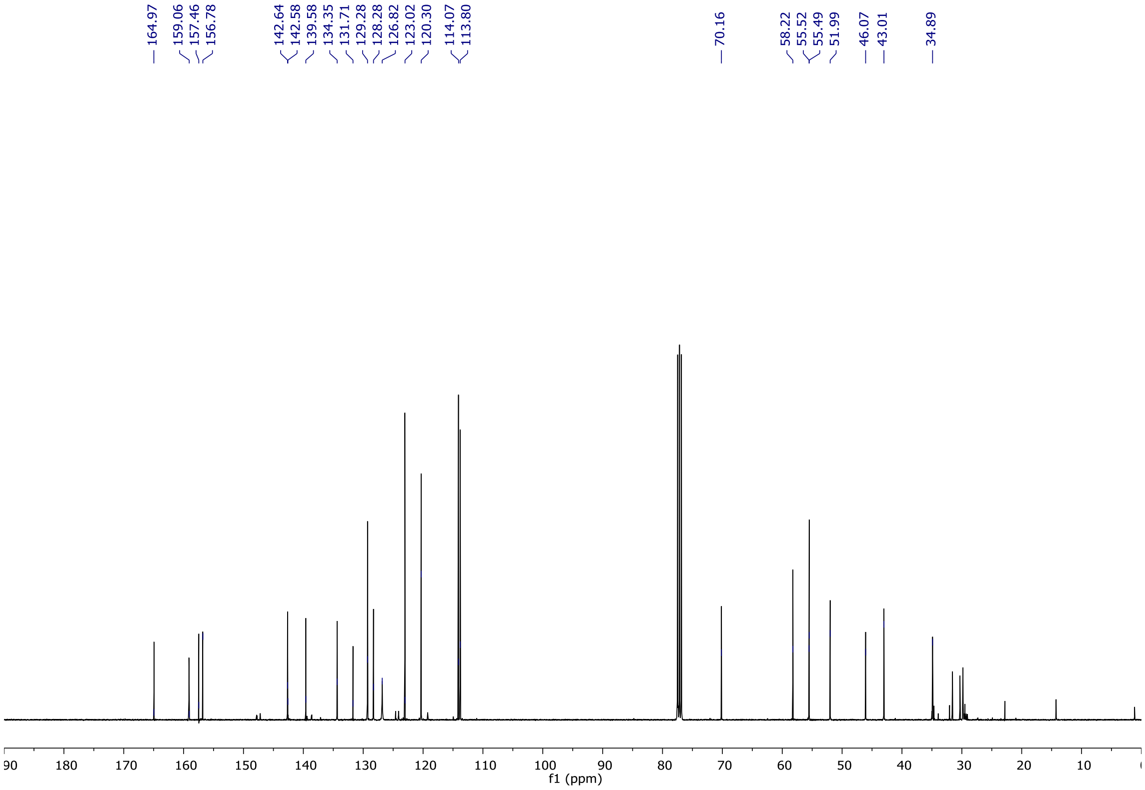

(1S*,2*R,2'*R**,4*S**,*Z*)-1'-(4-Bromophenyl)-4'-((4-bromophenyl)imino)-2'-phenylspiro[bicyclo[2.2.1] heptane-2,3'-pyrrolidin]-5-en-5'-one (12d)**

^{1}H NMR (400 MHz, $CDCl_3$)

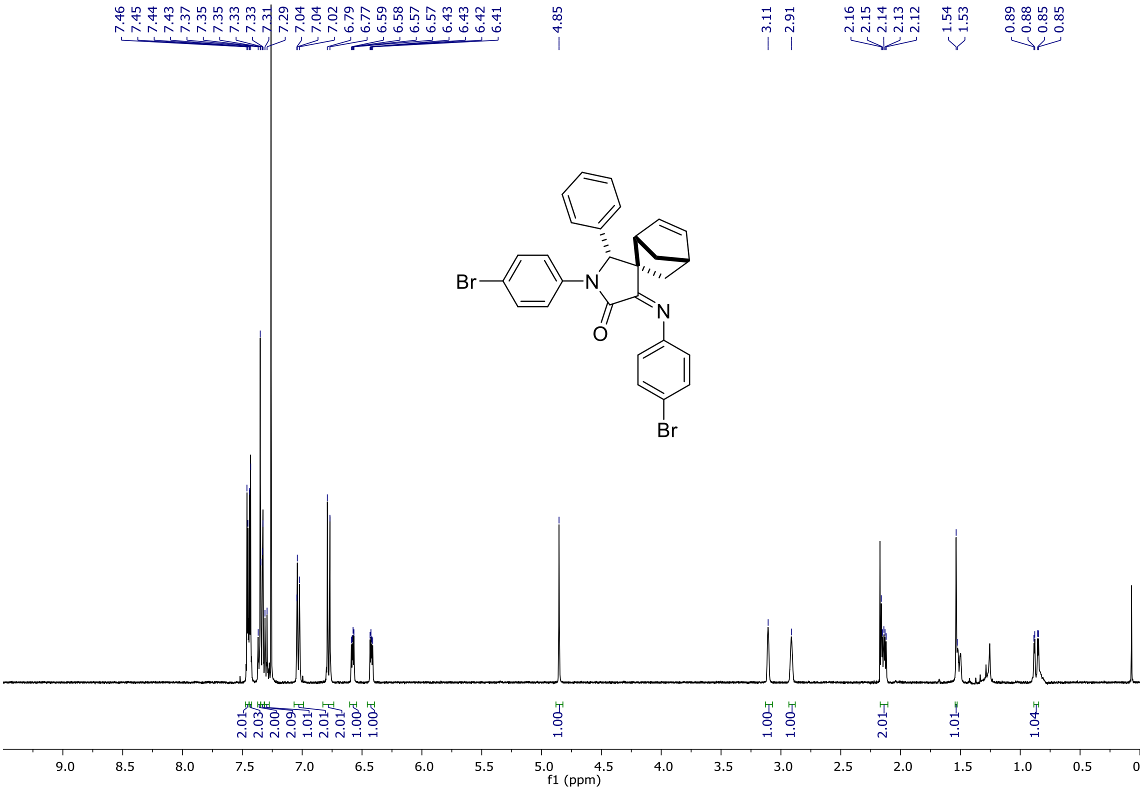

^{13}C {^{1}H} NMR (101 MHz, $CDCl_3$)

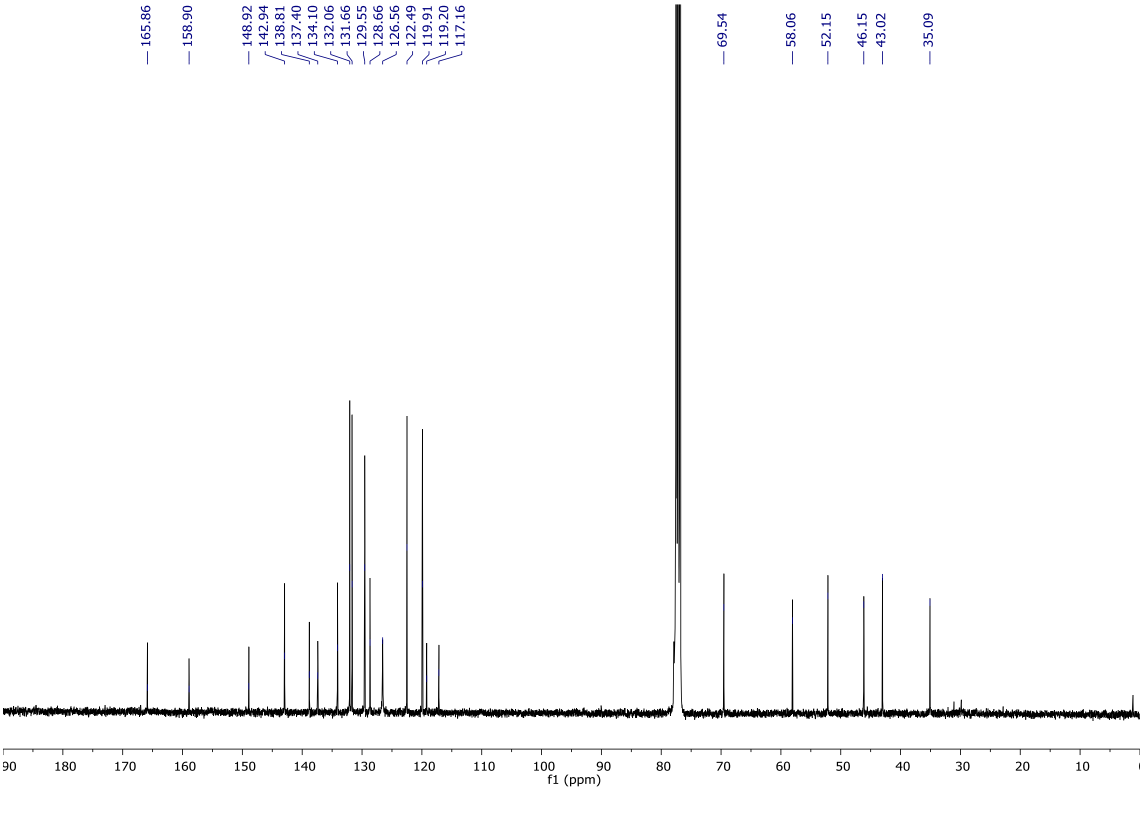

(1S*,2R*,2'R*,4S*,Z)-1'-(4-Chlorophenyl)-4'-((4-chlorophenyl)imino)-2'-phenylspiro[bicyclo[2.2.1]heptane-2,3'-pyrrolidin]-5-en-5'-one (12e)

^{1}H NMR (400 MHz, $CDCl_3$)

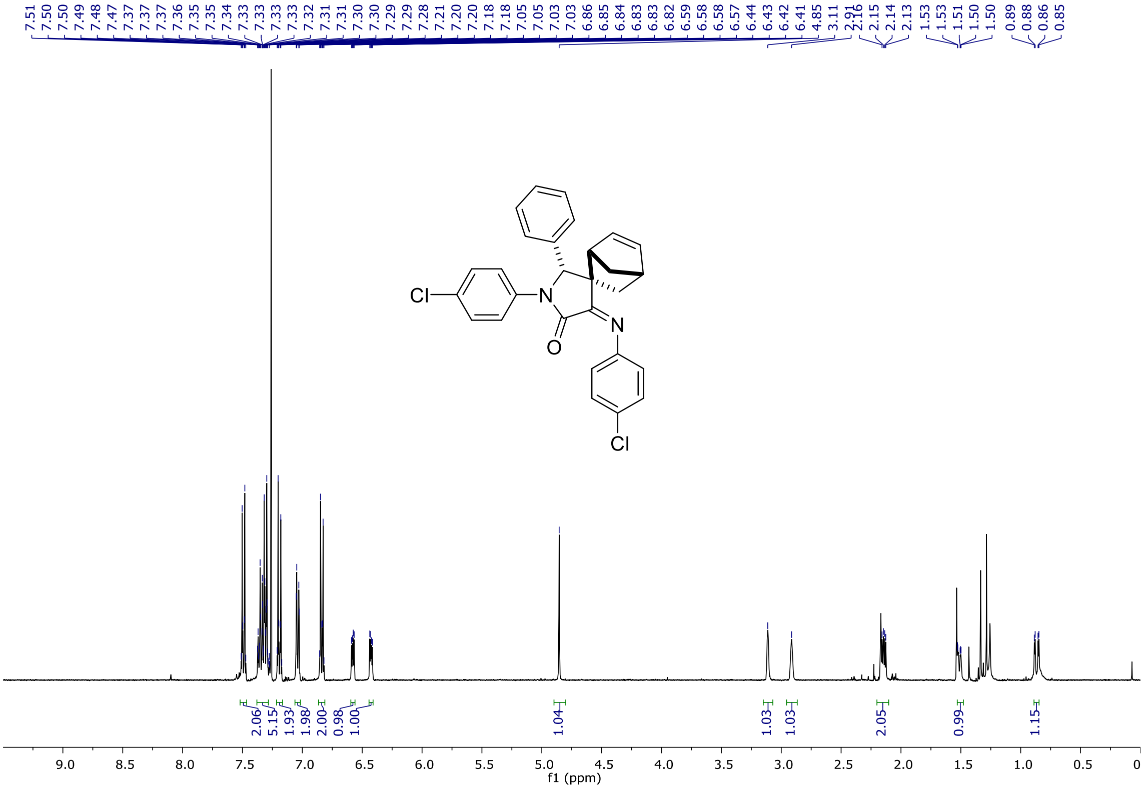

^{13}C {^{1}H} NMR (101 MHz, $CDCl_3$)

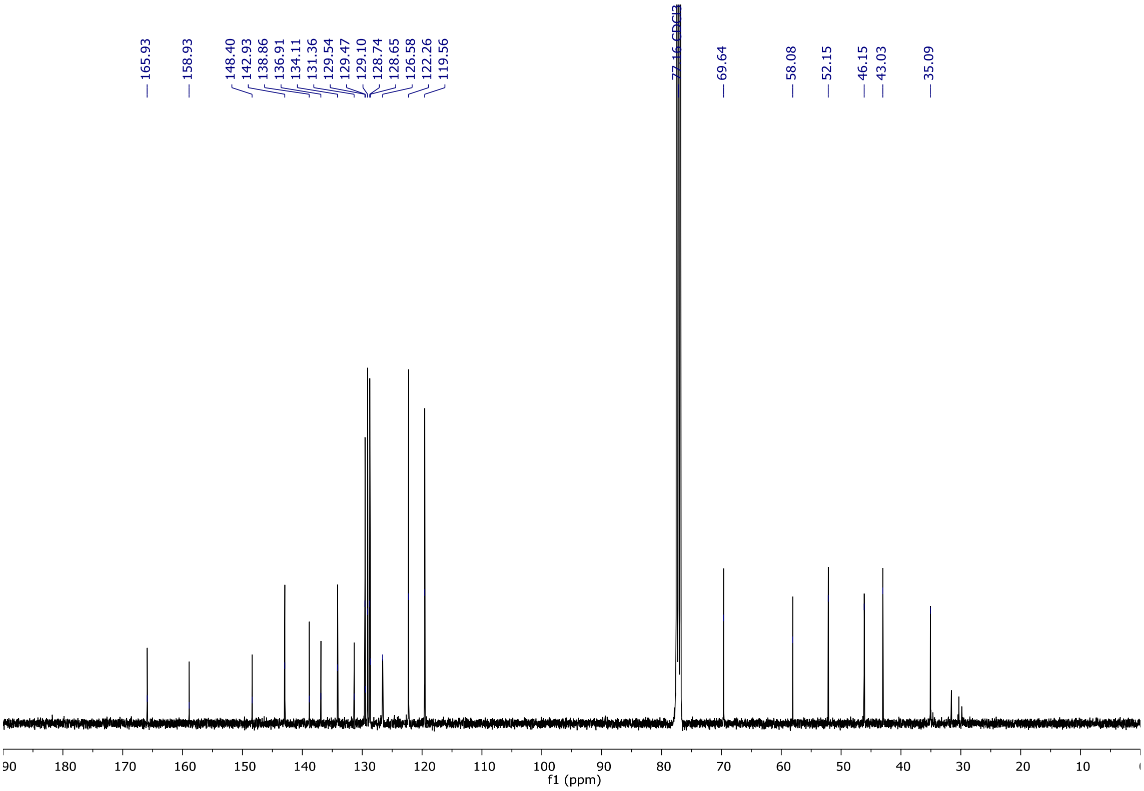

(1S*,2*R,2'*R**,4*S**,*Z*)-1'-(4-Fluorophenyl)-4'-((4-fluorophenyl)imino)-2'-phenylspiro[bicyclo[2.2.1]heptane-2,3'-pyrrolidin]-5-en-5'-one (12f)**

^{1}H NMR (400 MHz, $CDCl_3$)

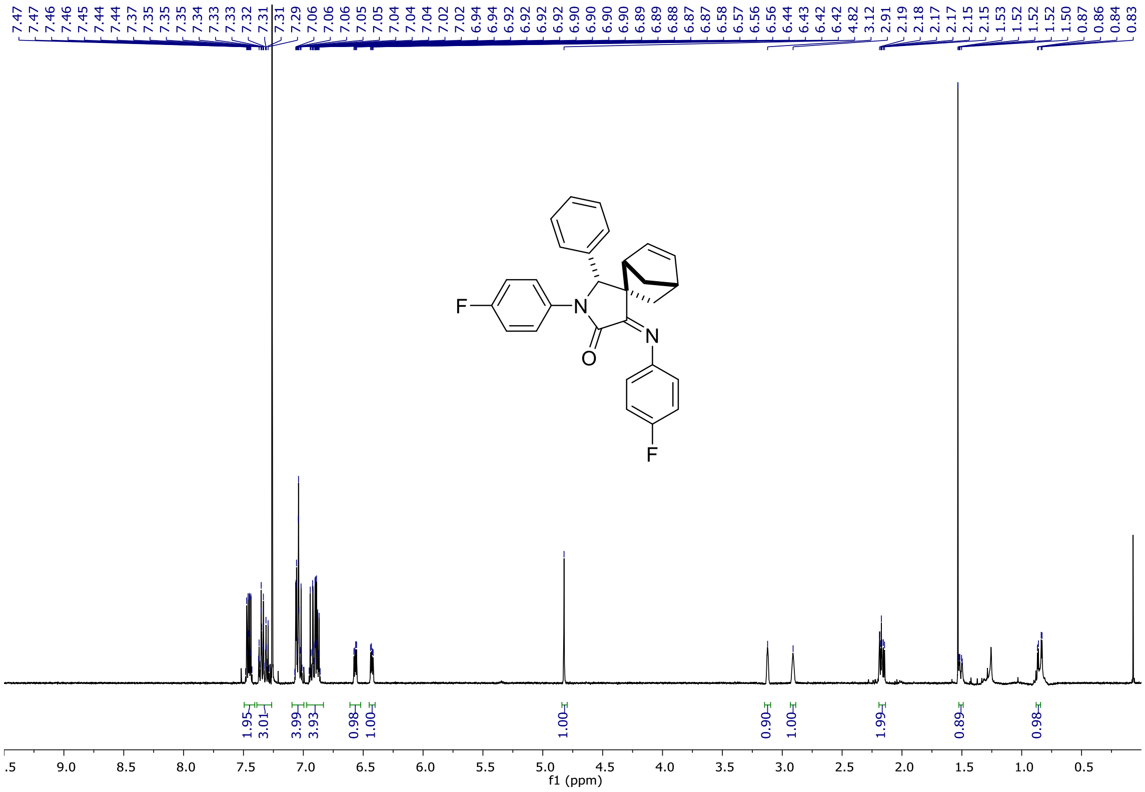

^{13}C {^{1}H} NMR (101 MHz, $CDCl_3$)

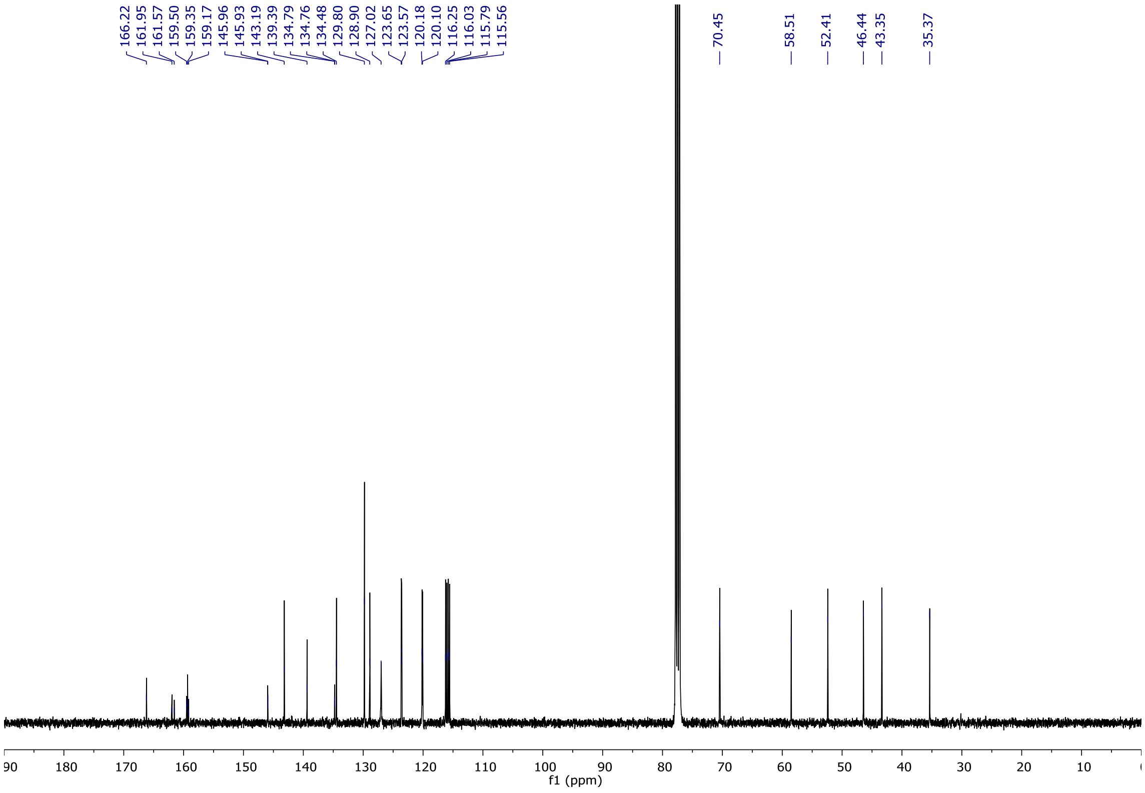

^{19}F {^{1}H} NMR (282 MHz, $CDCl_3$)

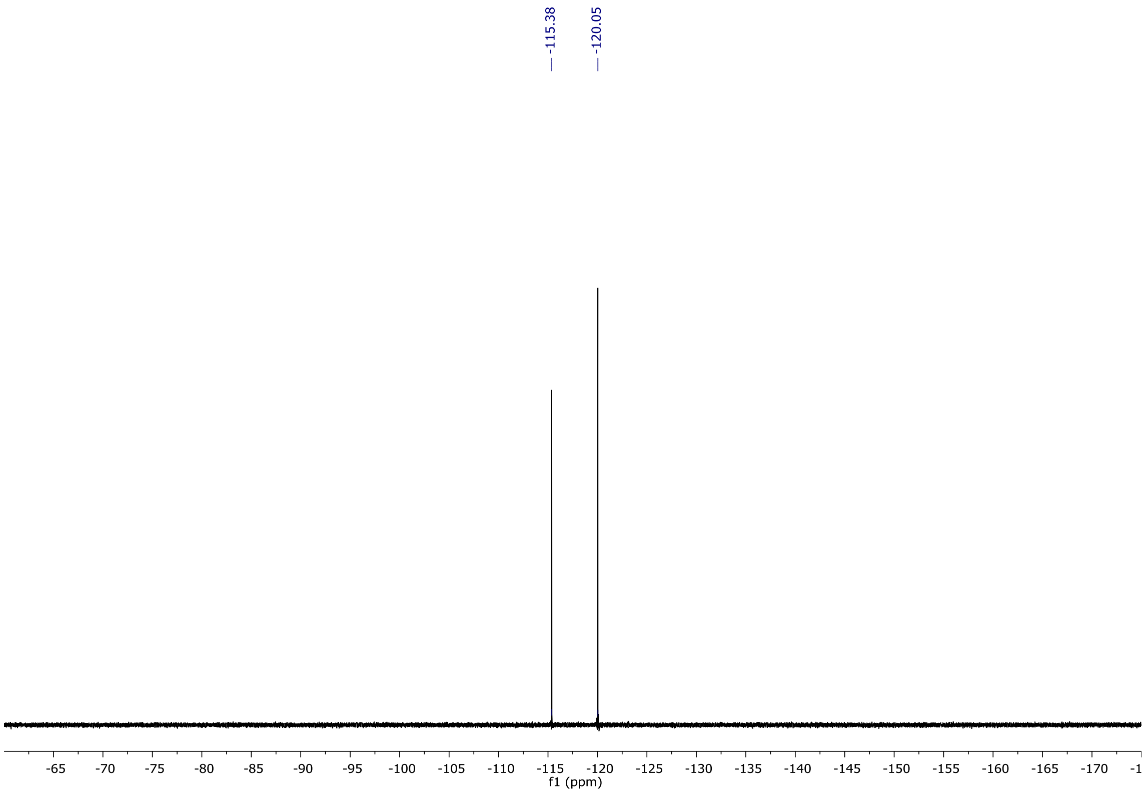

(1S*,2*R,2'*R**,4*S**,*Z*)-1'-(3-Chlorophenyl)-4'-((3-chlorophenyl)imino)-2'-phenylspiro[bicyclo[2.2.1] heptane-2,3'-pyrrolidin]-5-en-5'-one (12g)**

^{1}H NMR (400 MHz, $CDCl_3$)

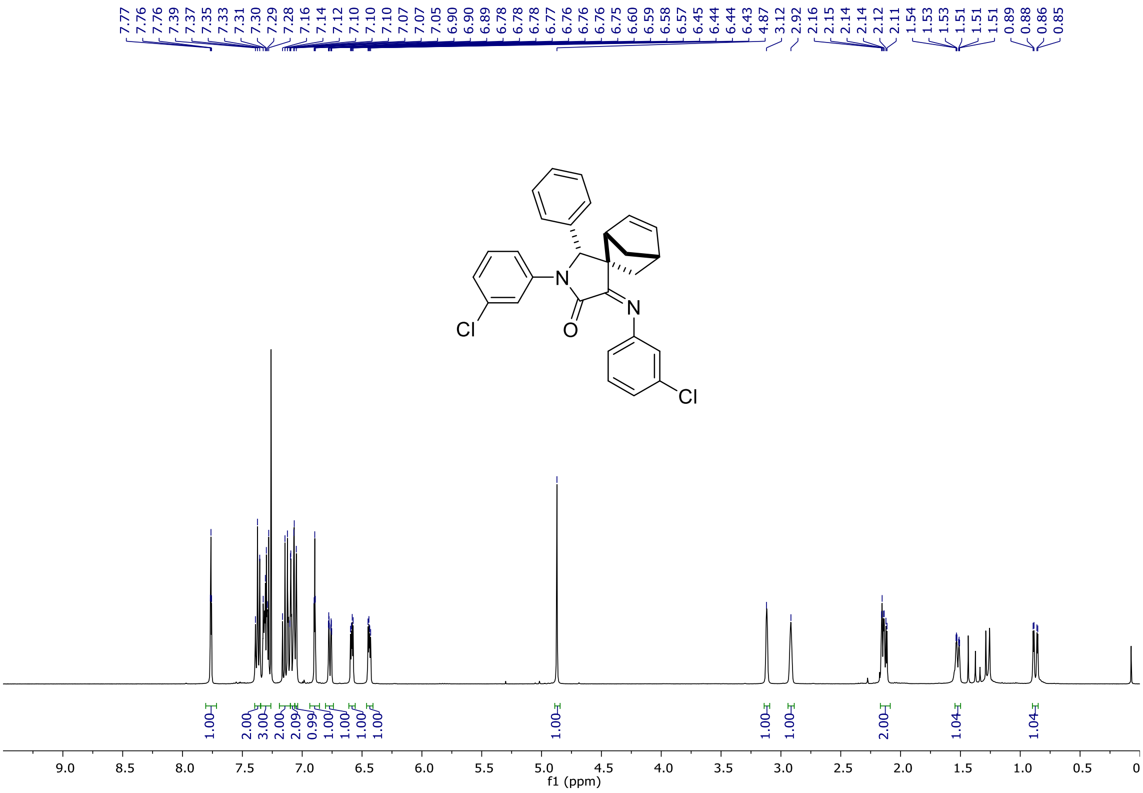

^{13}C {^{1}H} NMR (101 MHz, $CDCl_3$)

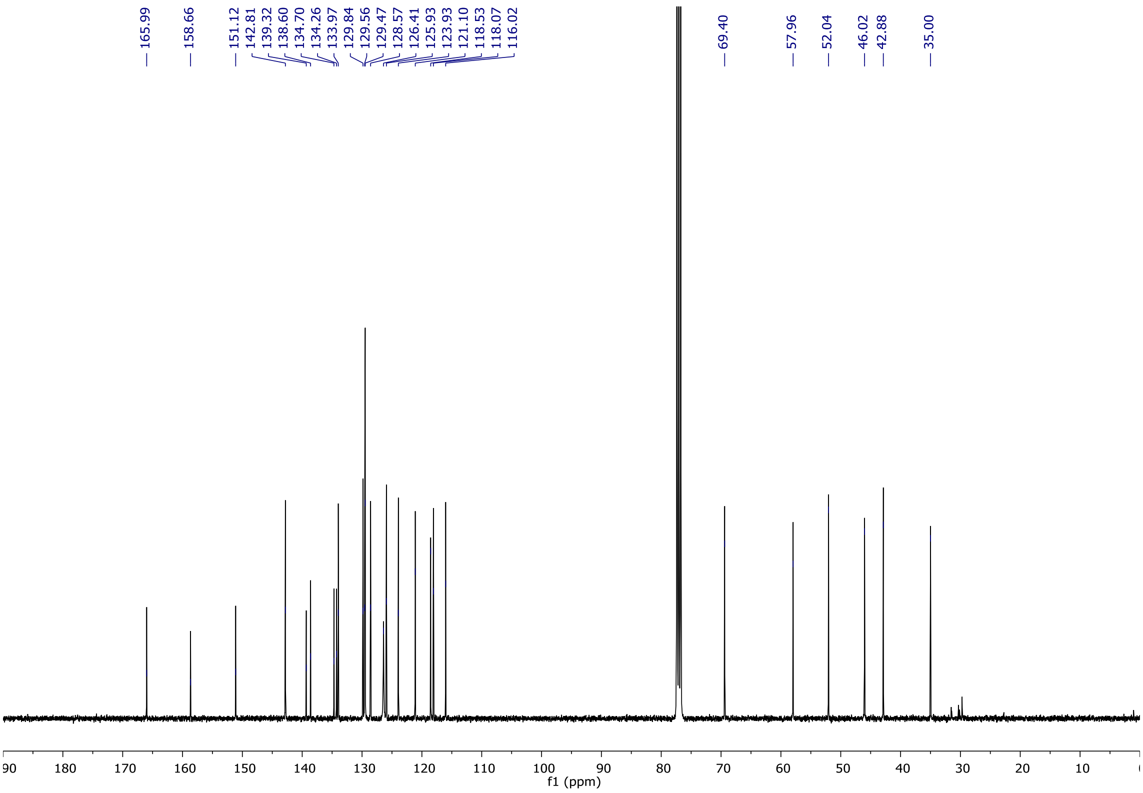

(1S*,2*R,2'*R**,4*S**,*Z*)-1'-(2-Fluorophenyl)-4'-((2-fluorophenyl)imino)-2'-phenylspiro[bicyclo[2.2.1] heptane-2,3'-pyrrolidin]-5-en-5'-one (12h)**

^{1}H NMR (400 MHz, $CDCl_3$)

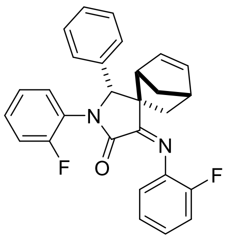

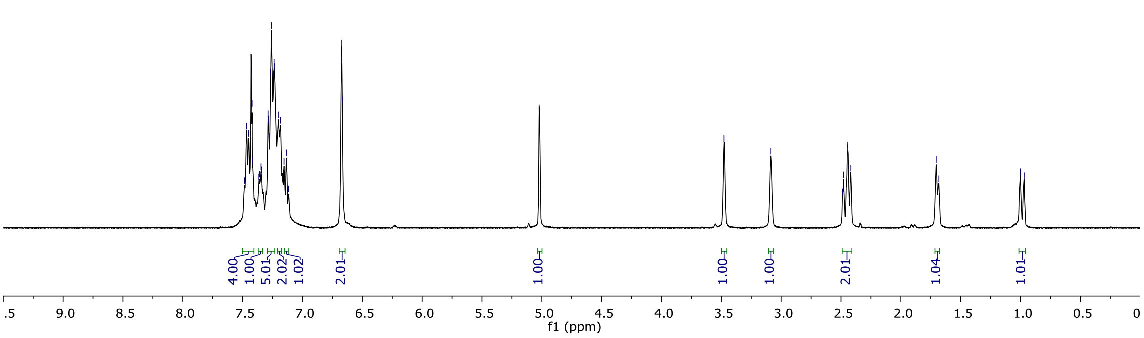

^{13}C {^{1}H} NMR (101 MHz, $CDCl_3$)

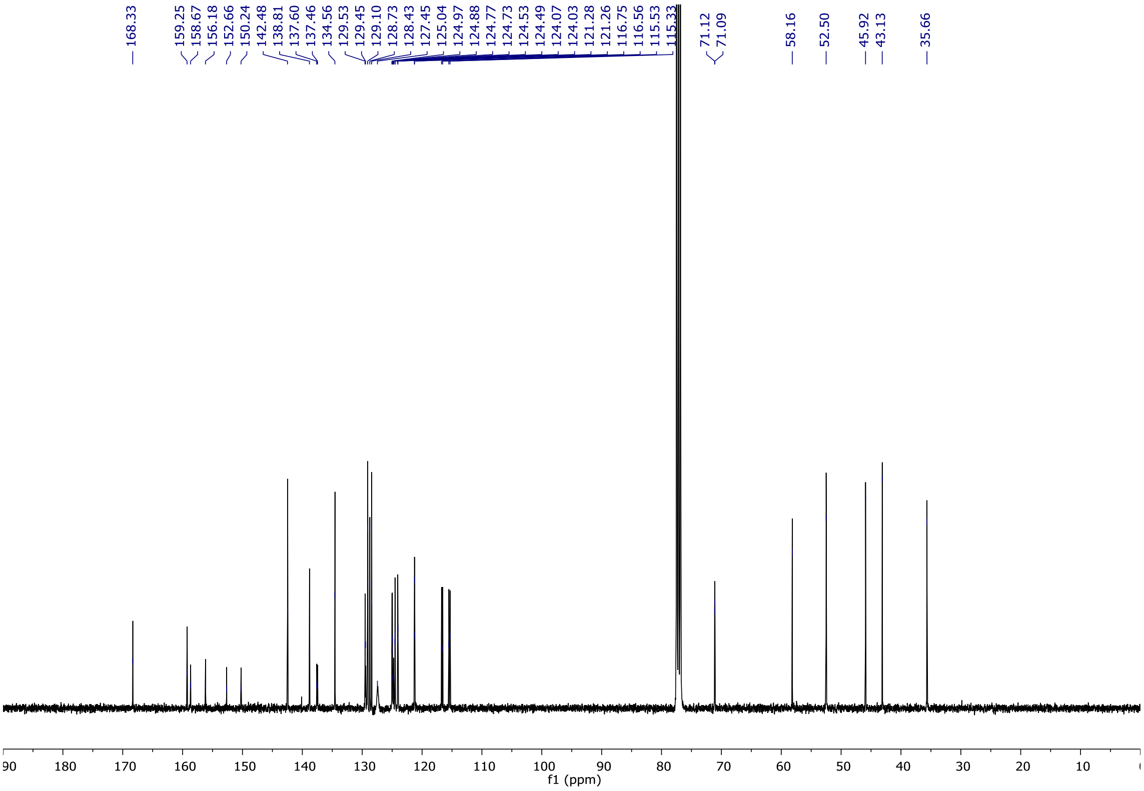

$^{19}F\{^{1}H\}$ NMR (282 MHz, $CDCl_3$)

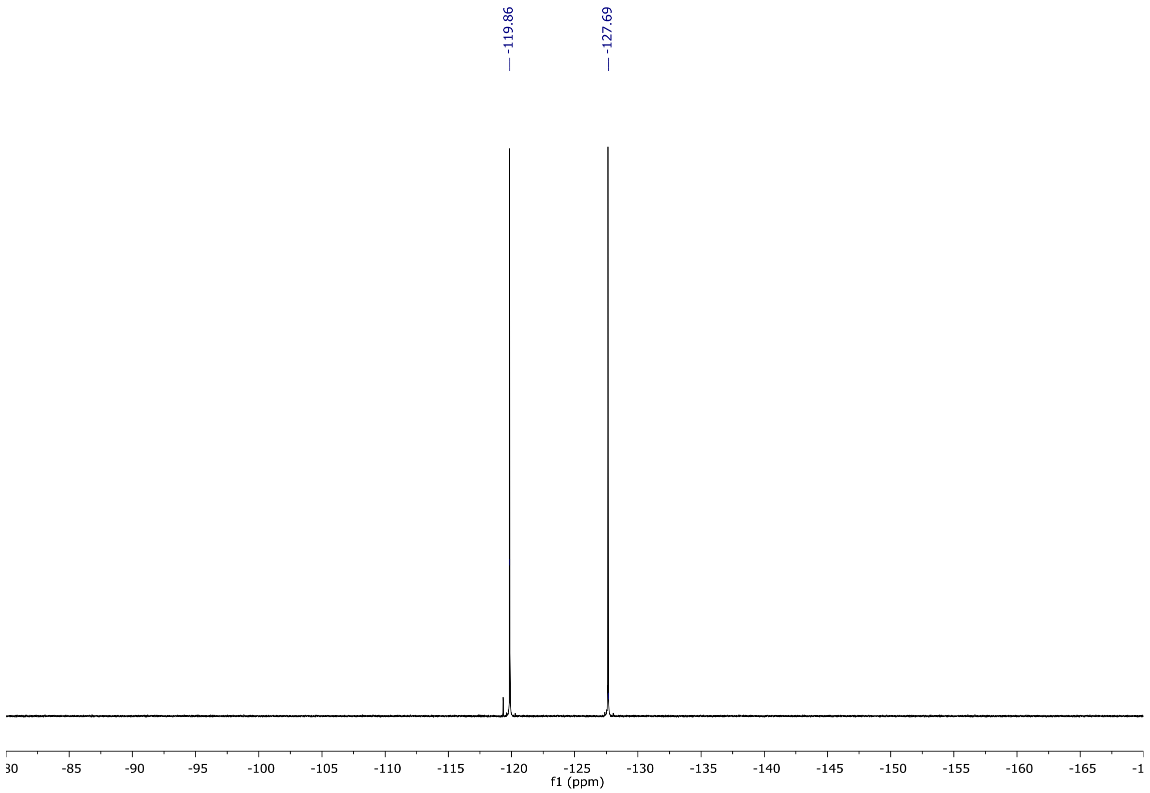

(1S*,2*R,2'*R**,4*S**,*Z*)-2'-Phenyl-1'-(3-(trifluoromethyl)phenyl)-4'-((3-(trifluoromethyl)phenyl)imino)spiro[bicyclo[2.2.1]heptane-2,3'-pyrrolidin]-5-en-5'-one (12i)**

^{1}H NMR (400 MHz, $CDCl_3$)

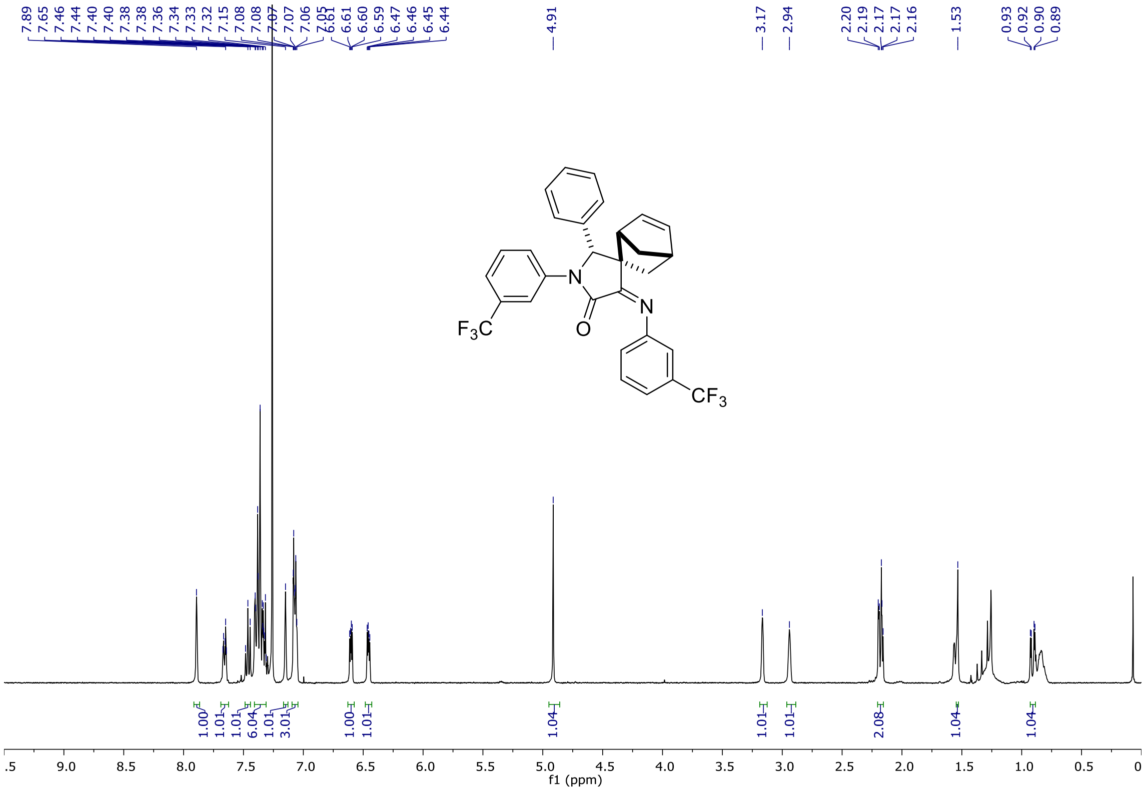

^{13}C {^{1}H} NMR (101 MHz, $CDCl_3$)

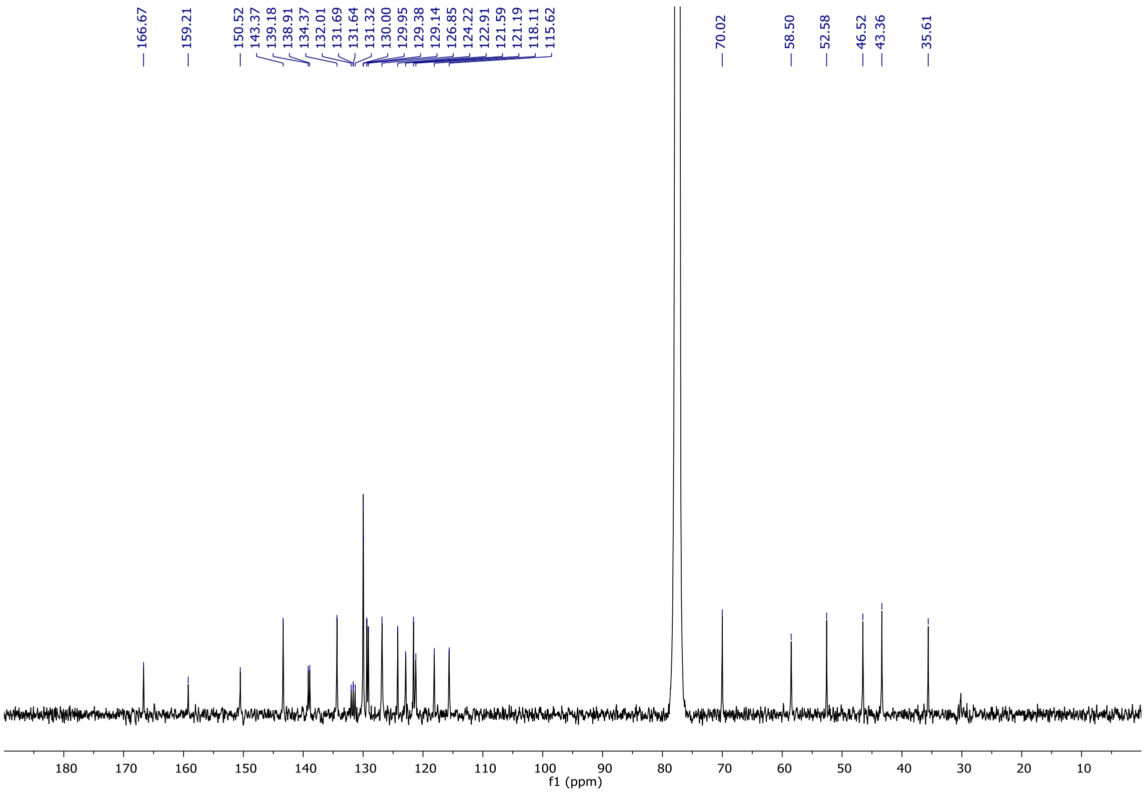

^{19}F {^{1}H} NMR (282 MHz, $CDCl_3$)

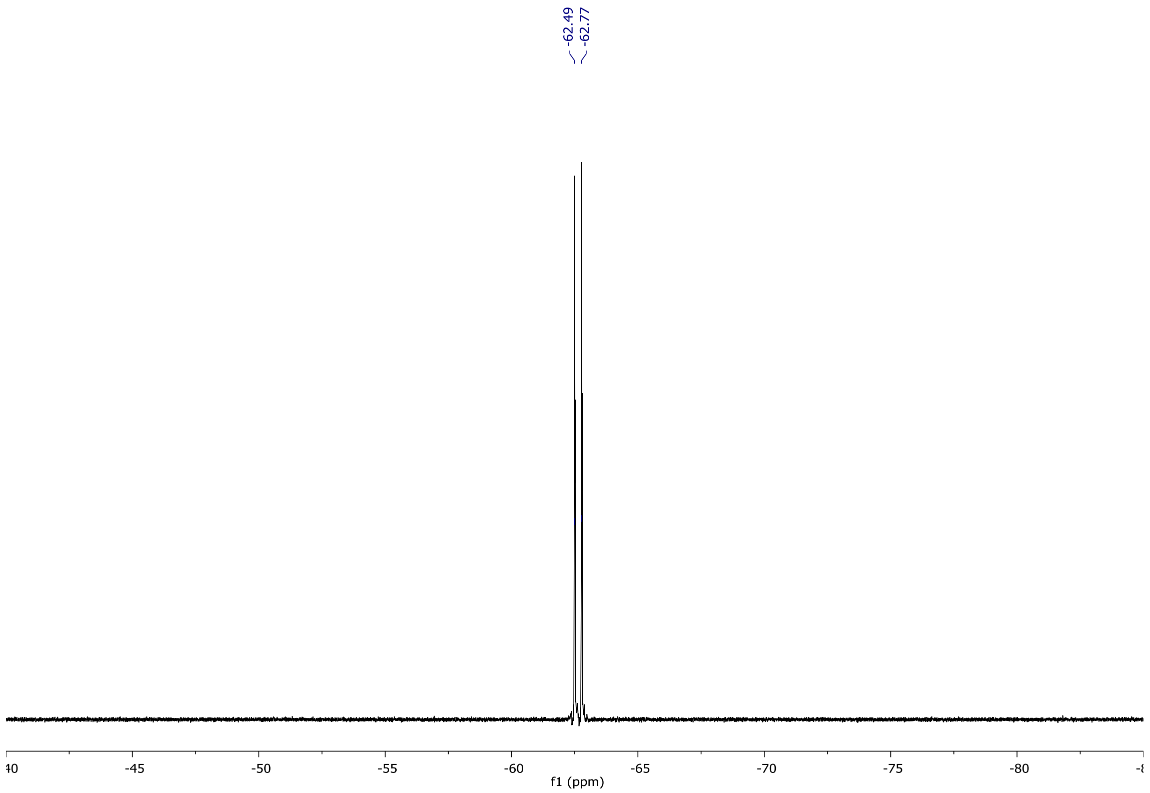

II. 2D NMR spectra copies of 9a, 5, 7, 12a and 12h

4-((Dimethylamino)methyl)-5-phenyl-1-(*p*-tolyl)-3-(*p*-tolylamino)-1,5-dihydro-2*H*-pyrrol-2-one (9a).

2D-COSY NMR {^{1}H – ^{1}H} (400 MHz, $CDCl_3$)

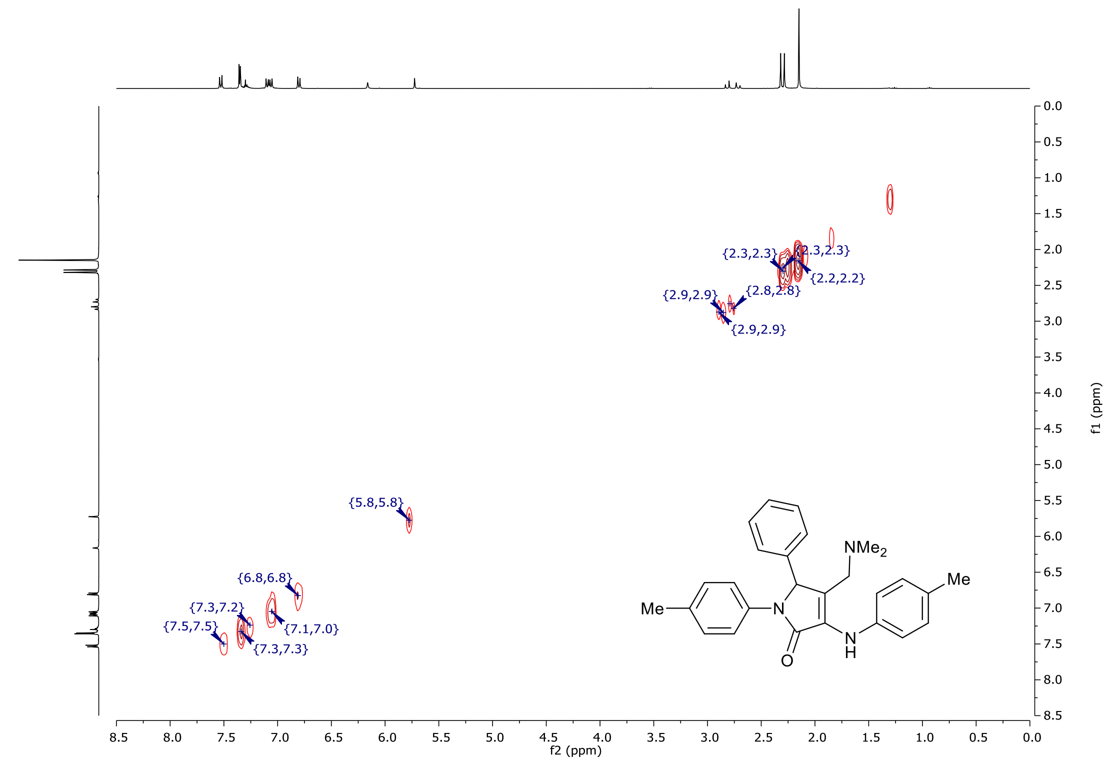

2D-HSQC NMR {^{1}H – ^{13}C} (^{1}H: 400 MHz, ^{13}C: 101 MHz, $CDCl_3$)

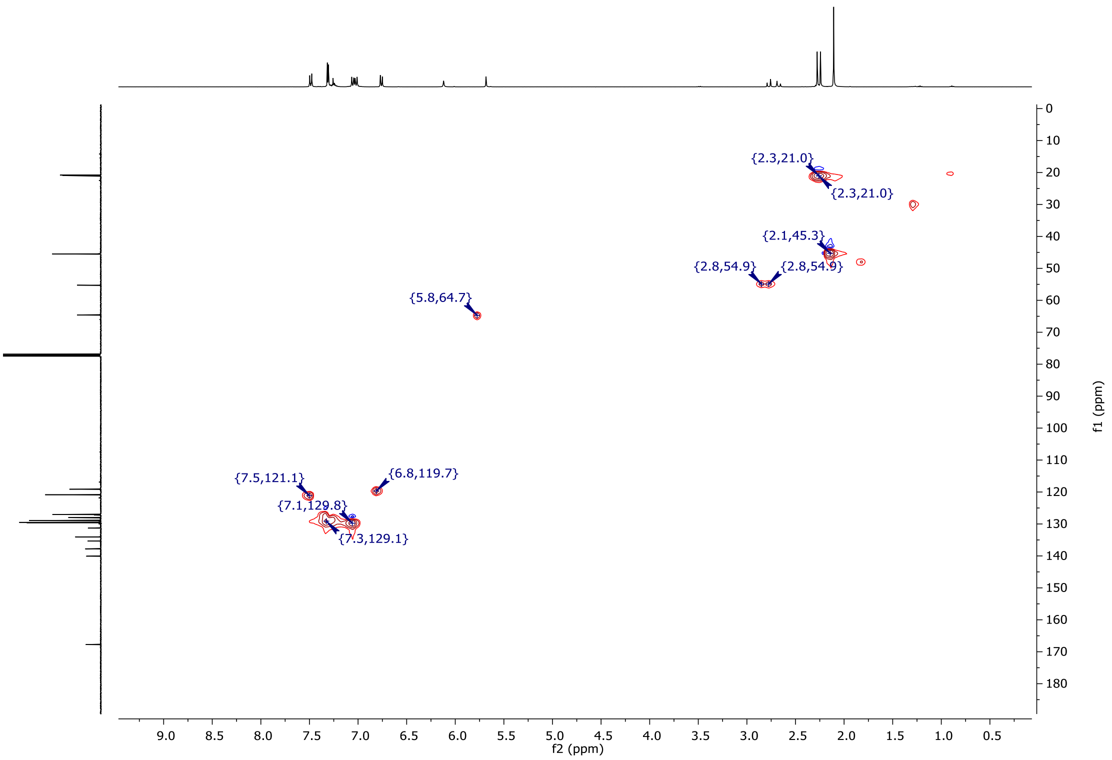

2D-HMBC NMR {^{1}H – ^{13}C} (^{1}H: 400 MHz, ^{13}C: 101 MHz, $CDCl_3$)

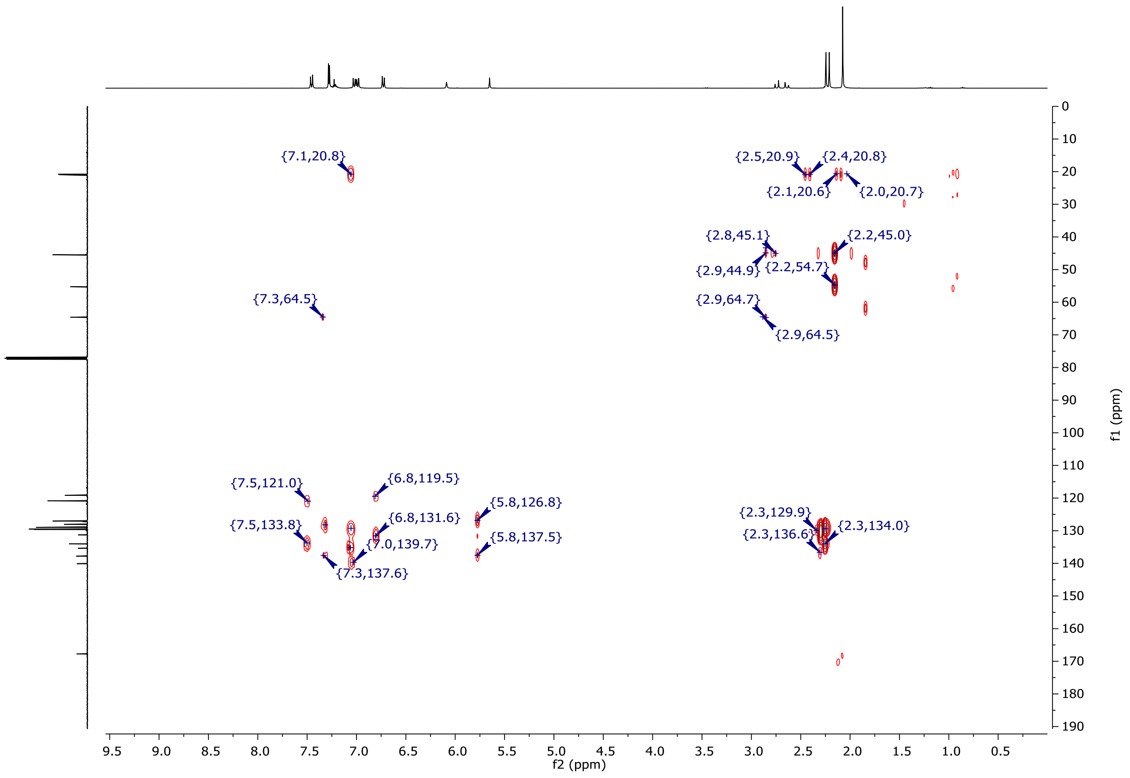

(*Z*)-1,1',6'-Tri-*p*-tolyl-4-(*p*-tolylimino)-3',4',5',6'-tetrahydrospiro[pyrrolidine-3,2'-pyrrolo[3,4-*b*]pyridine]-5,7'(1'*H*)-dione (5)

2D-COSY NMR {^{1}H – ^{1}H} (400 MHz, $CDCl_3$)

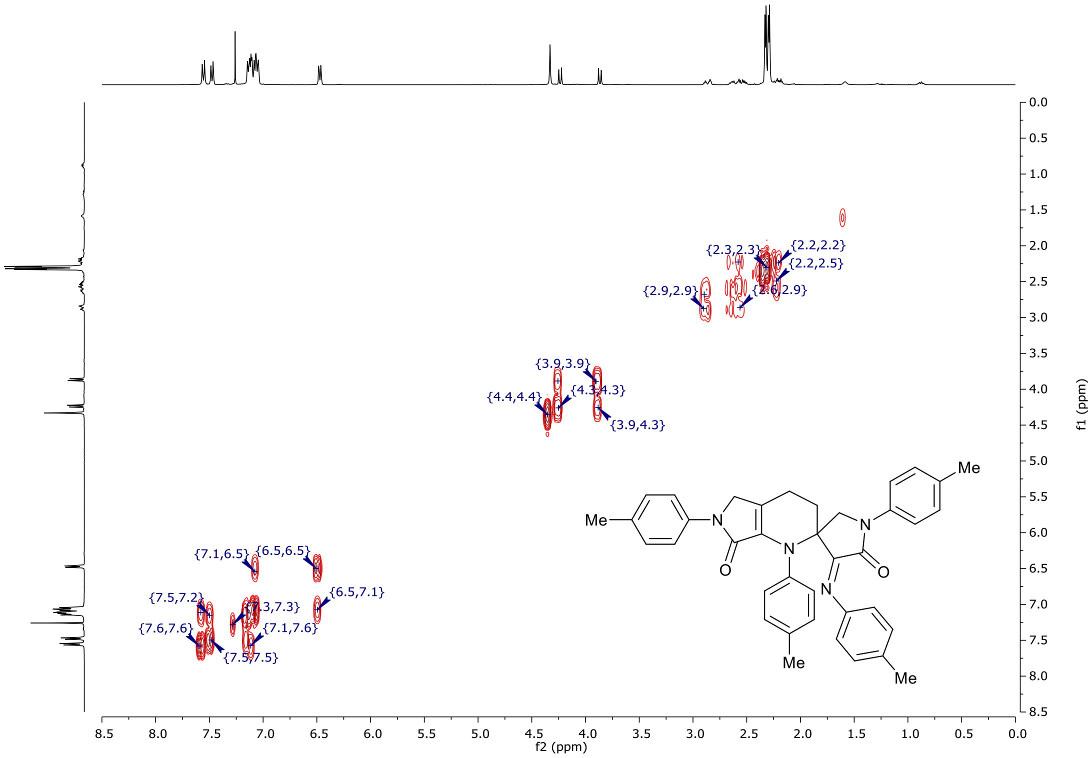

2D-HSQC NMR {^{1}H – ^{13}C} (^{1}H: 400 MHz, ^{13}C: 101 MHz, $CDCl_3$)

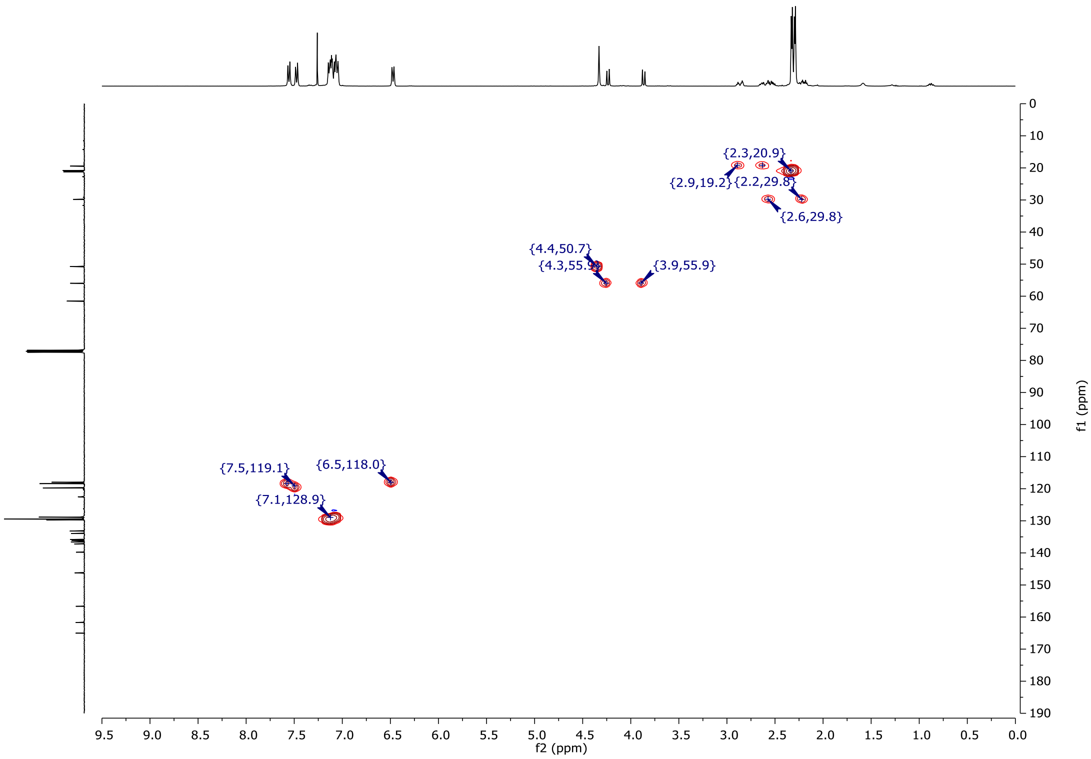

2D-HMBC NMR {^{1}H – ^{13}C} (^{1}H: 400 MHz, ^{13}C: 101 MHz, $CDCl_3$)

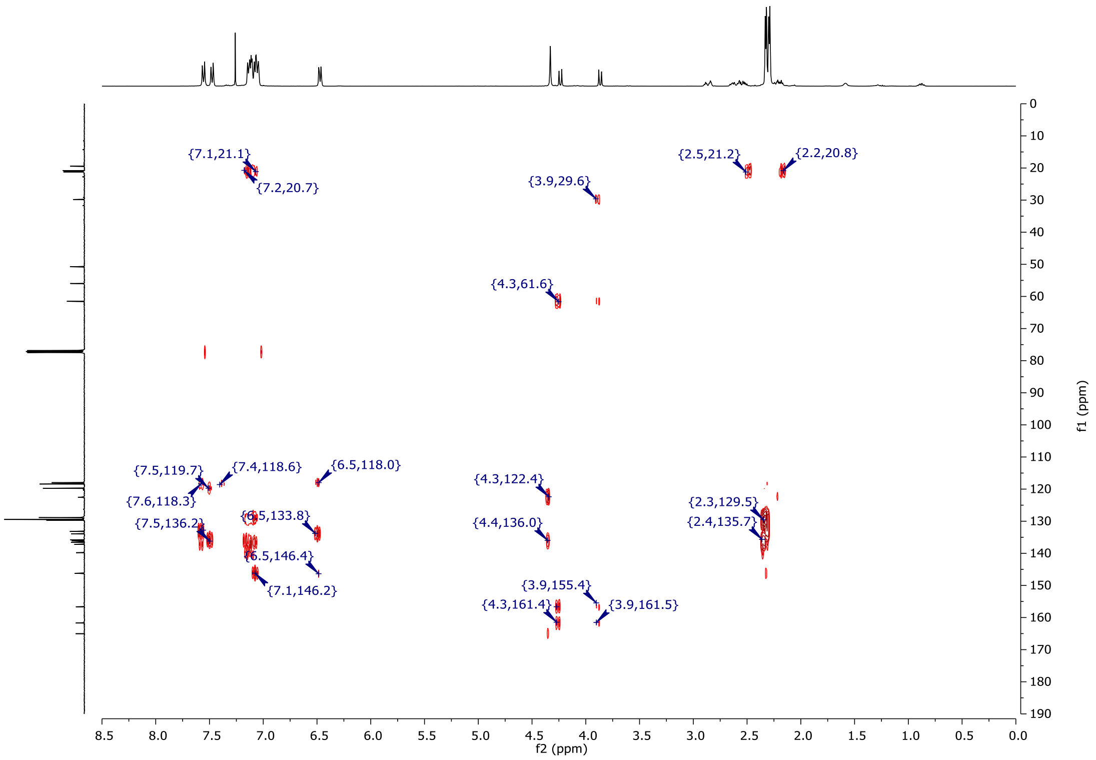

1,3,6-Tri-*p*-tolyl-1,2,3,4,5,6-hexahydro-7*H*-pyrrolo[3,4-*d*]pyrimidin-7-one (7).

2D-COSY NMR {^{1}H – ^{1}H} (400 MHz, $CDCl_3$)

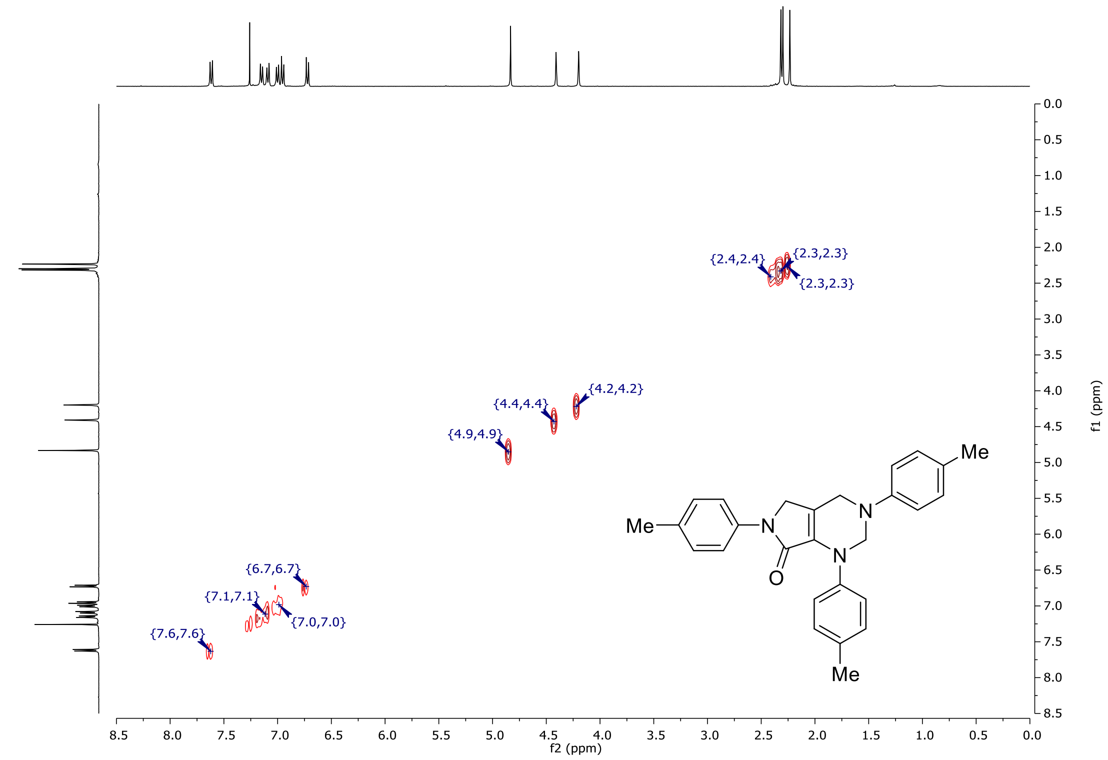

2D-HSQC NMR {^{1}H – ^{13}C} (^{1}H: 400 MHz, ^{13}C: 101 MHz, $CDCl_3$)

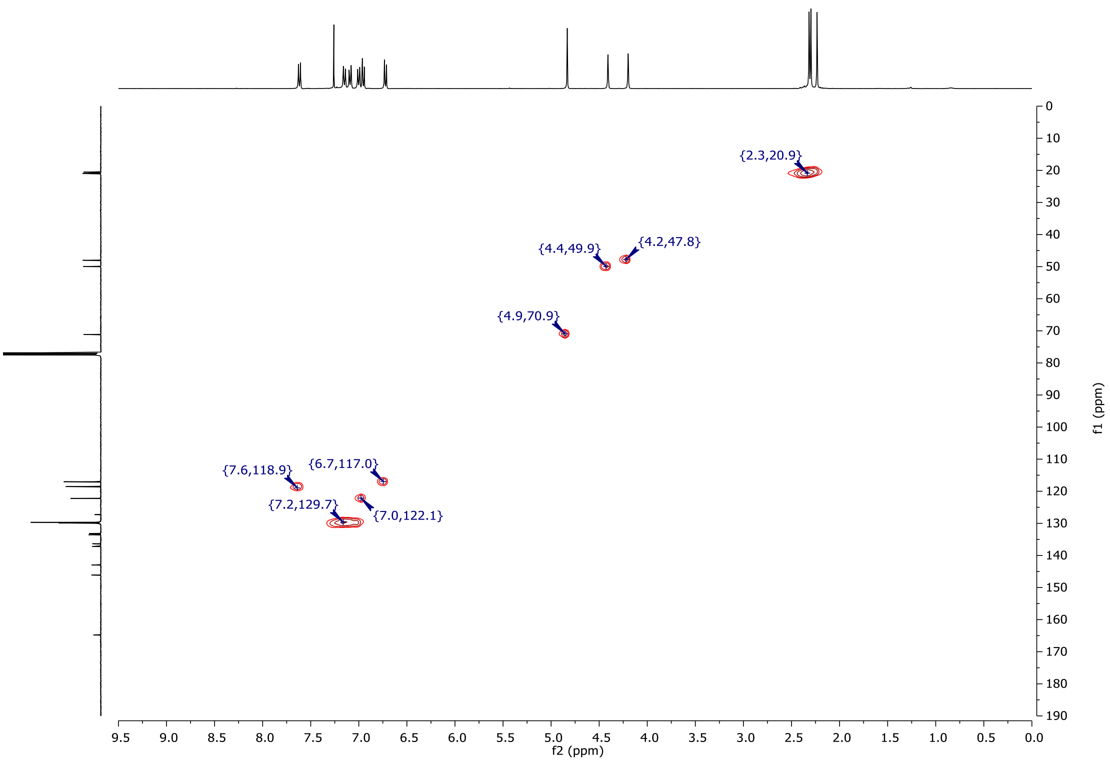

2D-HMBC NMR {^{1}H – ^{13}C} (^{1}H: 400 MHz, ^{13}C: 101 MHz, $CDCl_3$)

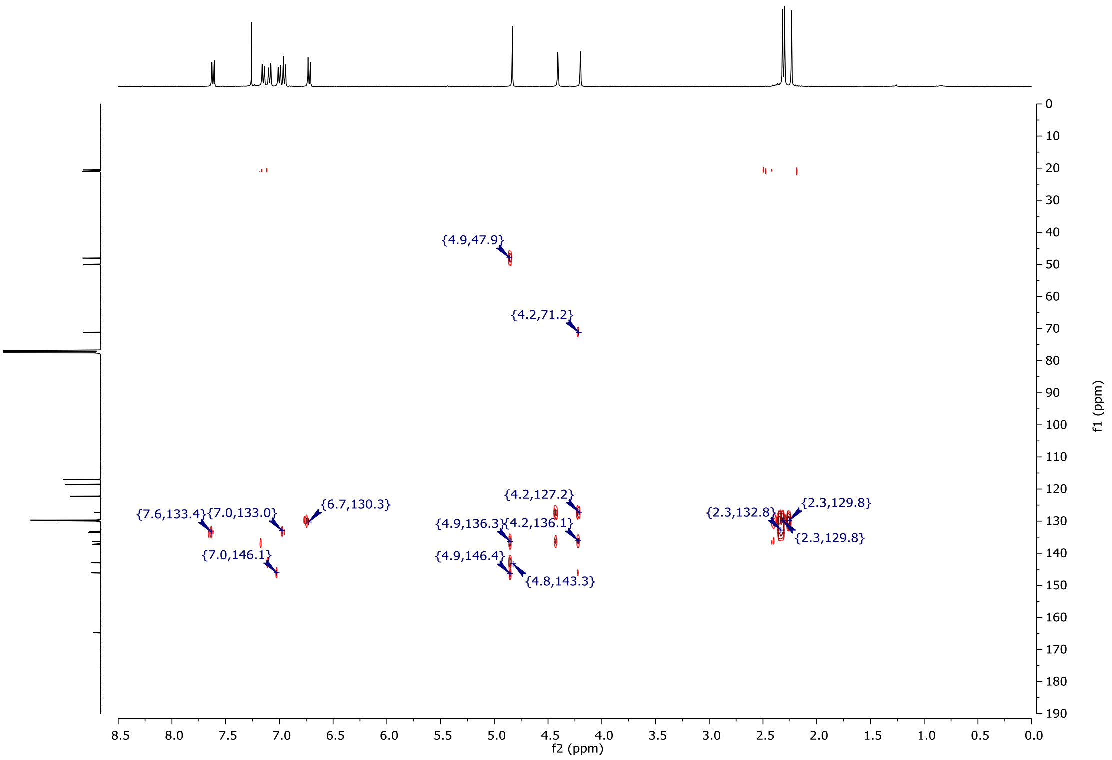

(1S*,2*R,2'*R**,4*S**,*Z*)-2'-Phenyl-1'-(*p*-tolyl)-4'-(*p*-tolylimino)spiro[bicyclo[2.2.1]heptane-2,3'-pyrrolidin]-5-en-5'-one (12a)**

2D-COSY NMR {^{1}H – ^{1}H} (400 MHz, $CDCl_3$)

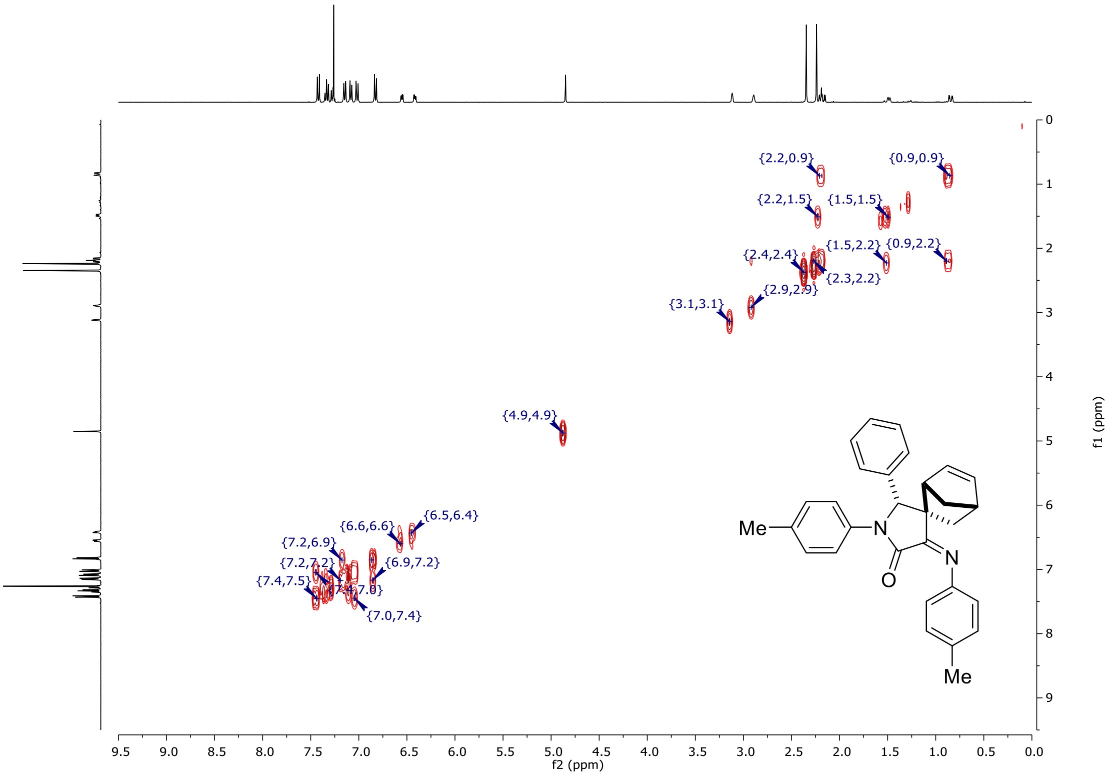

2D-HSQC NMR {^{1}H – ^{13}C} (^{1}H: 400 MHz, ^{13}C: 101 MHz, $CDCl_3$)

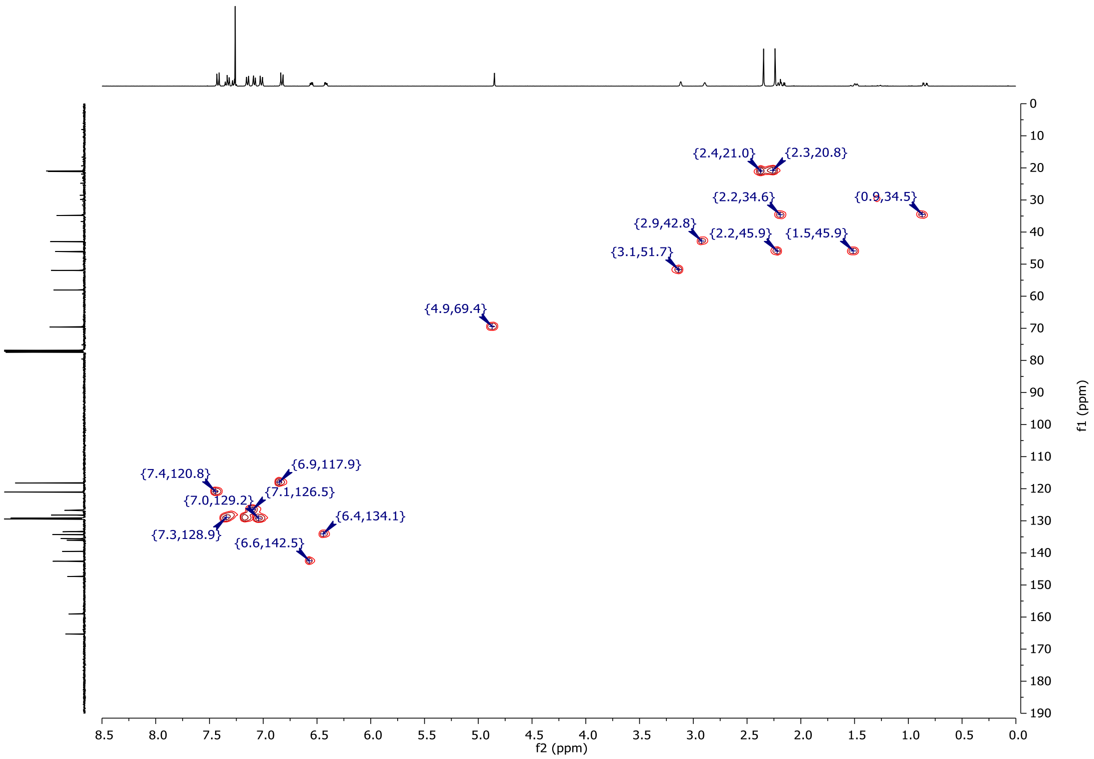

2D-HMBC NMR {^{1}H – ^{13}C} (^{1}H: 400 MHz, ^{13}C: 101 MHz, $CDCl_3$)

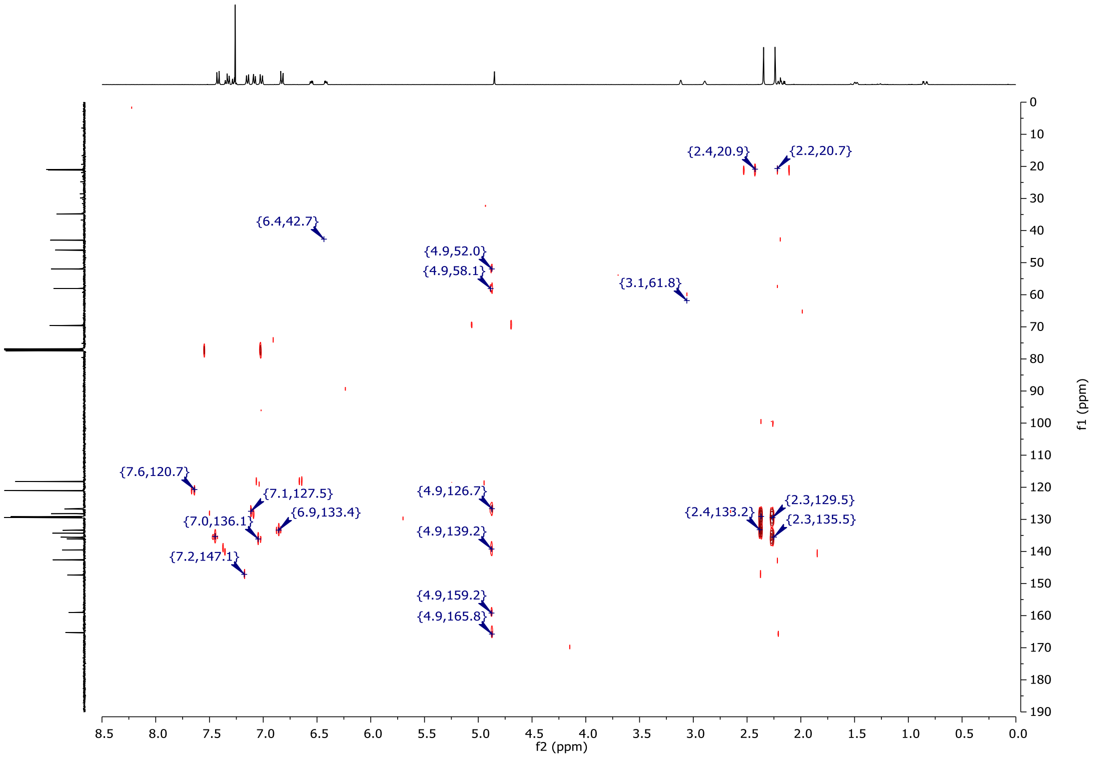

(1S*,2R*,2'R*,4S*,Z)-1'-(2-Fluorophenyl)-4'-((2-fluorophenyl)imino)-2'-phenylspiro[bicyclo[2.2.1]heptane-2,3'-pyrrolidin]-5-en-5'-one (12h)

2D-COSY NMR {^{1}H – ^{1}H} (400 MHz, $CDCl_3$)

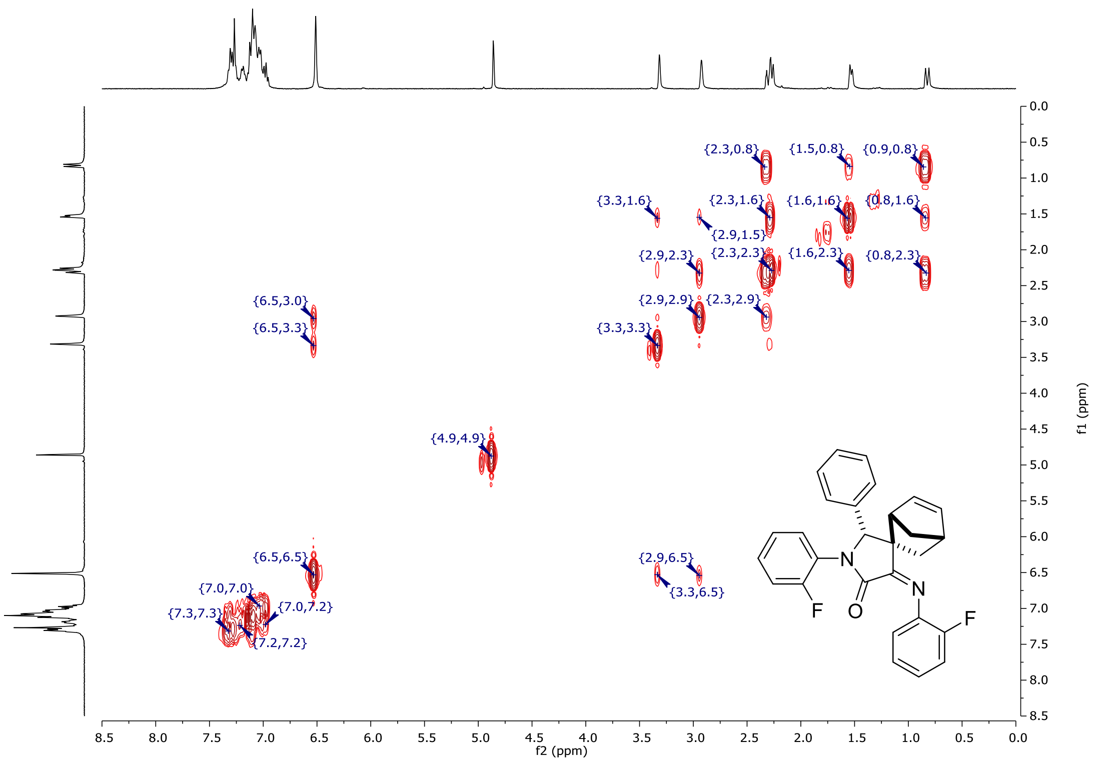

2D-HSQC NMR {^{1}H – ^{13}C} (^{1}H: 400 MHz, ^{13}C: 101 MHz, $CDCl_3$)

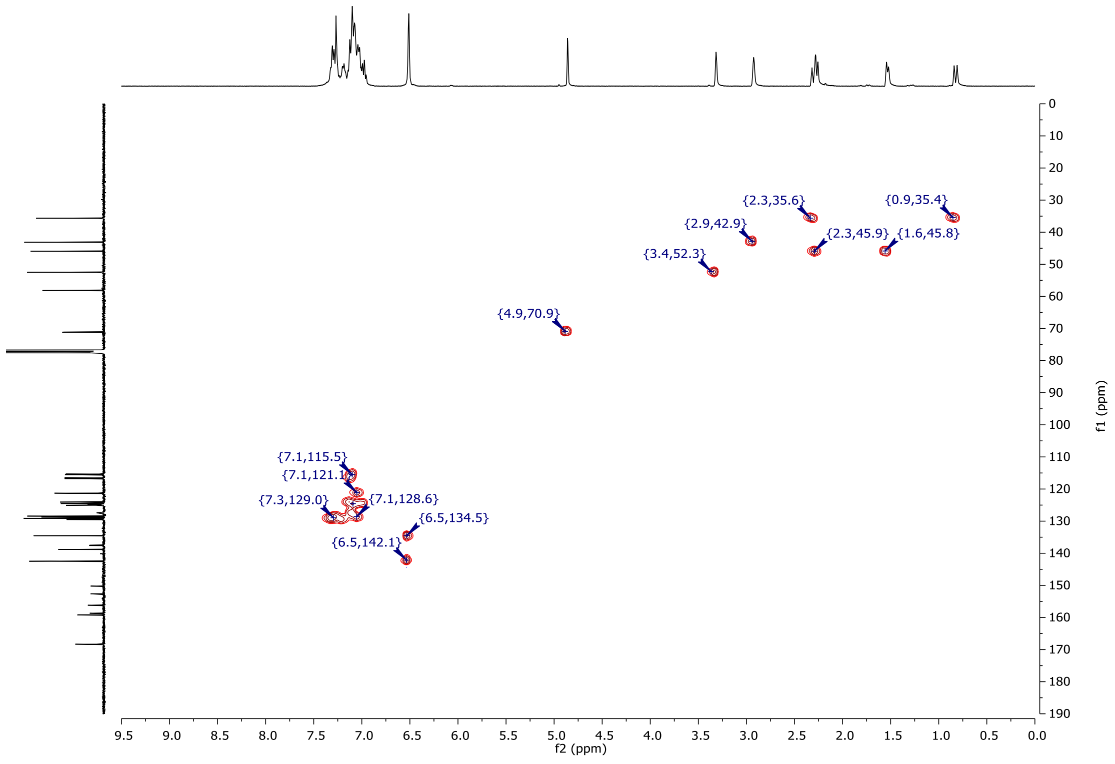

2D-HMBC NMR {^{1}H – ^{13}C} (^{1}H: 400 MHz, ^{13}C: 101 MHz, $CDCl_3$)

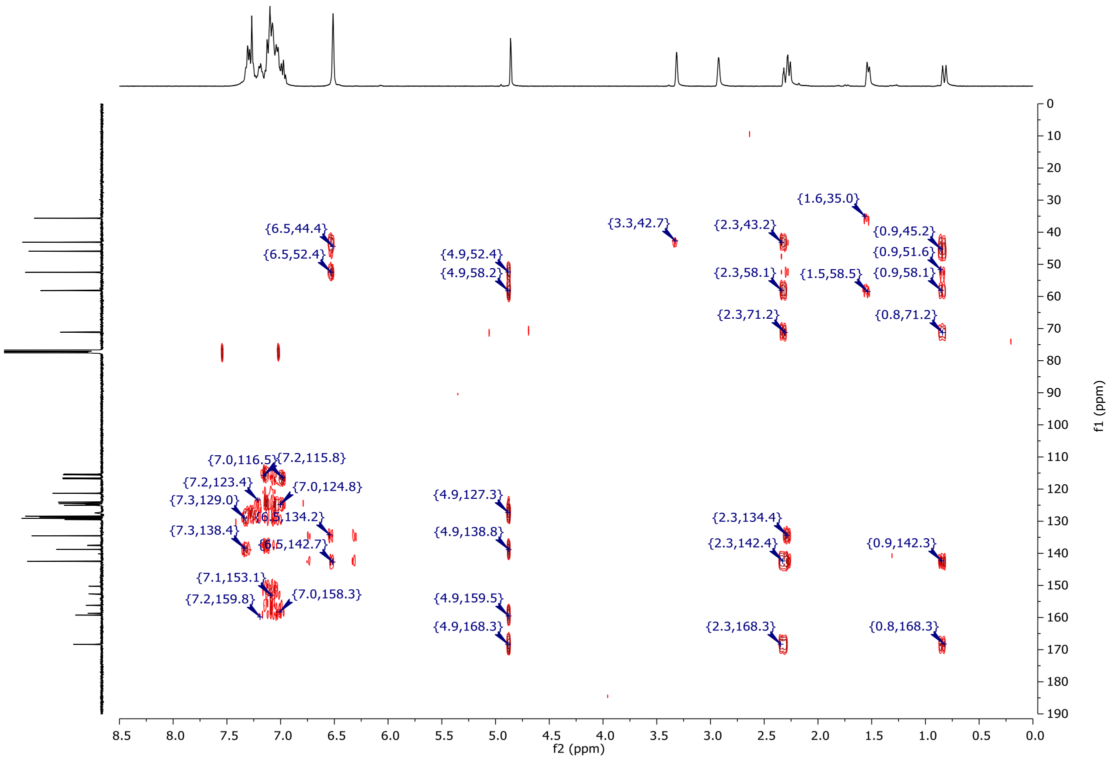

III. Crystallography of 12d and 12f

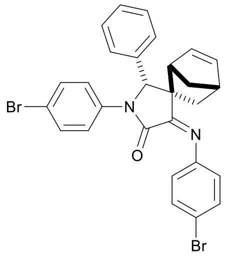

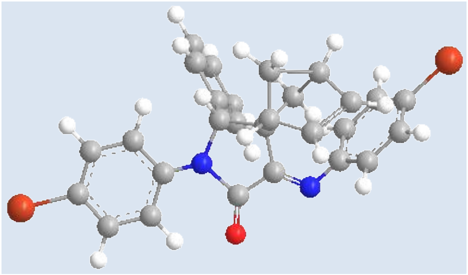

The structure of compound **12d** (b20220104_AL468DUCu2C) was determined on a crystal prepared from a $CDCl_3$ solvent system by slow evaporation in a vial at room temperature. The X-ray data have been deposited at the Cambridge Crystallographic Data Centre (CCDC 2257539).

Intensity data were collected on an Agilent Technologies Super-Nova diffractometer, wich was equipped with monochromated Cu kα radiation (λ= 1.54184 Å) and de HyPix-6000H Hybrid Pixel Array Detecto. Measurement was carried out at 151.0 (6) K with the help of an Oxford Cryostream 700 PLUS temperature device. Data frames were processed (united cell determination, analytical absorption correction with face indexing, intensity data integration and correction for Lorentz and polarization effects) using the Crysalis software package.[2] The structure was solved using SHELXT[3] and refined by full-matrix least-squares with SHELXL-97.[4] Final geometrical calculations were carried out with Mercury[5]. and PLATON[6] as integrated in WinGX.[7] and Olex 2.

Experimental

Single crystals of **12d**. A suitable crystal was selected and monted on a SuperNova, Dual, Cu at home/near, HyPix diffractometer. The crystal was kept at 170.15 K during data collection. Using Olex2,[8] the structure was solved with the SHELXT[3] structure solution program using Intrinsic Phasing and refined with the SHELXL[4]

---

[1] CrysAlisPro, Agilent Technologies, Version 1.171.37.31 (release 14-01-2014 CrysAlis171 .NET) (compiled Jan 14 2014,18:38:05).

[3] G. M. Sheldrick. SHELXT - Integrated space-group and crystal-structure determination. *Acta Cryst.*, 2015, **A71**, 3-8. https://doi.org/10.1107/S2053273314026370.

[4] a). G. M. Sheldrick. A short history of SHELX. *Acta Cryst.*, 2008, **A64**, 112-122. https://doi.org/10.1107/S0108767307043930 b). G. M. Sheldrick. Crystal structure refinement with *SHELXL*. *Acta Cryst.*, 2015, **C71**, 3-8. https://doi.org/10.1107/S2053229614024218.

[5] C. F. Macrae. Mercury CSD 2.0 – new features for the visualization and investigation of crystal structures. *J. Appl. Crystallogr.*, 2008, **41**, 466-470. https://doi.org/10.1107/S0021889807067908.

[6] A. L. Spek. Single-crystal structure validation with the program *PLATON*. *J. Appl. Cryst.*, 2003, **36**, 7-13. https://doi.org/10.1107/S0021889802022112.

[7] L. J. Farrugia. *WinGX* suite for small-molecule single-crystal crystallography. *J. Appl. Cryst.*, 1999, **32**, 837-838. https://doi.org/10.1107/S0021889899006020.

[8] O. V. Dolomanov, L. J. Bourhis, R. J. Gildea, J. A. K. Howard and H. Puschmann. OLEX2: A Complete Structure Solution, Refinement and Analysis Program. *J. Appl. Cryst.*, 2009, **42**, 339-341. https://doi.org/10.1107/S0021889808042726.

refinement package using Least Squares minimisation.

Crystal structure determination of 12d

Crystal Data for $C_{28}H_{22}Br_2N_2O$ (M =562.29 g/mol): monoclinic, space group P21/c (no. 14), a = 19.3417(5) Å, b = 14.1612(2) Å, c = 19.2633(6) Å, β = 117.841(4)°, V = 4665.5(2) Å3, Z = 8, T = 170.15 K, μ(CuKα) = 4.595 mm-1, $Dcalc$ = 1.601 g/cm^3, 84358 reflections measured (5.166° ≤ 2Θ ≤ 137.992°), 8665 unique (Rint = 0.1103, Rsigma = 0.0390) which were used in all calculations. The final R1 was 0.1311 (I > 2σ(I)) and wR2 was 0.3307 (all data).

Thermal ellipsoid plot/ORTEP for compound 12d. Contour probability level: 50%

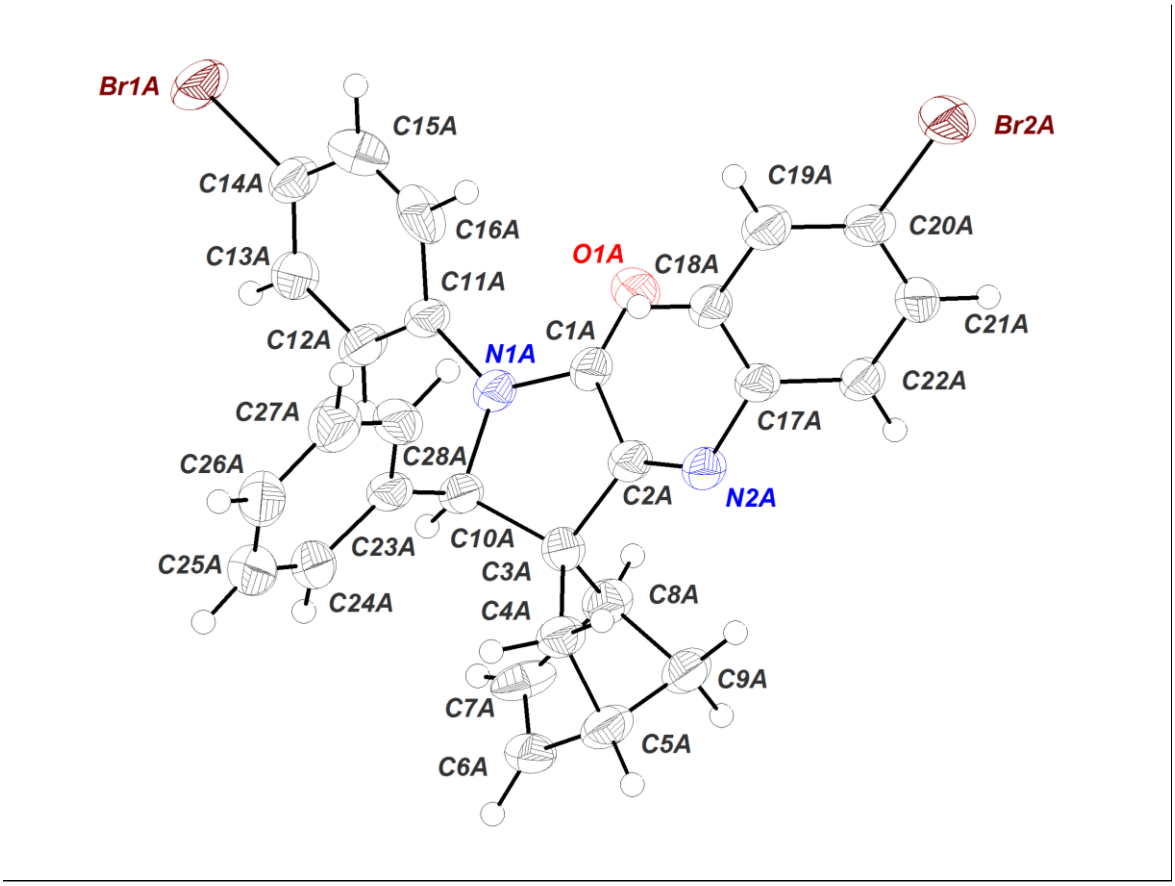

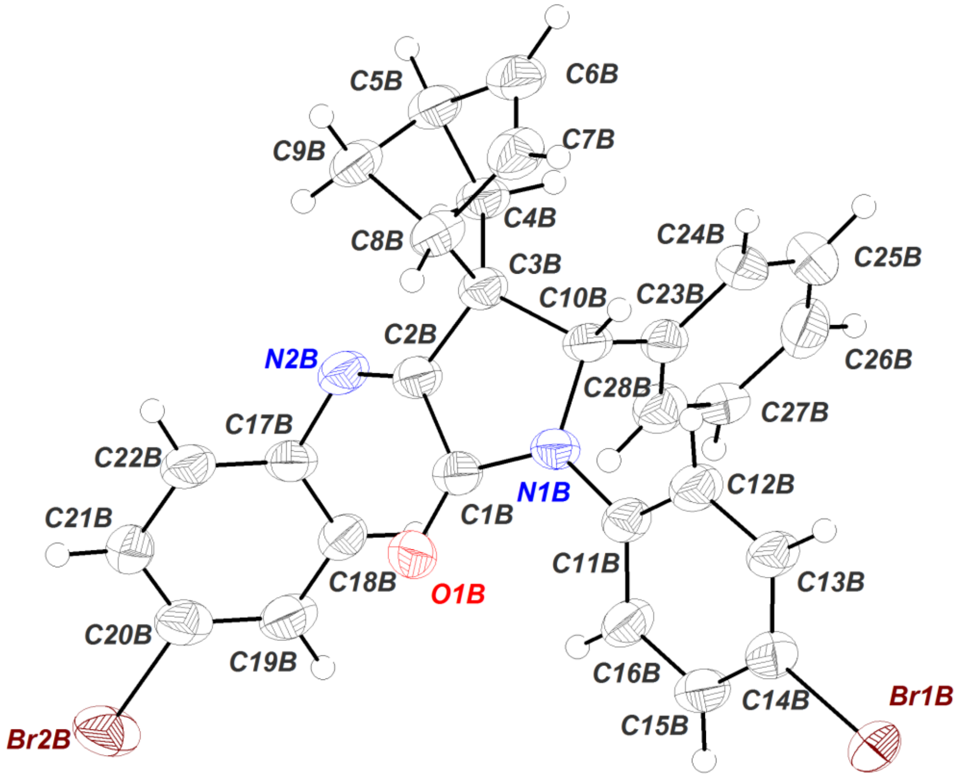

Table S1. Crystal data and structure refinement for 12d.

Identification code	b20220104_AL468DUCu2C
Empirical formula	$C_{28}H_{22}Br_2N_2O$
Formula weight	562.29
Temperature/K	170.01(10)
Crystal system	monoclinic
Space group	P21/c
a/Å	19.3417(5)
b/Å	14.1612(2)
c/Å	19.2633(6)
α/°	90.0
β/°	117.841(4)
γ/°	90.0
Volume/Å^3	4665.5(2)
Z	8
ρcalcg/cm^3	1.601
μ/mm^{-1}	4.595
F(000)	2256.0
Crystal size/mm^3	0.284 × 0.241 × 0.208
Radiation	CuKα (λ = 1.54184)

2Θ range for data collection/°	5.166 to 137.992
Index ranges	-23 ≤ h ≤ 23, -17 ≤ k ≤ 17, -23 ≤ l ≤ 22
Reflections collected	84358
Independent reflections	8665 [Rint = 0.1103, Rsigma = 0.0390]
Data/restraints/parameters	8665/12/595
Goodness-of-fit on F2	1.446
Final R indexes [I>=2σ (I)]	R1 = 0.1311, wR2 = 0.3125
Final R indexes [all data]	R1 = 0.1408, wR2 = 0.3307
Largest diff. peak/hole / e $Å^{-3}$	6.07/-0.73

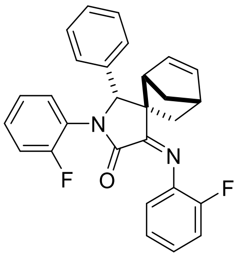

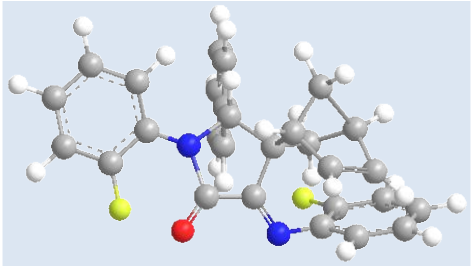

The structure of compound **12f** (b20220042_AL515DUCu2T170) was determined on a crystal prepared from a $CDCl_3$ solvent system by slow evaporation in a vial at room temperature. The X-ray data have been deposited at the Cambridge Crystallographic Data Centre (CCDC 2257537).

Intensity data were collected on an Agilent Technologies Super-Nova diffractometer, wich was equipped with monochromated Cu kα radiation (λ= 1.54184 Å) and de HyPix-6000H Hybrid Pixel Array Detecto. Measurement was carried out at 151.0 (6) K with the help of an Oxford Cryostream 700 PLUS temperature device. Data frames were processed (united cell determination, analytical absorption correction with face indexing, intensity data integration and correction for Lorentz and polarization effects) using the Crysalis software package.[2] The structure was solved using SHELXT[3] and refined by full-matrix least-squares with SHELXL-97.[4] Final geometrical calculations were carried out with Mercury[5]. and PLATON[6] as integrated in WinGX.[7] and Olex 2. The asymmetric unit has two crystallization water molecules.

Experimental

Single crystal of **12h**. A suitable crystal was selected and mounted on a SuperNova, Dual, Cu at home/near, HyPix diffractometer. The crystal was kept at 170.0(1) K during data collection. Using Olex2,[8] the structure was solved with the SHELXT[3] structure solution program using Intrinsic Phasing and refined with the SHELXL[4] refinement package using Least Squares minimisation.

Crystal structure determination of 12h.

Crystal Data for $C_{28}H_{22}F_2N_2O$ (*M* =440.47 g/mol): orthorhombic, space group Pbca (no. 61), *a* = 12.79692(13) Å, *b* = 12.42625(13) Å, *c* = 27.4810(4) Å, *V* = 4369.96(9) Å3, *Z* = 8, *T* = 231(80) K, μ(CuKα) = 0.764 mm-1, *Dcalc* = 1.339 g/cm^3, 27946 reflections measured (6.432° ≤ 2Θ ≤ 137.99°), 4068 unique (*R*int = 0.0361, Rsigma = 0.0274) which were used in all calculations. The final *R*1 was 0.0479 (I > 2σ(I)) and *wR*2 was 0.1451 (all data).

Thermal ellipsoid plot/ORTEP for compound 12h. Contour probability level: 50%

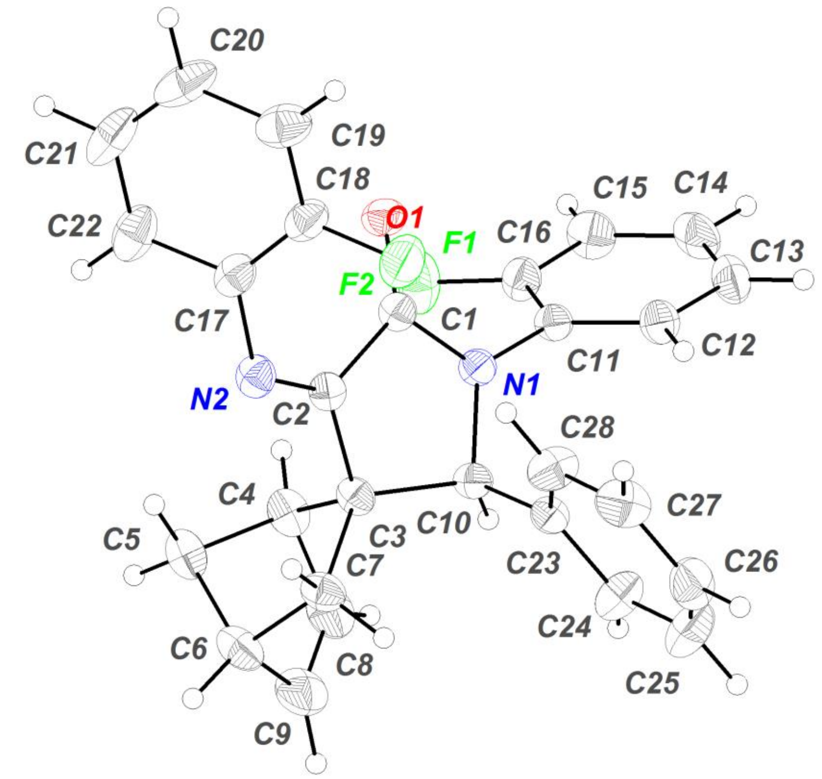

Table S1. Crystal data and structure refinement for 12h.

Identification code	b20220042_AL515DUCu2T170
Empirical formula	$C_{28}H_{22}F_2N_2O$
Formula weight	440.47
Temperature/K	170.0(1)
Crystal system	orthorhombic
Space group	Pbca
a/Å	12.79692(13)
b/Å	12.42625(13)
c/Å	27.4810(4)

α/°	90.0
β/°	90.0
γ/°	90.0
Volume/Å^3	4369.96(9)
Z	8
ρcalcg/cm^3	1.339
μ/mm^{-1}	0.764
F(000)	1840.0
Crystal size/mm^3	0.32 × 0.18 × 0.05
Radiation	CuKα (λ = 1.54184)
2Θ range for data collection/°	6.432 to 137.99
Index ranges	-10 ≤ h ≤ 15, -15 ≤ k ≤ 15, -33 ≤ l ≤ 32
Reflections collected	27946
Independent reflections	4068 [Rint = 0.0361, Rsigma = 0.0274]
Data/restraints/parameters	4068/0/298
Goodness-of-fit on F2	1.071
Final R indexes [I>=2σ (I)]	R1 = 0.0479, wR2 = 0.1381
Final R indexes [all data]	R1 = 0.0552, wR2 = 0.1451
Largest diff. peak/hole / e Å^{-3}	1.26/-0.47

IV. Computational methods

Table S1. Total electronic energies[a] (E, in a.u.), zero point correction of the energy[b] (ZPCE), thermal corrections to Gibbs free energies[b] (TCGFE, in a.u.), and number of imaginary frequencies[c] (NIMAG) of all stationary points discussed in the main text.

Structure	E	ZPCE	TCGFE	NIMAG(ν)
***R*-10d**	-6443.0699422	0.402704	0.337732	0
***S*-10d**	-6443.0612533	0.402655	0.337078	0
**CpH**	-194.0213814	0.093831	0.067242	0
**10d·10d**	-12886.1945572	0.807344	0.709917	0
**10d·CpH**	-6637.123027	0.497903	0.424356	0
**10d·CpH´**	-6637.0989018	0.498359	0.426711	0
**10d·CpH´´**	-6637.0954182	0.497190	0.425242	0
***R*-11d**	-6214.028288	0.334777	0.275856	0
***S*-11d**	-6214.028288	0.334780	0.275867	0
**RC11d·11d**	-12428.109777	0.671122	0.581642	0
**TS11d·11d**	-12428.090668	0.672722	0.588063	1 (-421.6719)
**PROD11d·11d**	-12428.138629	0.677417	0.592482	0
**RC1d·*exo***	-6408.063223	0.430023	0.362984	0
**TS1d·*exo***	-6408.043623	0.431418	0.367648	1 (-443.4828)
**PROD1d·*exo***	-6408.0987395	0.436738	0.375038	0
**RC1d·*exo´***	-6408.060806	0.429907	0.362740	0
**TS1d·*exo´***	-6408.032593	0.431588	0.369573	1 (-443.6333)
**PROD1d·*exo´***	-6408.093780	0.436510	0.375220	0
**RC1d·*endo***	-6408.061981	0.429953	0.363740	0
**TS1d·*endo***	-6408.040260	0.431631	0.370231	1 (-427.3171)
**PROD1d·*endo***	-6408.116630	0.436928	0.375019	0
**RC2d·*endo***	-6408.0632672	0.429763	0.362851	0
**TS2d·*endo***	-6408.0395404	0.431583	0.368897	1 (-417.5435)
**PROD2d·*endo***	-6408.0977928	0.436434	0.374623	0
**RC2d·*exo***	-6408.0577188	0.429646	0.361261	0
**TS2d·*exo***	-6408.0321117	0.430887	0.367034	1 (-464.0475)
**PROD2d·*exo***	-6408.1186929	0.437031	0.374892	0
**RC2d·*endo´***	-6408.06198	0.429954	0.363746	0
**TS2d·*endo´***	-6408.040258	0.431628	0.37022	1 (-427.2743)
**PROD2d·*endo´***	-6408.116631	0.436925	0.375006	0
**RC2d·*exo´***	-6408.060806	0.429903	0.362726	0
**TS2d·*exo´***	-6408.028738	0.431421	0.369358	1 (-494.5263)
**PROD2d·*exo´***	-6408.119399	0.437140	0.375051	0
**RC2d·*endo´´***	-6408.064601	0.429826	0.362994	0
**TS2d·*endo´´***	-6408.026862	0.43134	0.368819	1 (-486.9923)
**PROD2d·*endo´´***	-6408.116143	0.436598	0.373730	0
**RC2d·*exo´´***	-6408.057974	0.429446	0.360501	0
**TS2d·*exo´´***	-6408.026124	0.431025	0.368409	1 (-497.1488)
**PROD2d·*exo´´***	-6408.119847	0.437015	0.374849	0
**RC2d·*endo´´´***	-6408.060945	0.429880	0.363186	0

**TS2d·*endo*´´´**	-6408.027654	0.431709	0.370653	1 (-488.0784)
**PROD2d·*endo*´´´**	-6408.115807	0.436700	0.373670	0

[a]Computed at M06-2X-GD3 (PCM)/6-31+G**//M06-2X-GD3 (PCM)/6-31G* level of theory. [b]Computed at 298.15 K at M06-2X-GD3 (PCM)/6-31G* level of theory. [c] If NIMAGE is not zero, imaginary frequency is in cm^{-1}.

Cartesian coordinates (optimized at the M06-2X-GD3 (PCM)/6-31G* level) of all the stationary points collected in the main text.

***R*-10d**

Center Number	Atomic Number	Atomic Type	Coordinates (Angstroms) X	Y	Z
1	6	0	1.653252	-2.327380	0.646816
2	6	0	1.832322	-1.190617	-0.154498
3	6	0	2.998390	-1.077675	-0.923642
4	6	0	3.964130	-2.076058	-0.898645
5	6	0	3.767172	-3.197955	-0.104960
6	6	0	2.618737	-3.327284	0.664902
7	7	0	0.874462	-0.156293	-0.197828
8	6	0	-0.414300	-0.215393	0.299825
9	6	0	-1.040037	1.112201	-0.011729
10	6	0	-0.138490	1.892187	-0.626962
11	6	0	1.170070	1.155747	-0.771055
12	6	0	-0.305941	3.300324	-1.086459
13	8	0	0.225432	4.188120	-0.088423
14	6	0	1.172142	5.069590	-0.477335
15	8	0	1.510670	5.229593	-1.624610
16	7	0	-2.302376	1.436087	0.441519
17	6	0	-3.446837	0.642151	0.294208
18	6	0	-4.595524	0.982028	1.017685
19	6	0	-5.760629	0.238178	0.885118
20	6	0	-5.774601	-0.860597	0.034070
21	6	0	-4.643731	-1.212101	-0.693570
22	6	0	-3.485718	-0.454011	-0.574101
23	8	0	-0.940972	-1.142515	0.883479
24	6	0	2.318882	1.869069	-0.073267
25	6	0	3.219108	2.627872	-0.818766
26	6	0	4.251095	3.319202	-0.184180
27	6	0	4.393792	3.240488	1.198188
28	6	0	3.492076	2.482213	1.947101
29	6	0	2.454099	1.805591	1.314938
30	35	0	-7.360479	-1.886987	-0.142802
31	35	0	5.082141	-4.563138	-0.069784
32	6	0	1.733697	5.789789	0.715458
33	1	0	0.213772	3.476096	-2.031310
34	1	0	-1.364813	3.541541	-1.214671
35	1	0	1.407645	1.054868	-1.839351
36	1	0	3.112614	2.684927	-1.899854
37	1	0	4.945206	3.909634	-0.773702
38	1	0	5.203462	3.767482	1.693067
39	1	0	3.599278	2.417470	3.025316
40	1	0	1.753211	1.213118	1.897934
41	1	0	3.170960	-0.208372	-1.546708
42	1	0	4.863168	-1.977493	-1.496435
43	1	0	2.474197	-4.204882	1.285116
44	1	0	0.762353	-2.430595	1.248333
45	1	0	-2.613966	-0.715434	-1.163553
46	1	0	-4.669124	-2.064658	-1.363170
47	1	0	-6.648347	0.506612	1.446766
48	1	0	-4.571209	1.830359	1.695569
49	1	0	2.206564	5.056075	1.374945
50	1	0	2.466484	6.524774	0.386533
51	1	0	0.929130	6.276519	1.271781
52	1	0	-2.463360	2.410810	0.659646

***S*-10d**

Center Number	Atomic Number	Atomic Type	Coordinates (Angstroms) X	Y	Z
1	6	0	-1.196652	1.345838	1.942645
2	6	0	-1.741998	1.845421	0.759157
3	6	0	-2.714095	2.844317	0.813075
4	6	0	-3.138317	3.343891	2.044107
5	6	0	-2.592775	2.842889	3.223438
6	6	0	-1.624472	1.839861	3.170836
7	6	0	-1.214006	1.375993	-0.584580
8	6	0	0.155318	1.949439	-0.853837
9	6	0	1.027374	0.966812	-1.116102
10	6	0	0.308955	-0.350385	-1.059630
11	7	0	-0.987224	-0.064173	-0.669510
12	6	0	0.376507	3.416035	-0.703204
13	8	0	-0.696315	4.086408	-1.369003
14	6	0	-1.240726	5.226876	-0.879457
15	6	0	-0.635504	5.844222	0.356820
16	7	0	2.348874	1.035875	-1.516074
17	6	0	3.388556	0.286803	-0.952568
18	6	0	3.221749	-0.441195	0.230514
19	6	0	4.278553	-1.171555	0.759601
20	6	0	5.512979	-1.156699	0.121729
21	6	0	5.702762	-0.423137	-1.043819
22	6	0	4.639457	0.290113	-1.580998
23	8	0	0.779653	-1.439534	-1.319705
24	6	0	-2.035483	-0.998270	-0.542186
25	6	0	-3.363435	-0.553921	-0.555771
26	6	0	-4.412160	-1.454126	-0.410151
27	6	0	-4.136125	-2.805271	-0.252534
28	6	0	-2.824820	-3.263772	-0.232563
29	6	0	-1.774123	-2.365120	-0.372115
30	35	0	-5.565177	-4.035761	-0.057023
31	35	0	6.958533	-2.141890	0.856015
32	8	0	-2.193471	5.684423	-1.456340
33	1	0	-1.904745	1.694579	-1.376973
34	1	0	0.375987	3.656158	0.364971
35	1	0	-3.139995	3.233276	-0.109910
36	1	0	-3.896656	4.119758	2.077969
37	1	0	-2.924273	3.227905	4.182488
38	1	0	-1.201545	1.443649	4.088522
39	1	0	-0.440321	0.565496	1.897138
40	1	0	-0.754934	-2.723447	-0.357019
41	1	0	-2.618487	-4.320471	-0.103633
42	1	0	-5.437045	-1.100918	-0.421478
43	1	0	-3.596895	0.497368	-0.674866
44	1	0	2.268532	-0.432033	0.748098
45	1	0	4.144464	-1.738476	1.674173
46	1	0	6.669887	-0.415473	-1.534229
47	1	0	4.775648	0.848758	-2.502432
48	1	0	1.331732	3.736007	-1.129978
49	1	0	-0.848983	5.219528	1.231162
50	1	0	0.447899	5.956819	0.268944
51	1	0	-1.097957	6.819332	0.499331
52	1	0	2.624636	1.867355	-2.021976

CpH

Center Number	Atomic Number	Atomic Type	Coordinates (Angstroms) X	Y	Z
1	6	0	3.105970	3.104665	0.126351
2	6	0	3.466312	3.208228	-1.328753
3	6	0	2.421252	1.175250	-0.913484
4	6	0	2.973283	1.907497	-1.896974
5	6	0	2.504148	1.923272	0.350770
6	1	0	3.312809	3.878208	0.855970
7	1	0	2.993640	4.075017	-1.810627
8	1	0	1.980263	0.189995	-1.015365
9	1	0	3.063802	1.631713	-2.940640
10	1	0	2.132264	1.561662	1.302887
11	1	0	4.548598	3.332317	-1.473170

10d·10d

Center Number	Atomic Number	Atomic Type	Coordinates (Angstroms) X	Y	Z
1	6	0	-1.594160	-1.643119	1.316610
2	6	0	-1.765442	-0.895712	2.483527
3	6	0	-2.852035	-1.179936	3.320929
4	6	0	-3.754205	-2.182291	2.990653
5	6	0	-3.564282	-2.915839	1.824041
6	6	0	-2.485806	-2.655968	0.988601
7	7	0	-0.910934	0.158799	2.832361
8	6	0	0.227993	0.546173	2.149569
9	6	0	0.621876	1.787668	1.808070
10	6	0	2.024155	1.745458	1.242652
11	7	0	2.322052	0.309663	1.241143
12	6	0	1.323720	-0.430622	1.826167
13	6	0	-0.187158	3.042661	1.919807
14	8	0	1.327125	-1.630412	2.033376
15	6	0	3.568300	-0.202155	0.817045
16	6	0	4.183390	0.352876	-0.306711
17	6	0	5.392399	-0.153886	-0.765941
18	6	0	5.979169	-1.219415	-0.096104
19	6	0	5.386639	-1.774537	1.031717
20	6	0	4.182290	-1.257949	1.496019
21	6	0	3.077503	2.488489	2.055004
22	6	0	3.992381	3.331595	1.426675
23	6	0	4.996621	3.957011	2.164412
24	6	0	5.089299	3.740607	3.536560
25	6	0	4.172787	2.900051	4.170370
26	6	0	3.172752	2.275966	3.431727
27	35	0	7.593755	-1.950575	-0.759148
28	35	0	-4.807804	-4.265217	1.355788
29	6	0	0.186786	3.043143	-1.920514
30	6	0	-0.622011	1.787958	-1.808300
31	6	0	-0.227936	0.546467	-2.149622
32	6	0	-1.323448	-0.430476	-1.825951
33	7	0	-2.321826	0.309705	-1.240888
34	6	0	-2.024201	1.745561	-1.242663
35	7	0	0.911019	0.159149	-2.832350
36	6	0	1.765566	-0.895318	-2.483609
37	6	0	1.594543	-1.642629	-1.316561
38	6	0	2.486273	-2.655434	-0.988643
39	6	0	3.564598	-2.915361	-1.824266
40	6	0	3.754276	-2.181912	-2.990973
41	6	0	2.852012	-1.179610	-3.321185
42	6	0	-3.077830	2.488293	-2.054926
43	6	0	-3.992779	3.331257	-1.426505
44	6	0	-4.997256	3.956432	-2.164117
45	6	0	-5.090101	3.739933	-3.536238
46	6	0	-4.173528	2.899515	-4.170142
47	6	0	-3.173258	2.275660	-3.431619
48	6	0	-3.567987	-0.202299	-0.816736
49	6	0	-4.181897	-1.258129	-1.495736
50	6	0	-5.386235	-1.774779	-1.031488
51	6	0	-5.978832	-1.219713	0.096328
52	6	0	-5.392124	-0.154186	0.766223
53	6	0	-4.183123	0.352658	0.307028
54	8	0	-1.326680	-1.630293	-2.033014
55	35	0	-7.593512	-1.950881	0.759153
56	35	0	4.808247	-4.264656	-1.356078
57	1	0	-2.018728	2.138181	-0.216980
58	1	0	-0.471400	3.426307	0.935008
59	1	0	-1.097888	2.859795	2.493503
60	1	0	1.097191	2.860392	-2.494758
61	1	0	0.471622	3.426872	-0.935924
62	1	0	2.018772	2.137888	0.216894
63	1	0	-3.909320	3.511267	-0.357837
64	1	0	-5.705076	4.610411	-1.664809
65	1	0	-5.871975	4.224510	-4.112451
66	1	0	-4.240090	2.729062	-5.239998
67	1	0	-2.460859	1.618182	-3.924654
68	1	0	3.909045	3.511546	0.357989

69	1	0	5.704387	4.611103	1.665175
70	1	0	5.870989	4.225371	4.112864
71	1	0	4.239211	2.729675	5.240248
72	1	0	2.460403	1.618381	3.924690
73	1	0	3.703230	1.160354	-0.850338
74	1	0	5.862317	0.261359	-1.650387
75	1	0	5.858073	-2.607601	1.540877
76	1	0	3.705524	-1.688080	2.367401
77	1	0	-3.705098	-1.688192	-2.367134
78	1	0	-5.857638	-2.607823	-1.540713
79	1	0	-5.862102	0.261032	1.650650
80	1	0	-3.703037	1.160205	0.850626
81	1	0	0.775453	-1.427852	-0.643389
82	1	0	2.353560	-3.206099	-0.063792
83	1	0	4.603339	-2.389701	-3.633339
84	1	0	2.992258	-0.607401	-4.234341
85	1	0	-2.992449	-0.607642	4.234004
86	1	0	-4.603380	-2.390050	3.632879
87	1	0	-2.352896	-3.206709	0.063824
88	1	0	-0.774948	-1.428379	0.643577
89	8	0	0.506505	4.079667	2.626318
90	6	0	1.278529	4.890082	1.876662
91	8	0	1.384300	4.783005	0.676395
92	6	0	1.999574	5.885435	2.736416
93	1	0	2.698063	5.338594	3.378893
94	1	0	2.546974	6.584400	2.106304
95	1	0	1.292096	6.416248	3.377041
96	8	0	-0.507403	4.079999	-2.626719
97	6	0	-1.279349	4.890191	-1.876724
98	8	0	-1.384682	4.782964	-0.676433
99	6	0	-2.000792	5.885569	-2.736122
100	1	0	-1.293480	6.416836	-3.376564
101	1	0	-2.699112	5.338735	-3.378772
102	1	0	-2.548361	6.584179	-2.105761
103	1	0	-1.318842	0.858864	3.438365
104	1	0	1.318718	0.859070	-3.438631

10d·CpH

Center Number	Atomic Number	Atomic Type	Coordinates (Angstroms) X	Y	Z
1	6	0	-3.313837	-0.687865	0.305261
2	6	0	-2.090375	-0.934498	-0.336652
3	6	0	-1.950125	-2.108108	-1.094053
4	6	0	-3.006553	-3.005693	-1.195481
5	6	0	-4.208386	-2.743928	-0.550929
6	6	0	-4.367824	-1.587551	0.199631
7	7	0	-1.038540	-0.008780	-0.198422
8	6	0	0.249360	-0.150670	-0.686342
9	6	0	1.008947	1.049797	-0.212482
10	6	0	0.193596	1.828237	0.513835
11	6	0	-1.191407	1.239529	0.548148
12	6	0	0.536564	3.096820	1.219496
13	8	0	-0.017010	4.259003	0.576240
14	6	0	0.527428	4.605728	-0.597980
15	8	0	1.493376	4.050949	-1.075580
16	7	0	2.303628	1.310786	-0.606176
17	6	0	3.371128	0.419302	-0.545563
18	6	0	4.584433	0.778567	-1.147600
19	6	0	5.685441	-0.064692	-1.082520
20	6	0	5.575779	-1.282113	-0.420492
21	6	0	4.381179	-1.656906	0.183017
22	6	0	3.285369	-0.805182	0.130366
23	8	0	0.686238	-1.057758	-1.368993
24	6	0	0.133900	-1.229630	2.618326
25	6	0	0.877707	-0.177295	3.008965
26	6	0	0.092499	0.648988	3.939810
27	6	0	-1.122079	0.092092	4.109778
28	6	0	-2.200369	2.200016	-0.063726
29	6	0	-3.022482	2.961589	0.764614
30	6	0	-3.889262	3.905738	0.217951
31	6	0	-3.943132	4.084854	-1.162243

32	6	0	-3.123439	3.320420	-1.993515
33	6	0	-2.249076	2.386140	-1.445780
34	35	0	7.076160	-2.441296	-0.330841
35	35	0	-5.644531	-3.972626	-0.698064
36	6	0	-0.217924	5.750154	-1.221649
37	1	0	-3.163933	3.453778	-3.070132
38	1	0	-5.307401	-1.382747	0.700264
39	1	0	-1.465730	1.021232	1.590853
40	1	0	2.358918	-1.095140	0.613991
41	1	0	-1.019204	-2.316967	-1.599108
42	1	0	4.307457	-2.605160	0.703978
43	1	0	4.659901	1.725316	-1.674416
44	1	0	-2.981565	2.816418	1.842443
45	1	0	-1.606134	1.793513	-2.092280
46	1	0	-2.888583	-3.910318	-1.781686
47	1	0	6.621400	0.221625	-1.549451
48	1	0	-3.463117	0.211304	0.890747
49	1	0	-4.623749	4.813729	-1.590753
50	1	0	-4.526127	4.495204	0.869925
51	1	0	1.621780	3.203621	1.300536
52	1	0	0.458047	1.552464	4.416182
53	1	0	-1.920670	0.456043	4.745060
54	1	0	1.893476	0.046969	2.700236
55	1	0	2.501828	2.293507	-0.771604
56	1	0	-0.226574	6.602432	-0.538022
57	1	0	0.253652	6.025088	-2.163376
58	1	0	-1.254853	5.443903	-1.389635
59	1	0	0.093634	3.108743	2.218298
60	1	0	0.423872	-2.019919	1.933904
61	6	0	-1.206162	-1.166951	3.294407
62	1	0	-1.378396	-2.046188	3.931127
63	1	0	-2.036783	-1.154077	2.573865

10d·CpH´

Center Number	Atomic Number	Atomic Type	Coordinates (Angstroms) X	Y	Z
1	6	0	-2.657206	-1.329832	0.019049
2	6	0	-2.946446	-0.563938	-1.114895
3	6	0	-4.192222	-0.707112	-1.736171
4	6	0	-5.132790	-1.599758	-1.239204
5	6	0	-4.821644	-2.365386	-0.121288
6	6	0	-3.589812	-2.236824	0.508272
7	7	0	-2.045395	0.377816	-1.642019
8	6	0	-0.755114	0.549018	-1.173372
9	6	0	-0.103551	1.674738	-0.849073
10	6	0	1.323159	1.353481	-0.473146
11	7	0	1.404903	-0.087452	-0.685703
12	6	0	0.203344	-0.602769	-1.135600
13	6	0	-0.601406	3.081110	-0.797896
14	6	0	1.600124	1.772810	0.962844
15	6	0	1.322314	0.911788	2.025386
16	6	0	1.533573	1.329384	3.336453
17	6	0	2.014159	2.612853	3.593931
18	6	0	2.283639	3.477255	2.535032
19	6	0	2.079945	3.057591	1.221084
20	6	0	2.610143	-0.801494	-0.522320
21	6	0	3.804071	-0.097422	-0.316573
22	6	0	5.003719	-0.775097	-0.136641
23	6	0	5.015805	-2.163045	-0.161424
24	6	0	3.841526	-2.877609	-0.360004
25	6	0	2.639158	-2.203390	-0.537398
26	8	0	-0.037581	-1.748176	-1.462857
27	35	0	6.649659	-3.091380	0.085960
28	35	0	-6.096074	-3.594854	0.557438
29	6	0	-3.171496	2.125308	1.159284
30	6	0	-3.384142	3.574122	1.303069
31	6	0	-2.467387	4.074213	2.152601
32	6	0	-1.581780	2.961507	2.637198
33	6	0	-2.121670	1.757832	1.919361
34	1	0	1.324498	0.650999	4.157643
35	1	0	5.921628	-0.220694	0.022655

36	1	0	2.018962	1.871155	-1.147264
37	1	0	-1.706874	-1.219098	0.529212
38	1	0	1.728390	-2.762419	-0.693847
39	1	0	-3.361434	-2.831165	1.385823
40	1	0	-4.420828	-0.117616	-2.619542
41	1	0	2.287854	3.730488	0.392152
42	1	0	0.952937	-0.090932	1.824584
43	1	0	3.858686	-3.961720	-0.373936
44	1	0	-6.097167	-1.704683	-1.723542
45	1	0	3.813616	0.985623	-0.284714
46	1	0	2.179570	2.936819	4.616488
47	1	0	2.658493	4.477344	2.728197
48	1	0	-3.782004	1.467641	0.548571
49	1	0	-1.721513	0.757579	2.036293
50	8	0	0.297431	3.969649	-1.474273
51	1	0	-1.620266	3.170591	-1.185289
52	1	0	-4.164613	4.132948	0.798595
53	1	0	-2.364798	5.106936	2.463083
54	1	0	-0.517269	3.139789	2.423058
55	1	0	-1.643283	2.838440	3.728038
56	6	0	0.332099	4.047815	-2.827331
57	8	0	1.184203	4.732488	-3.330634
58	6	0	-0.710995	3.294903	-3.617371
59	1	0	-0.521108	3.472151	-4.674252
60	1	0	-0.660945	2.220775	-3.415905
61	1	0	-1.715896	3.649795	-3.369460
62	1	0	-0.616185	3.447819	0.231735
63	1	0	-2.489641	1.213060	-2.004288

10d·CpH´´

Center Number	Atomic Number	Atomic Type	Coordinates (Angstroms) X	Y	Z
1	6	0	-3.476256	3.607362	0.774116
2	6	0	-2.334710	4.194052	1.186100
3	6	0	-1.411643	3.165459	1.689016
4	6	0	-2.005072	1.961578	1.586553
5	6	0	-3.375965	2.123280	0.994510
6	6	0	-0.789044	2.750012	-1.535778
7	6	0	-0.154752	1.427905	-1.255935
8	6	0	-0.764117	0.248628	-1.415496
9	6	0	0.230584	-0.854036	-1.258566
10	7	0	1.431495	-0.239698	-0.946580
11	6	0	1.289847	1.217091	-0.860327
12	6	0	2.658908	-0.887052	-0.699978
13	6	0	3.837157	-0.130878	-0.641851
14	6	0	5.060630	-0.741512	-0.395352
15	6	0	5.113017	-2.115917	-0.207165
16	6	0	3.955477	-2.881677	-0.256605
17	6	0	2.729491	-2.273113	-0.498292
18	35	0	6.780781	-2.954541	0.124768
19	8	0	0.021795	-2.042352	-1.412422
20	7	0	-2.086410	0.035713	-1.807872
21	6	0	-2.915441	-0.910908	-1.209837
22	6	0	-4.071106	-1.340783	-1.873259
23	6	0	-4.945910	-2.231635	-1.264569
24	6	0	-4.666050	-2.702341	0.012587
25	6	0	-3.523713	-2.286470	0.687397
26	6	0	-2.655097	-1.388700	0.081540
27	6	0	1.599392	1.725399	0.546972
28	6	0	2.232267	2.952205	0.738712
29	6	0	2.492458	3.414241	2.026583
30	6	0	2.128740	2.647736	3.131953
31	6	0	1.505392	1.415262	2.943474
32	6	0	1.244570	0.956639	1.655472
33	35	0	-5.857315	-3.921003	0.846448
34	1	0	1.224879	0.808334	3.798568
35	1	0	5.965676	-0.146285	-0.350982
36	1	0	1.952139	1.709020	-1.584590
37	1	0	-1.769903	-1.056420	0.614268
38	1	0	1.831207	-2.871587	-0.537522
39	1	0	-3.314741	-2.656341	1.685179

40	1	0	-4.285811	-0.976110	-2.873857
41	1	0	2.498350	3.561570	-0.119858
42	1	0	0.771519	-0.012116	1.511843
43	1	0	4.004253	-3.953989	-0.102969
44	1	0	-5.838840	-2.560417	-1.784667
45	1	0	3.814956	0.943698	-0.778037
46	1	0	2.333740	3.006763	4.135582
47	1	0	2.980181	4.374043	2.163809
48	8	0	0.031878	3.775096	-0.999313
49	1	0	-1.793236	2.775466	-1.094521
50	1	0	-4.348366	4.101411	0.361697
51	1	0	-2.110987	5.256123	1.161037
52	1	0	-4.164037	1.752375	1.664060
53	1	0	-3.482757	1.553002	0.058556
54	1	0	-0.412175	3.363060	2.062968
55	1	0	-1.582789	1.008757	1.881611
56	6	0	-0.304000	5.078769	-1.135571
57	8	0	0.387406	5.899660	-0.588917
58	6	0	-1.504406	5.412229	-1.985260
59	1	0	-1.655872	6.489596	-1.948885
60	1	0	-1.336467	5.104730	-3.021900
61	1	0	-2.396654	4.900489	-1.615070
62	1	0	-0.886826	2.879365	-2.622264
63	1	0	-2.346858	0.341224	-2.737121

***R*-11d**

Center Number	Atomic Number	Atomic Type	Coordinates (Angstroms) X	Y	Z
1	35	0	5.336052	-3.566961	-0.024813
2	35	0	-6.943913	-1.707841	0.533151
3	6	0	3.025532	3.062963	2.357271
4	8	0	-0.913931	-0.664719	-0.317687
5	7	0	0.976361	0.656853	-0.552482
6	7	0	-2.313934	1.999508	-0.783199
7	6	0	1.974894	-0.339270	-0.433246
8	6	0	-3.325359	1.069325	-0.476352
9	6	0	2.296115	2.670782	0.089202
10	6	0	4.233348	-1.087226	-0.889607
11	6	0	1.329723	2.044268	-0.895302
12	6	0	-3.400413	0.434489	0.768283
13	6	0	-0.377069	0.411220	-0.486787
14	6	0	1.736311	-1.521243	0.280037
15	6	0	-4.477459	-0.387073	1.071551
16	6	0	-4.358517	0.895192	-1.401369
17	6	0	-1.071257	1.743940	-0.720231
18	6	0	3.293637	3.527367	-0.372910
19	6	0	2.164915	2.440515	1.459701
20	6	0	3.976424	-2.258301	-0.189607
21	6	0	2.736263	-2.478947	0.396756
22	6	0	-5.476560	-0.578786	0.122218
23	6	0	-5.426141	0.052127	-1.114939
24	6	0	3.233268	-0.129652	-1.009011
25	6	0	4.019187	3.925036	1.892875
26	6	0	4.151507	4.157877	0.527155
27	1	0	2.922149	2.875241	3.421301
28	1	0	5.205247	-0.918426	-1.338992
29	1	0	1.772560	2.063981	-1.899595
30	1	0	-2.612814	0.591103	1.498013
31	1	0	0.772786	-1.695782	0.736653
32	1	0	-4.539262	-0.879411	2.035622
33	1	0	-4.310932	1.417613	-2.351129
34	1	0	3.403495	3.699701	-1.441413
35	1	0	1.391898	1.766545	1.820850
36	1	0	2.548727	-3.392833	0.949083
37	1	0	-6.218157	-0.100461	-1.839394
38	1	0	3.452938	0.782104	-1.551391
39	1	0	4.690482	4.409004	2.595064
40	1	0	4.926397	4.822505	0.158592
41	6	0	-0.016272	2.746168	-0.934915
42	6	0	-0.215610	4.050833	-1.100993
43	1	0	-1.226248	4.446704	-1.110624

44	1	0	0.611490	4.744072	-1.221905

S-11d

Center Number	Atomic Number	Atomic Type	Coordinates (Angstroms) X	Y	Z
1	35	0	-5.249630	-3.640101	0.066409
2	35	0	7.000550	-1.688104	0.324697
3	6	0	-3.336717	3.133398	2.113201
4	8	0	0.931095	-0.579099	0.081672
5	7	0	-0.957638	0.650863	-0.461417
6	7	0	2.304493	2.077681	-0.499675
7	6	0	-1.941495	-0.358479	-0.332255
8	6	0	3.332387	1.138957	-0.289085
9	6	0	-2.388551	2.649089	-0.053774
10	6	0	-4.129680	-1.214106	-0.920728
11	6	0	-1.317325	2.006499	-0.910660
12	6	0	3.521396	0.037127	-1.130500
13	6	0	0.391432	0.448487	-0.275656
14	6	0	-1.742652	-1.461903	0.506967
15	6	0	4.613761	-0.800566	-0.951815
16	6	0	4.268654	1.408907	0.713026
17	6	0	1.071674	1.778380	-0.560992
18	6	0	-3.363772	3.445217	-0.651551
19	6	0	-2.378131	2.495433	1.333803
20	6	0	-3.911261	-2.309160	-0.094861
21	6	0	-2.726802	-2.436919	0.619718
22	6	0	5.513054	-0.537781	0.076936
23	6	0	5.349576	0.558435	0.915326
24	6	0	-3.145080	-0.240722	-1.037035
25	6	0	-4.308099	3.935213	1.513279
26	6	0	-4.319798	4.091892	0.130480
27	1	0	-3.327214	3.004885	3.190920
28	1	0	-5.059156	-1.117586	-1.470243
29	1	0	-1.665698	1.963037	-1.950861
30	1	0	2.810483	-0.157815	-1.926640
31	1	0	-0.822874	-1.560993	1.065023
32	1	0	4.763649	-1.654815	-1.602447
33	1	0	4.134414	2.284633	1.339378
34	1	0	-3.378973	3.557427	-1.733622
35	1	0	-1.622892	1.868192	1.800995
36	1	0	-2.571007	-3.290885	1.269207
37	1	0	6.065396	0.754511	1.705783
38	1	0	-3.331042	0.611238	-1.680281
39	1	0	-5.055743	4.431648	2.123617
40	1	0	-5.076202	4.709543	-0.343198
41	6	0	0.006962	2.748402	-0.862155
42	6	0	0.182285	4.053982	-1.046293
43	1	0	1.179813	4.478465	-0.992231
44	1	0	-0.652789	4.719990	-1.242095

RC11d·11d

Center Number	Atomic Number	Atomic Type	Coordinates (Angstroms) X	Y	Z
1	6	0	-0.383774	1.496010	-1.574273
2	7	0	0.879010	1.551256	-1.734792
3	6	0	-1.203452	2.682614	-1.293045
4	6	0	-2.612777	2.227869	-0.971049
5	7	0	-2.587509	0.799609	-1.326863
6	6	0	-1.343865	0.321642	-1.669577
7	8	0	-1.065795	-0.818295	-1.991691
8	6	0	-3.724714	-0.017486	-1.120094
9	6	0	-3.681437	3.004748	-1.709359
10	6	0	1.762457	0.484145	-1.980193
11	6	0	1.203435	2.681986	1.293570
12	6	0	0.774418	3.941160	1.347638
13	6	0	-0.774528	3.941829	-1.346878
14	6	0	0.383826	1.495271	1.574528

15	6	0	1.344018	0.320997	1.669876
16	7	0	2.587667	0.799105	1.327341
17	6	0	2.612812	2.227390	0.971600
18	6	0	3.681372	3.004384	1.709929
19	6	0	3.724899	-0.017908	1.120403
20	8	0	1.065978	-0.818990	1.991833
21	6	0	-4.368176	4.022703	-1.049530
22	6	0	-5.315453	4.787533	-1.728461
23	6	0	-5.585445	4.528867	-3.069165
24	6	0	-4.903716	3.506882	-3.730197
25	6	0	-3.952489	2.749891	-3.054735
26	6	0	4.367848	4.022555	1.050153
27	6	0	5.314990	4.787536	1.729099
28	6	0	5.585113	4.528810	3.069766
29	6	0	4.903654	3.506608	3.730741
30	6	0	3.952558	2.749463	3.055264
31	6	0	4.736134	0.420378	0.256615
32	6	0	5.831769	-0.388718	-0.014279
33	6	0	5.923574	-1.637546	0.586323
34	6	0	4.944026	-2.076756	1.467034
35	6	0	3.847893	-1.266629	1.742817
36	6	0	-3.847742	-1.266011	-1.742895
37	6	0	-4.943914	-2.076178	-1.467386
38	6	0	-5.923450	-1.637208	-0.586540
39	6	0	-5.831581	-0.388591	0.014489
40	6	0	-4.735917	0.420550	-0.256147
41	6	0	1.718616	-0.764723	-1.346199
42	6	0	2.720281	-1.701563	-1.566190
43	6	0	3.762665	-1.397982	-2.434759
44	6	0	3.831376	-0.165664	-3.075593
45	6	0	2.844515	0.778160	-2.822756
46	7	0	-0.878993	1.550372	1.734782
47	6	0	-1.762393	0.483202	1.980111
48	6	0	-2.844464	0.777163	2.822681
49	6	0	-3.831340	-0.166663	3.075436
50	6	0	-3.762666	-1.398914	2.434468
51	6	0	-2.720271	-1.702443	1.565897
52	6	0	-1.718533	-0.765638	1.346062
53	35	0	-7.397069	-2.754918	-0.191378
54	35	0	7.397056	-2.755274	0.190683
55	35	0	5.154474	-2.654041	-2.682943
56	35	0	-5.154546	-2.654930	2.682485
57	1	0	-2.771042	2.322889	0.111967
58	1	0	1.439190	4.779831	1.161940
59	1	0	-0.261771	4.146656	1.597233
60	1	0	0.261633	4.147456	-1.596484
61	1	0	-1.439352	4.780412	-1.160960
62	1	0	2.771099	2.322452	-0.111407
63	1	0	-4.164527	4.213948	0.002077
64	1	0	-5.846628	5.577095	-1.206381
65	1	0	-6.328275	5.117402	-3.598161
66	1	0	-5.115051	3.299150	-4.774394
67	1	0	-3.422857	1.951644	-3.568694
68	1	0	4.164095	4.213855	-0.001425
69	1	0	5.845954	5.577267	1.207062
70	1	0	6.327836	5.117467	3.598775
71	1	0	5.115094	3.298832	4.774908
72	1	0	3.423130	1.951052	3.569178
73	1	0	4.672859	1.385033	-0.232151
74	1	0	6.600185	-0.054734	-0.702768
75	1	0	5.032676	-3.048043	1.940752
76	1	0	3.084831	-1.612123	2.424301
77	1	0	-3.084694	-1.611309	-2.424496
78	1	0	-5.032616	-3.047298	-1.941437
79	1	0	-6.599991	-0.054796	0.703077
80	1	0	-4.672625	1.385070	0.232883
81	1	0	0.917703	-1.002747	-0.657843
82	1	0	2.700085	-2.654891	-1.049809
83	1	0	4.659682	0.061533	-3.737512
84	1	0	2.892170	1.760535	-3.282082
85	1	0	-2.892117	1.759507	3.282076
86	1	0	-4.659648	0.060497	3.737368
87	1	0	-2.700088	-2.655724	1.049431
88	1	0	-0.917587	-1.003658	0.657741

TS11d·11d

Center Number	Atomic Number	Atomic Type	Coordinates (Angstroms) X	Y	Z
1	6	0	-0.469502	1.256668	-1.421294
2	7	0	0.816926	1.253200	-1.560933
3	6	0	-1.143931	2.453497	-1.042365
4	6	0	-2.607288	2.176628	-0.830319
5	7	0	-2.723410	0.753382	-1.179154
6	6	0	-1.523888	0.177162	-1.547795
7	8	0	-1.350747	-0.968642	-1.920585
8	6	0	-3.936750	0.055243	-0.972904
9	6	0	-3.486463	3.079971	-1.677771
10	6	0	1.682950	0.182575	-1.785224
11	6	0	1.143902	2.453272	1.042501
12	6	0	0.468747	3.650078	0.877255
13	6	0	-0.468769	3.650277	-0.876959
14	6	0	0.469502	1.256393	1.421312
15	6	0	1.523933	0.176936	1.547862
16	7	0	2.723457	0.753231	1.179331
17	6	0	2.607293	2.176484	0.830532
18	6	0	3.486420	3.079818	1.678042
19	6	0	3.936783	0.055104	0.972961
20	8	0	1.350824	-0.968892	1.920590
21	6	0	-4.032058	4.230712	-1.109458
22	6	0	-4.787897	5.107829	-1.884818
23	6	0	-5.007051	4.832175	-3.231957
24	6	0	-4.466308	3.679614	-3.800930
25	6	0	-3.705105	2.807775	-3.028445
26	6	0	4.031875	4.230680	1.109839
27	6	0	4.787720	5.107752	1.885243
28	6	0	5.007025	4.831937	3.232326
29	6	0	4.466431	3.679256	3.801189
30	6	0	3.705229	2.807456	3.028656
31	6	0	5.008491	0.711273	0.352519
32	6	0	6.189734	0.035793	0.070310
33	6	0	6.310569	-1.302011	0.415189
34	6	0	5.273472	-1.962883	1.059396
35	6	0	4.092752	-1.289604	1.346341
36	6	0	-4.092598	-1.289516	-1.346153
37	6	0	-5.273325	-1.962822	-1.059309
38	6	0	-6.310534	-1.301939	-0.415295
39	6	0	-6.189823	0.035915	-0.070565
40	6	0	-5.008582	0.711432	-0.352695
41	6	0	1.506679	-1.141197	-1.343035
42	6	0	2.518295	-2.076843	-1.502785
43	6	0	3.708910	-1.708507	-2.125710
44	6	0	3.905695	-0.413228	-2.588515
45	6	0	2.902551	0.526840	-2.394067
46	7	0	-0.816940	1.252882	1.560817
47	6	0	-1.682985	0.182266	1.785056
48	6	0	-2.902651	0.526578	2.393741
49	6	0	-3.905810	-0.413478	2.588177
50	6	0	-3.708968	-1.708796	2.125509
51	6	0	-2.518299	-2.077173	1.502710
52	6	0	-1.506677	-1.141533	1.342961
53	35	0	-7.888464	-2.246366	0.027420
54	35	0	7.888508	-2.246377	-0.027599
55	35	0	5.093731	-2.981983	-2.314151
56	35	0	-5.093742	-2.982304	2.314048
57	1	0	-2.857204	2.306167	0.233659
58	1	0	1.042308	4.562998	0.737771
59	1	0	-0.456234	3.744322	1.439598
60	1	0	0.456192	3.744635	-1.439314
61	1	0	-1.042333	4.563167	-0.737274
62	1	0	2.857244	2.306073	-0.233431
63	1	0	-3.870867	4.436761	-0.053212
64	1	0	-5.211401	5.999526	-1.433514
65	1	0	-5.601587	5.509657	-3.836461
66	1	0	-4.638924	3.458453	-4.849466
67	1	0	-3.286405	1.907805	-3.471328

68	1	0	3.870588	4.436864	0.053634
69	1	0	5.211113	5.999540	1.434015
70	1	0	5.601558	5.509389	3.836867
71	1	0	4.639159	3.457963	4.849680
72	1	0	3.286646	1.907397	3.471466
73	1	0	4.934499	1.751878	0.063336
74	1	0	7.004786	0.550587	-0.425921
75	1	0	5.377849	-3.008254	1.328471
76	1	0	3.287964	-1.812409	1.839230
77	1	0	-3.287710	-1.812333	-1.838866
78	1	0	-5.377602	-3.008234	-1.328264
79	1	0	-7.004970	0.550720	0.425501
80	1	0	-4.934671	1.752070	-0.063604
81	1	0	0.579637	-1.435902	-0.871924
82	1	0	2.388829	-3.089459	-1.136588
83	1	0	4.841386	-0.138492	-3.062808
84	1	0	3.040973	1.553254	-2.720431
85	1	0	-3.041102	1.553015	2.720021
86	1	0	-4.841542	-0.138707	3.062367
87	1	0	-2.388788	-3.089823	1.136623
88	1	0	-0.579579	-1.436286	0.871990

PROD11d·11d

Center Number	Atomic Number	Atomic Type	Coordinates (Angstroms) X	Y	Z
1	6	0	-0.400219	1.443511	-1.278816
2	7	0	0.928380	1.378254	-0.878953
3	6	0	-1.193497	2.471211	-0.950258
4	6	0	-2.635247	2.068039	-1.062800
5	7	0	-2.562092	0.693650	-1.563228
6	6	0	-1.239333	0.271616	-1.659304
7	8	0	-0.855430	-0.851011	-1.932210
8	6	0	-3.655682	-0.188821	-1.419424
9	6	0	-3.474904	2.990202	-1.921932
10	6	0	1.939532	0.584851	-1.505620
11	6	0	1.158128	2.115306	0.415892
12	6	0	0.719600	3.569630	0.255129
13	6	0	-0.735761	3.707372	-0.254757
14	6	0	0.380934	1.246999	1.386206
15	6	0	1.316754	0.118368	1.779969
16	7	0	2.584245	0.530218	1.422778
17	6	0	2.608285	1.902879	0.888474
18	6	0	3.077905	2.921648	1.906807
19	6	0	3.727555	-0.296730	1.329742
20	8	0	1.006461	-0.920106	2.330536
21	6	0	-4.303775	3.936826	-1.322211
22	6	0	-5.036409	4.827043	-2.105747
23	6	0	-4.944798	4.768620	-3.493416
24	6	0	-4.116749	3.821063	-4.096155
25	6	0	-3.381148	2.937583	-3.313688
26	6	0	3.824262	4.018505	1.470772
27	6	0	4.227057	5.001201	2.370764
28	6	0	3.888826	4.890654	3.717678
29	6	0	3.151297	3.794128	4.159283
30	6	0	2.747915	2.811668	3.258527
31	6	0	4.937448	0.259138	0.891578
32	6	0	6.022445	-0.554680	0.589503
33	6	0	5.912335	-1.928532	0.752135
34	6	0	4.747180	-2.489140	1.259853
35	6	0	3.656793	-1.679530	1.555407
36	6	0	-3.610064	-1.494040	-1.929343
37	6	0	-4.646304	-2.383846	-1.667156
38	6	0	-5.740713	-1.972607	-0.918024
39	6	0	-5.829395	-0.667149	-0.454041
40	6	0	-4.792773	0.221710	-0.708886
41	6	0	2.026553	-0.792268	-1.290643
42	6	0	3.128867	-1.508579	-1.743479
43	6	0	4.151824	-0.834782	-2.400145
44	6	0	4.059128	0.523341	-2.684009
45	6	0	2.940992	1.225260	-2.242278
46	7	0	-0.850677	1.427653	1.628843

47	6	0	-1.780386	0.456296	2.042245
48	6	0	-2.821245	0.848929	2.892388
49	6	0	-3.902739	0.005681	3.119224
50	6	0	-3.960411	-1.216928	2.457522
51	6	0	-2.939554	-1.625018	1.606646
52	6	0	-1.847022	-0.791414	1.406937
53	35	0	-7.124660	-3.201911	-0.520338
54	35	0	7.360141	-3.044045	0.265623
55	35	0	5.731062	-1.778079	-2.843581
56	35	0	-5.488468	-2.318284	2.648228
57	1	0	-3.032786	2.056561	-0.035764
58	1	0	1.416026	4.035439	-0.451769
59	1	0	0.827799	4.092245	1.209048
60	1	0	-0.816500	4.578057	-0.916298
61	1	0	-1.411116	3.889422	0.587876
62	1	0	3.273056	1.937233	0.025051
63	1	0	-4.379917	3.973978	-0.237406
64	1	0	-5.682458	5.558789	-1.631079
65	1	0	-5.519408	5.456197	-4.105923
66	1	0	-4.045100	3.771481	-5.178132
67	1	0	-2.734755	2.196827	-3.777318
68	1	0	4.091548	4.102010	0.419252
69	1	0	4.809623	5.847534	2.021200
70	1	0	4.205086	5.652738	4.422695
71	1	0	2.892458	3.698895	5.208922
72	1	0	2.186461	1.953093	3.616923
73	1	0	5.050344	1.330477	0.774952
74	1	0	6.943513	-0.118673	0.219157
75	1	0	4.678484	-3.561240	1.407712
76	1	0	2.744498	-2.124639	1.924389
77	1	0	-2.756146	-1.821004	-2.503515
78	1	0	-4.596111	-3.398635	-2.046261
79	1	0	-6.690970	-0.347926	0.121848
80	1	0	-4.882776	1.233287	-0.332358
81	1	0	1.247416	-1.296027	-0.728652
82	1	0	3.218144	-2.569420	-1.538435
83	1	0	4.857149	1.028233	-3.216538
84	1	0	2.857719	2.292766	-2.427524
85	1	0	-2.782648	1.829355	3.356493
86	1	0	-4.710947	0.309731	3.775025
87	1	0	-3.013583	-2.569651	1.078601
88	1	0	-1.064898	-1.086739	0.714257

RC1d·*exo*

Center Number	Atomic Number	Atomic Type	Coordinates (Angstroms) X	Y	Z
1	6	0	-0.438656	0.484896	3.180142
2	6	0	-0.142935	1.774390	3.431570
3	6	0	1.346411	1.931024	3.538923
4	6	0	1.852192	0.533446	3.327060
5	6	0	0.809806	-0.291481	3.113819
6	6	0	-0.008059	2.543341	0.257647
7	6	0	0.991003	1.474479	0.077335
8	6	0	0.217194	0.178796	-0.100628
9	7	0	-1.119260	0.501879	-0.009298
10	6	0	-1.390100	1.919942	0.266649
11	7	0	2.237935	1.713990	0.000769
12	6	0	3.278593	0.792424	-0.202379
13	6	0	4.325148	1.240354	-1.020140
14	6	0	5.436103	0.443050	-1.261500
15	6	0	5.522113	-0.794537	-0.633994
16	6	0	4.517398	-1.243256	0.216683
17	6	0	3.392633	-0.454806	0.426025
18	35	0	7.046522	-1.882774	-0.927980
19	8	0	0.676852	-0.926571	-0.311590
20	6	0	-2.171683	-0.441787	-0.039450
21	6	0	-2.030443	-1.673808	-0.692254
22	6	0	-3.082783	-2.581523	-0.703591
23	6	0	-4.279383	-2.262031	-0.074155
24	6	0	-4.442016	-1.039807	0.564107
25	6	0	-3.389977	-0.132119	0.578645

26	6	0	-2.336362	2.546093	-0.737150
27	6	0	-3.282434	3.474140	-0.304972
28	6	0	-4.122909	4.102251	-1.222791
29	6	0	-4.025431	3.796004	-2.576993
30	6	0	-3.084177	2.862556	-3.011591
31	6	0	-2.240868	2.242044	-2.096261
32	6	0	0.262466	3.841801	0.364011
33	35	0	-5.710842	-3.503177	-0.096287
34	1	0	-3.008128	2.617425	-4.066244
35	1	0	-5.381131	-0.792902	1.045929
36	1	0	-1.810848	2.015760	1.275086
37	1	0	1.735842	2.617324	2.771929
38	1	0	1.653602	2.343070	4.509422
39	1	0	2.602895	-0.800323	1.078535
40	1	0	1.292969	4.182782	0.343301
41	1	0	-0.525610	4.582558	0.462252
42	1	0	-1.102164	-1.923337	-1.184660
43	1	0	4.608943	-2.206259	0.706609
44	1	0	4.246959	2.224010	-1.471476
45	1	0	-0.844508	2.593682	3.540819
46	1	0	-3.365818	3.703686	0.755339
47	1	0	-1.509075	1.512474	-2.434352
48	1	0	-2.969917	-3.534041	-1.209124
49	1	0	6.233287	0.784977	-1.911730
50	1	0	-3.537176	0.821757	1.071049
51	1	0	0.857427	-1.357256	2.917704
52	1	0	-4.683526	4.278198	-3.292809
53	1	0	-1.429861	0.062859	3.043733
54	1	0	2.902022	0.265502	3.338910
55	1	0	-4.857585	4.822579	-0.877072

TS1d·*exo*

Center Number	Atomic Number	Atomic Type	Coordinates (Angstroms) X	Y	Z
1	6	0	3.403229	3.316175	2.830255
2	6	0	2.890667	3.003498	1.574076
3	6	0	1.933282	2.001481	1.433521
4	6	0	1.490257	1.310203	2.562100
5	6	0	2.006068	1.616536	3.815929
6	6	0	2.964627	2.620199	3.953431
7	6	0	1.328584	1.736215	0.068767
8	7	0	1.012928	0.321243	-0.162196
9	6	0	-0.337489	0.078299	-0.290533
10	6	0	-1.047512	1.416862	-0.131320
11	6	0	-0.009693	2.429614	-0.044719
12	6	0	2.041203	-0.646857	-0.263084
13	6	0	1.763103	-2.016920	-0.392284
14	6	0	2.801312	-2.935533	-0.491935
15	6	0	4.120053	-2.501689	-0.461900
16	6	0	4.415215	-1.152273	-0.328799
17	6	0	3.380244	-0.230633	-0.229208
18	6	0	-0.184567	3.741316	0.109680
19	6	0	-0.162998	4.586553	-2.044122
20	6	0	-1.089137	3.484572	-2.485510
21	6	0	-0.134144	2.373744	-2.788891
22	6	0	1.124176	2.874426	-2.833262
23	6	0	1.108458	4.244734	-2.376043
24	8	0	-0.873589	-0.985273	-0.530450
25	7	0	-2.302291	1.646704	-0.087760
26	6	0	-3.267218	0.627342	0.006207
27	6	0	-3.273710	-0.275519	1.078350
28	6	0	-4.294195	-1.206934	1.209209
29	6	0	-5.315596	-1.234123	0.264982
30	6	0	-5.338000	-0.336352	-0.795257
31	6	0	-4.320079	0.604261	-0.909050
32	35	0	5.529843	-3.761287	-0.598258
33	35	0	-6.711765	-2.509411	0.439896
34	1	0	1.664485	1.070509	4.688041
35	1	0	5.445415	-0.815962	-0.299012
36	1	0	2.016920	2.092507	-0.706929
37	1	0	-1.929174	3.237112	-1.831536

38	1	0	-1.516008	3.817527	-3.448450
39	1	0	-2.472470	-0.240174	1.810530
40	1	0	-1.192844	4.167060	0.175317
41	1	0	0.638357	4.404268	0.393563
42	1	0	0.741213	-2.362626	-0.419728
43	1	0	-4.297850	-1.907778	2.036721
44	1	0	-4.328828	1.324131	-1.721575
45	1	0	-0.498216	5.570761	-1.716408
46	1	0	3.237194	3.538937	0.689924
47	1	0	0.743582	0.523953	2.458056
48	1	0	2.576875	-3.991665	-0.592626
49	1	0	-6.146014	-0.364396	-1.518077
50	1	0	3.636404	0.814276	-0.111425
51	1	0	2.016791	2.327427	-3.113456
52	1	0	3.370494	2.856974	4.932412
53	1	0	1.986622	4.872434	-2.263490
54	1	0	-0.441781	1.373397	-3.077741
55	1	0	4.153524	4.097130	2.928326

PROD1d·*exo*

Center Number	Atomic Number	Atomic Type	Coordinates (Angstroms) X	Y	Z
1	6	0	2.410024	3.274961	3.226419
2	6	0	2.275514	3.004761	1.867726
3	6	0	1.455289	1.962416	1.428116
4	6	0	0.782546	1.182969	2.369348
5	6	0	0.916729	1.451854	3.729847
6	6	0	1.727580	2.498706	4.161284
7	6	0	1.293365	1.732861	-0.064145
8	7	0	1.077578	0.314096	-0.396127
9	6	0	-0.247014	0.015766	-0.605126
10	6	0	-0.987290	1.344722	-0.536323
11	6	0	0.046036	2.431671	-0.676645
12	6	0	2.138264	-0.618915	-0.381922
13	6	0	1.995455	-1.894715	-0.947419
14	6	0	3.065244	-2.781275	-0.945440
15	6	0	4.279161	-2.402635	-0.385589
16	6	0	4.438268	-1.143325	0.175881
17	6	0	3.369639	-0.254579	0.178548
18	6	0	-0.372646	3.813957	-0.119844
19	6	0	-0.363852	4.728373	-1.382725
20	6	0	-0.894199	3.744552	-2.442764
21	6	0	0.250839	2.739667	-2.223886
22	6	0	1.454353	3.662287	-2.319728
23	6	0	1.086944	4.845075	-1.815290
24	8	0	-0.739989	-1.070518	-0.835824
25	7	0	-2.234300	1.533139	-0.433438
26	6	0	-3.152838	0.475182	-0.258139
27	6	0	-3.147613	-0.304221	0.901605
28	6	0	-4.120938	-1.275852	1.096609
29	6	0	-5.098697	-1.462827	0.125688
30	6	0	-5.127708	-0.685762	-1.026437
31	6	0	-4.161686	0.297430	-1.206849
32	35	0	5.733540	-3.617221	-0.389337
33	35	0	-6.427543	-2.791498	0.387769
34	1	0	0.391126	0.836454	4.453063
35	1	0	5.386175	-0.853945	0.615204
36	1	0	2.197421	2.076317	-0.575639
37	1	0	-1.881339	3.340856	-2.200106
38	1	0	-0.889007	4.160100	-3.453142
39	1	0	-2.381709	-0.139984	1.653634
40	1	0	-1.378740	3.748985	0.303822
41	1	0	0.304064	4.176888	0.655637
42	1	0	1.054266	-2.192036	-1.385646
43	1	0	-4.118703	-1.884424	1.994008
44	1	0	-4.176866	0.926390	-2.091030
45	1	0	-0.906380	5.665201	-1.252479
46	1	0	2.805960	3.610536	1.135248
47	1	0	0.168061	0.345779	2.047565
48	1	0	2.951251	-3.765593	-1.385583
49	1	0	-5.902649	-0.838949	-1.769113

50	1	0	3.505237	0.716634	0.638819
51	1	0	2.441750	3.363005	-2.653502
52	1	0	1.833263	2.704445	5.221597
53	1	0	1.717565	5.711084	-1.645243
54	1	0	0.270900	1.845021	-2.850081
55	1	0	3.050809	4.087256	3.554570

RC1d·*exo´*

Center Number	Atomic Number	Atomic Type	Coordinates (Angstroms) X	Y	Z
1	6	0	-4.079863	-0.595416	-1.701073
2	6	0	-3.140049	-0.054862	-0.820353
3	6	0	-3.199912	-0.379279	0.539293
4	6	0	-4.165894	-1.258971	1.007886
5	6	0	-5.069806	-1.817364	0.109506
6	6	0	-5.034834	-1.495814	-1.242048
7	7	0	-2.246131	0.923108	-1.296846
8	6	0	-0.984178	0.833473	-1.180667
9	6	0	-0.038869	1.894104	-1.546065
10	6	0	1.360535	1.420773	-1.192591
11	7	0	1.160360	0.016678	-0.797689
12	6	0	-0.161952	-0.365485	-0.737066
13	6	0	-0.365025	3.059168	-2.099244
14	6	0	2.014296	2.248424	-0.097293
15	6	0	2.762036	3.372106	-0.449087
16	6	0	3.353624	4.161277	0.535105
17	6	0	3.211752	3.822352	1.879027
18	6	0	2.467461	2.697833	2.233249
19	6	0	1.867695	1.916316	1.249589
20	6	0	2.269633	-0.826755	-0.547393
21	6	0	2.114635	-2.121232	-0.028013
22	6	0	3.228343	-2.921305	0.199209
23	6	0	4.499768	-2.440927	-0.084397
24	6	0	4.673254	-1.160517	-0.591426
25	6	0	3.562948	-0.357430	-0.819367
26	8	0	-0.601216	-1.460239	-0.447999
27	35	0	6.011511	-3.538455	0.228930
28	35	0	-6.383645	-3.028905	0.745960
29	6	0	-2.203243	5.137737	-0.207132
30	6	0	-2.509360	3.845943	0.496146
31	6	0	-1.252576	3.575517	1.271662
32	6	0	-0.366523	4.562852	1.045835
33	6	0	-0.960393	5.538152	0.122054
34	1	0	2.355828	2.425806	3.278173
35	1	0	5.667165	-0.784744	-0.805982
36	1	0	1.988735	1.451072	-2.090706
37	1	0	-2.485930	0.065987	1.225857
38	1	0	0.379256	3.815492	-2.329674
39	1	0	1.130585	-2.504803	0.192685
40	1	0	-4.214745	-1.513361	2.060779
41	1	0	-4.048347	-0.315101	-2.748724
42	1	0	2.895314	3.625100	-1.498703
43	1	0	1.296668	1.035499	1.531349
44	1	0	3.099521	-3.920877	0.599129
45	1	0	-5.752853	-1.933430	-1.926576
46	1	0	3.727874	0.643113	-1.198235
47	1	0	3.682617	4.428212	2.646637
48	1	0	3.936352	5.031569	0.250088
49	1	0	-1.403050	3.284130	-2.323374
50	1	0	-1.103231	2.705582	1.901776
51	1	0	0.636011	4.632614	1.453647
52	1	0	-2.727319	3.035678	-0.215570
53	1	0	-0.459926	6.433913	-0.229660
54	1	0	-2.896411	5.644814	-0.868049
55	1	0	-3.390338	3.930702	1.147632

TS1d·*exo´*

Center	Atomic	Atomic	Coordinates (Angstroms)

Number	Number	Type	X	Y	Z
1	6	0	-3.442496	-0.600173	-1.147545
2	6	0	-3.299991	0.564144	-0.380619
3	6	0	-4.280137	0.870636	0.570096
4	6	0	-5.354586	0.015010	0.787170
5	6	0	-5.465673	-1.139766	0.022972
6	6	0	-4.520200	-1.452133	-0.948671
7	7	0	-2.293101	1.506269	-0.637709
8	6	0	-1.036495	1.255501	-0.567353
9	6	0	0.024742	2.153776	-0.951827
10	6	0	1.338188	1.375044	-0.940375
11	7	0	0.957382	0.071239	-0.380657
12	6	0	-0.386198	-0.036577	-0.098950
13	6	0	-0.188808	3.307042	-1.712226
14	6	0	2.504960	1.999843	-0.197808
15	6	0	3.293290	2.943014	-0.856450
16	6	0	4.337562	3.581615	-0.189972
17	6	0	4.621938	3.254742	1.133897
18	6	0	3.854847	2.289840	1.787078
19	6	0	2.796168	1.672734	1.127713
20	6	0	1.888428	-0.986893	-0.267793
21	6	0	1.675232	-2.056081	0.612720
22	6	0	2.607884	-3.083488	0.702137
23	6	0	3.757685	-3.043759	-0.075334
24	6	0	3.992003	-1.984774	-0.942914
25	6	0	3.059014	-0.959926	-1.036718
26	8	0	-0.949007	-0.984527	0.411940
27	35	0	5.027690	-4.444890	0.055813
28	35	0	-6.941916	-2.303511	0.294613
29	6	0	-0.466998	4.905678	-0.557548
30	6	0	-1.239864	4.210772	0.540217
31	6	0	-0.128167	3.619519	1.339720
32	6	0	1.030114	4.320993	1.074669
33	6	0	0.819920	5.140278	-0.050267
34	1	0	4.078835	2.020621	2.814640
35	1	0	4.894742	-1.955968	-1.542535
36	1	0	1.646651	1.234413	-1.986925
37	1	0	-4.186524	1.786203	1.145916
38	1	0	0.615619	3.663749	-2.349478
39	1	0	0.782840	-2.086934	1.220378
40	1	0	-6.103713	0.249444	1.535526
41	1	0	-2.699639	-0.832567	-1.904239
42	1	0	3.088485	3.183161	-1.897985
43	1	0	2.191682	0.933901	1.646433
44	1	0	2.438260	-3.909639	1.383528
45	1	0	-4.625750	-2.353525	-1.542366
46	1	0	3.259845	-0.136571	-1.711968
47	1	0	5.443889	3.739797	1.650835
48	1	0	4.936633	4.322970	-0.709658
49	1	0	-1.180625	3.407115	-2.147268
50	1	0	-0.268902	2.907633	2.145760
51	1	0	1.977756	4.205809	1.586614
52	1	0	-2.010131	3.510645	0.212567
53	1	0	1.578260	5.753396	-0.525003
54	1	0	-0.946943	5.575966	-1.263561
55	1	0	-1.725423	4.988395	1.150844

RC1d·*endo*

Center Number	Atomic Number	Atomic Type	Coordinates (Angstroms) X	Y	Z
1	6	0	3.135377	-0.404935	-0.469316
2	6	0	3.124648	-0.007025	0.873362
3	6	0	4.132729	-0.456884	1.730794
4	6	0	5.106034	-1.337742	1.273061
5	6	0	5.091800	-1.729676	-0.060351
6	6	0	4.120203	-1.262285	-0.939958
7	7	0	2.216667	0.952783	1.352704
8	6	0	0.956445	0.855905	1.224264
9	6	0	0.007593	1.917416	1.579505
10	6	0	-1.397447	1.423778	1.277009

11	7	0	-1.187745	0.038243	0.831170
12	6	0	0.136083	-0.342333	0.774522
13	6	0	0.341480	3.105534	2.078037
14	6	0	-2.099831	2.280278	0.238950
15	6	0	-2.827627	3.395363	0.656845
16	6	0	-3.399166	4.253917	-0.280292
17	6	0	-3.258691	3.992786	-1.641497
18	6	0	-2.551616	2.867057	-2.060683
19	6	0	-1.973551	2.014171	-1.124312
20	6	0	-2.291235	-0.805252	0.558451
21	6	0	-3.579927	-0.387246	0.918672
22	6	0	-4.684732	-1.189270	0.661225
23	6	0	-4.508769	-2.415981	0.036020
24	6	0	-3.241548	-2.843226	-0.338196
25	6	0	-2.134038	-2.043468	-0.082184
26	8	0	0.569559	-1.439184	0.484375
27	35	0	-6.012531	-3.512406	-0.317144
28	35	0	6.431359	-2.911090	-0.699346
29	6	0	1.511678	5.155582	-0.195077
30	6	0	0.473978	4.532602	-1.084670
31	6	0	1.141116	3.270689	-1.552500
32	6	0	2.372499	3.192107	-1.014701
33	6	0	2.602340	4.366586	-0.163192
34	1	0	-2.448212	2.651895	-3.119721
35	1	0	-5.676062	-0.854772	0.944835
36	1	0	-1.985579	1.416815	2.202980
37	1	0	2.366886	-0.033880	-1.140834
38	1	0	-1.152346	-2.384560	-0.373248
39	1	0	4.131309	-1.570275	-1.979466
40	1	0	4.139139	-0.118650	2.761691
41	1	0	-2.949628	3.593667	1.719563
42	1	0	-1.421220	1.138469	-1.454686
43	1	0	-3.112194	-3.799629	-0.832376
44	1	0	5.876680	-1.703997	1.942100
45	1	0	-3.744781	0.572326	1.392368
46	1	0	-3.706772	4.658100	-2.372540
47	1	0	-3.959868	5.120906	0.054383
48	1	0	0.681375	2.562928	-2.232347
49	1	0	-0.459455	4.326169	-0.541530
50	1	0	3.095737	2.397705	-1.165783
51	1	0	1.374291	6.094541	0.328328
52	1	0	3.513118	4.548070	0.396818
53	1	0	0.200452	5.187732	-1.923896
54	1	0	-0.400602	3.865249	2.307123
55	1	0	1.384880	3.339175	2.258553

TS1d·*endo*

Center Number	Atomic Number	Atomic Type	Coordinates (Angstroms) X	Y	Z
1	6	0	-3.144296	-0.536678	0.437144
2	6	0	-3.196932	0.158354	-0.782880
3	6	0	-4.358407	0.046126	-1.562672
4	6	0	-5.414435	-0.766648	-1.171780
5	6	0	-5.326602	-1.451753	0.034899
6	6	0	-4.202705	-1.337022	0.846627
7	7	0	-2.228240	1.064213	-1.225581
8	6	0	-0.946307	0.912747	-1.066672
9	6	0	-0.033386	1.964469	-1.312045
10	6	0	1.386710	1.472969	-1.130234
11	7	0	1.193725	0.089667	-0.682663
12	6	0	-0.134976	-0.301546	-0.662285
13	6	0	-0.471133	3.219617	-1.713158
14	6	0	2.239877	2.303517	-0.194314
15	6	0	2.917149	3.411011	-0.706253
16	6	0	3.626032	4.258923	0.143439
17	6	0	3.678172	3.989680	1.508818
18	6	0	3.029762	2.864579	2.018161
19	6	0	2.313658	2.025029	1.170388
20	6	0	2.290676	-0.777599	-0.486782
21	6	0	3.550465	-0.428247	-0.990926
22	6	0	4.648801	-1.256550	-0.793792

23	6	0	4.492998	-2.441389	-0.087641
24	6	0	3.255124	-2.799976	0.430581
25	6	0	2.156625	-1.970879	0.237944
26	8	0	-0.542795	-1.424452	-0.435739
27	35	0	5.987131	-3.577467	0.177919
28	35	0	-6.770336	-2.551296	0.590187
29	6	0	-1.407197	4.177084	-0.208591
30	6	0	-0.354626	4.207721	0.883601
31	6	0	-0.712678	3.004462	1.698406
32	6	0	-1.956261	2.574169	1.359992
33	6	0	-2.418535	3.308671	0.220566
34	1	0	3.083331	2.640161	3.079082
35	1	0	5.618659	-0.976971	-1.189357
36	1	0	1.875673	1.456707	-2.115578
37	1	0	-2.264279	-0.454100	1.064272
38	1	0	1.195845	-2.253728	0.641962
39	1	0	-4.155125	-1.871167	1.789326
40	1	0	-4.411772	0.606003	-2.490988
41	1	0	2.886831	3.613205	-1.774915
42	1	0	1.802187	1.151901	1.566959
43	1	0	3.144406	-3.722695	0.989218
44	1	0	-6.300592	-0.859203	-1.789899
45	1	0	3.693317	0.496517	-1.536817
46	1	0	4.232983	4.645156	2.172480
47	1	0	4.141839	5.123138	-0.263064
48	1	0	-0.095998	2.615772	2.499739
49	1	0	0.683047	4.240916	0.544617
50	1	0	-2.511552	1.777057	1.840319
51	1	0	-1.630035	5.061101	-0.797803
52	1	0	-3.403585	3.220376	-0.218979
53	1	0	-0.524792	5.108562	1.493103
54	1	0	0.264928	3.984872	-1.951451
55	1	0	-1.374610	3.236541	-2.315557

PROD1d·*endo*

Center Number	Atomic Number	Atomic Type	Coordinates (Angstroms) X	Y	Z
1	6	0	-2.713545	-0.502450	0.569649
2	6	0	-2.972370	0.050250	-0.687767
3	6	0	-4.037119	-0.445802	-1.444156
4	6	0	-4.836312	-1.472833	-0.954626
5	6	0	-4.550670	-2.017676	0.291472
6	6	0	-3.490391	-1.545251	1.057658
7	7	0	-2.197740	1.121796	-1.188074
8	6	0	-0.834289	1.156974	-0.984903
9	6	0	-0.058135	2.250266	-0.978240
10	6	0	1.383778	1.872672	-0.783973
11	7	0	1.327038	0.412091	-0.718591
12	6	0	0.035640	-0.058404	-0.912943
13	6	0	-0.574376	3.632488	-1.159766
14	6	0	1.953494	2.508597	0.472148
15	6	0	2.843685	3.576068	0.378990
16	6	0	3.318180	4.199330	1.532812
17	6	0	2.904864	3.752421	2.784194
18	6	0	2.012141	2.683968	2.880436
19	6	0	1.534129	2.068666	1.729062
20	6	0	2.481922	-0.381339	-0.589312
21	6	0	3.744253	0.199208	-0.772265
22	6	0	4.901647	-0.556484	-0.627778
23	6	0	4.803268	-1.901881	-0.301777
24	6	0	3.561571	-2.495321	-0.112302
25	6	0	2.403189	-1.740521	-0.250338
26	8	0	-0.302343	-1.220830	-1.026061
27	35	0	6.379955	-2.937969	-0.107374
28	35	0	-5.629625	-3.427444	0.961276
29	6	0	-2.096790	3.643506	-1.008156
30	6	0	-2.620304	3.758701	0.434061
31	6	0	-4.036640	3.262794	0.282060
32	6	0	-4.186709	2.554085	-0.836839
33	6	0	-2.858777	2.406031	-1.562606
34	1	0	1.688325	2.332003	3.854907

35	1	0	5.872748	-0.096078	-0.770565
36	1	0	1.983402	2.182605	-1.651984
37	1	0	-1.900376	-0.105943	1.171499
38	1	0	1.439408	-2.205470	-0.102831
39	1	0	-3.285761	-1.976431	2.031335
40	1	0	-4.233116	-0.028331	-2.427475
41	1	0	3.170645	3.919639	-0.600230
42	1	0	0.836293	1.236981	1.798488
43	1	0	3.493568	-3.545664	0.148565
44	1	0	-5.662926	-1.856171	-1.542245
45	1	0	3.840599	1.248716	-1.022960
46	1	0	3.277228	4.232096	3.683809
47	1	0	4.013989	5.028415	1.451178
48	1	0	-4.821593	3.460316	1.004899
49	1	0	-2.045555	3.113775	1.116662
50	1	0	-5.103031	2.079255	-1.167056
51	1	0	-2.501578	4.497876	-1.560691
52	1	0	-2.944483	2.447455	-2.654473
53	1	0	-2.551842	4.781263	0.817901
54	1	0	-0.121963	4.315936	-0.428891
55	1	0	-0.283885	4.008290	-2.150047

PROD1d·*exo´*

Center Number	Atomic Number	Atomic Type	Coordinates (Angstroms) X	Y	Z
1	6	0	3.995957	0.211708	1.450294
2	6	0	3.280191	0.668969	0.342499
3	6	0	3.690001	0.308063	-0.944434
4	6	0	4.788741	-0.521862	-1.121452
5	6	0	5.472748	-0.993208	-0.005297
6	6	0	5.084522	-0.637293	1.280122
7	7	0	2.244416	1.607001	0.541249
8	6	0	1.027530	1.379070	0.276179
9	6	0	-0.086169	2.398569	0.404121
10	6	0	-1.320607	1.475320	0.671838
11	7	0	-0.923136	0.161156	0.133485
12	6	0	0.422961	0.039160	-0.094131
13	6	0	0.185146	3.474570	1.490703
14	6	0	-2.640746	1.930430	0.094918
15	6	0	-3.507517	2.688716	0.880878
16	6	0	-4.689685	3.190941	0.343946
17	6	0	-5.022376	2.919669	-0.981592
18	6	0	-4.174488	2.134301	-1.762075
19	6	0	-2.989442	1.640300	-1.225607
20	6	0	-1.804003	-0.948024	0.133627
21	6	0	-1.640421	-1.980551	-0.795565
22	6	0	-2.509308	-3.065376	-0.790504
23	6	0	-3.544731	-3.111506	0.134964
24	6	0	-3.726659	-2.088312	1.056976
25	6	0	-2.853456	-1.007110	1.055281
26	8	0	1.017520	-0.958459	-0.449837
27	35	0	-4.731722	-4.588645	0.133469
28	35	0	6.970659	-2.133717	-0.243470
29	6	0	0.404231	4.785060	0.670844
30	6	0	1.065567	4.230585	-0.605312
31	6	0	-0.113139	3.283256	-0.921662
32	6	0	-1.267059	4.275702	-0.826059
33	6	0	-0.952946	5.168849	0.116148
34	1	0	-4.436915	1.906007	-2.790312
35	1	0	-4.540496	-2.132465	1.771739
36	1	0	-1.427131	1.389885	1.762463
37	1	0	3.138273	0.678059	-1.802741
38	1	0	-0.656433	3.571221	2.183108
39	1	0	-0.834352	-1.937949	-1.515575
40	1	0	5.107334	-0.807369	-2.117828
41	1	0	3.690052	0.517860	2.445346
42	1	0	-3.246462	2.901955	1.915292
43	1	0	-2.328023	1.031699	-1.837011
44	1	0	-2.383638	-3.867045	-1.509319
45	1	0	5.631961	-1.008790	2.139128
46	1	0	-3.001881	-0.209073	1.774127

47	1	0	-5.945135	3.307165	-1.401645
48	1	0	-5.352114	3.789642	0.961109
49	1	0	1.078568	3.210560	2.062942
50	1	0	-0.055617	2.694601	-1.840524
51	1	0	-2.198772	4.200962	-1.374181
52	1	0	2.013319	3.722610	-0.419996
53	1	0	-1.584541	5.960960	0.503640
54	1	0	0.944555	5.559766	1.216055
55	1	0	1.186454	4.993027	-1.378102

RC2d·*endo*

Center Number	Atomic Number	Atomic Type	Coordinates (Angstroms) X	Y	Z
1	6	0	-4.194260	-0.823293	-1.432883
2	6	0	-3.260585	-0.718537	-0.395897
3	6	0	-3.455058	0.241286	0.604029
4	6	0	-4.546338	1.098417	0.553845
5	6	0	-5.439479	1.002935	-0.508155
6	6	0	-5.271658	0.051106	-1.507265
7	7	0	-2.245318	-1.689787	-0.344128
8	6	0	-1.008395	-1.451219	-0.175597
9	6	0	0.010836	-2.504677	-0.067383
10	6	0	1.377601	-1.851087	0.012279
11	7	0	1.071673	-0.416612	-0.076402
12	6	0	-0.272890	-0.121942	-0.134970
13	6	0	-0.235823	-3.811784	-0.033651
14	6	0	2.327428	-2.323212	-1.069709
15	6	0	3.301029	-3.271374	-0.760272
16	6	0	4.147065	-3.761758	-1.753514
17	6	0	4.026716	-3.296404	-3.059796
18	6	0	3.057734	-2.342218	-3.370741
19	6	0	2.209006	-1.858873	-2.380578
20	6	0	2.099708	0.549449	0.020733
21	6	0	1.913527	1.862126	-0.434150
22	6	0	2.942718	2.789793	-0.327893
23	6	0	4.161079	2.412402	0.222606
24	6	0	4.367973	1.112732	0.664620
25	6	0	3.339163	0.184393	0.561461
26	8	0	-0.771081	0.985721	-0.177851
27	35	0	5.560882	3.681797	0.360845
28	35	0	-6.922385	2.182451	-0.586319
29	6	0	-0.752785	-0.968352	2.950928
30	6	0	0.443063	-1.541571	3.185750
31	6	0	0.252823	-3.018622	3.384937
32	6	0	-1.229273	-3.197431	3.210263
33	6	0	-1.798740	-2.003601	2.967050
34	1	0	2.964513	-1.973311	-4.387285
35	1	0	5.323714	0.821482	1.085186
36	1	0	1.816104	-2.066150	0.994869
37	1	0	-2.745126	0.316746	1.419316
38	1	0	0.968417	2.158428	-0.864535
39	1	0	-4.697671	1.840710	1.329464
40	1	0	-4.059087	-1.593295	-2.185355
41	1	0	3.401604	-3.622905	0.264848
42	1	0	1.456304	-1.112336	-2.621920
43	1	0	2.794460	3.804345	-0.680435
44	1	0	-5.982906	-0.017310	-2.322660
45	1	0	3.522544	-0.828204	0.899842
46	1	0	4.688672	-3.670838	-3.834110
47	1	0	4.903740	-4.498576	-1.503248
48	1	0	-0.925119	0.091397	2.789127
49	1	0	1.399650	-1.034761	3.252199
50	1	0	0.565131	-4.543097	0.031796
51	1	0	-1.259466	-4.169559	-0.080501
52	1	0	-2.853358	-1.816097	2.796452
53	1	0	-1.732793	-4.154123	3.278114
54	1	0	0.589735	-3.338520	4.380680
55	1	0	0.824598	-3.615776	2.660025

TS2d·*endo*

Center Number	Atomic Number	Atomic Type	Coordinates (Angstroms) X	Y	Z
1	6	0	-4.271493	0.608107	1.321269
2	6	0	-3.219680	0.453448	0.405213
3	6	0	-3.303897	-0.584609	-0.537256
4	6	0	-4.387978	-1.452778	-0.544555
5	6	0	-5.398789	-1.291877	0.397434
6	6	0	-5.349538	-0.267086	1.335722
7	7	0	-2.223421	1.433652	0.415687
8	6	0	-0.966141	1.212794	0.199162
9	6	0	-0.034244	2.266878	0.004880
10	6	0	1.358405	1.709359	-0.178693
11	7	0	1.141548	0.262204	-0.047725
12	6	0	-0.183631	-0.086341	0.142149
13	6	0	-0.420548	3.601578	0.055331
14	6	0	2.342044	2.252305	0.842638
15	6	0	3.253663	3.236797	0.466223
16	6	0	4.119602	3.793117	1.407033
17	6	0	4.081301	3.357978	2.728298
18	6	0	3.172860	2.368745	3.107205
19	6	0	2.304046	1.821332	2.169810
20	6	0	2.216016	-0.648921	-0.145010
21	6	0	2.125256	-1.951277	0.366715
22	6	0	3.204438	-2.821019	0.261064
23	6	0	4.379838	-2.396650	-0.344875
24	6	0	4.493017	-1.106845	-0.845706
25	6	0	3.413725	-0.237909	-0.745212
26	8	0	-0.613267	-1.215195	0.285354
27	35	0	5.849586	-3.586092	-0.483494
28	35	0	-6.878038	-2.481151	0.391553
29	6	0	-1.214574	4.098485	-1.685113
30	6	0	-0.130686	3.573147	-2.607584
31	6	0	-0.586608	2.165011	-2.832764
32	6	0	-1.910339	2.064857	-2.511220
33	6	0	-2.321203	3.248790	-1.842123
34	1	0	3.142463	2.023887	4.136010
35	1	0	5.415011	-0.778118	-1.311697
36	1	0	1.744090	1.928438	-1.183026
37	1	0	-2.508889	-0.717280	-1.261993
38	1	0	1.213878	-2.284008	0.840746
39	1	0	-4.446495	-2.252117	-1.275222
40	1	0	-4.221898	1.428607	2.029921
41	1	0	3.290936	3.567466	-0.570438
42	1	0	1.595531	1.050753	2.464154
43	1	0	3.128087	-3.827099	0.658260
44	1	0	-6.149347	-0.148745	2.058318
45	1	0	3.522612	0.766847	-1.135281
46	1	0	4.758686	3.783844	3.461684
47	1	0	4.827300	4.558415	1.104044
48	1	0	-2.540793	1.196999	-2.666110
49	1	0	0.015900	1.398263	-3.307220
50	1	0	0.354401	4.364845	0.087760
51	1	0	-1.306997	3.817458	0.644215
52	1	0	-3.316579	3.435593	-1.458481
53	1	0	-1.331858	5.160820	-1.494009
54	1	0	-0.193049	4.120090	-3.560740
55	1	0	0.892901	3.684615	-2.241746

PROD2d·*endo*

Center Number	Atomic Number	Atomic Type	Coordinates (Angstroms) X	Y	Z
1	6	0	-3.149033	-0.284545	1.044994
2	6	0	-3.102162	0.404397	-0.169180
3	6	0	-4.017314	0.095395	-1.177478
4	6	0	-4.942753	-0.924997	-0.993390
5	6	0	-4.966400	-1.611808	0.215048
6	6	0	-4.081378	-1.296685	1.239550

7	7	0	-2.225754	1.497565	-0.349198
8	6	0	-0.978994	1.342205	-0.493576
9	6	0	0.015319	2.453623	-0.667063
10	6	0	1.301047	1.797976	-0.075764
11	7	0	1.113745	0.369639	-0.385920
12	6	0	-0.207822	0.033927	-0.563047
13	6	0	-0.407472	3.836952	-0.119213
14	6	0	-0.358447	4.755484	-1.380215
15	6	0	0.873112	4.169555	-2.093696
16	6	0	0.258874	2.761400	-2.208909
17	6	0	-1.092578	3.099550	-2.807636
18	6	0	-1.461060	4.284334	-2.311095
19	6	0	1.495924	2.047729	1.408951
20	6	0	2.365831	3.057765	1.826155
21	6	0	2.522227	3.344300	3.179734
22	6	0	1.811520	2.616565	4.131562
23	6	0	0.950161	1.600913	3.722709
24	6	0	0.794346	1.316236	2.368412
25	6	0	2.198263	-0.536216	-0.387386
26	6	0	2.072694	-1.821913	-0.934466
27	6	0	3.163974	-2.681564	-0.951552
28	6	0	4.382604	-2.266607	-0.428809
29	6	0	4.524799	-0.997315	0.114091
30	6	0	3.434569	-0.135227	0.135480
31	8	0	-0.677857	-1.068881	-0.760047
32	35	0	5.866020	-3.444902	-0.458930
33	35	0	-6.241114	-2.993580	0.476436
34	1	0	0.401398	1.022859	4.459407
35	1	0	5.476509	-0.679137	0.524341
36	1	0	2.182650	2.153764	-0.620409
37	1	0	-3.990852	0.652757	-2.108245
38	1	0	1.127952	-2.148200	-1.343372
39	1	0	-5.644763	-1.179368	-1.779622
40	1	0	-2.455520	-0.020467	1.837617
41	1	0	2.926193	3.623364	1.083984
42	1	0	0.136271	0.505718	2.065235
43	1	0	3.062952	-3.673449	-1.377609
44	1	0	-4.118190	-1.837108	2.178951
45	1	0	3.558718	0.844191	0.581050
46	1	0	1.933979	2.834651	5.187561
47	1	0	3.203138	4.130667	3.489569
48	1	0	-1.682474	2.427302	-3.420273
49	1	0	0.842979	1.986617	-2.710730
50	1	0	0.292672	4.193287	0.641077
51	1	0	-1.404154	3.791295	0.324679
52	1	0	-2.416202	4.781126	-2.440020
53	1	0	-0.342133	5.820849	-1.147998
54	1	0	1.060801	4.619593	-3.071141
55	1	0	1.784164	4.208598	-1.485805

RC2d·*exo*

Center Number	Atomic Number	Atomic Type	Coordinates (Angstroms) X	Y	Z
1	35	0	6.315775	-3.127312	-0.732764
2	35	0	-6.192314	-3.432578	0.395735
3	6	0	3.348702	2.546253	2.936296
4	8	0	-0.291169	-1.353171	0.169563
5	7	0	1.313853	0.296095	-0.112679
6	6	0	2.464398	-0.515605	-0.253142
7	6	0	-2.983429	-0.034542	0.401895
8	6	0	2.390728	2.380371	0.726223
9	6	0	4.728395	-0.785938	-1.071697
10	6	0	1.399698	1.761168	-0.237821
11	6	0	-3.055466	-0.936462	-0.667106
12	6	0	0.036600	-0.188132	0.063489
13	6	0	2.515843	-1.804581	0.293070
14	6	0	-4.010902	-1.943514	-0.672803
15	6	0	-3.904569	-0.138226	1.449939
16	6	0	-0.875195	1.024133	0.157820
17	6	0	3.192905	3.439494	0.305819
18	6	0	2.471654	1.935272	2.046895

19	6	0	4.757677	-2.067016	-0.536574
20	6	0	3.660997	-2.578245	0.145810
21	6	0	-4.890466	-2.053659	0.399654
22	6	0	-4.844608	-1.161801	1.465106
23	6	0	3.582808	-0.013015	-0.928390
24	6	0	4.146331	3.610564	2.514813
25	6	0	4.066651	4.057616	1.199321
26	1	0	3.411310	2.191969	3.960343
27	1	0	5.590493	-0.390574	-1.596536
28	1	0	1.687021	2.020462	-1.265055
29	1	0	-2.359875	-0.842504	-1.494710
30	1	0	1.664607	-2.203056	0.825685
31	1	0	-4.069853	-2.640988	-1.500801
32	1	0	-3.867332	0.586552	2.256707
33	1	0	3.138609	3.779544	-0.726273
34	1	0	1.851297	1.104242	2.373806
35	1	0	3.698162	-3.575837	0.568711
36	1	0	-5.545871	-1.255043	2.286773
37	1	0	3.576308	0.986776	-1.345818
38	1	0	4.830876	4.085348	3.210498
39	1	0	4.689001	4.880905	0.863206
40	6	0	-4.057129	3.297993	-0.858533
41	6	0	-3.856989	4.627817	-0.818892
42	1	0	-4.684507	2.716583	-0.191756
43	1	0	-4.297790	5.331639	-0.123168
44	7	0	-2.123248	1.074602	0.393708
45	6	0	-0.028352	2.213585	0.008627
46	6	0	-0.461383	3.471892	0.047790
47	1	0	0.219828	4.310222	-0.071076
48	1	0	-1.517488	3.675572	0.209533
49	6	0	-3.268860	2.723035	-1.958530
50	1	0	-3.252970	1.667674	-2.208926
51	6	0	-2.589097	3.705470	-2.576516
52	1	0	-1.920375	3.601342	-3.422568
53	6	0	-2.904494	5.018424	-1.915150
54	1	0	-2.003368	5.510981	-1.522907
55	1	0	-3.358756	5.730730	-2.617809

TS2d·*exo*

Center Number	Atomic Number	Atomic Type	Coordinates (Angstroms) X	Y	Z
1	35	0	6.204888	-3.297642	-0.585304
2	35	0	-6.380442	-3.201586	0.271813
3	6	0	3.496429	2.724526	2.676396
4	8	0	-0.338149	-1.336654	0.474602
5	7	0	1.276850	0.244042	-0.050841
6	6	0	2.406262	-0.593663	-0.169744
7	6	0	-3.059043	0.099803	0.379231
8	6	0	2.416505	2.376514	0.544338
9	6	0	4.625487	-0.974326	-1.067932
10	6	0	1.359543	1.690681	-0.300015
11	6	0	-3.028212	-0.914392	-0.590158
12	6	0	-0.003541	-0.191044	0.241969
13	6	0	2.472298	-1.827727	0.491037
14	6	0	-4.012044	-1.893381	-0.625343
15	6	0	-4.124726	0.127319	1.289294
16	6	0	-0.873606	1.046972	0.257397
17	6	0	3.215295	3.371880	-0.014254
18	6	0	2.559592	2.056904	1.896026
19	6	0	4.668217	-2.199808	-0.416426
20	6	0	3.600220	-2.629326	0.361150
21	6	0	-5.037775	-1.861360	0.314273
22	6	0	-5.104514	-0.858133	1.274392
23	6	0	3.495301	-0.175588	-0.944455
24	6	0	4.292647	3.723900	2.114978
25	6	0	4.149643	4.048372	0.769472
26	1	0	3.606928	2.466428	3.724965
27	1	0	5.464429	-0.643021	-1.669371
28	1	0	1.582555	1.868350	-1.362936
29	1	0	-2.224712	-0.933977	-1.319769
30	1	0	1.643372	-2.160888	1.098640

31	1	0	-3.984240	-2.676030	-1.375367
32	1	0	-4.164490	0.932168	2.016370
33	1	0	3.110316	3.616719	-1.069246
34	1	0	1.937346	1.278518	2.331175
35	1	0	3.647439	-3.584224	0.872593
36	1	0	-5.917067	-0.841788	1.992340
37	1	0	3.474007	0.778812	-1.457520
38	1	0	5.024089	4.243589	2.725723
39	1	0	4.768916	4.821175	0.324943
40	6	0	-3.879556	3.180056	-1.039138
41	6	0	-3.664482	4.511984	-0.993110
42	1	0	-4.754276	2.653151	-0.675203
43	1	0	-4.328204	5.255750	-0.568319
44	7	0	-2.163548	1.178067	0.402401
45	6	0	-0.040644	2.153930	0.018156
46	6	0	-0.566381	3.420964	-0.157966
47	1	0	0.110032	4.244053	-0.381032
48	1	0	-1.447863	3.676553	0.431161
49	6	0	-2.723423	2.526774	-1.617554
50	1	0	-2.710963	1.503470	-1.969543
51	6	0	-1.715431	3.472732	-1.828754
52	1	0	-0.924802	3.317750	-2.555360
53	6	0	-2.352696	4.843500	-1.643205
54	1	0	-1.756155	5.569218	-1.082320
55	1	0	-2.528371	5.277289	-2.637823

PROD2d·*exo*

Center Number	Atomic Number	Atomic Type	Coordinates (Angstroms) X	Y	Z
1	35	0	6.553436	-2.742204	-0.396043
2	35	0	-5.719373	-3.566847	0.943143
3	6	0	2.009117	2.233939	3.245721
4	8	0	-0.215855	-1.265600	-1.109226
5	7	0	1.334117	0.395535	-0.589368
6	6	0	2.528675	-0.347724	-0.556751
7	6	0	-3.008888	-0.061046	-0.579957
8	6	0	1.870639	2.371096	0.838828
9	6	0	4.953885	-0.391938	-0.605690
10	6	0	1.319112	1.855002	-0.478938
11	6	0	-4.212439	-0.365013	-1.227980
12	6	0	0.066992	-0.111410	-0.851570
13	6	0	2.520253	-1.741501	-0.396958
14	6	0	-5.020735	-1.401027	-0.774087
15	6	0	-2.642605	-0.808626	0.546752
16	6	0	-0.856831	1.059123	-0.778389
17	6	0	2.650446	3.525433	0.865645
18	6	0	1.547146	1.729206	2.035374
19	6	0	4.925348	-1.771665	-0.458016
20	6	0	3.716144	-2.447589	-0.352950
21	6	0	-4.623289	-2.145330	0.329186
22	6	0	-3.436511	-1.856163	0.993192
23	6	0	3.759022	0.315882	-0.656516
24	6	0	2.789201	3.390660	3.270245
25	6	0	3.107662	4.036921	2.079528
26	1	0	1.760613	1.725743	4.172135
27	1	0	5.899811	0.132223	-0.682792
28	1	0	1.890144	2.296133	-1.308817
29	1	0	-4.519417	0.199708	-2.101748
30	1	0	1.581776	-2.270304	-0.315746
31	1	0	-5.949188	-1.632687	-1.283935
32	1	0	-1.733867	-0.561220	1.085717
33	1	0	2.906620	4.023540	-0.067020
34	1	0	0.938237	0.828337	2.011401
35	1	0	3.702719	-3.525162	-0.231968
36	1	0	-3.142583	-2.433104	1.863015
37	1	0	3.800828	1.392805	-0.767665
38	1	0	3.149489	3.783169	4.215874
39	1	0	3.717208	4.934981	2.091475
40	6	0	-3.846391	2.811583	-0.330845
41	6	0	-3.858852	4.142795	-0.351330
42	1	0	-4.469106	2.169514	0.283285

43	1	0	-4.499521	4.774844	0.254784
44	7	0	-2.213542	1.009480	-1.032225
45	6	0	-0.139825	2.179065	-0.624190
46	6	0	-0.768272	3.525049	-0.658900
47	1	0	-0.054993	4.296380	-0.970715
48	1	0	-1.127154	3.805246	0.341491
49	6	0	-2.892505	2.272518	-1.380837
50	1	0	-3.453673	2.090110	-2.308762
51	6	0	-1.959780	3.486950	-1.635762
52	1	0	-1.574303	3.466738	-2.658754
53	6	0	-2.891726	4.689696	-1.376351
54	1	0	-2.337784	5.567614	-1.026715
55	1	0	-3.425192	4.986599	-2.288814

RC2d·*endo´*

Center Number	Atomic Number	Atomic Type	Coordinates (Angstroms) X	Y	Z
1	6	0	0.472412	4.532520	-1.084927
2	6	0	1.510150	5.155620	-0.195468
3	6	0	2.600983	4.366873	-0.163873
4	6	0	2.371252	3.192430	-1.015503
5	6	0	1.139732	3.270785	-1.553012
6	6	0	0.341350	3.105640	2.077599
7	6	0	0.007543	1.917389	1.579345
8	6	0	-1.397488	1.423575	1.277065
9	7	0	-1.187637	0.038170	0.830894
10	6	0	0.136212	-0.342274	0.774167
11	6	0	0.956475	0.855929	1.224119
12	6	0	-2.291010	-0.805483	0.558209
13	6	0	-3.579675	-0.387968	0.919075
14	6	0	-4.684355	-1.190191	0.661697
15	6	0	-4.508278	-2.416589	0.035910
16	6	0	-3.241081	-2.843322	-0.338978
17	6	0	-2.133700	-2.043360	-0.083043
18	35	0	-6.011861	-3.513297	-0.317128
19	6	0	-2.100344	2.280101	0.239368
20	6	0	-1.974345	2.014238	-1.123967
21	6	0	-2.552861	2.867129	-2.060052
22	6	0	-3.260101	3.992621	-1.640508
23	6	0	-3.400283	4.253511	-0.279229
24	6	0	-2.828299	3.394945	0.657629
25	7	0	2.216682	0.952857	1.352636
26	6	0	3.124857	-0.006760	0.873262
27	6	0	4.132913	-0.456471	1.730814
28	6	0	5.106455	-1.337115	1.273198
29	6	0	5.092469	-1.729027	-0.060216
30	6	0	4.120892	-1.261819	-0.939935
31	6	0	3.135810	-0.404678	-0.469428
32	8	0	0.569778	-1.439028	0.483785
33	35	0	6.432323	-2.910196	-0.699060
34	1	0	-2.449691	2.652155	-3.119151
35	1	0	-5.675674	-0.856098	0.945823
36	1	0	-1.985352	1.416307	2.203211
37	1	0	2.367311	-0.033846	-1.141053
38	1	0	-1.152017	-2.384047	-0.374625
39	1	0	4.132192	-1.569807	-1.979441
40	1	0	4.139120	-0.118256	2.761718
41	1	0	-2.950087	3.593063	1.720405
42	1	0	-1.421866	1.138730	-1.454607
43	1	0	-3.111650	-3.799475	-0.833621
44	1	0	5.877084	-1.703233	1.942333
45	1	0	-3.744602	0.571365	1.393234
46	1	0	-3.708537	4.657935	-2.371335
47	1	0	-3.961106	5.120313	0.055726
48	1	0	3.094722	2.398279	-1.166810
49	1	0	0.679995	2.562983	-2.232820
50	1	0	-0.400749	3.865414	2.306443
51	1	0	1.384736	3.339357	2.258073
52	1	0	3.511876	4.548582	0.395891
53	1	0	1.372692	6.094500	0.328053
54	1	0	-0.460884	4.325892	-0.541650

55	1	0	0.198680	5.187662	-1.924080

TS2d·*endo´*

Center Number	Atomic Number	Atomic Type	Coordinates (Angstroms) X	Y	Z
1	6	0	-0.359150	4.208897	0.880322
2	6	0	-1.410057	4.176260	-0.213400
3	6	0	-2.421351	3.307524	0.215116
4	6	0	-1.960386	2.574816	1.356278
5	6	0	-0.717746	3.006543	1.696227
6	6	0	-0.471236	3.218094	-1.716047
7	6	0	-0.033205	1.963509	-1.313548
8	6	0	1.387005	1.473171	-1.129532
9	7	0	1.194550	0.089718	-0.682178
10	6	0	-0.133969	-0.302233	-0.662765
11	6	0	-0.945681	0.911429	-1.068260
12	6	0	2.291989	-0.776863	-0.485955
13	6	0	3.552272	-0.425407	-0.987469
14	6	0	4.650980	-1.253269	-0.790595
15	6	0	4.495146	-2.439798	-0.087310
16	6	0	3.256872	-2.800368	0.428550
17	6	0	2.157963	-1.971744	0.236177
18	35	0	5.989712	-3.575513	0.177346
19	6	0	2.237815	2.304521	-0.192148
20	6	0	2.308836	2.026462	1.172787
21	6	0	3.022546	2.866721	2.021864
22	6	0	3.671317	3.992104	1.513602
23	6	0	3.622000	4.260896	0.148028
24	6	0	2.915497	3.412285	-0.702958
25	7	0	-2.227615	1.061911	-1.228247
26	6	0	-3.195833	0.155929	-0.784748
27	6	0	-4.357069	0.041798	-1.564604
28	6	0	-5.412766	-0.770875	-1.172555
29	6	0	-5.324833	-1.453926	0.035283
30	6	0	-4.201065	-1.337447	0.846940
31	6	0	-3.142995	-0.537267	0.436316
32	8	0	-0.541451	-1.425287	-0.436359
33	35	0	-6.768252	-2.552929	0.592444
34	1	0	3.073916	2.642673	3.082975
35	1	0	5.621169	-0.972036	-1.184166
36	1	0	1.877612	1.457260	-2.114049
37	1	0	-2.263108	-0.453218	1.063438
38	1	0	1.196888	-2.256258	0.638273
39	1	0	-4.153374	-1.870077	1.790487
40	1	0	-4.410569	0.600122	-2.493850
41	1	0	2.887361	3.614144	-1.771746
42	1	0	1.797028	1.153146	1.568518
43	1	0	3.146091	-3.724348	0.985087
44	1	0	-6.298755	-0.864883	-1.790700
45	1	0	3.695268	0.500659	-1.531084
46	1	0	4.224195	4.648166	2.178298
47	1	0	4.138131	5.125333	-0.257590
48	1	0	-2.515895	1.777934	1.836727
49	1	0	-0.102087	2.619364	2.499071
50	1	0	0.264630	3.983677	-1.953900
51	1	0	-1.373989	3.234055	-2.319553
52	1	0	-3.405640	3.217967	-0.225872
53	1	0	-1.632666	5.059459	-0.803923
54	1	0	0.679019	4.242137	0.542847
55	1	0	-0.530649	5.110487	1.488337

PROD2d·*endo´*

Center Number	Atomic Number	Atomic Type	Coordinates (Angstroms) X	Y	Z
1	6	0	-2.620200	3.759148	0.433971
2	6	0	-2.096872	3.643640	-1.008280
3	6	0	-2.858948	2.406088	-1.562419

4	6	0	-4.186780	2.554252	-0.836410
5	6	0	-4.036557	3.263235	0.282291
6	7	0	-2.197853	1.121967	-1.187809
7	6	0	-0.834359	1.157059	-0.984946
8	6	0	-0.058204	2.250339	-0.978167
9	6	0	-0.574432	3.632554	-1.159812
10	6	0	0.035544	-0.058334	-0.913095
11	7	0	1.326960	0.412114	-0.718734
12	6	0	1.383718	1.872697	-0.784006
13	6	0	2.481824	-0.381351	-0.589459
14	6	0	3.744166	0.199243	-0.772177
15	6	0	4.901563	-0.556438	-0.627652
16	6	0	4.803161	-1.901878	-0.301836
17	6	0	3.561458	-2.495380	-0.112605
18	6	0	2.403074	-1.740586	-0.250690
19	35	0	6.379861	-2.937938	-0.107332
20	6	0	1.953422	2.508610	0.472128
21	6	0	1.534740	2.068085	1.729060
22	6	0	2.012649	2.683464	2.880438
23	6	0	2.904551	3.752598	2.784187
24	6	0	3.317149	4.200123	1.532788
25	6	0	2.842781	3.576781	0.378958
26	8	0	-0.302482	-1.220740	-1.026282
27	6	0	-2.972365	0.050281	-0.687611
28	6	0	-4.037013	-0.445892	-1.444064
29	6	0	-4.836076	-1.473066	-0.954612
30	6	0	-4.550388	-2.017917	0.291471
31	6	0	-3.490200	-1.545390	1.057719
32	6	0	-2.713487	-0.502453	0.569789
33	35	0	-5.629183	-3.427845	0.961226
34	1	0	1.689384	2.331029	3.854921
35	1	0	5.872671	-0.095993	-0.770265
36	1	0	1.983306	2.182699	-1.652022
37	1	0	-1.900393	-0.105854	1.171678
38	1	0	1.439291	-2.205589	-0.103363
39	1	0	-3.285539	-1.976601	2.031376
40	1	0	-4.233039	-0.028427	-2.427380
41	1	0	3.169159	3.920867	-0.600276
42	1	0	0.837521	1.235886	1.798504
43	1	0	3.493447	-3.545760	0.148106
44	1	0	-5.662609	-1.856502	-1.542279
45	1	0	3.840515	1.248787	-1.022705
46	1	0	3.276831	4.232335	3.683804
47	1	0	4.012298	5.029760	1.451144
48	1	0	-5.103156	2.079347	-1.166373
49	1	0	-4.821409	3.460907	1.005201
50	1	0	-0.122095	4.316066	-0.428944
51	1	0	-0.283810	4.008270	-2.150085
52	1	0	-2.944886	2.447396	-2.654271
53	1	0	-2.501612	4.497939	-1.560963
54	1	0	-2.045381	3.114328	1.116609
55	1	0	-2.551627	4.781780	0.817600

RC2d·*exo´*

Center Number	Atomic Number	Atomic Type	Coordinates (Angstroms) X	Y	Z
1	35	0	6.011894	-3.538160	0.229018
2	35	0	-6.383520	-3.029297	0.745952
3	6	0	2.467081	2.698205	2.233167
4	8	0	-0.600995	-1.460494	-0.448080
5	7	0	1.160473	0.016571	-0.797701
6	6	0	2.269805	-0.826775	-0.547378
7	6	0	-3.139982	-0.055178	-0.820324
8	6	0	2.014190	2.248462	-0.097367
9	6	0	4.673469	-1.160264	-0.591181
10	6	0	1.360550	1.420674	-1.192635
11	6	0	-3.199598	-0.379916	0.539254
12	6	0	-0.161812	-0.365698	-0.737117
13	6	0	2.114890	-2.121341	-0.028197
14	6	0	-4.165559	-1.259632	1.007840
15	6	0	-4.080043	-0.595418	-1.700974

16	6	0	-0.984122	0.833208	-1.180687
17	6	0	2.761876	3.372163	-0.449221
18	6	0	1.867482	1.916511	1.249543
19	6	0	4.500067	-2.440762	-0.084343
20	6	0	3.228660	-2.921321	0.199046
21	6	0	-5.069714	-1.817721	0.109511
22	6	0	-5.035002	-1.495841	-1.241971
23	6	0	3.563100	-0.357273	-0.819148
24	6	0	3.211328	3.822732	1.878880
25	6	0	3.353312	4.161498	0.534931
26	1	0	2.355360	2.426300	3.278113
27	1	0	5.667363	-0.784350	-0.805568
28	1	0	1.988766	1.450990	-2.090742
29	1	0	-2.485408	0.065078	1.225773
30	1	0	1.130856	-2.505054	0.192326
31	1	0	-4.214207	-1.514281	2.060680
32	1	0	-4.048723	-0.314842	-2.748561
33	1	0	2.895240	3.625038	-1.498854
34	1	0	1.296511	1.035677	1.531362
35	1	0	3.099902	-3.920965	0.598809
36	1	0	-5.753207	-1.933221	-1.926455
37	1	0	3.727952	0.643336	-1.197870
38	1	0	3.682067	4.428725	2.646463
39	1	0	3.936007	5.031796	0.249861
40	6	0	-2.509767	3.846062	0.496302
41	6	0	-2.204163	5.137931	-0.207057
42	1	0	-3.390675	3.930555	1.147915
43	1	0	-2.897592	5.644793	-0.867863
44	7	0	-2.246085	0.922809	-1.296817
45	6	0	-0.038888	1.893899	-1.546091
46	6	0	-0.365156	3.059013	-2.099097
47	1	0	0.379057	3.815430	-2.329443
48	1	0	-1.403213	3.283948	-2.323106
49	6	0	-1.252787	3.576060	1.271645
50	1	0	-1.103100	2.706134	1.901680
51	6	0	-0.367072	4.563658	1.045670
52	1	0	0.635507	4.633727	1.453316
53	6	0	-0.961393	5.538751	0.121947
54	1	0	-0.461268	6.434671	-0.229850
55	1	0	-2.727564	3.035687	-0.215342

TS2d·*exo*´

Center Number	Atomic Number	Atomic Type	Coordinates (Angstroms) X	Y	Z
1	35	0	6.702022	-2.726185	0.121918
2	35	0	-5.924177	-3.462186	0.761225
3	6	0	1.513825	2.838032	2.604572
4	8	0	-0.129570	-1.520793	-0.692969
5	7	0	1.430866	0.203104	-0.799221
6	6	0	2.635239	-0.498244	-0.579869
7	6	0	-2.893016	-0.204397	-0.687776
8	6	0	1.749863	2.460479	0.229255
9	6	0	5.045000	-0.441134	-0.280301
10	6	0	1.426542	1.653567	-1.019134
11	6	0	-2.697136	-0.868074	0.531111
12	6	0	0.159067	-0.349153	-0.844883
13	6	0	2.671415	-1.897039	-0.460932
14	6	0	-3.594642	-1.834909	0.962725
15	6	0	-4.023917	-0.513048	-1.451215
16	6	0	-0.777759	0.787052	-1.174442
17	6	0	2.387504	3.694671	0.105421
18	6	0	1.312829	2.035779	1.485477
19	6	0	5.059520	-1.823816	-0.163851
20	6	0	3.879924	-2.551011	-0.253457
21	6	0	-4.696694	-2.142505	0.169614
22	6	0	-4.920994	-1.492770	-1.037305
23	6	0	3.838725	0.217160	-0.486367
24	6	0	2.145444	4.075171	2.476626
25	6	0	2.582384	4.501880	1.225451
26	1	0	1.175640	2.497900	3.578341
27	1	0	5.966277	0.125897	-0.207951

28	1	0	2.136670	1.899921	-1.817459
29	1	0	-1.832176	-0.628518	1.142447
30	1	0	1.759903	-2.470191	-0.531758
31	1	0	-3.442401	-2.347386	1.905974
32	1	0	-4.183251	0.020858	-2.382843
33	1	0	2.733887	4.025568	-0.871391
34	1	0	0.815893	1.073590	1.588324
35	1	0	3.897678	-3.631570	-0.163722
36	1	0	-5.788753	-1.738995	-1.638930
37	1	0	3.851979	1.297448	-0.558450
38	1	0	2.299630	4.700415	3.350140
39	1	0	3.080055	5.460613	1.118870
40	6	0	-3.917020	2.812351	0.050119
41	6	0	-3.831401	4.205249	-0.517998
42	1	0	-4.524554	2.798376	0.965954
43	1	0	-4.681239	4.735647	-0.931236
44	7	0	-2.086467	0.864607	-1.131800
45	6	0	-0.000442	1.924261	-1.432956
46	6	0	-0.598942	3.146430	-1.652345
47	1	0	-0.000880	4.051816	-1.730554
48	1	0	-1.541605	3.140022	-2.189271
49	6	0	-2.484764	2.493125	0.385279
50	1	0	-2.220769	1.733304	1.110053
51	6	0	-1.684864	3.606432	0.104398
52	1	0	-0.764337	3.818324	0.640056
53	6	0	-2.575901	4.664125	-0.427735
54	1	0	-2.222395	5.638401	-0.748262
55	1	0	-4.372817	2.085925	-0.634299

PROD2d·*exo´*

Center Number	Atomic Number	Atomic Type	Coordinates (Angstroms) X	Y	Z
1	35	0	6.284404	-3.307161	-0.067573
2	35	0	-6.184375	-3.373497	0.049741
3	6	0	3.087792	3.333765	2.016002
4	8	0	-0.321023	-1.157184	0.714336
5	7	0	1.291056	0.182387	-0.307896
6	6	0	2.429911	-0.643454	-0.239236
7	6	0	-3.070062	0.126194	0.174311
8	6	0	2.276298	2.461083	-0.084784
9	6	0	4.731660	-1.061433	-0.888292
10	6	0	1.346096	1.539508	-0.856204
11	6	0	-4.191796	0.140246	1.012702
12	6	0	0.016799	-0.136831	0.144903
13	6	0	2.445394	-1.824537	0.518427
14	6	0	-5.119219	-0.894853	0.975656
15	6	0	-2.904577	-0.946216	-0.712000
16	6	0	-0.838770	1.038596	-0.194498
17	6	0	3.081138	3.372198	-0.766184
18	6	0	2.278244	2.449494	1.311340
19	6	0	4.725627	-2.229345	-0.138354
20	6	0	3.589773	-2.612428	0.562522
21	6	0	-4.923051	-1.956815	0.102448
22	6	0	-3.817887	-1.990615	-0.740730
23	6	0	3.588059	-0.274188	-0.938452
24	6	0	3.893273	4.244356	1.331280
25	6	0	3.887533	4.264252	-0.060245
26	1	0	3.091061	3.313521	3.101257
27	1	0	5.620426	-0.763337	-1.432997
28	1	0	1.658130	1.506309	-1.909180
29	1	0	-4.340744	0.957748	1.710249
30	1	0	1.565900	-2.125595	1.067475
31	1	0	-5.983799	-0.877120	1.629972
32	1	0	-2.058108	-0.959147	-1.390358
33	1	0	3.081913	3.381568	-1.854190
34	1	0	1.650460	1.738519	1.843468
35	1	0	3.593447	-3.523840	1.150186
36	1	0	-3.680053	-2.818405	-1.427276
37	1	0	3.614858	0.635008	-1.526422
38	1	0	4.524780	4.933445	1.882948
39	1	0	4.513883	4.968006	-0.599273

40	6	0	-3.832692	2.920042	-0.635088
41	6	0	-3.713437	4.423372	-0.706296
42	1	0	-4.805806	2.576551	-0.272931
43	1	0	-4.505375	5.060542	-1.085814
44	7	0	-2.156100	1.195170	0.196124
45	6	0	-0.083267	1.993908	-0.752325
46	6	0	-0.629501	3.350607	-1.028549
47	1	0	0.162902	4.105395	-1.072774
48	1	0	-1.153787	3.370199	-1.993622
49	6	0	-2.677916	2.563031	0.326873
50	1	0	-3.046805	2.695828	1.351598
51	6	0	-1.630001	3.684873	0.102908
52	1	0	-1.053488	3.861329	1.017286
53	6	0	-2.524107	4.843595	-0.275266
54	1	0	-2.181907	5.873325	-0.255527
55	1	0	-3.673299	2.457326	-1.619474

RC2d·*endo*´´

Center Number	Atomic Number	Atomic Type	Coordinates (Angstroms) X	Y	Z
1	6	0	4.302651	0.836973	0.611889
2	6	0	3.194147	0.846816	-0.240526
3	6	0	3.168384	-0.018876	-1.340510
4	6	0	4.213253	-0.905439	-1.560987
5	6	0	5.285013	-0.930427	-0.673820
6	6	0	5.339820	-0.067218	0.413742
7	7	0	2.213666	1.836503	-0.041822
8	6	0	0.964900	1.601064	-0.011930
9	6	0	-0.067208	2.646226	0.036475
10	6	0	-1.427639	1.974258	0.084756
11	7	0	-1.105609	0.544333	-0.025863
12	6	0	0.241945	0.263999	0.009014
13	6	0	0.160431	3.956723	0.019348
14	6	0	-2.373454	2.455289	-0.995499
15	6	0	-3.362488	3.385457	-0.680449
16	6	0	-4.204489	3.881490	-1.674552
17	6	0	-4.064911	3.439905	-2.987066
18	6	0	-3.080425	2.503616	-3.303851
19	6	0	-2.235768	2.015073	-2.312958
20	6	0	-2.119010	-0.437748	0.063984
21	6	0	-1.898302	-1.744801	-0.391243
22	6	0	-2.908605	-2.693970	-0.303623
23	6	0	-4.142898	-2.342219	0.229524
24	6	0	-4.383109	-1.048953	0.672777
25	6	0	-3.371906	-0.098760	0.588330
26	8	0	0.753415	-0.836201	0.081021
27	35	0	-5.517751	-3.640802	0.341150
28	35	0	6.706221	-2.151329	-0.967829
29	6	0	2.144318	1.870638	3.331584
30	6	0	1.619308	0.480533	3.111119
31	6	0	0.131514	0.683912	3.055581
32	6	0	-0.143516	1.989800	3.243960
33	6	0	1.113221	2.731994	3.409177
34	1	0	-2.971830	2.153308	-4.325453
35	1	0	-5.350825	-0.779169	1.080073
36	1	0	-1.875324	2.160943	1.069488
37	1	0	2.327345	0.009577	-2.025533
38	1	0	-0.939887	-2.019890	-0.807301
39	1	0	4.195014	-1.575774	-2.412924
40	1	0	4.336907	1.541477	1.435978
41	1	0	-3.478249	3.719422	0.348689
42	1	0	-1.470135	1.283064	-2.558006
43	1	0	-2.734715	-3.704407	-0.656096
44	1	0	6.189042	-0.088750	1.087440
45	1	0	-3.579952	0.908820	0.927288
46	1	0	-4.723540	3.819105	-3.761901
47	1	0	-4.972819	4.604620	-1.419981
48	1	0	-0.582777	-0.118712	2.906595
49	1	0	-0.651963	4.677517	0.026435
50	1	0	1.180072	4.328080	-0.006453
51	1	0	3.195277	2.112024	3.435947

52	1	0	1.177113	3.802102	3.570037
53	1	0	-1.129697	2.440649	3.285631
54	1	0	1.896977	-0.190371	3.936590
55	1	0	2.012733	0.012594	2.197365

TS2d·*endo´´*

Center Number	Atomic Number	Atomic Type	Coordinates (Angstroms) X	Y	Z
1	6	0	4.438932	0.942325	-0.794502
2	6	0	3.247592	0.411626	-0.271353
3	6	0	3.249870	-0.921140	0.167830
4	6	0	4.400331	-1.698035	0.060649
5	6	0	5.555206	-1.147895	-0.479637
6	6	0	5.587883	0.174373	-0.911985
7	7	0	2.193564	1.340793	-0.182245
8	6	0	0.897156	1.106024	-0.194164
9	6	0	0.022548	2.204113	-0.107238
10	6	0	-1.385075	1.733312	0.150871
11	7	0	-1.260652	0.283982	-0.033344
12	6	0	0.049039	-0.144149	-0.165275
13	6	0	0.509962	3.481069	0.023208
14	6	0	-2.405711	2.368046	-0.771074
15	6	0	-3.239351	3.377421	-0.294126
16	6	0	-4.142953	4.007462	-1.149044
17	6	0	-4.220126	3.622480	-2.484139
18	6	0	-3.389330	2.609037	-2.963715
19	6	0	-2.483225	1.987140	-2.112037
20	6	0	-2.376777	-0.573404	0.063174
21	6	0	-2.386640	-1.827150	-0.562044
22	6	0	-3.502287	-2.649359	-0.459769
23	6	0	-4.614160	-2.219100	0.253741
24	6	0	-4.628291	-0.972926	0.866009
25	6	0	-3.509659	-0.153917	0.770580
26	8	0	0.395555	-1.308140	-0.264523
27	35	0	-6.133659	-3.345348	0.387970
28	35	0	7.116075	-2.214651	-0.626972
29	6	0	1.573637	3.348269	1.905155
30	6	0	0.544109	2.828457	2.817662
31	6	0	0.848058	1.573438	3.186536
32	6	0	2.212613	1.203357	2.669161
33	6	0	2.546818	2.349896	1.745509
34	1	0	-3.448784	2.303939	-4.003758
35	1	0	-5.501920	-0.641594	1.415891
36	1	0	-1.652203	1.949606	1.196403
37	1	0	2.348375	-1.364883	0.566567
38	1	0	-1.523605	-2.159266	-1.121320
39	1	0	4.394462	-2.729188	0.396187
40	1	0	4.437106	1.978821	-1.117513
41	1	0	-3.185164	3.669346	0.752710
42	1	0	-1.834451	1.196828	-2.481910
43	1	0	-3.506582	-3.620129	-0.942732
44	1	0	6.496948	0.593009	-1.328957
45	1	0	-3.530934	0.816976	1.252165
46	1	0	-4.927512	4.106212	-3.150288
47	1	0	-4.789785	4.791642	-0.768285
48	1	0	0.247792	0.922387	3.811307
49	1	0	-0.163383	4.315923	0.202739
50	1	0	1.460739	3.703998	-0.448467
51	1	0	3.558081	2.545289	1.411694
52	1	0	1.786187	4.404787	1.786147
53	1	0	-0.351412	3.377818	3.090527
54	1	0	2.935932	1.195619	3.498390
55	1	0	2.269735	0.209887	2.213002

PROD2d·*endo´´*

Center Number	Atomic Number	Atomic Type	Coordinates (Angstroms) X	Y	Z

1	6	0	-2.976570	-0.243614	0.943925
2	6	0	-3.006441	0.127842	-0.401373
3	6	0	-3.741025	-0.629548	-1.310701
4	6	0	-4.449696	-1.748654	-0.883852
5	6	0	-4.406129	-2.102187	0.459719
6	6	0	-3.672417	-1.364539	1.382719
7	7	0	-2.302906	1.298062	-0.844724
8	6	0	-3.027934	2.565432	-0.566942
9	6	0	-2.137103	3.818607	-0.768652
10	6	0	-0.721473	3.756276	-0.179584
11	6	0	-0.173853	2.385842	-0.362598
12	6	0	-0.935102	1.317769	-0.624393
13	6	0	-2.237867	4.019917	-2.268976
14	6	0	-3.339501	3.446474	-2.749823
15	6	0	-4.110462	2.754299	-1.646430
16	6	0	-0.065263	0.115957	-0.784269
17	7	0	1.227505	0.545564	-0.511792
18	6	0	1.271018	1.985651	-0.253735
19	6	0	1.836544	2.340083	1.110914
20	6	0	2.633984	3.472778	1.263192
21	6	0	3.100731	3.839848	2.524721
22	6	0	2.774385	3.070736	3.637894
23	6	0	1.976330	1.936112	3.487922
24	6	0	1.504508	1.575410	2.230532
25	6	0	2.390920	-0.244108	-0.561901
26	6	0	2.329369	-1.646107	-0.576675
27	6	0	3.497848	-2.397474	-0.615538
28	6	0	4.732451	-1.761371	-0.631892
29	6	0	4.813813	-0.376070	-0.608642
30	6	0	3.647289	0.378024	-0.574968
31	8	0	-0.409207	-1.006780	-1.101317
32	35	0	6.323641	-2.792715	-0.680249
33	35	0	-5.363293	-3.630794	1.048133
34	1	0	1.722474	1.331667	4.353114
35	1	0	5.779320	0.116992	-0.617228
36	1	0	1.863305	2.485374	-1.033717
37	1	0	-3.731914	-0.350441	-2.359282
38	1	0	1.371132	-2.144179	-0.564770
39	1	0	-5.018473	-2.345889	-1.587494
40	1	0	-2.396826	0.348263	1.648375
41	1	0	2.895943	4.067483	0.390520
42	1	0	0.882970	0.691180	2.109287
43	1	0	3.442309	-3.480435	-0.628416
44	1	0	-3.648270	-1.662009	2.425025
45	1	0	3.732002	1.457694	-0.551061
46	1	0	3.142517	3.350526	4.619869
47	1	0	3.723812	4.721925	2.634043
48	1	0	-3.683559	3.493600	-3.777495
49	1	0	-0.084043	4.505138	-0.667524
50	1	0	-0.735063	4.016597	0.887071
51	1	0	-3.439453	2.526726	0.450206
52	1	0	-2.667616	4.649508	-0.280515
53	1	0	-1.525582	4.616252	-2.831260
54	1	0	-4.911851	3.405221	-1.269253
55	1	0	-4.573955	1.810172	-1.942843

RC2d·*exo´´*

Center Number	Atomic Number	Atomic Type	Coordinates (Angstroms) X	Y	Z
1	35	0	6.322501	-3.115503	-0.752547
2	35	0	-6.183808	-3.425889	0.409318
3	6	0	3.378602	2.553688	2.936134
4	8	0	-0.279025	-1.345587	0.192384
5	7	0	1.322962	0.305016	-0.099426
6	6	0	2.473166	-0.506024	-0.247267
7	6	0	-2.974458	-0.028939	0.418634
8	6	0	2.404705	2.389299	0.732949
9	6	0	4.731024	-0.775769	-1.082436
10	6	0	1.407234	1.770296	-0.224464
11	6	0	-3.040341	-0.935395	-0.646783
12	6	0	0.047331	-0.180440	0.083301

13	6	0	2.529423	-1.794244	0.300102
14	6	0	-3.995859	-1.942312	-0.653311
15	6	0	-3.901430	-0.127488	1.461630
16	6	0	-0.865409	1.030947	0.178427
17	6	0	3.203647	3.448795	0.307410
18	6	0	2.495155	1.943295	2.052694
19	6	0	4.765129	-2.056206	-0.546016
20	6	0	3.673935	-2.567390	0.145147
21	6	0	-4.881681	-2.047385	0.414600
22	6	0	-4.841846	-1.150764	1.476190
23	6	0	3.586067	-0.003410	-0.931465
24	6	0	4.173059	3.618356	2.509565
25	6	0	4.083749	4.066383	1.195026
26	1	0	3.448768	2.198658	3.959432
27	1	0	5.588898	-0.380520	-1.614223
28	1	0	1.687265	2.030154	-1.253550
29	1	0	-2.340750	-0.844499	-1.471278
30	1	0	1.682439	-2.192590	0.839581
31	1	0	-4.050374	-2.643385	-1.478559
32	1	0	-3.868659	0.600433	2.265892
33	1	0	3.141745	3.789679	-0.723972
34	1	0	1.877260	1.111923	2.383403
35	1	0	3.714917	-3.564484	0.568863
36	1	0	-5.547853	-1.240078	2.294212
37	1	0	3.575720	0.995878	-1.350017
38	1	0	4.862608	4.092712	3.200584
39	1	0	4.703460	4.890052	0.855013
40	6	0	-4.198731	3.320271	-0.938729
41	6	0	-3.822927	4.773692	-1.016062
42	1	0	-3.981577	2.887963	0.048397
43	1	0	-4.257120	5.545124	-0.391404
44	7	0	-2.114054	1.080760	0.410737
45	6	0	-0.019487	2.221329	0.031452
46	6	0	-0.452624	3.478807	0.079496
47	1	0	0.223772	4.320593	-0.040122
48	1	0	-1.506479	3.686581	0.241670
49	6	0	-3.340143	2.688691	-1.995495
50	1	0	-3.347611	1.628000	-2.218720
51	6	0	-2.584204	3.635762	-2.580857
52	1	0	-1.861303	3.483859	-3.374721
53	6	0	-2.889161	4.939658	-1.970741
54	1	0	-2.423033	5.875725	-2.258411
55	1	0	-5.270043	3.160193	-1.122083

TS2d·*exo´´*

Center Number	Atomic Number	Atomic Type	Coordinates (Angstroms) X	Y	Z
1	35	0	6.232565	-3.255064	-0.599416
2	35	0	-6.342382	-3.161585	0.417715
3	6	0	3.477479	2.702505	2.744088
4	8	0	-0.325739	-1.298162	0.357207
5	7	0	1.307446	0.291992	-0.077582
6	6	0	2.435881	-0.546766	-0.194702
7	6	0	-3.038813	0.149763	0.286073
8	6	0	2.437484	2.409885	0.584191
9	6	0	4.672553	-0.913255	-1.054625
10	6	0	1.399380	1.742610	-0.297075
11	6	0	-3.031689	-0.899217	-0.642925
12	6	0	0.017167	-0.146232	0.170793
13	6	0	2.483416	-1.797606	0.435454
14	6	0	-4.011400	-1.882390	-0.606075
15	6	0	-4.064640	0.205705	1.235828
16	6	0	-0.843112	1.092326	0.194126
17	6	0	3.240073	3.424919	0.068007
18	6	0	2.558695	2.052120	1.928307
19	6	0	4.696842	-2.155393	-0.434511
20	6	0	3.610859	-2.600311	0.308847
21	6	0	-5.004177	-1.818078	0.367652
22	6	0	-5.040831	-0.783684	1.293878
23	6	0	3.542850	-0.113243	-0.934757
24	6	0	4.277556	3.721472	2.225453

25	6	0	4.156573	4.083267	0.887189
26	1	0	3.570799	2.415243	3.786709
27	1	0	5.525465	-0.569817	-1.628990
28	1	0	1.641013	1.942114	-1.351559
29	1	0	-2.249115	-0.948833	-1.393205
30	1	0	1.640410	-2.142914	1.016387
31	1	0	-4.005158	-2.694098	-1.324993
32	1	0	-4.078233	1.033702	1.937725
33	1	0	3.151873	3.699651	-0.981053
34	1	0	1.934155	1.257714	2.329804
35	1	0	3.643660	-3.568306	0.796238
36	1	0	-5.825750	-0.744559	2.040785
37	1	0	3.536680	0.854437	-1.422830
38	1	0	4.994967	4.227507	2.863677
39	1	0	4.779112	4.871694	0.475952
40	6	0	-4.041952	2.827224	-1.290255
41	6	0	-3.835388	4.318873	-1.240851
42	1	0	-4.431634	2.407410	-0.354520
43	1	0	-4.605873	5.019891	-0.942223
44	7	0	-2.145506	1.241890	0.245113
45	6	0	-0.006422	2.201591	0.004931
46	6	0	-0.541100	3.463401	-0.125367
47	1	0	0.092157	4.313404	-0.370243
48	1	0	-1.441921	3.677367	0.439834
49	6	0	-2.676161	2.304809	-1.650555
50	1	0	-2.540606	1.335714	-2.113329
51	6	0	-1.808871	3.385591	-1.847745
52	1	0	-0.951707	3.340326	-2.509894
53	6	0	-2.585975	4.624631	-1.616326
54	1	0	-2.160087	5.620032	-1.684346
55	1	0	-4.761946	2.556041	-2.075024

PROD2d·*exo´´*

Center Number	Atomic Number	Atomic Type	Coordinates (Angstroms) X	Y	Z
1	35	0	6.552761	-2.736329	-0.388205
2	35	0	-5.688342	-3.571506	0.952260
3	6	0	2.014337	2.256473	3.230286
4	8	0	-0.216100	-1.254398	-1.106208
5	7	0	1.337150	0.406909	-0.597739
6	6	0	2.530750	-0.337966	-0.561074
7	6	0	-2.996143	-0.062223	-0.587032
8	6	0	1.873778	2.387707	0.823101
9	6	0	4.956084	-0.384653	-0.603403
10	6	0	1.324167	1.866663	-0.493556
11	6	0	-4.181155	-0.394900	-1.253307
12	6	0	0.069376	-0.098982	-0.857176
13	6	0	2.520528	-1.731655	-0.400480
14	6	0	-4.985118	-1.431872	-0.794903
15	6	0	-2.639515	-0.782440	0.559054
16	6	0	-0.853169	1.073617	-0.794918
17	6	0	2.646220	3.547124	0.847228
18	6	0	1.555059	1.746272	2.021152
19	6	0	4.925819	-1.764273	-0.455022
20	6	0	3.715664	-2.438856	-0.352774
21	6	0	-4.598512	-2.148432	0.330985
22	6	0	-3.428226	-1.832167	1.011284
23	6	0	3.762090	0.324321	-0.657760
24	6	0	2.786860	3.418265	3.252100
25	6	0	3.100610	4.064047	2.059844
26	1	0	1.769859	1.748372	4.157834
27	1	0	5.902701	0.138576	-0.678251
28	1	0	1.897576	2.303312	-1.324152
29	1	0	-4.473610	0.150035	-2.145038
30	1	0	1.581377	-2.259528	-0.321525
31	1	0	-5.900132	-1.687163	-1.317559
32	1	0	-1.742175	-0.513568	1.107391
33	1	0	2.898783	4.045035	-0.086544
34	1	0	0.952650	0.841014	1.999365
35	1	0	3.700785	-3.516351	-0.231259
36	1	0	-3.143192	-2.389674	1.896592

37	1	0	3.805459	1.401144	-0.769006
38	1	0	3.145098	3.815019	4.196729
39	1	0	3.704330	4.966052	2.069653
40	6	0	-3.876537	2.774610	-0.263441
41	6	0	-3.947985	4.248936	-0.580671
42	1	0	-3.459560	2.599409	0.739030
43	1	0	-4.730187	4.898307	-0.201750
44	7	0	-2.210482	1.015396	-1.047732
45	6	0	-0.134246	2.193332	-0.642697
46	6	0	-0.756046	3.544995	-0.667944
47	1	0	-0.046825	4.311698	-0.999578
48	1	0	-1.086465	3.834950	0.339020
49	6	0	-2.902041	2.276290	-1.352483
50	1	0	-3.482308	2.134144	-2.271961
51	6	0	-1.975383	3.491456	-1.618557
52	1	0	-1.604440	3.470943	-2.648237
53	6	0	-2.927720	4.635131	-1.347285
54	1	0	-2.734129	5.648060	-1.685480
55	1	0	-4.845437	2.268605	-0.302547

RC2d·*endo*´´´

Center Number	Atomic Number	Atomic Type	Coordinates (Angstroms) X	Y	Z
1	6	0	-3.764604	0.104351	0.500386
2	6	0	-2.536957	-0.568749	0.582824
3	6	0	-2.511506	-1.959143	0.392073
4	6	0	-3.687939	-2.648713	0.124436
5	6	0	-4.893781	-1.964069	0.046674
6	6	0	-4.938451	-0.589735	0.234392
7	7	0	-1.365302	0.165549	0.879677
8	6	0	-0.077742	-0.326467	0.850614
9	6	0	0.829784	0.803016	1.319184
10	6	0	-0.048525	1.902916	1.736124
11	6	0	-1.438623	1.596242	1.210265
12	6	0	0.322721	2.972502	2.433221
13	7	0	2.095570	0.814103	1.424536
14	6	0	2.916191	-0.207223	0.911444
15	6	0	3.891211	-0.761030	1.745245
16	6	0	4.779648	-1.710865	1.253816
17	6	0	4.716728	-2.067792	-0.087960
18	6	0	3.779252	-1.497732	-0.944107
19	6	0	2.877850	-0.570428	-0.440369
20	8	0	0.270854	-1.456318	0.573030
21	6	0	-1.759113	2.465804	0.002802
22	6	0	-2.352201	3.713185	0.201067
23	6	0	-2.567226	4.568960	-0.876867
24	6	0	-2.197658	4.177846	-2.162605
25	6	0	-1.618012	2.926903	-2.364705
26	6	0	-1.398177	2.073627	-1.286058
27	35	0	5.947443	-3.338772	-0.771414
28	35	0	-6.492372	-2.912989	-0.318418
29	6	0	2.330276	4.552206	0.320674
30	6	0	1.389393	3.944724	-0.631701
31	6	0	2.034042	3.006030	-1.348865
32	6	0	3.472005	2.948849	-0.913840
33	6	0	3.540065	3.982623	0.173316
34	1	0	-1.333733	2.613421	-3.364389
35	1	0	-5.880483	-0.057128	0.170616
36	1	0	-2.191290	1.746562	1.992034
37	1	0	2.141440	-0.110283	-1.093087
38	1	0	-1.579342	-2.499222	0.454079
39	1	0	3.753184	-1.779056	-1.990923
40	1	0	3.938252	-0.449643	2.783493
41	1	0	-2.646613	4.018284	1.202890
42	1	0	-0.944740	1.099769	-1.452610
43	1	0	-3.660504	-3.723002	-0.020096
44	1	0	5.523841	-2.157501	1.903592
45	1	0	-3.822822	1.178169	0.626162
46	1	0	-2.366500	4.842444	-3.003749
47	1	0	-3.028151	5.537822	-0.712546
48	1	0	-0.380590	3.755018	2.703192

49	1	0	1.358361	3.084686	2.737911
50	1	0	4.439693	4.211810	0.731809
51	1	0	2.068833	5.334603	1.025277
52	1	0	4.156639	3.182993	-1.741384
53	1	0	3.760857	1.953736	-0.549928
54	1	0	0.344267	4.222056	-0.724185
55	1	0	1.611342	2.392054	-2.136634

TS2d·*endo´´´*

Center Number	Atomic Number	Atomic Type	Coordinates (Angstroms) X	Y	Z
1	6	0	3.702520	-0.279319	-0.911699
2	6	0	2.443631	-0.746422	-0.504724
3	6	0	2.362824	-1.992991	0.135841
4	6	0	3.509969	-2.749521	0.345035
5	6	0	4.744555	-2.271949	-0.073917
6	6	0	4.847275	-1.037460	-0.699654
7	7	0	1.304429	0.058233	-0.724660
8	6	0	-0.013744	-0.359193	-0.613956
9	6	0	-0.867992	0.804038	-1.058452
10	6	0	-0.000183	1.825199	-1.478149
11	6	0	1.434821	1.434243	-1.210372
12	6	0	-0.498590	3.040578	-1.885858
13	7	0	-2.168426	1.005594	-1.098118
14	6	0	-3.165659	0.103942	-0.682858
15	6	0	-4.383790	0.215673	-1.371992
16	6	0	-5.478672	-0.571171	-1.043365
17	6	0	-5.364235	-1.469697	0.012313
18	6	0	-4.181791	-1.586009	0.732651
19	6	0	-3.086871	-0.800127	0.387788
20	8	0	-0.378439	-1.473385	-0.285088
21	6	0	2.144873	2.361320	-0.240620
22	6	0	2.698296	3.546479	-0.727048
23	6	0	3.268811	4.469692	0.147229
24	6	0	3.307605	4.201743	1.513897
25	6	0	2.779754	3.005859	1.999129
26	6	0	2.197736	2.091373	1.126439
27	35	0	-6.850953	-2.548764	0.483181
28	35	0	6.304403	-3.309162	0.217938
29	6	0	-1.574266	3.798842	-0.186066
30	6	0	-0.597007	3.790205	0.914659
31	6	0	-0.967391	2.898514	1.845696
32	6	0	-2.310819	2.316185	1.500499
33	6	0	-2.566117	2.855562	0.116134
34	1	0	2.817620	2.786008	3.061688
35	1	0	5.812864	-0.662850	-1.020184
36	1	0	1.984182	1.424838	-2.161327
37	1	0	-2.164190	-0.910505	0.941266
38	1	0	1.407469	-2.372920	0.463361
39	1	0	-4.113754	-2.286609	1.557446
40	1	0	-4.447600	0.932396	-2.184908
41	1	0	2.683696	3.749835	-1.795816
42	1	0	1.772108	1.167697	1.508654
43	1	0	3.436789	-3.712083	0.839039
44	1	0	-6.409488	-0.486448	-1.592787
45	1	0	3.811155	0.686591	-1.388983
46	1	0	3.757418	4.915703	2.196571
47	1	0	3.690827	5.391396	-0.240945
48	1	0	0.166771	3.871857	-2.104702
49	1	0	-1.455517	3.029273	-2.396524
50	1	0	-3.550968	2.848612	-0.333629
51	1	0	-1.763777	4.670830	-0.802340
52	1	0	-3.084438	2.710223	2.176891
53	1	0	-2.366088	1.226861	1.590585
54	1	0	0.317462	4.376099	0.917525
55	1	0	-0.411055	2.637384	2.738605

PROD2d·*endo´´´*

Center Number	Atomic Number	Atomic Type	Coordinates (Angstroms) X	Y	Z
1	6	0	3.428861	-0.483543	-0.982625
2	6	0	2.257475	-0.688933	-0.239962
3	6	0	2.198429	-1.778728	0.642273
4	6	0	3.282888	-2.639208	0.765127
5	6	0	4.433303	-2.418534	0.019153
6	6	0	4.513379	-1.342351	-0.853672
7	7	0	1.181579	0.206443	-0.384906
8	6	0	-0.116248	0.008393	0.067634
9	6	0	-0.880392	1.219880	-0.346975
10	6	0	-0.067329	2.063208	-0.993639
11	6	0	1.326469	1.501072	-1.056465
12	6	0	-0.505982	3.403729	-1.467047
13	7	0	-2.191723	1.446862	0.031558
14	6	0	-3.085958	0.325968	0.012030
15	6	0	-3.352639	-0.339770	-1.185295
16	6	0	-4.236889	-1.412730	-1.215210
17	6	0	-4.857278	-1.803605	-0.033604
18	6	0	-4.605034	-1.154499	1.169510
19	6	0	-3.710716	-0.088646	1.186412
20	8	0	-0.539085	-0.954711	0.679231
21	6	0	2.324225	2.420512	-0.373021
22	6	0	3.244837	3.145473	-1.126594
23	6	0	4.117536	4.037821	-0.504067
24	6	0	4.071805	4.205073	0.876575
25	6	0	3.148395	3.482558	1.633745
26	6	0	2.274422	2.598467	1.011090
27	35	0	-6.070681	-3.261372	-0.063924
28	35	0	5.911226	-3.594102	0.197234
29	6	0	-1.748503	3.826616	-0.673834
30	6	0	-1.498360	4.343847	0.730493
31	6	0	-2.534714	4.075598	1.523066
32	6	0	-3.604205	3.313070	0.769794
33	6	0	-2.805912	2.724204	-0.409806
34	1	0	3.109349	3.610763	2.710936
35	1	0	5.413651	-1.171426	-1.433123
36	1	0	1.631196	1.348366	-2.101077
37	1	0	-3.470805	0.413883	2.117769
38	1	0	1.306383	-1.952688	1.225357
39	1	0	-5.090604	-1.485112	2.080645
40	1	0	-2.857026	-0.016409	-2.097134
41	1	0	3.282778	3.009520	-2.205629
42	1	0	1.548824	2.039213	1.597300
43	1	0	3.229515	-3.479268	1.448816
44	1	0	-4.443642	-1.937942	-2.140892
45	1	0	3.511915	0.351779	-1.667056
46	1	0	4.753312	4.894950	1.364179
47	1	0	4.834055	4.595746	-1.098646
48	1	0	0.290908	4.145977	-1.331079
49	1	0	-0.728788	3.379936	-2.541728
50	1	0	-3.425757	2.538606	-1.296530
51	1	0	-2.262954	4.615607	-1.242066
52	1	0	-4.379368	3.998598	0.399120
53	1	0	-4.108711	2.542584	1.358046
54	1	0	-0.609357	4.907043	0.999207
55	1	0	-2.641421	4.386762	2.556795
